# Supplementary material for: Manchester Intermittent Diet in Gestational Diabetes Acceptability Study (MIDDAS-GDM): a two-arm randomised feasibility protocol trial of an intermittent low-energy diet (ILED) in women with gestational diabetes and obesity in Greater Manchester
Source: BMJ Open. 2024 Feb 10;14(2):e078264. doi: 10.1136/bmjopen-2023-078264 (PMC10862275; doi:10.1136/bmjopen-2023-078264)
Supplement: Supplementary data [file bmjopen-2023-078264supp001.pdf]

Manchester University NHS Foundation Trust
The University of Manchester
NIHR | National Institute for Health Research

Consultant Endocrinologist – Dr. Basil Issa
Research Dietitian – Dr. Michelle Harvie
Email: mft.middas.gdm@nhs.net
Tel: 07815987910

1st Floor Education & Research Centre
Manchester University NHS Foundation Trust
Wythenshawe Hospital
Manchester
M23 9LT

MIDDAS-GDM

Manchester Intermittent Diet in Gestational Diabetes Acceptability Study

Participant information sheet

We would like to invite you to take part in a research study **that is testing two different diet programmes which aim to help people with gestational diabetes control their blood sugars.**

If you decide to take part:

- You will be assigned to one of two diet programmes for the duration of your pregnancy. One involves following the standard NHS healthy diet recommendations for pregnancy, and the other follows the standard NHS healthy diet for 5 days/week plus two non-consecutive calorie restricted days of 1,000 kcal per week (both groups will be encouraged to be physically active).

- You will be asked to attend your routine appointments at Wythenshawe or St Marys Hospital and will have fortnightly appointments until delivery of your baby (some appointments may be virtual depending on COVID-19 restrictions). You will be asked to attend the hospital for a blood test 12 weeks after having your baby.

- You will be supported by a diabetes specialist dietitian, midwife, consultant endocrinologist, and your obstetric team throughout the study to help manage your pregnancy and blood glucose safely.

- Throughout the study you will be asked to monitor your food intake via a smartphone/tablet app, or on paper if you prefer, and you will receive feedback on this during your dietary reviews. Comprehensive dietary advice and recipes will be provided.

- Throughout the study you will be asked to monitor your blood sugar using a blood sugar meter four times a day, and you will also be asked to monitor your ketone levels two times on two days of the week (ketones indicate how well your body is using sugar or fat as an energy source). You will be taught how to check your blood sugar and ketone levels.

- If you would like to take part, or you have any questions, then please contact **mft.middas.gdm@nhs.uk**

Dapre E, *et al. BMJ Open* 2024; 14:e078264. doi: 10.1136/bmjopen-2023-078264

This study is being carried out by a team of trained dietitians, doctors, nurses, midwives and researchers under the supervision of Dr. Basil Issa and Dr. Michelle Harvie at Wythenshawe and St Marys hospitals (Manchester University NHS Foundation Trust, MFT).

Before you decide if you would like to take part, it is important for you to understand why the research is being done and what taking part would involve for you. Please take your time to read the following information carefully. Discuss it with your friends, relatives, or GP if you wish to. Take time to consider whether or not you wish to take part.

Please ring the research team at the number at the top of the first page, or e-mail mft.middas.gdm@nhs.net if there is anything that is not clear, or if you would like more information. You can attend an information session about the diets and the study before agreeing to take part if you would like to.

Your participation in the study is entirely voluntary; you do not have to take part if you do not want to and you can opt out of the study at any time without giving a reason. Thank you for reading this information. We hope this research will be of interest to you.

Why are we doing this research?

Around 1 in 8 pregnant women can develop gestational diabetes. This condition causes risks to mother and baby from high blood sugar, high blood pressure, induced labours, caesarean-sections, and larger babies. Women often need medication to control blood sugar despite following recommended NHS healthy eating plans for pregnancy. Intermittent low-calorie diets (two non-consecutive days over the course of the week) improve blood sugar control and reduce the need for medication in patients with type 2 diabetes. We want to find out whether intermittent low-calorie diets might also improve blood sugar control in gestational diabetes and reduce the need for medication as it is a similar condition to type 2 diabetes.

What is the purpose of this research?

This study aims to assess the acceptability (to you) and safety of an intermittent low-calorie diet compared to the usual recommended NHS healthy eating and lifestyle plan for gestational diabetes. **A computer system will randomly allocate you to one of the two diets**. We want to find out which diet is most acceptable to women, whether there is any difference in the two diets' effect on blood sugar control, and any side effects experienced by women. The findings of this study will inform a larger study which will be designed to more closely compare the effect of the two diets on blood sugar control in women with gestational diabetes.

Why have I been asked to take part?

You have been invited to take part in this study because you have been diagnosed with gestational diabetes. We hope to recruit around 48 people to take part in this study.

Dapre E, *et al. BMJ Open* 2024; 14:e078264. doi: 10.1136/bmjopen-2023-078264

NHS Manchester University NHS Foundation Trust

MANCHESTER 1824 The University of Manchester

NIHR | National Institute for Health Research

What happens if you agree to take part?

If you agree to take part you will be randomly allocated via a computer system to one of two diet and lifestyle programmes for the remaining weeks of your pregnancy.

Best NHS Care Healthy Diet programme

You will receive personalised advice from a specialist dietician. Recommendations will include increased fruit/vegetable intake, low glycaemic index starchy foods (i.e starchy foods which are slowly absorbed and take a while to raise your blood sugar level), reducing refined sugar, and having regular mealtimes. You will be advised how to design your diet to include the right amount of protein, fats, carbohydrates, and fibre, and will be given meal plans and recipes. You will also be advised to try to complete 150 minutes of moderate intensity exercise a week.

Intermittent Low-Calorie Diet programme

If you are allocated to this group you will receive personalised advice to follow a low-calorie diet of 1,000 kcal on two non-consecutive days of the week and the NHS healthy diet on the other five days of the week. The 1,000 kcal days include a set number of portions of protein, carbohydrates and fat foods, fruits, vegetables and dairy/dairy alternatives typically including ~210g (7 oz) of lean protein foods and 3-4 portions of wholegrain carbohydrates, 5 portions of vegetables, 2 of fruit, and 3 of dairy or dairy alternatives and a small amount of healthy fat. You will also be advised to try to complete 150 minutes of moderate intensity exercise a week.

Monitoring

You will have all your usual routine antenatal appointments including checks on your weight, blood pressure, blood tests and ultrasound scans. Extra blood tests will be done as part of the study, and these will be added on to samples taken during your routine blood tests.

You will be asked to monitor your blood sugar at home four times a day until your baby is born which is usual care in the clinic. In addition, you will be asked to record ketone levels on two days of the week (you will be taught how to do this using a finger prick machine). The results will be recorded when you attend clinic.

When babies are born to mothers with gestational diabetes it is normal that their birth weight is recorded and that their blood sugar is monitored for 12 hours following delivery; these results will be recorded by the research team.

You will be asked to attend an additional glucose tolerance test at the hospital 12 weeks after delivery to assess whether you have any residual diabetes (95% of women do not) and also to assess how sensitive your body is to insulin (an important risk factor for the development of diabetes in the future). You will be asked to attend at 9:00am having fasted (no food or drink apart from water) from midnight. A blood sample (around 10 mL/2 teaspoons) will be taken for glucose and insulin and you will be asked to drink a sugary drink with 75 grams of glucose. A further blood sample will be taken after 2 hours for glucose and insulin. You will need to

Dapre E, *et al. BMJ Open* 2024; 14:e078264. doi: 10.1136/bmjopen-2023-078264

remain in hospital during this time. The reason for this is to help us to understand whether there could be any difference in the body's ability to process sugar between the two diet groups, and also to find out whether any women still have signs of diabetes after pregnancy.

Any blood samples taken as part of the study will be identifiable only using your study identification number and will have none of your personal details. Part of the sample will be sent for immediate analysis and any remaining will be stored securely, accessible only by the research team. At the end of the study any left over samples will be disposed of in accordance with the Human Tissue Act (2004).

You will be asked to record your food intake via the Libro smartphone/tablet app or in a paper diary for four days once a month throughout the study. You will also be asked to complete three questionnaires to assess your wellbeing and level of physical activity in these weeks, and a final end of study questionnaire at the final appointment.

Ongoing support from a specialist team of healthcare professionals

Your specialist team includes a Consultant Endocrinologist, Consultant Obstetrician, diabetes specialist dietitian, midwives, and a GP trainee with a special interest in women's health. The specialist team work closely with the usual obstetric teams involved in your care. Reviews with the team will be either face to face when you attend clinic or remotely using video calls.

Mobile Applications and Glucose Meters

You will be given the option of using a smartphone application called 'Libro' to record your dietary intake during the study. We will ask you to record 4 days of food and drink intake once a month across the study. Your diaries will be viewed by your allocated dietitian who will provide personalised dietary feedback via the app. You are also free to record more days of your diet should you wish, which some people find helpful. If you do not want to use the mobile app you can use paper instead. You will be supported to set up and use the Nutritics Libro App at your appointments. You do not have to use the application to be part of the study.

Your blood sugar will be monitored using a glucose monitoring device which checks your blood sugar using a 'fingerprick' blood test. You will be shown how to do this yourself. With your permission the research team will make a note of your glucose readings at every visit, either by checking your glucose monitoring device, or by uploading your glucose meter readings onto the computer if you are using a mobile application.

The self-monitoring schedule is as follows:

**Intermittent low-energy diet monitoring**		**Best NHS care monitoring**	
Ketones (low kcal days)	Glucose	Ketones (2 days/wk)	Glucose
Fasting before breakfast the morning after each of the low-calorie days	Fasting (morning)	Fasting before breakfast on 2 non-consecutive days/weeks	Fasting (morning)
	1hr post breakfast		1hr post breakfast
	1hr post lunch		1hr post lunch

Dapre E, et al. BMJ Open 2024; 14:e078264. doi: 10.1136/bmjopen-2023-078264

NHS
Manchester University
NHS Foundation Trust

MANCHESTER 1824
The University of Manchester

NIHR | National Institute for Health Research

1 hour post evening meal on each of the low-calorie days	1hr post dinner	1 hour post evening meal on 2 non-consecutive days/week	1hr post dinner

What should I do if my blood glucose or ketones are out of range?

Low Blood Sugar

It is unlikely that your blood sugar levels will drop too low as a result of being on the intermittent low calorie or the NHS standard diets. You are advised to take 15-20g of ‘rapid acting’ carbohydrate if your blood glucose is <4 mmol/L). Examples include 170-225ml Lucozade Original (not Lucozade Sport), a small carton of fruit juice, 5-6 glucose tablets, 4/5 jelly babies, or a small tin of cola (150-200ml). You will need to repeat the treatment every 15 minutes until your blood glucose is ≥4 mmol/l.

The following table highlights when you need to consider an additional slower acting carbohydrate:

Situation	Acceptable slow acting carbohydrate
Less than 1 hour before the next meal	Try and avoid
1-2 hour before the next meal	10g (eg half of one of the items below)
More than 2 hours before the next meal	15-20g (eg slice of toast, piece of fruit, small bowl of cereal, glass of milk)

Raised Ketones

It is unlikely that your ketone levels will rise significantly as a result of being on the intermittent low calorie or NHS standard diets.

If your ketone levels are ⩾1.0 mmol/L on a fasting sample:

- Drink 1L fluids and repeat ketone levels after 4 hours.
- If your ketone level has improved (<1.0mmol/L), no further action is required.
- If your ketone level has increased or remains the same, repeat your ketone level after 2 hours.
- If your ketone level is persistently increased, consume 40g carbohydrates (eg one bagel, bowl of cereal and a banana, small jacket potato), and repeat in 2 hours.
- Continue to do this until your ketone levels are <1.0mmol/L.

Make Immediate Contact with the research team if:

Blood glucose	• Your blood glucose is <3.0 mmol/l or you have symptoms requiring medical attention which are thought to be due to low blood glucose,<br>• Your fasting blood glucose is >5.2 mmol/L on more than a quarter of your measurements on two days in a row,<br>• Your 1 hour post-meal blood glucose is >7.7 mmol/L on more than a quarter of your measurements on two days in a row

Ketones	• Your blood ketones are >1.0 mmol/L

Will I need medications?
If your blood sugars are found to be high despite following the recommended diet and lifestyle programmes you may be advised to start medication to help control your blood sugar. You will be advised on changes to your medications by a diabetes specialist nurse/diabetes midwife and also a Consultant Endocrinologist if required. This is usual practice regardless of whether you are taking part in the study.

What care will I receive after the study has stopped?
At the end of the study, you will be provided appropriate ongoing dietary advice from the study dietitian following your final glucose tolerance test to follow the NHS healthy eating and lifestyle plan. You will receive routine postnatal care from your GP, hospital team, and dietitian if required. You will be advised to see your GP for an annual blood test to check your blood sugar levels (this is routine care for women with gestational diabetes). Approximately 5% of women with gestational diabetes have residual diabetes after delivery. This will be identified from your glucose tolerance test/HbA1c; if this is the case you and your GP will be informed. Your GP will take over the management of your diabetes as per routine care outside the study.

Interview sub study
Women in this study may be invited to take part in an interview at the end of the study . You will be asked about your views and experiences on trying to follow your allocated diet programme. This interview can be arranged at a time that suits you, either at Wythenshawe or St Marys Hospital, at your home, or over the telephone. There is no obligation to take part in this interview study.

<u>Frequently asked questions</u>

Do I have to take part?
No, you do not have to take part if you do not wish to and your decision will not affect any standard of care you receive at Wythenshawe or St Marys hospitals (Manchester University NHS Foundation Trust, MFT).

What happens if I change my mind?
It is OK if you agree to take part in the study but later change your mind. You do not need to give a reason and it will not affect the standard of care you receive. The study team may also choose to withdraw you if it is necessary for your health or safety due to unexpected findings during the study. If you decide to withdraw from the study, or the study is stopped for any reason, you will be asked whether or not you are happy for us to keep the data that may have already been collected. If you do withdraw from the study you will continue to be cared for by your usual specialist diabetes and obstetric teams for the duration of your pregnancy. You will still have the option of completing the end of study questionnaire and/or interview to provide feedback; this is very useful for the research team to help us understand potential reasons you may have chosen to withdraw from the study.

Dapre E, *et al. BMJ Open* 2024; 14:e078264. doi: 10.1136/bmjopen-2023-078264

You will also have the option that if you withdraw, researchers may still collect relevant information about your pregnancy and/or gestational diabetes from your medical records within the 18-month study duration. This will be an option on the consent form.

Are there any benefits from taking part?
You will receive frequent personalised advice and support to follow two diet and lifestyle programmes which may help to control your blood sugar levels throughout your pregnancy. The information gained from this study will also help inform the future NHS care of patients with gestational diabetes.

Are there any risks from taking part?
Research has found that diets consisting of two low-calorie days a week are very low risk. Pregnant women will develop slightly higher levels of ketones when following low calorie diets than women who are not pregnant. Ketones are produced naturally by the body when the body uses fat stores for energy (i.e. when we follow a low calorie diet or haven't eaten enough because we are ill).

Some research suggests that very high levels of ketones throughout pregnancy may cause a higher risk of babies being slightly smaller than average. It is very unlikely that you will develop high levels of ketones by following this diet. You will be provided with a ketone meter, and you will be asked to check your ketone levels after an evening meal on your low-calorie day, and the following morning, to make sure that your ketone levels are normal.

On your low-calorie days you may feel slightly more hungry, or you may experience other effects such as increased nausea, light headedness, or tiredness. It is important that you eat regularly throughout the day to reduce the risk of this happening. You will be asked to report any side effects of following the diet to the team at each appointment.

What happens if my baby or I become unwell during the study?
The safety of you and your baby are of utmost importance and remain our priority. In the instance that either of you become unwell your case will be reviewed by our specialist team and your suitability for continuing in the trial will be decided. Although it remains exceptionally rare, were you to experience the unexpected loss of your baby you will be withdrawn from the trial and supported by the dedicated specialist bereavement team at the hospital. Any information which has been collected as part of the trial will be stored securely and once we have finished the study we will keep some of the data so we can check the results. We will write our reports in a way that no-one can work out that you took part in the study.

What will happen to blood samples which are taken?
Some blood samples taken as part of the study will be sent to the laboratory immediately for analysis and any remaining will be stored securely for the duration of the study. Only your 'study ID' will be used – the samples will have none of your personal details on them. At the end of the study any remaining samples will be disposed of in accordance with the Human Tissue Act (2004).

What happens if something goes wrong?
If you have a concern about any aspect of this study, you should ask to speak with the lead researchers who will do their best to answer your questions (**Dr. Basil Issa** or **Dr. Michelle Harvie – via the study office – via michelle.harvie@manchester.ac.uk or telephone 0161 291 4410**). If you remain unhappy and wish to complain formally, you can do so through the NHS complaints procedure. Details can be obtained from the NHS Patient and Liaison Service (PALS) on **Tel:** 0161 276 8686 or contact the team by email pals@mft.nhs.uk.

The hospital is insured to carry out clinical research through the NHS Indemnity scheme. If something did go wrong and you were harmed or suffered deterioration in your health as a result of taking part in this study then you may have grounds for legal action or compensation.

<u>Additional information about the study</u>

Will my lifestyle be affected if I take part?
An essential aspect of this study is a change to your diet and physical activity patterns with support from a specialist team of healthcare professionals.

Payments
We are able to offer free parking at Wythenshawe/St Marys Hospitals for study visits and offer reimbursement for reasonable travel expenses (car, bus or tram) linked to visits for this study. There are no other payments for taking part.

Will my details be kept confidential?
Yes. The study team and any associated regulatory authorities follow strict ethical and legal guidance regarding participant confidentiality. Any information we have about you will be handled in confidence and will only be used for the purposes of this study. All data recorded will be coded and your name will remain anonymous.

During the study we will inform your GP via letter of your participation in the study and your ongoing results, including your weight, blood tests, any abnormal findings and any recommendations for treatment.

If you join the study, some relevant parts of your medical records may be looked at by authorised personnel at Wythenshawe or St Marys hospitals prior to starting the study. These records may also be looked at by an independent auditing body and regulatory authorities to check that the study is being carried out correctly. We will only access parts of your medical records that are relevant to this research and all information accessed will be kept strictly confidential.

How will we use information about you?
We will need to use information from you and from your medical records for this research project.

Dapre E, *et al. BMJ Open* 2024; 14:e078264. doi: 10.1136/bmjopen-2023-078264

This information will include the following:

- Initials
- NHS number
- Name
- Contact details
- Medical History including test results
- Demographic details

People will use this information to do the research or to check your records to make sure that the research is being done properly. People who do not need to know who you are will not be able to see your name or contact details. Your data will have a code number instead. We will keep all information about you safe and secure.

Once we have finished the study, we will keep some of the data so we can check the results. We will write our reports in a way that no-one can work out that you took part in the study. Other researchers from outside the Trust may ask to see this data for the purposes of furthering their research. We will only share this upon written request to the Trust. The external researchers will be asked to sign a Confidentiality Agreement before any data is shared.

What are your choices about how your information is used?

You can stop being part of the study at any time, without giving a reason, but we will keep information about you that we already have. If you choose to stop taking part in the study, we would like to continue collecting information about your health during pregnancy from your hospital records. If you do not want this to happen, tell us and we will stop. We need to manage your records in specific ways for the research to be reliable. This means that we won't be able to let you see or change the data we hold about you.

Where can you find out more about how your information is used?

You can find out more about how we use your information

- at https://research.cmft.nhs.uk/getting-involved/gdpr-and-research
- by asking one of the research team
- by sending an email to **mft.middas.gdm@nhs.net** or
- by ringing us on **07815987910**

How will my details be used to access the Mobile Applications?

None of your personal details (other than email from which an application is downloaded) will be needed to access the nutritics mobile applications. You can opt to have a 'dummy' e-mail and password under a pseudonym (fake name). Only the research team will know the dummy e-mail address you have been assigned to, in order to be able to review your data. The application will not contain your identifiable data. If you choose to use a mobile application to monitor your blood sugar levels the relevant terms of service for the app and the app developers privacy policy will apply. It will be your responsibility to read and understand these prior to download.

Will my insurance be affected if I take part in this study?

Dapre E, *et al. BMJ Open* 2024; 14:e078264. doi: 10.1136/bmjopen-2023-078264

NHS
Manchester University
NHS Foundation Trust

MANCHESTER 1824
The University of Manchester
NIHR | National Institute for Health Research

It is unlikely that your insurance premiums will be affected by participation in this study as the study has the potential to improve your diabetic control and reduce your risk of ill health. However, if you are at all concerned, then we advise that you contact your insurers and seek expert advice before agreeing to participate.

Who has reviewed this study?
Research in the NHS is looked at by an independent group of people called a Research Ethics Committee (REC). The REC is made up of experts, non-experts and members of the general public. Together they review research applications to ensure your safety, rights, wellbeing and dignity are protected at all times. This study has been reviewed and given favourable opinion by REC.

What will happen to the study results?
It is intended that the results of this study will be presented at conferences and published in medical journals so that we can explain to the medical community what our research results have shown. To do this our study information is double-checked by other professionals in research and healthcare. There is a possibility that the study and its results may be publicised for example on radio, television, magazines, books and websites. **You will not be identified in any publicity, reports or publication arising from this study.** If you would like a general summary of the results of the study you can select this on the consent form or please contact the research team.

Who is organising and funding the research?
Researchers from Wythenshawe hospital have designed this study and will be carrying out this research. This study has been funded by the National Institute of Health and Research.

<u>Further information and contact details</u>
For further information about this study, please contact **mft.middas.gdm@nhs.net** or **07815987910.**

Thank you for taking the time to read this information sheet.
We hope it has been of interest to you.

Dapre E, *et al. BMJ Open* 2024; 14:e078264. doi: 10.1136/bmjopen-2023-078264

Participant pathway for the study

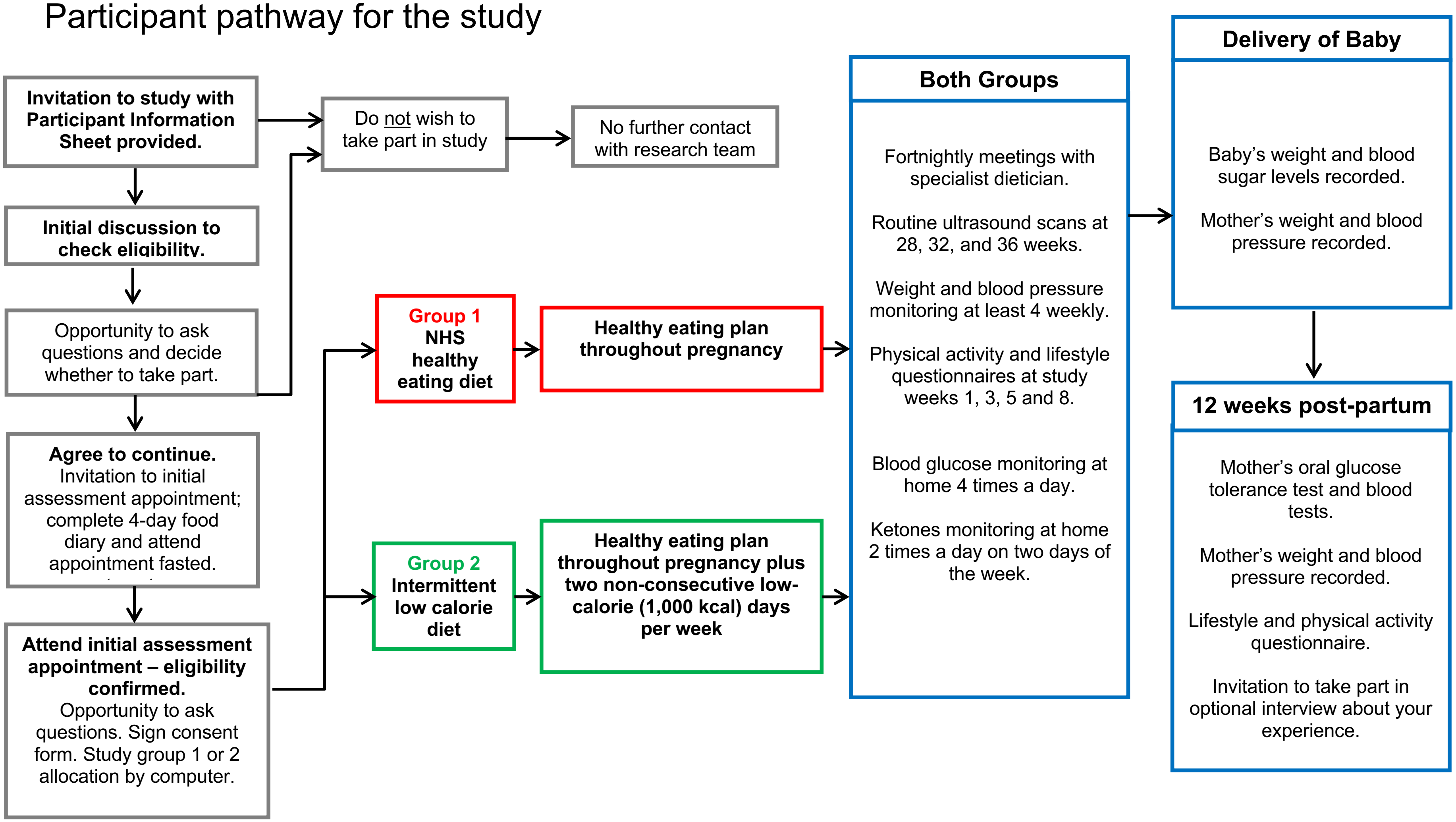

Dapre E, *et al. BMJ Open* 2024; 14:e078264. doi: 10.1136/bmjopen-2023-078264
