# Supplementary material for: Manchester Intermittent Diet in Gestational Diabetes Acceptability Study (MIDDAS-GDM): a two-arm randomised feasibility protocol trial of an intermittent low-energy diet (ILED) in women with gestational diabetes and obesity in Greater Manchester
Source: BMJ Open. 2024 Feb 10;14(2):e078264. doi: 10.1136/bmjopen-2023-078264 (PMC10862275; doi:10.1136/bmjopen-2023-078264)
Supplement: Supplementary data [file bmjopen-2023-078264supp002.pdf]

Consultant Endocrinologist – Dr. Basil Issa
Tel: 0161 291 7070
Research Dietitian – Dr. Michelle Harvie
Tel: 07815987910
Email: mft.middas.gdm@nhs.net

1st Floor Education and Research Centre
Manchester University NHS Foundation Trust
Wythenshawe Hospital
Manchester
M23 9LT

MIDDAS-GDM

Manchester Intermittent Diet in Gestational Diabetes Acceptability Study

Participant Informed Consent Form

Participant Identification Number:

Please **initial** box

1. I confirm that I have read and understand the participant information sheet (version …..) for the above study. I have had the opportunity to consider the information and ask questions, and have had these answered satisfactorily. ☐

2. I understand that my participation is voluntary and that I am free to withdraw at any time, without giving any reason, without my medical care or legal rights being affected. ☐

3. I understand that relevant sections of my medical notes and data collected during the study may be looked at by individuals from Manchester University NHS Foundation Trust and regulatory authorities, where it is relevant to my taking part in the research. I give permission for these individuals to have access to my records. ☐

4. I consent to the collection of blood samples to be collected as described in the participant information sheet. ☐

5. I understand that the information collected about me will be used to support other research in the future and may be shared anonymously with other researchers. ☐

6. I agree that my blood sugar readings can be recorded by the study team. ☐

7. I agree to my GP being informed of my participation in this study and changes to my weight, body measurements, blood results, questionnaire results and medications as required ☐

8. I understand that the information I provide to mobile applications as described in the Patient Information Sheet will be treated in line with the relevant terms of service and the app developers privacy policy at the time of downloading the application. ☐

Dapre E, *et al. BMJ Open* 2024; 14:e078264. doi: 10.1136/bmjopen-2023-078264

9. I have informed the study team of any health issues, including those which may affect my ability to follow the diet, and I will inform the study team of any unusual symptoms that occur during the diet. I will inform the study team of changes to my health status during the study. ☐

10. I have informed the study team of any health issues, including those which may affect my ability to exercise, and I will inform the study team of changes to my health status during the study. ☐

11. I consent to the storage of personal information (including electronic) for the purposes of this study. I understand that any information that could identify me will be kept strictly confidential and that no personal information will be included in the study report or other publication. ☐

12. I agree to take part in the above study. ☐

13. I agree that relevant information about my pregnancy and/or gestational diabetes can be obtained from my medical records within the 18-month study duration if I withdraw from the study early. ☐

14. I am aware that my non-identifiable trial data may be shared with other researchers for the purposes of research. ☐

Optional (delete as appropriate)

15. I agree to be approached to take part in sub-study 1 (interview study), and understand that I will be approached to take part in the sub-study regardless of whether I withdraw from the main study YES/NO

16. I would like to receive a summary of the final study results YES/NO

17. I agree to be contacted regarding future research opportunities YES/NO

My preferred contact (*please tick and include email if preferred*)

Do not contact ☐ Post ☐ Email ☐ ______________________

Signatures

……………………………..………..	…………………..	…………..……………..
Name of participant	Date	Signature
………………………………..……...	………………….	……………..…………..
Name of person taking consent	Date	Signature

When completed: 1 for participant; 1 for patient file; 1 for medical notes;; 1 (original) for site file

Dapre E, *et al. BMJ Open* 2024; 14:e078264. doi: 10.1136/bmjopen-2023-078264
