# Supplementary material for: Manchester Intermittent Diet in Gestational Diabetes Acceptability Study (MIDDAS-GDM): a two-arm randomised feasibility protocol trial of an intermittent low-energy diet (ILED) in women with gestational diabetes and obesity in Greater Manchester
Source: BMJ Open. 2024 Feb 10;14(2):e078264. doi: 10.1136/bmjopen-2023-078264 (PMC10862275; doi:10.1136/bmjopen-2023-078264)
Supplement: Supplementary data [file bmjopen-2023-078264supp003.pdf]

Self-monitoring schedule for capillary glucose and ketone monitoring

ILED		Best NHS Care	
**Ketones (low kcal days)**	**Glucose**	**Ketones (2 days/wk)**	**Glucose**
Fasting before breakfast the morning after each of the low-energy days	Fasting (morning)	Fasting before breakfast on 2 non-consecutive days / week	Fasting (morning)
1 hour post evening meal on each of the low-energy days	1hr post breakfast	1 hour post evening meal on 2 non-consecutive days / week	1hr post breakfast
	1hr post lunch		1hr post lunch
	1hr post dinner		1hr post dinner
