# Supplementary material for: Manchester Intermittent Diet in Gestational Diabetes Acceptability Study (MIDDAS-GDM): a two-arm randomised feasibility protocol trial of an intermittent low-energy diet (ILED) in women with gestational diabetes and obesity in Greater Manchester
Source: BMJ Open. 2024 Feb 10;14(2):e078264. doi: 10.1136/bmjopen-2023-078264 (PMC10862275; doi:10.1136/bmjopen-2023-078264)
Supplement: Supplementary data [file bmjopen-2023-078264supp004.pdf]

Example of 1 day meal plan for Diet Day

The diet days aim to limit the calories to 1000 calories per day. You are aiming to include 2 (not consecutive) diet days each week. The other 5 days, follow the Mediterranean diet as described earlier. To keep the calories to 1000, the diet day will look like this:

Mixed diet		Vegetarian/ vegan diet
**4**	Carbohydrate portions	3
**6**	Protein portions	7
**5**	Vegetable portions	5
**2**	Fruit portions	2
**3**	Dairy portions	3
**1**	Fat portions	1

Below are some examples of meals that can be used to help you follow a 1000 calorie diet.. There are options for a mixed diet or vegan or vegetarian options, if you feel you wanted to try meat free days. Filling up on vegetables will make you feel less hungry

Mixed diet options

Breakfast	Portion	Dairy	Protein	Carb	Veg	Fruit	Fat
Grilled lean bacon	1 rasher	0	1	0	0	0	0
Grilled tomatoes	7 cherry tomatoes	0	0	0	1	0	0
Tea/ coffee	1 mug	0	0	0	0	0	0
Midmorning							
Diet or natural yogurt	1 small carton	1	0	0	0	0	0
Lunch							
Wholegrain bread	2 medium slices	0	0	2	0	0	0
Tuna	⅓ of a 120g can	0	1	0	0	0	0
Green salad	Cereal bowl full / 80 g with oil-free dressing	0	0	0	1	0	0
Satsumas	2	0	0	0	0	1	0
Mid afternoon							
Low fat cheese	30g / match box size	1	0	0	0	0	0
Apple slices	I medium apple (80g)	0	0	0	0	1	0
Tea/ coffee		0	0	0	0	0	0
Evening							
Vegetable rice	4 tablespoons cooked rice 160g of mix vegetables	0	0	2	2	0	0
Chicken curry	90g /average chicken breast (no skin) & ½ can tomatoes, 1 desertspoon oil	0	3	0	1	0	1
Bedtime							
Low fat houmous	1 level tablespoon	0	1	0	0	0	0
Pepper sticks	½ red pepper	0	0	0	1	0	0
Milk	1 small glass	1	0	0	0	0	0
**Total portions a day**		**3 portions**	**6 portions**	**4 portions**	**5 portions**	**2 portions**	**1 portion**

Vegetarian option

Breakfast		Dairy	Protein	Carb	Veg	Fruit	Fat
Egg	2 poached	0	2	0	0	0	0
Mushrooms	2 cupped handfuls / 80g	0	0	0	1	0	0
Tea/ coffee	1 mug	0	0	0	0	0	0
Midmorning							
Cheddar cheese	1 match box size / 30g	1	0	0	0	0	0
Cucumber	Sliced handful	0	0	0	1	0	0
Lunch							
Baked beans	2 tablespoons	0	1	0	0	0	0
Seeded bread toasted	1 medium sliced	0	0	1	0	0	0
Blueberries	1 handful	0	0	0	0	1	0
Mid afternoon							
Meat free ham	2 slice small	0	1	0	0	0	0
Pepper	½ sliced	0	0	0	1	0	0
Avocado	¼	0	0	0	0	0	1
Tea/ coffee	1 mug	0	0	0	0	0	0
Evening							
Vegetarian sausage casserole Jacket potato (100g)	1 grilled sausage 2 cereal bowls vegetables 1 ½ egg sized (100 g)	0	2	1	2	0	0
Bedtime							
Pear	1 medium	0	0	0	0	1	0
Low fat cream cheese	1 tablespoon	1	0	0	0	0	0
Whole wheat cracker	2 biscuits	0	0	1	0	0	0
Milk	1 small glass	1	1	0	0	0	0
**Total portions a day**		**3 portions**	**7 portions**	**3 portions**	**5 portions**	**2 portions**	**1 portions**

Vegan options

Breakfast		Dairy equivalent	Protein	Carb	Veg	Fruit	Fat
Branflakes	3 tablespoons	0	0	1	0	0	0
Milk- soya	200 ml	1	0	0	0	0	0
Tea/ coffee	1 mug	0	0	0	0	0	0
Midmorning							
Soya yogurt	3 tablespoons	1	0	0	0	0	0
Lunch							
Kidney bean & Vegetable chilli<br><br>Wholemeal pitta	3 tablespoons of beans 60g with 1 cereal bowl mixed vegetables & 1/2 can chopped tomatoes<br>½ pitta	0	2	1	2	0	0
Banana	1 medium	0	0	0	0	1	0
Mid afternoon							
Low fat hummus	2 level tablespoon	0	2	0	0	0	0
1 carrot	I medium carrot (80g)	0	0	0	1	0	0
Tea/ coffee	1 mug	0	0	0	0	0	0
Evening							
Quinoa	2 tablespoon cooked	0	0	1	0	0	0
Tofu	4 matchbox	0	2	0	0	0	0
Mixed salad with edamame beans	2 x Cereal bowl full with oil free dressing & 1 tablespoons of edamame	0	1	0	2	0	0
Bedtime							
Peanut butter	1 heaped teaspoon	0	0	0	0	0	1
Apple	1 medium sliced	0	0	0	0	1	0
Milk	1 small glass	1	0	0	0	0	0
**Total portions a day**		**3 portions**	**7 portions**	**2 portions**	**5 portions**	**2 portions**	**1 portions**

To help with estimation of portions the following tables outline weight and measures of the different food groups. Where possible household measures are given to make things a little easier. Use these to help you plan your 2 days in the week of 1000 calories.

**Carbohydrate<br>4 portions - mixed diet<br>3 portions - vegan/vegetarian**	**Equal to**
Wholewheat or oat breakfast cereal, e.g. wholewheat biscuit, malted wholewheat squares, Grapenuts, bran flakes, fruit & fibre	24g or 3 tablespoons or<br>1 whole wheat biscuit
Porridge oats or no-added sugar muesli	20g or 1 heaped tablespoon
Wholegrain, wholemeal, rye, granary bread	36g or medium slice of bread (other than rye), 1½ slices of rye, or ½ roll
Wholemeal or multigrain pitta bread or tortilla wrap, chapatti made without fat	60g or 1x 8" tortilla<br>or 1 standard pitta<br>or small thin chapatti
Rye crispbread, crackers, oak cakes	22g or 2 crispbreads/ 2 oatcakes
Wholegrain rice cake	16g or 2 rice cakes
Wholewheat pasta or rice - cooked amount<br><br>Cous cous, Bulgar wheat, Quinoa, Pearl barley	1 tablespoon uncooked 2 tablespoons cooked<br><br>30g- raw weight or 60g cooked
Lasagne (wholemeal if possible)	20g raw weight or<br>1 large sheet or 1½ smaller sheets
Noodles (wholemeal if possible)	25g raw weight or ½ block/nest
Baked or boiled potato (in skin), cassava,<br>sweet potato	1½ egg sized potatoes or<br>100g raw weight
Wholemeal pizza base (topping is from other food groups)	35g or $^{1}/_{6}$ of thin 10" pizza base
Unsweetened popcorn	20g or 2 handfuls

**Protein**<br>**6 portions – mixed diet**<br>**7 portions – vegan/vegetarian**	**Equal to**
Fresh or smoked white fish (e.g. haddock or cod)	60g or 2oz<br>2 fish finger size
Seafood, e.g. prawns, mussels, crab	45g or 1½oz
Canned tuna or salmon in brine or spring water	45g or 1½oz<br>⅓ standard tin (120g)
Oily fish (fresh or tinned in tomato sauce or olive oil - drained), e.g. mackerel, sardines, salmon, fresh tuna, kippers, smoked salmon or trout	30g or 1oz or<br>¼ standard tin (120g) or<br>¼ fillet of salmon
Chicken, turkey, duck, pheasant (cooked without skin)<br>Lean beef, pork, lamb, rabbit, venison, offal (fat removed)<br>**Quorn fillets, steak, mince or pieces**<br>**Vegetarian mince frozen**	30g or 1oz or<br>1 slice size of playing card
Lean grilled bacon<br>**Quorn ham**	25g or ¾oz or<br>1 rasher
Lean ham<br>**Quorn bacon rashers (not slices)**	30g or 1oz or<br>1 medium, 2 small or 4 wafer thin slices
Eggs	60 g or 2 oz or<br>1 egg
Tofu	50g or 1$^{2}/_{3}$ oz or<br>Size of 2 match boxes
Tempeh	25 g or 1 oz or<br>Size of 1 match box
Baked beans (reduced sugar)	60 g or 2 oz or<br>2 tablespoons
Lentils, chickpeas and kidney beans, mung beans, black eye beans, puy lentils, toor dahl, urad dahl,<br>Raw weight<br><br>Cooked or tinned weight	20g or ⅔ oz or<br>1 tablespoon raw<br><br>65g or 2oz or<br>1½ tablespoons cooked /tinned or 1 cupped handful
Soya beans (frozen or cooked) or edamame beans	30g or 1oz or<br>1 tablespoon
Vegetarian sausage	25g or ¾ oz or ½ sausage
Textured vegetable protein (TVP)	10g or ⅓ oz uncooked or<br>1 heaped tablespoon uncooked
Low fat hummus	30g or 1oz or<br>1 level tablespoon

Vegetables – min 5 portions<br>1 portion = 80g or 2⅔oz	1 portion is equal to
Asparagus, Aubergines, Broccoli, Brussel sprouts, Carrots, Cabbage, Cauliflower, Chinese leaves, Courgettes, Cucumber, Curly kale, Green beans, Lettuce (mixed leaves), Mange tout, Methi, Mushrooms, Okra, Pak choi, Peas, Sugar snap, Spinach, Spring greens cooked, Sweetcorn, Tomatoes, Watercress fresh	80g or 2 ⅔ oz or 2 spears of broccoli, 8 cauliflower florets. 3 heaped tablespoons of vegetables or large cereal bowl of salad.

Fruit - 2 portions.<br>1 portion = 80g or 2⅔oz (30g or 1oz dried fruits)	1 portion is equal to
Berries (e.g. blackberries, blueberries, redcurrants, raspberries, strawberries)<br>Cherries or grapes	80g or 2⅔oz<br>1 handful
Grapefruit, guava and mango	80g or 2⅔oz or<br>½ a whole fruit
Large fruit (e.g. melon, pineapple, papaya)	80g or 2⅔oz or<br>1 medium slice
Medium fruits (e.g. apple, pear, nectarine, orange, peach, banana, pomegranate)	80g or 2⅔oz<br>1 fruit
Small fruit (e.g. fresh apricots, kiwi, clementine, passion fruit, plums)	80g or 2⅔oz or<br>2 fruits
Any stewed fruit—unsweetened or with calorie-free sweetener e.g. apple, rhubarb	80g or 2⅔oz or<br>3 tablespoons
Kumquats, lychees, physalis	5 fruits
Dried fruits (raisins, currants, apricots)	30g or 1oz or<br>1 tablespoon

Milk and dairy foods - 3 portions	Equal to
Milk (semi-skimmed or skimmed)<br>Alternative 'milks' with added calcium, e.g. soya **	⅓ pint or 200ml or<br>1 small glass
Diet yoghurts, Low fat/fat-free Greek or Greek Style or natural yoghurts, fromage frais or plain soya yoghurt, high protein yogurt	120-150g or 4-5oz or<br>1 small pot or<br>3 tablespoons
Whole milk natural yogurt	80g or 1 ⅔ oz or<br>2 tablespoons
Cottage cheese	75g or 1½oz or<br>¼ pot, 2 tablespoons
Cream cheese (light or extra light)	30g or 1oz or<br>1 tablespoon
Lower fat hard cheeses e.g.:<br>Reduced fat cheddar, Edam,<br>Bavarian smoked, feta, ricotta, mozzarella, reduced fat halloumi, paneer made from semi-skimmed milk	30g or 1oz or Matchbox size<br>No more than 180g or 6oz a week

** we recommend soya milk as coconut, oat and almond milks are lower in protein and calcium

**Fat – 2 portions only**	**Equal to**
Margarine or low-fat spread (avoid the buttery types)<br>Olive oil or other oil<br>Oil based dressing<br>Pesto<br>Mayonnaise	8g or 1 teaspoon<br>1 dessertspoon of oil
Seeds (e.g. linseed, pumpkin, sunflower, sesame, chia, hemp)	7g or 1 dessertspoon
Unsalted or salted or dry roasted nuts	7g or 1 dessertspoon<br>3 walnut halves, 3 Brazil,<br>4 almonds, 8 peanuts,<br>10 cashews or pistachios
Olives	50g or 10 olives
Low fat mayonnaise, Curry paste or Harissa paste	15g or ½ oz or 1 tablespoon
Peanut butter (without palm oil)	11g or ⅓ oz or<br>1 heaped teaspoon
Cocoa powder	12 g or ⅓ oz or<br>2 heaped teaspoons
Avocado	40g or 1⅓ oz or<br>1/4 of an average pear
Low fat guacamole	40g or 1⅓ oz or 2 tablespoons
