# Supplementary material for: Manchester Intermittent Diet in Gestational Diabetes Acceptability Study (MIDDAS-GDM): a two-arm randomised feasibility protocol trial of an intermittent low-energy diet (ILED) in women with gestational diabetes and obesity in Greater Manchester
Source: BMJ Open. 2024 Feb 10;14(2):e078264. doi: 10.1136/bmjopen-2023-078264 (PMC10862275; doi:10.1136/bmjopen-2023-078264)
Supplement: Supplementary data [file bmjopen-2023-078264supp005.pdf]

Medical Management Protocols

Hypoglycaemia

Participants will be advised to take 15-20g of rapid acting carbohydrate in the event of hypoglycaemia, (defined as blood glucose <4 mmol/L) which is anticipated to raise blood glucose by 3 mmol/L. Examples of rapid acting carbohydrate include 170-225ml Lucozade Original (not Lucozade Sport), a small carton of fruit juice, 5-6 glucose tablets, 4/5 jelly babies, or a small tin of cola (150-200ml). Participants will be advised to repeat the treatment every 15 minutes until blood glucose is ≥4 mmol/l. The following table highlights when participants should consider taking additional follow-up slower acting carbohydrate:

Situation	Acceptable slow acting carbohydrate
Less than 1 hour before the next meal	Try and avoid
1-2 hour before the next meal	10g
More than 2 hours before the next meal	15-20g

Ketonaemia

Ketone levels ≥1.0 mmol/L on a fasting sample:

- Drink 1L fluids and repeat ketone levels after 4 hours.
- If ketone level has improved (<1.0mmol/L), no further action required.
- If ketone level has increased or remains the same, repeat ketone level after 2 hours.
- If ketone level is persistently increased, consume 40g carbohydrates and repeat in 2 hours.
- Continue to do this until ketone levels <1.0mmol/L.

If a participant experiences >2 episodes of the above throughout the course of the study their notes will be reviewed by the PI and their suitability for remaining in the trial will be assessed.

Guidance for the introduction of diabetes medication (week 24-delivery)

Diabetes medication will be introduced according to the following protocol:

- If ≥25% fasting blood glucose readings are >5 mmol/l and/or ≥25% of 1 hour postprandial glucose readings are >7 mmol/l in a 7 day period: commence Metformin MR 500 mg daily to be increased every 3 days by 500 mg to 1 gram BD if tolerated.

- If after reaching optimal or maximum tolerated dose Metformin ≥25% fasting blood glucose readings are >5 mmol/l in a 7-day period: commence bedtime isophane insulin 4 units and uptitrate the dose by 2 units every 3 days aiming for a fasting glucose of ≤5 mmol/l,

- and/or ≥25% of 1 hour postprandial glucose readings are >7 mmol/l in a 7 day period: commence prandial fast acting insulin analogue (Humalog or Novorapid) 2-4 units with the relevant meal. Uptitrate the dose by 2 units every 3 days aiming for a 1 hour postprandial glucose of ≤7 mmol/l.

- Medication adjustment will be made in accordance with the above guidance.
