# Supplementary material for: Manchester Intermittent Diet in Gestational Diabetes Acceptability Study (MIDDAS-GDM): a two-arm randomised feasibility protocol trial of an intermittent low-energy diet (ILED) in women with gestational diabetes and obesity in Greater Manchester
Source: BMJ Open. 2024 Feb 10;14(2):e078264. doi: 10.1136/bmjopen-2023-078264 (PMC10862275; doi:10.1136/bmjopen-2023-078264)
Supplement: Supplementary data [file bmjopen-2023-078264supp006.pdf]

Manchester University NHS Foundation Trust

MIDDAS-GDM End of Study Questionnaire

Thank you for taking part in the MIDDAS-GDM Study.

This is one of the first studies of its kind. We hope to learn as much as possible from this study, in particular the views of people who have taken part. We are inviting you to provide your views on different aspects of the study and following the diet, and how we can improve our programmes and research studies in future.

Please complete the following questions and return this questionnaire to the MIDDAS-GDM study team in the envelope provided. If there is anything else you would like to say about your experiences of the study, please use the section at the end. Your answers to the questions below will remain anonymous.

1. **What were your reasons for joining the study?**

……………………………………………………………………………………………………...............

……………………………………………………………………………………………………...............

……………………………………………………………………………………………………...............

……………………………………………………………………………………………………...............

……………………………………………………………………………………………………...............

2. **How satisfied were you with study overall (recruitment, appointments etc)?** *(circle)*

1	2	3	4	5	6	7	8	9	10
Not at all satisfied		Slightly satisfied		Quite satisfied		Very satisfied			Extremely satisfied

Comments:………………………………………………………………………………………………

……………………………………………………………………………………………………...............

……………………………………………………………………………………………………...............

Study Appointments

3. **You were asked to attend ………. additional face to face appointments at the hospital by the study team** *(please fill in the number)*

Dapre E, *et al. BMJ Open* 2024; 14:e078264. doi: 10.1136/bmjopen-2023-078264

NHS
Manchester University
NHS Foundation Trust

4. **You were asked to attend** ……….. **additional virtual (i.e. video call or telephone) appointments by the study team** *(please fill in the number)*

5. **How do you feel about the number of additional appointments you were asked to attend?** *(tick)*

☐ I was happy with the number of appointments
☐ I would have preferred fewer face to face appointments
☐ I would have preferred more face to face appointments
☐ I would have preferred fewer virtual appointments
☐ I would have preferred more virtual appointments

Comments:………………………………………………………………………………………………
………………………………………………………………………………………………………...............

6. **How do you feel about virtual (i.e. video call or telephone) appointments?**

☐ I prefer virtual appointments to face to face appointments (please explain why below)
☐ I prefer face to face appointments to virtual appointments (please explain why below)

Comments:………………………………………………………………………………………………
………………………………………………………………………………………………………...............
....………………………………………………………………………………
…………………………..……………………………………

Diet

7. **Which diet were you asked to follow? (*please tick*)**

☐ Best NHS Care (i.e. increased fruit/vegetable intake, low-GI foods, reduction in free sugars, regular meals)
☐ Intermittent low energy diet (5 days of the best NHS care diet plus 2 non-consecutive days of 1000 kcal each week)

8. **Was the diet easy to follow?**

1	2	3	4	5	6	7	8	9	10
Not at all		Slightly		Moderately		Very			Extremely

Manchester University NHS Foundation Trust

Comments:…………………………………………………………………………………………………

…………………………………………………………………………………………………...............

9. Did you enjoy following the diet plan?

1	2	3	4	5	6	7	8	9	10
Not at all		Slightly		Moderately		Very			Extremely

Comments:…………………………………………………………………………………………………

…………………………………………………………………………………………………...............

10. Would you make any changes to the written information (i.e. diet booklets, recipes) you were given on how to follow the diet plan?

☐ Yes

☐ No

If yes, what changes would you make?

Comments:…………………………………………………………………………………………………

…………………………………………………………………………………………………...............

11. How was your first appointment with the dietitian? *(tick all that apply)***?**

☐ The amount of information I received was OK

☐ The amount of information I received was too little

☐ The amount of information I received was too much

☐ I was happy with the advice I received

☐ The advice I received could be improved (specify below)

Comments:…………………………………………………………………………………………………

…………………………………………………………………………………………………...............

Dapre E, *et al. BMJ Open* 2024; 14:e078264. doi: 10.1136/bmjopen-2023-078264

Manchester University
NHS Foundation Trust

12. Would you make any changes to the reviews (calls/face to face appointments) you had with the dietitian during your pregnancy ?

☐ Yes

☐ No

Comments:……………………………………………………………………………………………

……………………………………………………………………………………………………

13. How useful was your final appointment with the dietitian at 12 weeks post-delivery?

1	2	3	4	5	6	7	8	9	10
Not at all		Slightly		Quite		Very			Extremely

Comments:……………………………………………………………………………………………

……………………………………………………………………………………………………

14. Did you feel confident to exercise whilst on the diet plan?

1	2	3	4	5	6	7	8	9	10
Not at all confident		Slightly confident		Quite confident		Very confident			Extremely confident

Comments:……………………………………………………………………………………………

……………………………………………………………………………………………………

Additional support

15. Would any of the following have been useful & if so how often?

	No	Yes	Preferred method of contact	How often
Additional support from dietitian	☐	☐	Face to face / phone	
Additional support from the midwife	☐	☐	Face to face / phone	

Dapre E, *et al. BMJ Open* 2024; 14:e078264. doi: 10.1136/bmjopen-2023-078264

NHS
Manchester University
NHS Foundation Trust

Additional support from the doctors in the clinic	☐	☐	Face to face / phone	
More contact with other women in the study following the diets	☐	☐	Face to face / phone	
Other, please specify:				

16. Did you receive any support outside of the study team help to keep you on track as you progressed through the study?

☐ No

☐ Yes

If yes, what support did you receive?

………

………

Record keeping

17. How did you find the finger prick testing requirements on the study? *(tick all those that apply)*

☐ Challenging but on the whole achievable

☐ Challenging and not achievable

☐ Not challenging at all

☐ I felt it was necessary to test this often to ensure my safety

☐ I felt it was unnecessary to test this often to ensure my safety

Comments:……………………………………………………………………………………………

………

18. How did you find the ketone testing requirements on the study? *(tick all those that apply)*

☐ Challenging but on the whole achievable

☐ Challenging and not achievable

☐ Not challenging at all

☐ I felt it was necessary to test this often to ensure my safety

☐ I felt it was unnecessary to test this often to ensure my safety

Comments:……………………………………………………………………………………………

……………………………………………………………………………………………………

19. How did you find using Diasend software?

☐ Straightforward

☐ Challenging but on the whole achievable

☐ Challenging and not achievable

☐ I felt uncomfortable using computer software to keep track of my medical details

☐ I felt comfortable using computer software to keep track of my medical details

Comments:……………………………………………………………………………………………

……………………………………………………………………………………………………

20. How did you find completing the food diary during the study? *(tick all those that apply)*

☐ Challenging but on the whole achievable

☐ Challenging and not achievable

☐ Not challenging at all

Comments:……………………………………………………………………………………………

……………………………………………………………………………………………………

21. How did you find the physical activity questionnaires on the study? *(tick all those that apply)*

☐ Challenging but on the whole achievable

☐ Challenging and not achievable

☐ Not challenging at all

Comments:……………………………………………………………………………………………

……………………………………………………………………………………………………

Manchester University
NHS Foundation Trust

22. How did you find the quality of life questionnaires on the study? *(tick all those that apply)*

- ☐ Challenging but on the whole achievable
- ☐ Challenging and not achievable
- ☐ Not challenging at all

Comments:……………………………………………………………………………………………

……………………………………………………………………………………………………

Libro® app

23. Did you use the Libro® app?

☐ Yes

☐ No (please move to question 25)

24. Did you find the App helpful?

1	2	3	4	5	6	7	8	9	10
Not at all		Slightly		Moderately		Very			Extremely

Comments……………………………………………………………………………………………

……………………………………………………………………………………………………

25. What did you like about the App?

……………………………………………………………………………………………………

……………………………………………………………………………………………………

……………………………………………………………………………………………………

26. What did you dislike about the App and could be improved?

……………………………………………………………………………………………………

……………………………………………………………………………………………………

27. If you didn't use the app what were the reasons for this? *(tick all that apply)*

☐ Would prefer not to say ☐ Already using other health apps

Dapre E, *et al. BMJ Open* 2024; 14:e078264. doi: 10.1136/bmjopen-2023-078264

- ☐ Don't like using apps in general
- ☐ Labour intensive / time consuming
- ☐ Find mobile devices challenging
- ☐ Lack of regular internet access
- ☐ Other (provide details below)
- ☐ Not user friendly
- ☐ Prefer to use pen and paper

………

………

………

Diasend Software

28. Did you find the Diasend software helpful?

1	2	3	4	5	6	7	8	9	10
Not at all		Slightly		Moderately		Very			Extremely

Comments……………………………………………………………………………………………

………

29. What did you like about the Diasend software?

………

………

30. What did you dislike about the Diasend software and could be improved?

………

………

Study Improvements

31. What did you enjoy most about the study?

………

………

………

………

Manchester University
NHS Foundation Trust

32. What did you enjoy least about the study and could be improved?

……
……
……
……
……

Any other comments about the study

……
……
……
……
……
……
……
……

Thank you for completing this questionnaire, please return to:

MIDDAS-GDM Study Team, Nightingale Centre,

Wythenshawe Hospital, Manchester, M23 9LT

Dapre E, *et al. BMJ Open* 2024; 14:e078264. doi: 10.1136/bmjopen-2023-078264
