# Supplementary material for: Manchester Intermittent Diet in Gestational Diabetes Acceptability Study (MIDDAS-GDM): a two-arm randomised feasibility protocol trial of an intermittent low-energy diet (ILED) in women with gestational diabetes and obesity in Greater Manchester
Source: BMJ Open. 2024 Feb 10;14(2):e078264. doi: 10.1136/bmjopen-2023-078264 (PMC10862275; doi:10.1136/bmjopen-2023-078264)
Supplement: Author's manuscript [file bmjopen-2023-078264.draft_revisions.pdf]

BMJ Open

BMJ Open is committed to open peer review. As part of this commitment we make the peer review history of every article we publish publicly available.

When an article is published we post the peer reviewers’ comments and the authors’ responses online. We also post the versions of the paper that were used during peer review. These are the versions that the peer review comments apply to.

The versions of the paper that follow are the versions that were submitted during the peer review process. They are not the versions of record or the final published versions. They should not be cited or distributed as the published version of this manuscript.

BMJ Open is an open access journal and the full, final, typeset and author-corrected version of record of the manuscript is available on our site with no access controls, subscription charges or pay-per-view fees (http://bmjopen.bmj.com).

If you have any questions on BMJ Open’s open peer review process please email info.bmjopen@bmj.com

BMJ Open

Manchester Intermittent Diet in Gestational Diabetes Acceptability Study (MIDDAS-GDM): A Randomised Feasibility Trial of an Intermittent Low-Energy Diet (ILED) in women with Gestational Diabetes and Obesity

Journal:	*BMJ Open*
Manuscript ID	bmjopen-2023-078264
Article Type:	Protocol
Date Submitted by the Author:	28-Jul-2023
Complete List of Authors:	Dapre, Elizabeth; Wythenshawe Hospital<br>Issa, Basil; Manchester University NHS Foundation Trust, Department of Endocrinology and Diabetes<br>Harvie, Michelle; University Hospital of South Manchester NHS Foundation Trust, Genesis prevention centre<br>Su, Ting-Li; University of Manchester, Dentistry<br>McMillan, Brian; The University of Manchester, Centre for Primary Care and Health Services Research<br>Pilkington, A.; Wythenshawe Hospital<br>Hanna, F.; University Hospitals of North Midlands NHS Trust<br>Vyas, Avni; Manchester Metropolitan University Faculty of Health Psychology and Social Care, Health Professionals<br>Mackie, S.; Wythenshawe Hospital<br>Yates, James; Manchester University NHS Foundation Trust<br>Evans, Benjamin; Manchester University NHS Foundation Trust<br>Mubita, Womba; Manchester University NHS Foundation Trust, Department of Endocrinology and Diabetes<br>Lombardelli, Cheryl; Manchester University NHS Foundation Trust
Keywords:	Diabetes in pregnancy < DIABETES & ENDOCRINOLOGY, DIABETES & ENDOCRINOLOGY, Obesity, NUTRITION & DIETETICS, Feasibility Studies, Maternal medicine < OBSTETRICS

SCHOLARONE™
Manuscripts

Manchester **I**ntermittent **D**iet in Gestational **D**iabetes **A**cceptability **S**tudy (MIDDAS-GDM): A Randomised Feasibility Trial of an Intermittent Low-Energy Diet (ILED) in women with Gestational Diabetes and Obesity

Authors: Dapre, E., Issa, B., Harvie, M., Su, T., McMillan, B., Hanna, F., Pilkington, A., Vyas, A., Yates, J., Mackie, S., Evans, B., Mubita, W., Lombardelli, C.

Dr Elizabeth Dapre (corresponding author)	elizabeth.dapre@hotmail.com<br>c/o Michelle Harvie, The Nightingale Centre, Wythenshawe Hospital, Southmoor Rd, Manchester M23 9LT
Dr Basil Issa	Diabetes and Endocrinology, Wythenshawe Hospital, Manchester, UK
Dr Michelle Harvie	The Nightingale Centre, Wythenshawe Hospital, Manchester, UK
Dr Ting-Li Su	Division of Dentistry, The University of Manchester, UK
Dr Brian McMillan	Division of Population Health, The University of Manchester, UK
Prof Fahmy Hanna	Diabetes and Endocrinology, University Hospitals of North Midlands, UK
Dr Andrea Pilkington	Obstetrics, St Mary's Wythenshawe Hospital, Manchester, UK
Avni Vyas	Division of Dietetics, Manchester Metropolitan University, UK
Benjamin Evans	Division of Clinical Trials, Manchester Foundation Trust, UK
James Yates	Division of Clinical Trials, Manchester Foundation Trust, UK
Sarah Mackie	Division of Clinical Trials, Manchester Foundation Trust, UK
Womba Mubita	Division of Clinical Trials, Manchester Foundation Trust, UK
Cheryl Lombardelli	The Nightingale Centre, Wythenshawe Hospital, Manchester, UK

Word Count: 3572

Submitted to: British Medical Journal Open

Start date of trial: 15th November 2022 (trial ongoing)

Date of submission:

For peer review only

Manchester Intermittent Diet in Gestational Diabetes Acceptability Study (MIDDAS-GDM): A Randomised Feasibility Trial of an Intermittent Low-Energy Diet (ILED) in women with Gestational Diabetes and Obesity

Authors: Dapre, E., Issa, B., Harvie, M., Su, T., McMillan, B., Hanna, F., Pilkington, A., Vyas, A., Yates, J., Mackie, S., Evans, B., Mubita, W., Lombardelli, C.

Corresponding Author: elizabeth.dapre@nhs.net

Abstract (word count 298)

Introduction: The prevalence of gestational diabetes mellitus (GDM) is rising in the UK and is associated with maternal and neonatal complications. National Institute for Health and Care Excellence (NICE) guidance advises first line management with healthy eating and physical activity which is only moderately effective for achieving glycaemic targets. Approximately 30% of women require medication with metformin and/or insulin. There is currently no strong evidence base for any particular dietary regimen to improve outcomes in GDM. Intermittent low-energy diets (ILEDs) are associated with improved glycaemic control and reduced insulin resistance in type 2 diabetes (T2DM) and could be a viable option in the management of GDM. This study aims to test the safety and feasibility of an ILED intervention amongst women with GDM compared to best NHS care.

Method and analysis: We aim to recruit 48 women with GDM diagnosed between 24-28 weeks gestation from antenatal clinics at Wythenshawe and St Mary's hospitals, Manchester Foundation Trust (MFT) over 13 months starting in November 2022. Participants will be randomised (1:1) to ILED (2 low-energy diet days/week of 1000kcal and 5 days/week of the best NHS care healthy diet and physical activity advice) or best NHS care 7 days/week until delivery of their baby. Primary outcomes include uptake and retention of participants to the trial, and adherence to both dietary interventions. Safety outcomes will include birthweight, gestational age at delivery, neonatal hypoglycaemic episodes requiring intervention, neonatal hyperbilirubinaemia, admission to special care baby unit or neonatal intensive care unit, stillbirths, the percentage of women with hypoglycaemic episodes requiring third-party assistance, and significant maternal ketonaemia (defined as ≥1.0mmol/L) Secondary outcomes will assess the fidelity of delivery of the interventions, and

For peer review only

qualitative analysis of participant and healthcare professionals' experiences of the diet. Exploratory outcomes include the number of women requiring metformin and/or insulin.

Ethics and dissemination: Ethical approval has been granted by the Cambridge East Research Ethics Committee (22/EE/0119). Findings will be disseminated via publication in peer-reviewed journals, conference presentations, and shared with diabetes charitable bodies and organisations in the UK, such as Diabetes UK and the Association of British Clinical Diabetologists.

Trial Registration Number: NCT05344066

For peer review only

Strengths and limitations of this study

- This trial will inform the feasibility of recruiting and retaining women with GDM by testing an intermittent low-energy diet, and assessing its acceptability and safety in relation to foetal and maternal complications.
- The outcomes of the trial will inform the design of a future definitive trial.
- Limitations include a limited sample size.
- Women joining this study are likely to be highly motivated and adherence may not reflect that seen in the wider general population.

INTRODUCTION (word count: 3539)

Background

In the UK up to 16% of pregnant women develop gestational diabetes (GDM) and the incidence is rising, in part due to increasing rates of obesity and maternal age(1,2). GDM is associated with maternal and neonatal complications (the risk increases with poor glycaemic control), including macrosomia, shoulder dystocia, caesarean-sections, neonatal hypoglycaemia and/or hyperbilirubinaemia, preterm delivery, preeclampsia, and stillbirth(2). Women who have had GDM have an estimated seven to ten-fold risk of developing type 2 diabetes (T2DM) later in life, and their children have a higher risk of developing adult obesity and T2DM(2–4).

Excessive weight gain in pregnancy is a particular problem for women with GDM(5). Harper *et al* demonstrated that, in women with GDM, every additional 1lb/week gained following diagnosis of GDM resulted in a 36-83% increased risk of pre-eclampsia, caesarean-section, macrosomia, and large for gestational age babies(5). Such studies highlight the importance of adequate weight control throughout pregnancy in women with GDM in order to reduce both maternal and neonatal complications.

First-line therapy for GDM is diet and physical activity. NICE guidance encourages a healthy diet with increased fruit and vegetables, low-glycaemic index (GI) foods, reduced refined sugars, regular mealtimes and regular physical activity(6,7). These dietary measures fail to achieve glycaemic targets in ~30% of women who require medication with metformin and/or insulin(8). A range of dietary approaches have been studied including daily diets promoting low-GI diets (limiting refined and

For peer review only

promoting complex carbohydrates), continuous modest energy-restriction (1800 Kcal/day), and low carbohydrate diets(9). There is currently no strong evidence base for any particular dietary regimen to improve outcomes in GDM.

Intermittent Low-Energy Diets (ILED)

The pathogenesis of GDM is strongly linked to obesity and chronic insulin resistance with many clinicians viewing GDM as a form of evolving T2DM. ILEDs typically include several days of a food based or meal replacement (e.g. drinks/bars) low-energy diet (650-1000kcal) diet, with normal eating on the remaining days of the week. These diets are associated with significant reductions in weight, insulin resistance and hyperglycaemia in patients with prediabetes (HbA1c between 42-47mmol/mol, impaired glucose tolerance, or impaired fasting glycaemia), those with T2DM, and otherwise healthy subjects with overweight/obesity(10–17). These changes are equivalent to, or greater than, those achieved with standard daily energy restriction. A popular intermittent diet involves 2 consecutive or non-consecutive days/week of a low-energy diet (650-1000kcal) and 5 days of normal eating/week, known as the 5:2 diet. The Manchester Intermittent vs. Daily Diabetes App Study (MIDDAS), a study comparing an ILED and a continuous low-energy diet in T2D conducted in our unit, has shown the feasibility and safety of an ILED (800kcal for 2 days/week) in patients with T2DM and obesity, including those using insulin(18). At the end of the study approximately 70% of participants in the ILED group completed the study and achieved a 6% reduction of their baseline body weight. Forty two percent achieved an HbA1c of <48 mmol/mol(18). Given the strong overlap between GDM and T2DM, an ILED may be a promising dietary intervention for those with GDM.

A successful dietary approach to glycaemic control could empower women to take charge of the management of their GDM. Women with GDM are motivated to modify their diet driven by a desire to improve foetal outcomes(19–21).

Our Patient and Public Involvement and Engagement (PPIE) work indicates that women find the current National Institute for Health and Care Excellence (NICE) healthy eating guidance(6,7) confusing and vague. Women are keen to try alternative dietary approaches, particularly if alternative diets are more effective in preventing the need to progress to medications such as metformin and insulin.

For peer review only

Aim

The aim of this trial is to test the feasibility of being able to recruit and retain participants for a future randomised controlled trial (RCT) of an ILED in GDM, assess and ensure the safety of the intervention, and assess the ability of participants to adhere to the diet. An exploratory outcome will be the difference in progression to, and the magnitude of, antidiabetic medication in women with GDM following the ILED and best NHS care. This will inform the primary outcomes for a future large-scale RCT.

METHODS

Trial Design

The study is a 28-week feasibility two-arm RCT in one NHS trust performed in patients with GDM and BMI ≥27.5 kg/m^2, or ≥25 kg/m^2 in high-risk minority ethnic groups (i.e. South Asian, Black African, African Caribbean). There will be an embedded qualitative sub-study for participants and healthcare professionals. Due to the nature of the intervention, it will not be possible to blind the participants, clinicians, or study team to the treatment allocation after randomisation (the statistician and laboratory technicians will remain blinded).

Trial Setting and Recruitment

Participants will be recruited from antenatal clinics at Wythenshawe and St Mary's Hospitals, MFT. Assessments will be carried out at MFT, or remotely if required by COVID-19 restrictions. The qualitative sub-study will be carried out at MFT, remotely, or at a location of the participant's choosing. We aim to recruit eligible participants over a period of 13 months. Potential participants will be given written information about the study and the opportunity to ask questions about the study prior to providing written consent (figure 1).

For peer review only

Eligibility Criteria

<IMAGE ONE; figure1 inclusion exclusion jpg>

Participant Flow

Participants who fulfil the broad eligibility criteria will be notified about the trial by the GDM nurse/midwife at the time of their diagnosis. Those who are interested will be provided with a comprehensive patient information sheet (see appendix) and more detailed eligibility screening questions. They will be asked to attend their first appointment having fasted for at least 6 hours and complete a four-day food diary (in line with our departments usual care). On attending their first routine clinic appointment, interested participants will receive further information from the research team. They will have the opportunity to ask questions, have their eligibility confirmed, and will be asked for their written consent to take part. Baseline assessments will be taken and participants will be randomised to their allocated treatment group using an online randomisation platform. Participant flow through the study is demonstrated in figure 2.

Sample Size

We plan to recruit 24 participants per study arm (n=48) which, when considering an estimated attrition rate of 15%, will provide complete outcome data on 40 participants(22–24). It has been estimated that 24 participants per group will be sufficient to determine study outcomes, in line with sample size recommendations for feasibility studies(25–27). This number will allow us to evaluate the other objectives of the trial, to assess the impact of the intervention on each of the outcome measures, to estimate parameters necessary to design a main trial, and will enable estimation of recruitment/retention parameters with sufficient precision.

Randomisation

The randomisation schedule will be independently set up and known only by the trial statistician. The trial statistician will be blinded to the participant's identity using "sealed envelope" software (https://www.sealedenvelope.com/). Randomisation will be carried out by generating an online pseudo-random list with random permuted

For peer review only

blocks of varying size, known only to the statistician, and will be stratified for two variables:

- Age (18-35, >35 years)
- BMI (27.5-34.99kg/m^2 and >35kg/m2, >25-32.49kg/m^2 and >32.5kg/m^2 for high-risk minority ethnic groups (i.e. South Asian, Black African, African Caribbean)

Treatment to intervention and control groups will be allocated in a 1:1 ratio. A member of the research team (who will be unaware of the randomisation algorithm) will trigger the randomisation procedure onsite; participants and clinicians will then be informed of the allocated treatment group. Clinicians will not be blinded due to the need to remain astute to safety, adherence, and side effects, requiring open and honest discussions with patients at each appointment. The statistician will remain blinded to treatment allocation until all outcome measures for all subjects have been collected.

Interventions

Study Arm 1: Best NHS Care Diet

All dietetic advice will be face to face or via video calls or the telephone. Participants will receive one to one personalised written and verbal advice from a dietitian to follow NICE diet and physical activity recommendations(6,7). Dietitians and midwives will receive training to ensure standardised delivery of information in clinic, and standardised patient information leaflets will be supplied to include information about increased fruit/vegetable intake, low-glycaemic index foods, and a reduction in free sugars. Information will include advice about the importance of regular meals; dietary advice aims to ensure that participants include at least 70g protein/28g fibre, and predominantly mono- and polyunsaturated fats as per American Diabetes Association recommendations(28). Participants will be advised to be physically active, for example walking for 30 minutes after a meal. Participants will receive ongoing dietetic education and support every 2 weeks until delivery. They will receive suggested menus and recipes to follow the NICE recommended healthy diet for GDM. Participants will be asked to measure their capillary glucose four times each day and their ketones on two random (recorded) days of the week of their choosing (see appendix).

For peer review only

Study Arm 2: Intermittent Low-Energy Diet (ILED)

Participants will receive advice on adopting an ILED which involves 2 non-consecutive low-energy diet days/week (1000kcal to include 100g low-GI carbohydrate and 70g of protein) and 5 days/week of the NICE healthy eating low-GI diet and physical activity recommended for the best NHS care group. The low-energy days involve women selecting a set number of portions of protein, carbohydrate, fat, fruit, vegetables, and dairy/dairy alternatives as described in previous studies(29). Each low-energy day includes ~210g of lean protein foods, 3-4 portions of wholegrain carbohydrates, 1x7g portion of fat, 5 portions of vegetables, 2 of fruit, and 3 of dairy/dairy alternatives. Food and drink will be self-selected and not provided by the study team. Participants will be provided with comprehensive food lists, advice on portion sizes for the low-energy days and suggested menus and recipes to follow for both the low-energy and NICE recommended healthy diet days. Both diets can be successfully adapted for people of different ethnicities and those following omnivorous, vegetarian and vegan diets. Participants will be asked to measure their capillary glucose four times each day and their ketones on (and the morning after) the two low-energy days (see appendix).

<IMAGE 2: figure 2 flow jpg>

<IMAGE 3: figure 3 sched assessments jpg>

For peer review only

Outcomes

Primary outcomes

- Uptake rate measured as a percentage of eligible participants who consent to take part, including the proportion of women who were screened who did not meet the eligibility criteria, and the number of women who did not give consent to take part
- Recruitment rate measured as the number of eligible participants who consent to take part per month
- Retention rate measured as the number of randomised participants who complete the trial (those who attend the final visit) and the percentage of participants who attend all 8 visits
- Adherence to the dietary interventions assessed from self-reported adherence to the potential low calorie days between randomisation and delivery
- Completion of self-assessed glucose and ketone readings assessed as a percentage of the required readings

- Safety outcomes:
  - Percentage of women following ILED/best NHS care with hypoglycaemia (episodes of blood glucose of <3.0mmol/mol) and hypoglycaemia requiring third-party assistance
  - Percentage of women who develop significant ketonaemia in both groups (defined as ≥1.0mmol/L)
  - Percentage of neonatal hypoglycaemic episodes requiring intervention (blood glucose checked 2- hours post-delivery and 2-hours thereafter for 12 hours according to local protocol), neonatal birth weight, gestational age at delivery, hyperbilirubinaemia/jaundice, and/or admission to Special Care Baby Unit or neonatal intensive care, and stillbirths
  - The incidence and rate of other adverse effects (e.g. headaches, lethargy, and constipation between the start of the trial intervention and delivery – mild, moderate and severe, as defined by Common Terminology Criteria for Adverse Events version 5 (CTCAEv5)(30). Hospital admission for routine labour and delivery will not be classified as an adverse event.

For peer review only

Secondary outcomes

- Completeness of collection of trial endpoints including the percentage of completed weight measurements, 4-day food diaries, and International Physical Activity Questionnaire (IPAQ) scores
- Fidelity of delivery of the interventions will be measured through the number and modality of completed planned patient contacts with the dietitian
- Qualitative analysis of the acceptability and implementation of the interventions will be explored amongst a subset of participants (~10 in each group) and healthcare professionals through in-depth interviews
- Dietary changes in both groups will be assessed using the UK Diabetes and Diet Questionnaire (UKDDQ), analysis of completed food diaries, and any other dietary modifications self-reported at the two weekly dietitian reviews

Exploratory outcomes

1. Maternal outcomes:
    - The percentage of women requiring metformin and/or insulin
    - Four-point capillary glucose profiles during third trimester (four times daily until delivery)
    - Change in fasting blood test results between baseline measurements, 36-37 weeks' gestation, and 12 weeks post-delivery (including oral glucose tolerance tests (OGTT)
    - Mode of delivery, development of preeclampsia, polyhydramnios (maximum liquor volume pool depth ≥8 cm)
    - Quality of life and health status questionnaires (WHOQoL-BREF and SF-36 questionnaires)(31,32)
2. Foetal outcomes:
    - Foetal weight
    - Gestational age at delivery
    - Cord-blood glucose, insulin and C-peptide, where collection is possible.

For peer review only

Measurements

The full schedule of assessments can be found in figure 3.

Physical measurements

Height, weight and blood pressure will be measured using standardised calibrated equipment in antenatal clinic.

Blood samples

Fasting venous blood samples will be collected to assess maternal HbA1c, fasting glucose, insulin, beta-hydroxybutyrate, liver function tests, free fatty acids, thyroid function tests, and full blood count. A cord blood sample will be collected at the time of delivery to measure neonatal glucose, and insulin and C-peptide where collection is possible. At the end of the study all samples will be disposed of in accordance with the Human Tissue Act (2004).

Questionnaires

Participants will be asked to complete four questionnaires at four time points throughout the trial. Quality of life and health status will be assessed using the World Health Organisation Quality of Life Questionnaire (brief version) and the 36-Item Short Form Survey respectively(31,32). Physical activity will be measured using the International Physical Activity Questionnaire – Short Form, and diet quality will be assessed using the UK Diabetes and Diet Questionnaire(33,34).

Food Diaries

4-day dietary records will be completed using Libro (Nutritics Mobile Application) or paper food diaries, which will be entered into Nutritics software (Nutritics, Dublin, Ireland)(35). Diaries will provide the research team with information about the intake of energy, carbohydrate, fat, protein, fibre, glycaemic index, and the timing of meals for participants in both groups. Participants will be asked what other dietary modifications, if any, they have made at their fortnightly dietitian reviews to establish the adoption of any alternative dietary practices in the cohort.

For peer review only

Adverse Events

Participants in both groups will be asked about any adverse effects that they have experienced at each visit. These will include, but are not limited to, the potential effects of a low-energy diet, e.g. headache, lethargy, dizziness, constipation, indigestion, poor concentration, and hunger. Adverse events will be graded as per CTAEv5(30). Participants will be issued with a participation/emergency card with emergency contact details for the research team to be carried at all times and to be shown to the attending physician in case of emergency admission to hospital. All participants will be issued with clear instructions as to how to manage a hypoglycaemic and/or ketonaemic event (see appendix).

Data management

Participant data will be anonymised and will be stored in line with the Medicines for Human Use (Clinical Trials) Amended Regulations 2006 and the Data Protection Act (2018) and archived in line with the Medicines for Human Use (Clinical Trials) Amended Regulations (2006) as defined in the MFT Clinical Trials Office Archiving SOP (11; Retention of Data, Off-Site Archiving, and Destroying Documents). Deidentified data will be stored in a study-specific Research Electronic Data Capture (REDCap) database. The sponsor will periodically audit the site study file, a sample of the case report form, consent forms, and source data, and check accuracy of the study database to ensure satisfactory completion.

Statistical methods

A statistical analysis plan specifying the full details of the primary and secondary outcomes, other variables, and methods, will be produced prior to trial analysis. The main analysis will be conducted via intention-to-treat population and will not undertake any significance tests. Descriptive, graphical (summary), and basic statistics (e.g. i. number, frequencies and percentages, ii. mean and standard deviation, or iii. median and quartiles as appropriate) will be presented as appropriate for each group respectively, for group difference jointly, and for each stratum. Per-protocol analysis will be considered as a secondary analysis. Levels of missing data will be investigated and used to inform future studies. No imputation will be used. The end of study questionnaire will be analysed using appropriate

For peer review only

descriptive statistics for closed questions and key themes will be extracted without formal analysis from open questions to inform future research.

Progression Criterion

The success of the feasibility trial will be defined by the progression criteria as outlined in table 1.

	Feasible (green)	Feasible with modification of the protocol (amber)	Not feasible (red)
**Recruitment**	≥4 patients/month	>2 patients/month	≤2 patients/month
**Uptake to the feasibility study**	≥15%	10-15%	<10%
**Retention to the feasibility study**	>70%	50-70%	<50%
**Adherence to the ILED intervention**	>50% of the low-energy days completed (2/week between weeks 24-28 and delivery)	30-50% of the low-energy days completed (2/week between weeks 24-28 and delivery)	<30% of the low-energy days completed (2/week between weeks 24-28 and delivery)

Table 1: Trial progression criterion

Qualitative sub-study

Participants will be invited to take part in an optional qualitative sub-study at 11-13 weeks post-partum. Healthcare professionals delivering the interventions will also be invited to take part in this study.

We will undertake 110-120 semi-structured interviews with a subset of women from each group (ILED n=10 and best NHS Care n=10) at around 12 weeks post-delivery. The final sample size will be contingent on obtaining data saturation. We will also interview a sample of healthcare professionals (HCPs) involved in the delivery of care to study participants, including dieticians, obstetricians and midwives. Sampling will be purposive, aiming to obtain women from a range of ethnic groups, ages, socioeconomic backgrounds, and self-reported engagement with the intervention. Participants and HCPs will be asked about their experiences and thoughts regarding

For peer review only

the intervention, including motivating factors, and facilitators/barriers to engagement. Interviews will be conducted by a researcher from the University of Manchester/MFT who is independent from the research staff involved in the delivery and assessment of the programmes. Analysis will be conducted by two independent researchers at the University of Manchester/MFT using Braun and Clarke's thematic analysis approach to identify key issues around the acceptability, usefulness of the programmes, and feasibility of a subsequent trial(36). Analysis will be inductive: open-ended, exploratory, and driven by the data.

All participants will also be asked to complete an optional and anonymous end of study questionnaire developed by the study team at their post-partum visit (see appendix). This will give participants the opportunity to feedback on their experience and will enable the study team to identify improvements to the design of a possible follow-up study.

Trial Steering Committee (TSC)

The trial steering committee will include an independent consultant endocrinologist, obstetrician, dietitian, and the patient representative. The committee will oversee the trial to ensure that it is carried out to the expected standards. The TSC will liaise with the CI to develop a schedule of meetings, proposed to occur every four months, with meetings to occur no less than annually. Minutes will be taken at TSC meetings and copies of the minutes will be filed in the Trial Master File; they will be shared with relevant stakeholders as appropriate.

Patient and public involvement

Patient and public involvement was actively sought throughout the planning and design of this trial and continues to form a key part of the trial as it progresses. The patient and public involvement and engagement (PPIE) group assisted in the development of all participant materials and provided valuable insight into the wording of participant information and acceptability of the proposed intervention. The PPIE group will be updated as the trial progresses and a further focus group will be held to advise on the interview schedule and wording for the qualitative sub-study. The group will also be invited to aid in the development of summarising key findings for dissemination to relevant patient groups.

For peer review only

Ethics and dissemination

This study has been approved by the Cambridge East Research Ethics Committee and is sponsored by MFT. Findings will be disseminated via publication in peer-reviewed journals, conference presentations, and shared with diabetes charitable bodies and organisations in the UK, such as Diabetes UK and the Association of British Clinical Diabetologists. Anonymised data will be available upon formal request once the principal results of the study have been published. Planned modifications to the protocol will be approved by the research ethics committee before they are adopted into the study. An audit trail of ethical amendments and documentation will allow monitoring by the research team and external regulatory bodies.

This is the first study to assess the feasibility and safety of an ILED in GDM as compared to best NHS care. Given the increasing incidence of GDM and associated health risks this research is both pertinent and important. The study is not powered to show differences between ILED and best NHS care, however the planned quantitative and qualitative assessments will inform the feasibility of the programme and a future definitive trial.

For peer review only

Full References

1. The British Diabetic Association. What is gestational diabetes? [Internet]. Diabetes UK. [cited 2019 Nov 27]. Available from: https://www.diabetes.org.uk/diabetes-the-basics/gestational-diabetes

2. Kim C. Gestational diabetes: risks, management, and treatment options. Int J Womens Health. 2010 Oct 7;2:339–51.

3. Kim SY, Sharma AJ, Callaghan WM. Gestational diabetes and childhood obesity: what is the link? Curr Opin Obstet Gynecol. 2012 Dec;24(6):376–81.

4. Vounzoulaki E, Khunti K, Abner SC, Tan BK, Davies MJ, Gillies CL. Progression to type 2 diabetes in women with a known history of gestational diabetes: systematic review and meta-analysis. BMJ. 2020 May 13;m1361.

5. Harper L, Tita A, Biggio J. The Institute of Medicine Guidelines for Gestational Weight Gain after a Diagnosis of Gestational Diabetes and Pregnancy Outcomes. Am J Perinatol. 2014 Jun 27;32(03):239–46.

6. Webber J, Charlton M, Johns N. Diabetes in pregnancy: management of diabetes and its complications from preconception to the postnatal period (NG3). Br J Diabetes. 2015 Sep 1;15(3):107.

7. NHS. NHS Treatment Gestational Diabetes [Internet]. 2019 [cited 2020 Jan 10]. Available from: https://www.nhs.uk/conditions/gestational-diabetes/treatment/

8. Mayo K, Melamed N, Vandenberghe H, Berger H. The impact of adoption of the international association of diabetes in pregnancy study group criteria for the screening and diagnosis of gestational diabetes. Am J Obstet Gynecol. 2015 Feb;212(2):224.e1-9.

9. Han S, Middleton P, Shepherd E, Van Ryswyk E, Crowther CA. Different types of dietary advice for women with gestational diabetes mellitus. Cochrane Database Syst Rev. 2017 25;2:CD009275.

10. Carter S, Clifton PM, Keogh JB. Effect of Intermittent Compared With Continuous Energy Restricted Diet on Glycemic Control in Patients With Type 2 Diabetes: A Randomized Noninferiority Trial. JAMA Netw Open. 2018 Jul 20;1(3):e180756.

11. Antoni R, Johnston KL, Collins AL, Robertson MD. Effects of intermittent fasting on glucose and lipid metabolism. Proc Nutr Soc. 2017 Aug;76(3):361–8.

12. Cioffi I, Evangelista A, Ponzo V, Ciccone G, Soldati L, Santarpia L, et al. Intermittent versus continuous energy restriction on weight loss and cardiometabolic outcomes: a systematic review and meta-analysis of randomized controlled trials. J Transl Med. 2018 Dec;16(1):371.

13. Harris L, Hamilton S, Azevedo LB, Olajide J, De Brún C, Waller G, et al. Intermittent fasting interventions for treatment of overweight and obesity in adults: a systematic

For peer review only

review and meta-analysis. JBI Database Syst Rev Implement Rep. 2018 Feb;16(2):507–47.

14. Harvie M, Howell A. Potential Benefits and Harms of Intermittent Energy Restriction and Intermittent Fasting Amongst Obese, Overweight and Normal Weight Subjects—A Narrative Review of Human and Animal Evidence. Behav Sci. 2017 Jan 19;7(4):4.

15. Harvie MN, Pegington M, Mattson MP, Frystyk J, Dillon B, Evans G, et al. The effects of intermittent or continuous energy restriction on weight loss and metabolic disease risk markers: a randomized trial in young overweight women. Int J Obes. 2011 May;35(5):714–27.

16. Wang X, Li Q, Liu Y, Jiang H, Chen W. Intermittent fasting versus continuous energy-restricted diet for patients with type 2 diabetes mellitus and metabolic syndrome for glycemic control: A systematic review and meta-analysis of randomized controlled trials. Diabetes Res Clin Pract. 2021 Sep;179:109003.

17. He S, Wang J, Zhang J, Xu J. Intermittent Versus Continuous Energy Restriction for Weight Loss and Metabolic Improvement: A Meta-Analysis and Systematic Review. Obesity. 2021 Jan;29(1):108–15.

18. McDiarmid S, Harvie M, Johnson R, Vyas A, Aglan A, Moran J, et al. Manchester Intermittent versus Daily Diet App Study (MIDDAS): A pilot randomized controlled trial in patients with type 2 diabetes. Diabetes Obes Metab. 2022 Mar;24(3):432–41.

19. Carolan M. Women's experiences of gestational diabetes self-management: A qualitative study. Midwifery. 2013 Jun;29(6):637–45.

20. Gao F, Luo H, Jones K, Nicholson W, Bell RA. Gestational Diabetes and Health Behaviors Among Women: National Health and Nutrition Examination Survey, 2007–2014. Prev Chronic Dis. 2018 Oct 25;15:180094.

21. McMillan B, Easton K, Goyder E, Delaney B, Madhuvrata P, Abdelgalil R, et al. Reducing risk of type 2 diabetes after gestational diabetes: a qualitative study to explore the potential of technology in primary care. Br J Gen Pract. 2018 Apr;68(669):e260–7.

22. H. Al Wattar B, Dodds J, Placzek A, Beresford L, Spyreli E, Moore A, et al. Mediterranean-style diet in pregnant women with metabolic risk factors (ESTEEM): A pragmatic multicentre randomised trial. Persson LÅ, editor. PLOS Med. 2019 Jul 23;16(7):e1002857.

23. Moreno-Castilla C, Hernandez M, Bergua M, Alvarez MC, Arce MA, Rodriguez K, et al. Low-carbohydrate diet for the treatment of gestational diabetes mellitus: a randomized controlled trial. Diabetes Care. 2013 Aug;36(8):2233–8.

24. Rae A, Bond D, Evans S, North F, Roberman B, Walters B. A randomised controlled trial of dietary energy restriction in the management of obese women with gestational diabetes. Aust N Z J Obstet Gynaecol. 2000 Nov;40(4):416–22.

25. Julious SA. Sample size of 12 per group rule of thumb for a pilot study. Pharm Stat. 2005 Oct;4(4):287–91.

For peer review only

26. Sim J, Lewis M. The size of a pilot study for a clinical trial should be calculated in relation to considerations of precision and efficiency. J Clin Epidemiol. 2012 Mar;65(3):301–8.

27. Lancaster GA. Pilot and feasibility studies come of age! Pilot Feasibility Stud. 2015 Dec;1(1):1, 2055-5784-1–1.

28. American Diabetes Association. 13. Management of Diabetes in Pregnancy: *Standards of Medical Care in Diabetes—2018*. Diabetes Care. 2018 Jan;41(Supplement 1):S137–43.

29. Harvie M, Wright C, Pegington M, McMullan D, Mitchell E, Martin B, et al. The effect of intermittent energy and carbohydrate restriction *v* . daily energy restriction on weight loss and metabolic disease risk markers in overweight women. Br J Nutr. 2013 Oct 28;110(8):1534–47.

30. National Cancer Institute. Common Terminology Criteria for Adverse Events (CTCAE) [Internet]. Cancer Therapy Evaluation Program. 2017 [cited 2020 May 7]. Available from: https://ctep.cancer.gov/protocolDevelopment/electronic_applications/ctc.htm#ctc_50

31. World Health Organisation. WHOQOL: Measuring Quality of Life [Internet]. Available from: https://www.who.int/tools/whoqol

32. Nair R, Kachan P. Outcome tools for diabetes-specific quality of life: Study performed in a private family practice clinic. Can Fam Physician Med Fam Can. 2017 Jun;63(6):e310–5.

33. Ekelund U, Sepp H, Brage S, Becker W, Jakes R, Hennings M, et al. Criterion-related validity of the last 7-day, short form of the International Physical Activity Questionnaire in Swedish adults. Public Health Nutr. 2006 Apr;9(2):258–65.

34. England CY, Thompson JL, Jago R, Cooper AR, Andrews RC. Development of a brief, reliable and valid diet assessment tool for impaired glucose tolerance and diabetes: the UK Diabetes and Diet Questionnaire. Public Health Nutr. 2017 Feb;20(2):191–9.

35. Nutritics [Internet]. [cited 2020 Jul 12]. Available from: https://www.nutritics.com/p/home?utm_source=adwords&utm_campaign=Nutritics+Brand&utm_term=&utm_medium=ppc&hsa_grp=78385303355&hsa_mt=b&hsa_kw=&hsa_src=g&hsa_ad=380634402696&hsa_cam=1619635404&hsa_net=adwords&hsa_acc=2361676125&hsa_ver=3&hsa_tgt=dsa-814211721343&gclid=Cj0KCQjw6ar4BRDnARIsAITGzlAm_uy2YNRBU5N-egXXyehUQ_9CHKylLQI_LgCYJK9Jpet4oIvUM5saAkzREALw_wcB

36. Braun V, Clarke V. Successful qualitative research: a practical guide for beginners. Los Angeles: SAGE; 2013. 382 p.

For peer review only

Authors' contributions

Michelle Harvie, Basil G. Issa, Elizabeth Dapre, Brian McMillan, Ting-Li Su, Fahmy Hanna, Andrea Pilkington and Avni Vyas designed the study, wrote the protocol, and secured the funding. Elizabeth Dapre drafted the manuscript for publication, with input from Michelle Harvie, Basil G. Issa, Brian McMillan, and Ting-Li Su. All other authors have proofed and checked the manuscript.

Acknowledgments

With thanks to Mrs Rebecca Lumsden, our patient expert, whose insight has been invaluable in the design of the study. We are most grateful to Mrs Jodie Aspinall, and Miss Lisa Brew-Butler, specialist midwives who continue to help identify suitable candidates for the study.

Funding statement:

This trial is funded by the National Institute for Health Research (NIHR201944) and sponsored by Manchester University NHS Foundation Trust (MFT). The funders of the study had no role in the study design or writing of the report. Dr Dapre is an NIHR sponsored GP academic clinical fellow.

Competing interests statement.

Michelle Harvie has co-authored three self-help books for the public to follow intermittent diets. All author proceeds are paid directly to the charity Prevent Breast Cancer (registered charity number 1109839) to fund breast cancer research.

For peer review only

Appendix

1.0 Patient Information Sheet (supplementary document 1)

2.0 Consent Form (supplementary document 2)

3.0 Self-monitoring schedule for capillary glucose and ketone monitoring

ILED		Best NHS Care	
**Ketones (low kcal days)**	**Glucose**	**Ketones (2 days/wk)**	**Glucose**
Fasting before breakfast the morning after each of the low-energy days	Fasting (morning)	Fasting before breakfast on 2 non-consecutive days / week	Fasting (morning)
1 hour post evening meal on each of the low-energy days	1hr post breakfast	1 hour post evening meal on 2 non-consecutive days / week	1hr post breakfast
	1hr post lunch		1hr post lunch
	1hr post dinner		1hr post dinner

4.0 Medical Management Protocols

Hypoglycaemia

Participants will be advised to take 15-20g of rapid acting carbohydrate in the event of hypoglycaemia, (defined as blood glucose <4 mmol/L) which is anticipated to raise blood glucose by 3 mmol/L. Examples of rapid acting carbohydrate include 170-225ml Lucozade Original (not Lucozade Sport), a small carton of fruit juice, 5-6 glucose tablets, 4/5 jelly babies, or a small tin of cola (150-200ml). Participants will be advised to repeat the treatment every 15 minutes until blood glucose is ≥4 mmol/l. The following table highlights when participants should consider taking additional follow-up slower acting carbohydrate:

Situation	Acceptable slow acting carbohydrate
Less than 1 hour before the next meal	Try and avoid
1-2 hour before the next meal	10g
More than 2 hours before the next meal	15-20g

For peer review only

Ketonaemia

Ketone levels ≥1.0 mmol/L on a fasting sample:

- Drink 1L fluids and repeat ketone levels after 4 hours.
- If ketone level has improved (<1.0mmol/L), no further action required.
- If ketone level has increased or remains the same, repeat ketone level after 2 hours.
- If ketone level is persistently increased, consume 40g carbohydrates and repeat in 2 hours.
- Continue to do this until ketone levels <1.0mmol/L.

If a participant experiences >2 episodes of the above throughout the course of the study their notes will be reviewed by the PI and their suitability for remaining in the trial will be assessed.

Guidance for the introduction of diabetes medication (week 24-delivery)

Diabetes medication will be introduced according to the following protocol:

- If ≥25% fasting blood glucose readings are >5 mmol/l and/or ≥25% of 1 hour postprandial glucose readings are >7 mmol/l in a 7 day period: commence Metformin MR 500 mg daily to be increased every 3 days by 500 mg to 1 gram BD if tolerated.

- If after reaching optimal or maximum tolerated dose Metformin ≥25% fasting blood glucose readings are >5 mmol/l in a 7-day period: commence bedtime isophane insulin 4 units and uptitrate the dose by 2 units every 3 days aiming for a fasting glucose of ≤5 mmol/l,

- and/or ≥25% of 1 hour postprandial glucose readings are >7 mmol/l in a 7 day period: commence prandial fast acting insulin analogue (Humalog or Novorapid) 2-4 units with the relevant meal. Uptitrate the dose by 2 units every 3 days aiming for a 1 hour postprandial glucose of ≤7 mmol/l.

- Medication adjustment will be made in accordance with the above guidance.

For peer review only

5.0 Intermittent Low Energy Diet Day Example (supplementary document 3)

6.0 End of Study Questionnaire (supplementary document 4)

LEGENDS

- <image 1>: Figure 1: Inclusion and exclusion criteria
- <image 2>: Figure 2: Participant flow through trial
- <image 3>: Figure 3: Schedule of assessments

For peer review only

Inclusion Criteria

- Pregnant women ≥18 years
- BMI of ≥27.5kg/m2 or a BMI ≥25 kg/m^2 in high risk minority ethnic group (i.e. South Asian, Black African, African Caribbean) and <50 kg/m2 at booking appointment (8-12 weeks' gestation)
- Newly diagnosed GDM according to local diagnostic criteria (fasting glucose ≥5.3mmol/l and/or 2-hour postprandial glucose ≥8.5mmol/l in a 75g OGTT) scheduled to receive first line diet and physical activity (best NHS care)
- 24-30 weeks' pregnant at screening appointment

Exclusion Criteria

- Pregestational type 1 or type 2 diabetes.
- Fasting glucose of ≥7 or 2-hour postprandial of ≥11 on OGTT (immediate intervention with medication would be required in this group of women)
- Current multiple pregnancy
- Maturity Onset Diabetes of the Young (MODY)
- Significant comorbid disease that in PI's opinion would preclude participation in the study e.g. chronic kidney disease, significant cardiac disease, significant history of disordered eating or severe psychological problems.
- Current participation in a GDM medication treatment trial
- People who are not capable of providing informed consent or adhering to the monitoring and safety protocols
- People who have previously had bariatric surgery for weight loss including gastric bypass and sleeve gastrectomy, and/or those prescribed weight loss medications (e.g. orlistat).
- Medications at the time of the OGTT that may interfere with results (e.g. high dose oral steroids, immunosuppressants)
- Previous history of intrauterine growth restriction
- Women who have lost more than 5% of their weight from booking appointment to screening appointment.

Figure 1: Inclusion and exclusion criteria

159x144mm (220 x 220 DPI)

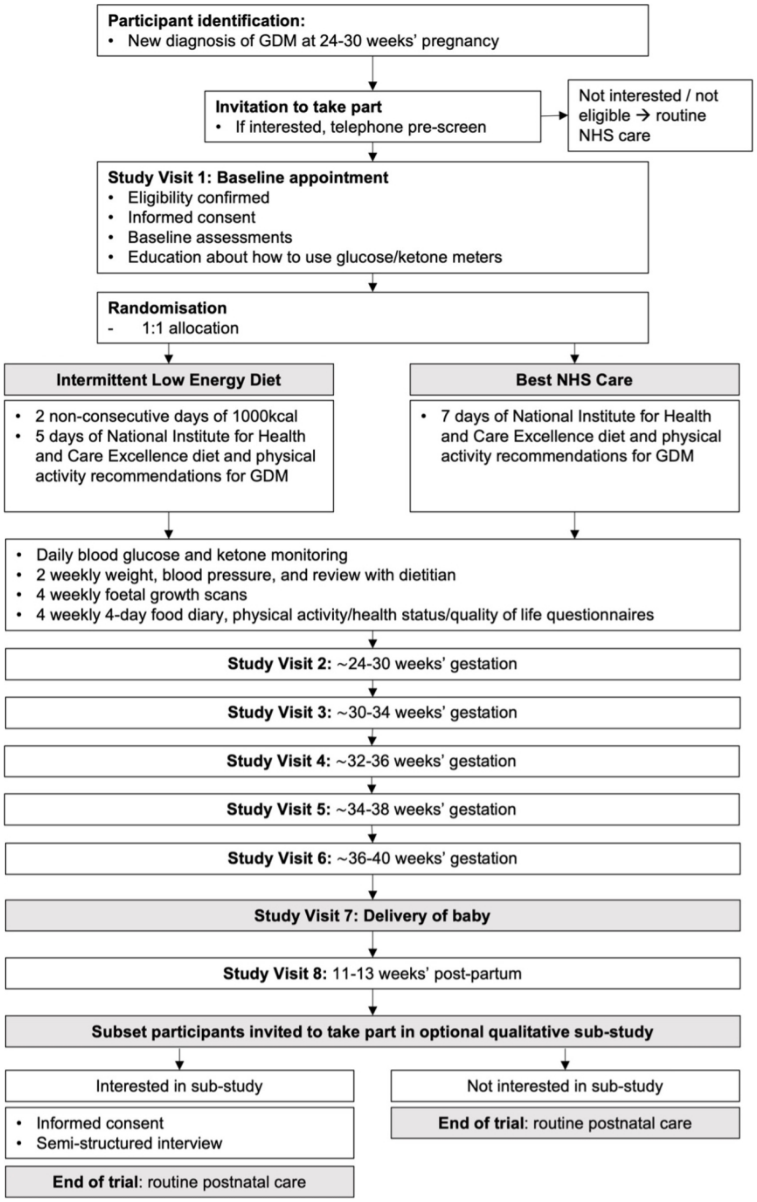

Figure 2: Participant flow through trial

154x245mm (220 x 220 DPI)

	Study Visit							
	1	2	3	4	5	6	7	8
Gestation (weeks)	~24-30	~24-30	~30-34	~32-36	~34-38	~36-40	delivery	11-13 post-partum
Eligibility confirmed	X							
Informed consent	X							
Randomisation	X							
Tailored dietitian review (face to face or remote)	X	X	X	X	X	X		X
Height	X							
Weight^	X	X	X	X	X	X	X	X
Blood Pressure^	X	X	X	X	X	X	X	X
Fasting blood sample*	X				X			X
Questionnaires‖	X		X		X			X
4-day food diary			X		X			X
Foetal growth scan	X		X		X			
Review of glucose and ketone measurements		X	X	X	X	X		
Neonatal measurements†							X	
Oral glucose tolerance test								X
Exit interview / end of study questionnaires								X
Invitation to optional qualitative sub-study$								X

^Frequency of assessment will be 2-4 weekly depending on whether appointment is face-to-face or virtual due to COVID-19 restrictions
*Fasting bloods: urea and electrolytes, liver function tests, bone profile, lipids, thyroid function tests, HbA1c, beta hydroxybutyrate, free fatty acids, full blood count, fasting glucose, insulin
‖Questionnaires: World Health Organisation Quality of Life (brief version), 36-Item Short Form Survey, International Physical Activity Questionnaire (short form), UK Diabetes and Diet Questionnaire
†Neonatal measurements include gestational age at delivery, mode of delivery, neonatal weight, cord blood glucose
$Sub-study involves semi-structured interviews exploring participants' thoughts and experiences of the trial

Figure 3: Schedule of assessments

154x236mm (72 x 72 DPI)

Manchester University NHS Foundation Trust

Consultant Endocrinologist – Dr. Basil Issa
Research Dietitian – Dr. Michelle Harvie
Email: mft.middas.gdm@nhs.net
Tel: 07815987910

The University of Manchester
NIHR | National Institute for Health Research

Nightingale Centre
Manchester University NHS Foundation Trust
Wythenshawe Hospital
Manchester
M23 9LT

MIDDAS-GDM

Manchester Intermittent Diet in Gestational Diabetes Acceptability Study

Participant information sheet

We would like to invite you to take part in a research study **that is testing two different diet programmes which aim to help people with gestational diabetes control their blood sugars.**

If you decide to take part:

- You will be assigned to one of two diet programmes for the duration of your pregnancy. One involves following the standard NHS healthy diet recommendations for pregnancy, and the other follows the standard NHS healthy diet for 5 days/week plus two non-consecutive calorie restricted days of 1,000 kcal per week (both groups will be encouraged to be physically active).
- You will be asked to attend your routine appointments at Wythenshawe or St Marys Hospital and will have fortnightly appointments until delivery of your baby (some appointments may be virtual depending on COVID-19 restrictions). You will be asked to attend the hospital for a blood test 12 weeks after having your baby.
- You will be supported by a diabetes specialist dietitian, midwife, consultant endocrinologist, and your obstetric team throughout the study to help manage your pregnancy and blood glucose safely.
- Throughout the study you will be asked to monitor your food intake via a smartphone/tablet app, or on paper if you prefer, and you will receive feedback on this during your dietary reviews. Comprehensive dietary advice and recipes will be provided.
- Throughout the study you will be asked to monitor your blood sugar using a blood sugar meter four times a day, and you will also be asked to monitor your ketone levels three times on two days of the week (ketones indicate how well your body is using sugar or fat as an energy source). You will be taught how to check your blood sugar and ketone levels.
- If you would like to take part, or you have any questions, then please contact mft.middas.gdm@nhs.net

For peer review only - http://bmjopen.bmj.com/site/about/guidelines.xhtml

This study is being carried out by a team of trained dietitians, doctors, nurses, midwives and researchers under the supervision of Dr. Basil Issa and Dr. Michelle Harvie at Wythenshawe and St Marys hospitals (Manchester University NHS Foundation Trust, MFT).

Before you decide if you would like to take part, it is important for you to understand why the research is being done and what taking part would involve for you. Please take your time to read the following information carefully. Discuss it with your friends, relatives, or GP if you wish to. Take time to consider whether or not you wish to take part.

Please ring the research team at the number at the top of the first page, or e-mail mft.middas.gdm@nhs.net if there is anything that is not clear, or if you would like more information. You can attend an information session about the diets and the study before agreeing to take part if you would like to.

Your participation in the study is entirely voluntary; you do not have to take part if you do not want to and you can opt out of the study at any time without giving a reason. Thank you for reading this information. We hope this research will be of interest to you.

Why are we doing this research?

Around 1 in 8 pregnant women can develop gestational diabetes. This condition causes risks to mother and baby from high blood sugar, high blood pressure, induced labours, caesarean-sections, and larger babies. Women often need medication to control blood sugar despite following recommended NHS healthy eating plans for pregnancy. Intermittent low-calorie diets (two non-consecutive days over the course of the week) improve blood sugar control and reduce the need for medication in patients with type 2 diabetes. We want to find out whether intermittent low-calorie diets might also improve blood sugar control in gestational diabetes and reduce the need for medication as it is a similar condition to type 2 diabetes.

What is the purpose of this research?

This study aims to assess the acceptability (to you) and safety of an intermittent low-calorie diet compared to the usual recommended NHS healthy eating and lifestyle plan for gestational diabetes. **A computer system will randomly allocate you to one of the two diets**. We want to find out which diet is most acceptable to women, whether there is any difference in the two diets' effect on blood sugar control, and any side effects experienced by women. The findings of this study will inform a larger study which will be designed to more closely compare the effect of the two diets on blood sugar control in women with gestational diabetes.

Why have I been asked to take part?

You have been invited to take part in this study because you have been diagnosed with gestational diabetes. We hope to recruit around 48 people to take part in this study.

For peer review only - http://bmjopen.bmj.com/site/about/guidelines.xhtml

NHS
Manchester University
NHS Foundation Trust

MANCHESTER 1824
The University of Manchester

NIHR | National Institute for Health Research

What happens if you agree to take part?

If you agree to take part you will be randomly allocated via a computer system to one of two diet and lifestyle programmes for the remaining weeks of your pregnancy.

Best NHS Care Healthy Diet programme

You will receive personalised advice from a specialist dietician. Recommendations will include increased fruit/vegetable intake, low glycaemic index starchy foods (i.e starchy foods which are slowly absorbed and take a while to raise your blood sugar level), reducing refined sugar, and having regular mealtimes. You will be advised how to design your diet to include the right amount of protein, fats, carbohydrates, and fibre, and will be given meal plans and recipes. You will also be advised to try to complete 150 minutes of moderate intensity exercise a week in addition to strength exercises on at least two days of the week.

Intermittent Low-Calorie Diet programme

If you are allocated to this group you will receive personalised advice to follow a low-calorie diet of 1,000 kcal on two non-consecutive days of the week and the NHS healthy diet on the other five days of the week. The 1,000 kcal days include a set number of portions of protein, carbohydrates and fat foods, fruits, vegetables and dairy/dairy alternatives typically including ~210g (7 oz) of lean protein foods and 3-4 portions of wholegrain carbohydrates, 5 portions of vegetables, 2 of fruit, and 3 of dairy or dairy alternatives and a small amount of healthy fat. You will also be advised to try to complete 150 minutes of moderate intensity exercise a week in addition to strength exercises on at least two days of the week.

Monitoring

You will have all of your usual routine antenatal appointments including checks on your weight, blood pressure, blood tests and ultrasound scans. Extra blood tests will be done as part of the study and these will be added on to samples taken during your routine blood tests.

You will be asked to monitor your blood sugar at home four times a day until your baby is born, and your ketone levels on two days of the week (you will be taught how to do this using a finger prick machine). The results will be recorded when you attend clinic.

We would like to collect a sample of blood from the umbilical cord at delivery to check blood sugar and insulin levels, and your baby’s birthweight will be documented. Guidelines currently recommend that wherever possible the umbilical cord is clamped after 1-2 minutes; this is referred to as ‘delayed cord clamping’ and means that babies receive as much blood as possible from the placenta. This is normal practice. The cord blood sample will not affect your ability to have delayed cord clamping and will not cause any harm to your baby. It will not be possible, however, to delay clamping of the cord for so long that all blood has left the cord (the sample needs to be taken whilst there is still some blood left in the cord).

When babies are born to mothers with gestational diabetes it is normal that their birth weight is recorded and that their blood sugar is monitored for 12 hours following delivery; these results will be recorded by the research team.

For peer review only - http://bmjopen.bmj.com/site/about/guidelines.xhtml

Manchester University NHS Foundation Trust
MANCHESTER 1824 The University of Manchester
NIHR | National Institute for Health Research

You will be asked to attend an additional glucose tolerance test at the hospital 12 weeks after delivery to assess whether you have any residual diabetes (95% of women do not) and also to assess how sensitive your body is to insulin (an important risk factor for the development of diabetes in the future). You will be asked to attend at 9:00am having fasted (no food or drink apart from water) from midnight. A blood sample (around 10 mL/2 teaspoons) will be taken for glucose and insulin and you will be asked to drink a sugary drink with 75 grams of glucose. A further blood sample will be taken after 2 hours for glucose and insulin. You will need to remain in hospital during this time. The reason for this is to help us to understand whether there could be any difference in the body's ability to process sugar between the two diet groups, and also to find out whether any women still have signs of diabetes after pregnancy.

Any blood samples taken as part of the study will be identifiable only using your study identification number and will have none of your personal details. Part of the sample will be sent for immediate analysis and any remaining will be stored securely, accessible only by the research team. At the end of the study any left over samples will be disposed of in accordance with the Human Tissue Act (2004).

You will be asked to record your food intake via the Libro smartphone/tablet app or in a paper diary for four days during four weeks throughout the study. You will also be asked to complete three questionnaires to assess your wellbeing and level of physical activity in these weeks, and a final end of study questionnaire at the final appointment.

Ongoing support from a specialist team of healthcare professionals
Your specialist team includes a Consultant Endocrinologist, Consultant Obstetrician, diabetes specialist dietitian, midwives, and a GP trainee with a special interest in women's health. The specialist team work closely with the usual obstetric teams involved in your care. Reviews with the team will be either face to face when you attend clinic or remotely using video calls.

Mobile Applications and Glucose Meters
The study will use a smartphone application called 'Libro' to help you record information. Libro is a smartphone application which allows you to record your dietary intake during the study. We will ask you to record 4 days of food and drink intake during 4 weeks across the study. Your diaries will be viewed by your allocated dietitian who will provide personalised dietary feedback via the app. You are also free to record more days of your diet should you wish, which some people find helpful. If you do not want to use the mobile app you can use paper instead. You will be supported to set up and use the Nutritics Libro App at your appointments. You do not have to use the application to be part of the study.

Your blood sugar will be monitored using a glucose monitoring device which checks your blood sugar using a 'fingerprick' blood test. You will be shown how to do this yourself. With your permission the research team will make a note of your glucose readings at every visit, either by checking your glucose monitoring device, or by uploading your glucose meter readings onto the computer if you are using a mobile application.

The self-monitoring schedule is as follows:

For peer review only - http://bmjopen.bmj.com/site/about/guidelines.xhtml

Manchester University NHS Foundation Trust

MANCHESTER 1824 The University of Manchester

NIHR | National Institute for Health Research

Intermittent low-energy diet monitoring		Best NHS care monitoring	
Ketones (low kcal days)	Glucose	Ketones (2 days/wk)	Glucose
Fasting before breakfast the morning after each of the low calorie days	Fasting (morning)	Fasting before breakfast on 2 non-consecutive days / week	Fasting (morning)
1 hour post evening meal on each of the low calorie days	1hr post breakfast	1 hour post evening meal on 2 non consecutive days / week	1hr post breakfast
	1hr post lunch		1hr post lunch
	1hr post dinner		1hr post dinner

What should I do if my blood glucose or ketones are out of range?

Low Blood Sugar

You are advised to take 15-20g of 'rapid acting' carbohydrate if your blood glucose is <4 mmol/L). Examples include 170-225ml Lucozade Original (not Lucozade Sport), a small carton of fruit juice, 5-6 glucose tablets, 4/5 jelly babies, or a small tin of cola (150-200ml). You will need to repeat the treatment every 15 minutes until your blood glucose is ≥4 mmol/l.

The following table highlights when you need to consider an additional slower acting carbohydrate:

Situation	Acceptable slow acting carbohydrate
Less than 1 hour before the next meal	Try and avoid
1-2 hour before the next meal	10g (eg half of one of the items below)
More than 2 hours before the next meal	15-20g (eg slice of toast, piece of fruit, small bowl of cereal, glass of milk)

Raised Ketones

If your ketone levels are ⩾1.0 mmol/L on a fasting sample:

- Drink 1L fluids and repeat ketone levels after 4 hours.
- If your ketone level has improved (<1.0mmol/L), no further action is required.
- If your ketone level has increased or remains the same, repeat your ketone level after 2 hours.
- If your ketone level is persistently increased, consume 40g carbohydrates (eg one bagel, bowl of cereal and a banana, small jacket potato), and repeat in 2 hours.
- Continue to do this until your ketone levels are <1.0mmol/L.

Make Immediate Contact with the research team if:

Blood glucose	• Your blood glucose is <3.0 mmol/l or you have symptoms requiring medical attention which are thought to be due to low blood glucose,

For peer review only - http://bmjopen.bmj.com/site/about/guidelines.xhtml

	• Your fasting blood glucose is >5.2 mmol/L on more than a quarter of your measurements on two days in a row,<br>• Your 1 hour post-meal blood glucose is >7.7 mmol/L on more than a quarter of your measurements on two days in a row
Ketones	• Your blood ketones are >1.0 mmol/L

Will I need medications?
If your blood sugars are found to be high despite following the recommended diet and lifestyle programmes you may be advised to start medication to help control your blood sugar. You will be advised on changes to your medications by a diabetes specialist nurse/diabetes midwife and also a Consultant Endocrinologist if required. This is usual practice regardless of whether you are taking part in the study.

What care will I receive after the study has stopped?
At the end of the study, you will be provided appropriate ongoing dietary advice from the study dietitian following your final glucose tolerance test to follow the NHS healthy eating and lifestyle plan. You will receive routine postnatal care from your GP, hospital team, and dietitian if required. You will be advised to see your GP for an annual blood test to check your blood sugar levels (this is routine care for women with gestational diabetes). Approximately 5% of women with gestational diabetes have residual diabetes after delivery. This will be identified from your glucose tolerance test/HbA1c; if this is the case you and your GP will be informed. Your GP will take over the management of your diabetes as per routine care outside the study.

Interview sub study
Women in this study may be invited to take part in an interview at the end of the study . You will be asked about your views and experiences on trying to follow your allocated diet programme. This interview can be arranged at a time that suits you, either at Wythenshawe or St Marys Hospital, at your home, or over the telephone. There is no obligation to take part in this interview study.

Frequently asked questions

Do I have to take part?
No, you do not have to take part if you do not wish to and your decision will not affect any standard of care you receive at Wythenshawe or St Marys hospitals (Manchester University NHS Foundation Trust, MFT).

What happens if I change my mind?
It is OK if you agree to take part in the study but later change your mind. You do not need to give a reason and it will not affect the standard of care you receive. The study team may also choose to withdraw you if it is necessary for your health or safety due to unexpected findings during the study. If you decide to withdraw from the study, or the study is stopped for any reason, you will be asked whether or not you are happy for us to keep the data that may have already been collected. If you do withdraw from the study you will continue to be cared for by your usual specialist diabetes and obstetric teams for the duration of your pregnancy. You will

For peer review only - http://bmjopen.bmj.com/site/about/guidelines.xhtml

NHS Manchester University NHS Foundation Trust

MANCHESTER 1824 The University of Manchester

NIHR | National Institute for Health Research

still have the option of completing the end of study questionnaire and/or interview to provide feedback; this is very useful for the research team to help us understand potential reasons you may have chosen to withdraw from the study.

You will also have the option that if you withdraw, researchers may still collect relevant information about your pregnancy and/or gestational diabetes from your medical records within the 18-month study duration. This will be an option on the consent form.

Are there any benefits from taking part?
You will receive frequent personalised advice and support to follow two diet and lifestyle programmes which may help to control your blood sugar levels throughout your pregnancy. The information gained from this study will also help inform the future NHS care of patients with gestational diabetes.

Are there any risks from taking part?
Research has found that diets consisting of two low-calorie days a week are very low risk. Pregnant women will develop slightly higher levels of ketones when following low calorie diets than women who are not pregnant. Ketones are produced naturally by the body when the body uses fat stores for energy (i.e. when we follow a low calorie diet or haven't eaten enough because we are ill).

Some research suggests that very high levels of ketones throughout pregnancy may cause a higher risk of babies being slightly smaller than average. It is very unlikely that you will develop high levels of ketones by following this diet. You will be provided with a ketone meter and you will be asked to check your ketone levels before your lunch and evening meal on your low-calorie days, and the following morning, to make sure that your ketone levels are normal.

On your low-calorie days you may feel slightly more hungry, or you may experience other effects such as increased nausea, light headedness, or tiredness. It is important that you eat regularly throughout the day to reduce the risk of this happening. You will be asked to report any side effects of following the diet to the team at each appointment.

What happens if my baby or I become unwell during the study?
The safety of you and your baby are of utmost importance and remain our priority. In the instance that either of you become unwell your case will be reviewed by our specialist team and your suitability for continuing in the trial will be decided. Although it remains exceptionally rare, were you to experience the unexpected loss of your baby you will be withdrawn from the trial and supported by the dedicated specialist bereavement team at the hospital. Any information which has been collected as part of the trial will be stored securely and once we have finished the study we will keep some of the data so we can check the results. We will write our reports in a way that no-one can work out that you took part in the study.

What will happen to blood samples which are taken?
Some blood samples taken as part of the study will be sent to the laboratory immediately for analysis and any remaining will be stored securely for the duration of the study. Only your 'study ID' will be used – the samples will have none of your personal details on them. At the end of

For peer review only - http://bmjopen.bmj.com/site/about/guidelines.xhtml

the study any remaining samples will be disposed of in accordance with the Human Tissue Act (2004).

What happens if something goes wrong?
If you have a concern about any aspect of this study, you should ask to speak with the lead researchers who will do their best to answer your questions (**Dr. Basil Issa** or **Dr. Michelle Harvie – via the study office – via michelle.harvie@manchester.ac.uk or telephone 0161 291 4410**). If you remain unhappy and wish to complain formally, you can do so through the NHS complaints procedure. Details can be obtained from the NHS Patient and Liaison Service (PALS) on **Tel:** 0161 276 8686 or contact the team by email pals@mft.nhs.uk.

The hospital is insured to carry out clinical research through the NHS Indemnity scheme. If something did go wrong and you were harmed or suffered deterioration in your health as a result of taking part in this study then you may have grounds for legal action or compensation.

Additional information about the study

Will my lifestyle be affected if I take part?
An essential aspect of this study is a change to your diet and physical activity patterns with support from a specialist team of healthcare professionals.

Payments
We are able to offer free parking at Wythenshawe/St Marys Hospitals for study visits and offer reimbursement for reasonable travel expenses (car, bus or tram) linked to visits for this study. There are no other payments for taking part.

Will my details be kept confidential?
Yes. The study team and any associated regulatory authorities follow strict ethical and legal guidance regarding participant confidentiality. Any information we have about you will be handled in confidence and will only be used for the purposes of this study. All data recorded will be coded and your name will remain anonymous.

During the study we will inform your GP via letter of your participation in the study and your ongoing results, including your weight, blood tests, any abnormal findings and any recommendations for treatment.

If you join the study, some relevant parts of your medical records may be looked at by authorised personnel at Wythenshawe or St Marys hospitals prior to starting the study. These records may also be looked at by an independent auditing body and regulatory authorities to check that the study is being carried out correctly. We will only access parts of your medical records that are relevant to this research and all information accessed will be kept strictly confidential.

How will we use information about you?

For peer review only - http://bmjopen.bmj.com/site/about/guidelines.xhtml

NHS Manchester University NHS Foundation Trust

MANCHESTER 1824 The University of Manchester

NIHR | National Institute for Health Research

We will need to use information from you and from your medical records for this research project.

This information will include the following:

- Initials
- NHS number
- Name
- Contact details
- Medical History including test results

People will use this information to do the research or to check your records to make sure that the research is being done properly. People who do not need to know who you are will not be able to see your name or contact details. Your data will have a code number instead. We will keep all information about you safe and secure.

Once we have finished the study, we will keep some of the data so we can check the results. We will write our reports in a way that no-one can work out that you took part in the study. Other researchers from outside the Trust may ask to see this data for the purposes of furthering their research. We will only share this upon written request to the Trust. The external researchers will be asked to sign a Confidentiality Agreement before any data is shared.

What are your choices about how your information is used?
You can stop being part of the study at any time, without giving a reason, but we will keep information about you that we already have. If you choose to stop taking part in the study, we would like to continue collecting information about your health during pregnancy from your hospital records. If you do not want this to happen, tell us and we will stop. We need to manage your records in specific ways for the research to be reliable. This means that we won't be able to let you see or change the data we hold about you.

Where can you find out more about how your information is used?
You can find out more about how we use your information

- at https://research.cmft.nhs.uk/getting-involved/gdpr-and-research
- by asking one of the research team
- by sending an email to mft.middas.gdm@nhs.net or
- by ringing us on 07815987910

How will my details be used to access the Mobile Applications?
None of your personal details (other than the telephone number from which an application is downloaded) will be needed to access the mobile applications. Once you have given your consent to take part in the study you will be issued with a 'dummy' e-mail and password under a pseudonym (fake name). Only the research team will know the dummy e-mail address you have been assigned to, in order to be able to review your data. The application will not contain your identifiable data. If you choose to use a mobile application to monitor your blood sugar

For peer review only - http://bmjopen.bmj.com/site/about/guidelines.xhtml

Manchester University NHS Foundation Trust

NIHR | National Institute for Health Research

levels the relevant terms of service for the app and the app developers privacy policy will apply. It will be your responsibility to read and understand these prior to download.

Will my insurance be affected if I take part in this study?

It is unlikely that your insurance premiums will be affected by participation in this study as the study has the potential to improve your diabetic control and reduce your risk of ill health. However, if you are at all concerned, then we advise that you contact your insurers and seek expert advice before agreeing to participate.

Who has reviewed this study?

Research in the NHS is looked at by an independent group of people called a Research Ethics Committee (REC). The REC is made up of experts, non-experts and members of the general public. Together they review research applications to ensure your safety, rights, wellbeing and dignity are protected at all times. This study has been reviewed and given favourable opinion by REC.

What will happen to the study results?

It is intended that the results of this study will be presented at conferences and published in medical journals so that we can explain to the medical community what our research results have shown. To do this our study information is double-checked by other professionals in research and healthcare. There is a possibility that the study and its results may be publicised for example on radio, television, magazines, books and websites. **You will not be identified in any publicity, reports or publication arising from this study.** If you would like a general summary of the results of the study you can select this on the consent form or please contact the research team.

Who is organising and funding the research?

Researchers from Wythenshawe hospital, have designed this study and will be carrying out this research. This study has been funded by the National Institute of Health and Research.

Further information and contact details

For further information about this study, please contact **mft.middas.gdm@nhs.net** **or 07815987910**

Thank you for taking the time to read this information sheet.
We hope it has been of interest to you.

For peer review only - http://bmjopen.bmj.com/site/about/guidelines.xhtml

NHS Manchester University NHS Foundation Trust

MANCHESTER 1824 The University of Manchester

NIHR | National Institute for Health Research

Participant pathway for the study

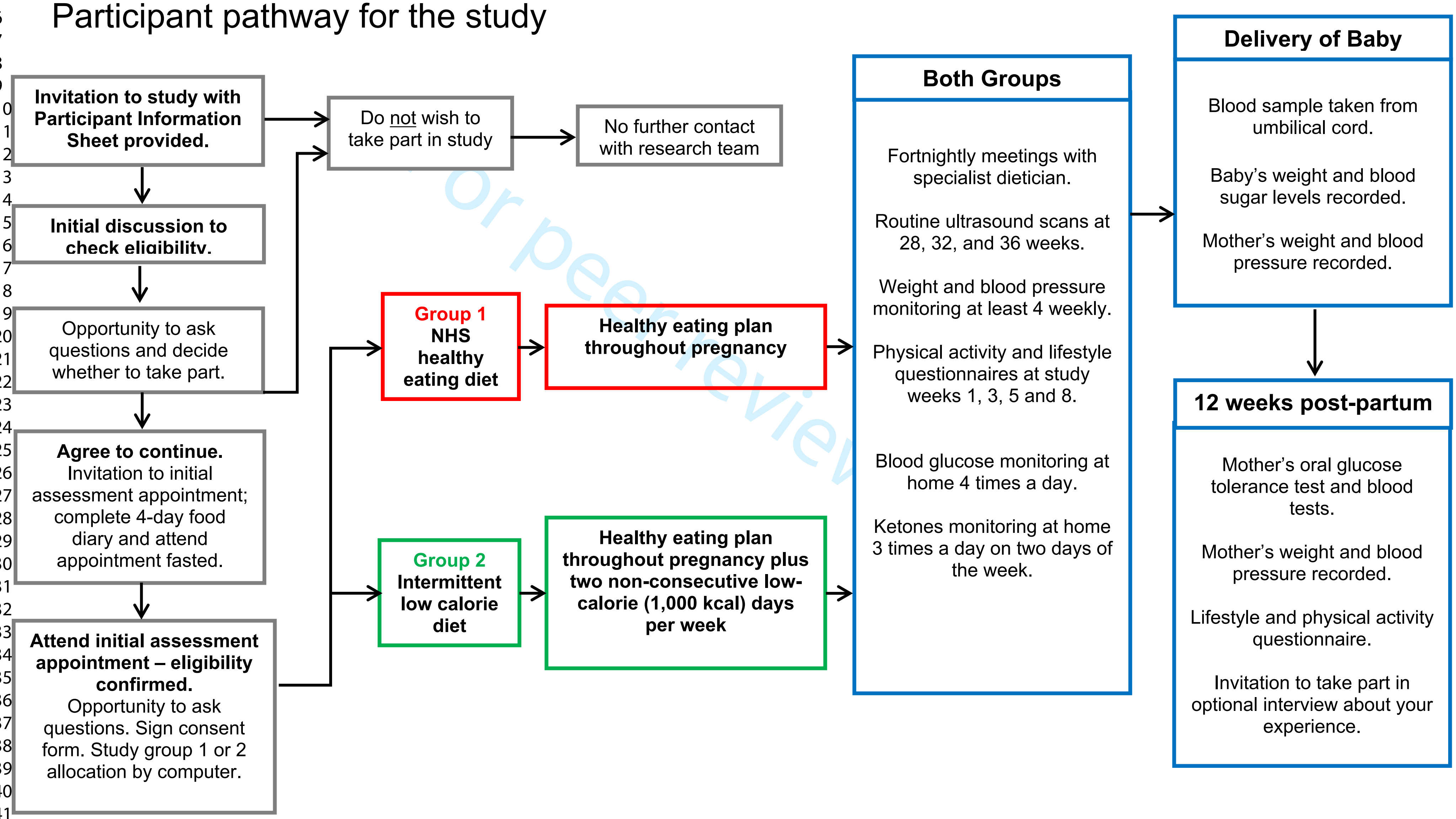

For peer review only - http://bmjopen.bmj.com/site/about/guidelines.xhtml

Example of 1 day meal plan for Diet Day

The diet days aim to limit the calories to 1000 calories per day. You are aiming to include 2 (not consecutive) diet days each week. The other 5 days, follow the Mediterranean diet as described earlier. To keep the calories to 1000, the diet day will look like this:

Mixed diet		Vegetarian/ vegan diet
**4**	Carbohydrate portions	3
**6**	Protein portions	7
**5**	Vegetable portions	5
**2**	Fruit portions	2
**3**	Dairy portions	3
**1**	Fat portions	1

Below are some examples of meals that can be used to help you follow a 1000 calorie diet.. There are options for a mixed diet or vegan or vegetarian options, if you feel you wanted to try meat free days. Filling up on vegetables will make you feel less hungry

For peer review only

Mixed diet options

Breakfast	Portion	Dairy	Protein	Carb	Veg	Fruit	Fat
Grilled lean bacon	1 rasher	0	1	0	0	0	0
Grilled tomatoes	7 cherry tomatoes	0	0	0	1	0	0
Tea/ coffee	1 mug	0	0	0	0	0	0
Midmorning							
Diet or natural yogurt	1 small carton	1	0	0	0	0	0
Lunch							
Wholegrain bread	2 medium slices	0	0	2	0	0	0
Tuna	⅓ of a 120g can	0	1	0	0	0	0
Green salad	Cereal bowl full / 80 g with oil-free dressing	0	0	0	1	0	0
Satsumas	2	0	0	0	0	1	0
Mid afternoon							
Low fat cheese	30g / match box size	1	0	0	0	0	0
Apple slices	I medium apple (80g)	0	0	0	0	1	0
Tea/ coffee		0	0	0	0	0	0
Evening							
Vegetable rice	4 tablespoons cooked rice 160g of mix vegetables	0	0	2	2	0	0
Chicken curry	90g /average chicken breast (no skin) & ½ can tomatoes, 1 desertspoon oil	0	3	0	1	0	1
Bedtime							
Low fat houmous	1 level tablespoon	0	1	0	0	0	0
Pepper sticks	½ red pepper	0	0	0	1	0	0
Milk	1 small glass	1	0	0	0	0	0
**Total portions a day**		**3 portions**	**6 portions**	**4 portions**	**5 portions**	**2 portions**	**1 portion**

For peer review only

Vegetarian option

Breakfast		Dairy	Protein	Carb	Veg	Fruit	Fat
Egg	2 poached	0	2	0	0	0	0
Mushrooms	2 cupped handfuls / 80g	0	0	0	1	0	0
Tea/ coffee	1 mug	0	0	0	0	0	0
Midmorning							
Cheddar cheese	1 match box size / 30g	1	0	0	0	0	0
Cucumber	Sliced handful	0	0	0	1	0	0
Lunch							
Baked beans	2 tablespoons	0	1	0	0	0	0
Seeded bread toasted	1 medium sliced	0	0	1	0	0	0
Blueberries	1 handful	0	0	0	0	1	0
Mid afternoon							
Meat free ham	2 slice small	0	1	0	0	0	0
Pepper	½ sliced	0	0	0	1	0	0
Avocado	¼	0	0	0	0	0	1
Tea/ coffee	1 mug	0	0	0	0	0	0
Evening							
Vegetarian sausage casserole Jacket potato (100g)	1 grilled sausage 2 cereal bowls vegetables 1 ½ egg sized (100 g)	0	2	1	2	0	0
Bedtime							
Pear	1 medium	0	0	0	0	1	0
Low fat cream cheese	1 tablespoon	1	0	0	0	0	0
Whole wheat cracker	2 biscuits	0	0	1	0	0	0
Milk	1 small glass	1	1	0	0	0	0
**Total portions a day**		**3 portions**	**7 portions**	**3 portions**	**5 portions**	**2 portions**	**1 portions**

For peer review only

Vegan options

Breakfast		Dairy equivalent	Protein	Carb	Veg	Fruit	Fat
Branflakes	3 tablespoons	0	0	1	0	0	0
Milk- soya	200 ml	1	0	0	0	0	0
Tea/ coffee	1 mug	0	0	0	0	0	0
Midmorning							
Soya yogurt	3 tablespoons	1	0	0	0	0	0
Lunch							
Kidney bean & Vegetable chilli<br><br>Wholemeal pitta	3 tablespoons of beans 60g with 1 cereal bowl mixed vegetables & 1/2 can chopped tomatoes<br>½ pitta	0	2	1	2	0	0
Banana	1 medium	0	0	0	0	1	0
Mid afternoon							
Low fat hummus	2 level tablespoon	0	2	0	0	0	0
1 carrot	I medium carrot (80g)	0	0	0	1	0	0
Tea/ coffee	1 mug	0	0	0	0	0	0
Evening							
Quinoa	2 tablespoon cooked	0	0	1	0	0	0
Tofu	4 matchbox	0	2	0	0	0	0
Mixed salad with edamame beans	2 x Cereal bowl full with oil free dressing & 1 tablespoons of edamame	0	1	0	2	0	0
Bedtime							
Peanut butter	1 heaped teaspoon	0	0	0	0	0	1
Apple	1 medium sliced	0	0	0	0	1	0
Milk	1 small glass	1	0	0	0	0	0
**Total portions a day**		**3 portions**	**7 portions**	**2 portions**	**5 portions**	**2 portions**	**1 portions**

For peer review only

To help with estimation of portions the following tables outline weight and measures of the different food groups. Where possible household measures are given to make things a little easier. Use these to help you plan your 2 days in the week of 1000 calories.

**Carbohydrate**<br>**4 portions - mixed diet**<br>**3 portions - vegan/vegetarian**	**Equal to**
Wholewheat or oat breakfast cereal, e.g. wholewheat biscuit, malted wholewheat squares, Grapenuts, bran flakes, fruit & fibre	24g or 3 tablespoons or<br>1 whole wheat biscuit
Porridge oats or no-added sugar muesli	20g or 1 heaped tablespoon
Wholegrain, wholemeal, rye, granary bread	36g or medium slice of bread (other than rye), 1½ slices of rye, or ½ roll
Wholemeal or multigrain pitta bread or tortilla wrap, chapatti made without fat	60g or 1x 8" tortilla<br>or 1 standard pitta<br>or small thin chapatti
Rye crispbread, crackers, oak cakes	22g or 2 crispbreads/ 2 oatcakes
Wholegrain rice cake	16g or 2 rice cakes
Wholewheat pasta or rice - cooked amount<br><br>Cous cous, Bulgar wheat, Quinoa, Pearl barley	1 tablespoon uncooked 2 tablespoons cooked<br><br>30g- raw weight or 60g cooked
Lasagne (wholemeal if possible)	20g raw weight or<br>1 large sheet or 1½ smaller sheets
Noodles (wholemeal if possible)	25g raw weight or ½ block/nest
Baked or boiled potato (in skin), cassava, sweet potato	1½ egg sized potatoes or<br>100g raw weight
Wholemeal pizza base (topping is from other food groups)	35g or $^{1}/_{6}$ of thin 10" pizza base
Unsweetened popcorn	20g or 2 handfuls

For peer review only

**Protein**<br>**6 portions – mixed diet**<br>**7 portions – vegan/vegetarian**	**Equal to**
Fresh or smoked white fish (e.g. haddock or cod)	60g or 2oz<br>2 fish finger size
Seafood, e.g. prawns, mussels, crab	45g or 1½oz
Canned tuna or salmon in brine or spring water	45g or 1½oz<br>⅓ standard tin (120g)
Oily fish (fresh or tinned in tomato sauce or olive oil - drained), e.g. mackerel, sardines, salmon, fresh tuna, kippers, smoked salmon or trout	30g or 1oz or<br>¼ standard tin (120g) or<br>¼ fillet of salmon
Chicken, turkey, duck, pheasant (cooked without skin)<br>Lean beef, pork, lamb, rabbit, venison, offal (fat removed)<br>**Quorn fillets, steak, mince or pieces**<br>**Vegetarian mince frozen**	30g or 1oz or<br>1 slice size of playing card
Lean grilled bacon<br>**Quorn ham**	25g or ¾oz or<br>1 rasher
Lean ham<br>**Quorn bacon rashers (not slices)**	30g or 1oz or<br>1 medium, 2 small or 4 wafer thin slices
Eggs	60 g or 2 oz or<br>1 egg
Tofu	50g or $1^{2}/_{3}$ oz or<br>Size of 2 match boxes
Tempeh	25 g or 1 oz or<br>Size of 1 match box
Baked beans (reduced sugar)	60 g or 2 oz or<br>2 tablespoons
Lentils, chickpeas and kidney beans, mung beans, black eye beans, puy lentils, toor dahl, urad dahl,<br>Raw weight<br><br>Cooked or tinned weight	20g or ⅔ oz or<br>1 tablespoon raw<br><br>65g or 2oz or<br>1½ tablespoons cooked /tinned or 1 cupped handful
Soya beans (frozen or cooked) or edamame beans	30g or 1oz or<br>1 tablespoon
Vegetarian sausage	25g or ¾ oz or ½ sausage
Textured vegetable protein (TVP)	10g or ⅓ oz uncooked or<br>1 heaped tablespoon uncooked
Low fat hummus	30g or 1oz or<br>1 level tablespoon

Vegetables – min 5 portions<br>1 portion = 80g or 2⅔oz	1 portion is equal to
Asparagus, Aubergines, Broccoli, Brussel sprouts, Carrots, Cabbage, Cauliflower, Chinese leaves, Courgettes, Cucumber, Curly kale, Green beans, Lettuce (mixed leaves), Mange tout, Methi, Mushrooms, Okra, Pak choi, Peas, Sugar snap, Spinach, Spring greens cooked, Sweetcorn, Tomatoes, Watercress fresh	80g or 2 ⅔ oz or 2 spears of broccoli, 8 cauliflower florets. 3 heaped tablespoons of vegetables or large cereal bowl of salad.

Fruit - 2 portions.<br>1 portion = 80g or 2⅔oz (30g or 1oz dried fruits)	1 portion is equal to
Berries (e.g. blackberries, blueberries, redcurrants, raspberries, strawberries)<br>Cherries or grapes	80g or 2⅔oz<br>1 handful
Grapefruit, guava and mango	80g or 2⅔oz or<br>½ a whole fruit
Large fruit (e.g. melon, pineapple, papaya)	80g or 2⅔oz or<br>1 medium slice
Medium fruits (e.g. apple, pear, nectarine, orange, peach, banana, pomegranate)	80g or 2⅔oz<br>1 fruit
Small fruit (e.g. fresh apricots, kiwi, clementine, passion fruit, plums)	80g or 2⅔oz or<br>2 fruits
Any stewed fruit—unsweetened or with calorie-free sweetener e.g. apple, rhubarb	80g or 2⅔oz or<br>3 tablespoons
Kumquats, lychees, physalis	5 fruits
Dried fruits (raisins, currants, apricots)	30g or 1oz or<br>1 tablespoon

Milk and dairy foods - 3 portions	Equal to
Milk (semi-skimmed or skimmed)<br>Alternative 'milks' with added calcium, e.g. soya **	⅓ pint or 200ml or<br>1 small glass
Diet yoghurts, Low fat/fat-free Greek or Greek Style or natural yoghurts, fromage frais or plain soya yoghurt, high protein yogurt	120-150g or 4-5oz or<br>1 small pot or<br>3 tablespoons
Whole milk natural yogurt	80g or 1 ⅔ oz or<br>2 tablespoons
Cottage cheese	75g or 1½oz or<br>¼ pot, 2 tablespoons
Cream cheese (light or extra light)	30g or 1oz or<br>1 tablespoon
Lower fat hard cheeses e.g.:<br>Reduced fat cheddar, Edam,<br>Bavarian smoked, feta, ricotta, mozzarella, reduced fat halloumi, paneer made from semi-skimmed milk	30g or 1oz or Matchbox size<br>No more than 180g or 6oz a week

** we recommend soya milk as coconut, oat and almond milks are lower in protein and calcium

For peer review only - http://bmjopen.bmj.com/site/about/guidelines.xhtml

**Fat – 2 portions only**	**Equal to**
Margarine or low-fat spread (avoid the buttery types)<br>Olive oil or other oil<br>Oil based dressing<br>Pesto<br>Mayonnaise	8g or 1 teaspoon<br>1 dessertspoon of oil
Seeds (e.g. linseed, pumpkin, sunflower, sesame, chia, hemp)	7g or 1 dessertspoon
Unsalted or salted or dry roasted nuts	7g or 1 dessertspoon<br>3 walnut halves, 3 Brazil,<br>4 almonds, 8 peanuts,<br>10 cashews or pistachios
Olives	50g or 10 olives
Low fat mayonnaise, Curry paste or Harissa paste	15g or ½ oz or 1 tablespoon
Peanut butter (without palm oil)	11g or ⅓ oz or<br>1 heaped teaspoon
Cocoa powder	12 g or ⅓ oz or<br>2 heaped teaspoons
Avocado	40g or 1⅓ oz or<br>1/4 of an average pear
Low fat guacamole	40g or 1⅓ oz or 2 tablespoons

For peer review only

Manchester University
NHS Foundation Trust

MIDDAS-GDM End of Study Questionnaire

Thank you for taking part in the MIDDAS-GDM Study.

This is one of the first studies of its kind. We hope to learn as much as possible from this study, in particular the views of people who have taken part. We are inviting you to provide your views on different aspects of the study and following the diet, and how we can improve our programmes and research studies in future.

Please complete the following questions and return this questionnaire to the MIDDAS-GDM study team in the envelope provided. If there is anything else you would like to say about your experiences of the study, please use the section at the end. Your answers to the questions below will remain anonymous.

1. What were your reasons for joining the study?

………

………

………

………

………

2. How satisfied were you with study overall (recruitment, appointments etc)? *(circle)*

1	2	3	4	5	6	7	8	9	10
Not at all satisfied		Slightly satisfied		Quite satisfied		Very satisfied			Extremely satisfied

Comments:……………………………………………………………………………………………

………

………

Study Appointments

3. You were asked to attend ……….. additional face to face appointments at the hospital by the study team *(please fill in the number)*

For peer review only - http://bmjopen.bmj.com/site/about/guidelines.xhtml

NHS Manchester University NHS Foundation Trust

4. **You were asked to attend ……….. additional virtual (i.e. video call or telephone) appointments by the study team** *(please fill in the number)*

5. **How do you feel about the number of additional appointments you were asked to attend?** *(tick)*

☐ I was happy with the number of appointments
☐ I would have preferred fewer face to face appointments
☐ I would have preferred more face to face appointments
☐ I would have preferred fewer virtual appointments
☐ I would have preferred more virtual appointments

Comments:……………………………………………………………………………………………
……………………………………………………………………………………………………

6. **How do you feel about virtual (i.e. video call or telephone) appointments?**

☐ I prefer virtual appointments to face to face appointments (please explain why below)
☐ I prefer face to face appointments to virtual appointments (please explain why below)

Comments:……………………………………………………………………………………………
……………………………………………………………………………………………………
……………………………………………………………………………………………………
……………………………………………………………………………………………………

Diet

7. **Which diet were you asked to follow? (*please tick*)**

☐ Best NHS Care (i.e. increased fruit/vegetable intake, low-GI foods, reduction in free sugars, regular meals)
☐ Intermittent low energy diet (5 days of the best NHS care diet plus 2 non-consecutive days of 1000 kcal each week)

8. **Was the diet easy to follow?**

1	2	3	4	5	6	7	8	9	10
Not at all		Slightly		Moderately		Very			Extremely

For peer review only - http://bmjopen.bmj.com/site/about/guidelines.xhtml

Manchester University
NHS Foundation Trust

Comments:……………………………………………………………………………………………

……………………………………………………………………………………………………

9. Did you enjoy following the diet plan?

1	2	3	4	5	6	7	8	9	10
Not at all		Slightly		Moderately		Very			Extremely

Comments:……………………………………………………………………………………………

……………………………………………………………………………………………………

10. Would you make any changes to the written information (i.e. diet booklets, recipes) you were given on how to follow the diet plan?

☐ Yes

☐ No

If yes, what changes would you make?

Comments:……………………………………………………………………………………………

……………………………………………………………………………………………………

11. How was your first appointment with the dietitian? *(tick all that apply)***?**

☐ The amount of information I received was OK

☐ The amount of information I received was too little

☐ The amount of information I received was too much

☐ I was happy with the advice I received

☐ The advice I received could be improved (specify below)

Comments:……………………………………………………………………………………………

……………………………………………………………………………………………………

For peer review only - http://bmjopen.bmj.com/site/about/guidelines.xhtml

Manchester University
NHS Foundation Trust

12. Would you make any changes to the reviews (calls/face to face appointments) you had with the dietitian during your pregnancy ?

☐ Yes

☐ No

Comments:……………………………………………………………………………………………

……………………………………………………………………………………………………

13. How useful was your final appointment with the dietitian at 12 weeks post-delivery?

1	2	3	4	5	6	7	8	9	10
Not at all		Slightly		Quite		Very			Extremely

Comments:……………………………………………………………………………………………

……………………………………………………………………………………………………

14. Did you feel confident to exercise whilst on the diet plan?

1	2	3	4	5	6	7	8	9	10
Not at all confident		Slightly confident		Quite confident		Very confident			Extremely confident

Comments:……………………………………………………………………………………………

……………………………………………………………………………………………………

For peer review only

<u>Additional support</u>

15. Would any of the following have been useful & if so how often?

	No	Yes	Preferred method of contact	How often
Additional support from dietitian	☐	☐	Face to face / phone	
Additional support from the midwife	☐	☐	Face to face / phone	

For peer review only - http://bmjopen.bmj.com/site/about/guidelines.xhtml

Manchester University
NHS Foundation Trust

Additional support from the doctors in the clinic	☐	☐	Face to face / phone	
More contact with other women in the study following the diets	☐	☐	Face to face / phone	
Other, please specify:				

16. Did you receive any support outside of the study team help to keep you on track as you progressed through the study?

☐ No

☐ Yes

If yes, what support did you receive?

………

………

Record keeping

17. How did you find the finger prick testing requirements on the study? *(tick all those that apply)*

☐ Challenging but on the whole achievable

☐ Challenging and not achievable

☐ Not challenging at all

☐ I felt it was necessary to test this often to ensure my safety

☐ I felt it was unnecessary to test this often to ensure my safety

Comments:……………………………………………………………………………………………

………

18. How did you find the ketone testing requirements on the study? *(tick all those that apply)*

☐ Challenging but on the whole achievable

☐ Challenging and not achievable

For peer review only - http://bmjopen.bmj.com/site/about/guidelines.xhtml

NHS
Manchester University
NHS Foundation Trust

☐ Not challenging at all

☐ I felt it was necessary to test this often to ensure my safety

☐ I felt it was unnecessary to test this often to ensure my safety

Comments:……………………………………………………………………………………………

……………………………………………………………………………………………..............

19. How did you find using Diasend software?

☐ Straightforward

☐ Challenging but on the whole achievable

☐ Challenging and not achievable

☐ I felt uncomfortable using computer software to keep track of my medical details

☐ I felt comfortable using computer software to keep track of my medical details

Comments:……………………………………………………………………………………………

……………………………………………………………………………………………..............

20. How did you find completing the food diary during the study? *(tick all those that apply)*

☐ Challenging but on the whole achievable

☐ Challenging and not achievable

☐ Not challenging at all

Comments:……………………………………………………………………………………………

……………………………………………………………………………………………..............

21. How did you find the physical activity questionnaires on the study? *(tick all those that apply)*

☐ Challenging but on the whole achievable

☐ Challenging and not achievable

☐ Not challenging at all

Comments:……………………………………………………………………………………………

……………………………………………………………………………………………..............

For peer review only

For peer review only - http://bmjopen.bmj.com/site/about/guidelines.xhtml

Manchester University
NHS Foundation Trust

22. How did you find the quality of life questionnaires on the study? *(tick all those that apply)*

☐ Challenging but on the whole achievable

☐ Challenging and not achievable

☐ Not challenging at all

Comments:……………………………………………………………………………………………

……………………………………………………………………………………………………

Libro® app

23. Did you use the Libro® app?

☐ Yes

☐ No (please move to question 25)

24. Did you find the App helpful?

1	2	3	4	5	6	7	8	9	10
Not at all		Slightly		Moderately		Very			Extremely

Comments……………………………………………………………………………………………

……………………………………………………………………………………………………

25. What did you like about the App?

……………………………………………………………………………………………………

……………………………………………………………………………………………………

……………………………………………………………………………………………………

26. What did you dislike about the App and could be improved?

……………………………………………………………………………………………………

……………………………………………………………………………………………………

27. If you didn't use the app what were the reasons for this? *(tick all that apply)*

☐ Would prefer not to say ☐ Already using other health apps

For peer review only - http://bmjopen.bmj.com/site/about/guidelines.xhtml

Manchester University
NHS Foundation Trust

- ☐ Don't like using apps in general
- ☐ Not user friendly
- ☐ Labour intensive / time consuming
- ☐ Prefer to use pen and paper
- ☐ Find mobile devices challenging
- ☐ Lack of regular internet access
- ☐ Other (provide details below)

……………………………………………………………………………………………

……………………………………………………………………………………………

……………………………………………………………………………………………

Diasend Software

28. Did you find the Diasend software helpful?

1	2	3	4	5	6	7	8	9	10
Not at all		Slightly		Moderately		Very			Extremely

Comments……………………………………………………………………………………

……………………………………………………………………………………………

29. What did you like about the Diasend software?

……………………………………………………………………………………………

……………………………………………………………………………………………

30. What did you dislike about the Diasend software and could be improved?

……………………………………………………………………………………………

……………………………………………………………………………………………

Study Improvements

31. What did you enjoy most about the study?

……………………………………………………………………………………………

……………………………………………………………………………………………

……………………………………………………………………………………………

……………………………………………………………………………………………

For peer review only

For peer review only - http://bmjopen.bmj.com/site/about/guidelines.xhtml

Manchester University
NHS Foundation Trust

32. What did you enjoy least about the study and could be improved?

……

……

……

……

……

Any other comments about the study

……

……

……

……

……

……

……

……

Thank you for completing this questionnaire, please return to:

MIDDAS-GDM Study Team, Nightingale Centre,

Wythenshawe Hospital, Manchester, M23 9LT

For peer review only - http://bmjopen.bmj.com/site/about/guidelines.xhtml

MeSH Descriptors

Infant, newborn
Pregnancy
Glucose Intolerance
Diabetes, Gestational
Insulin
Hyperglycaemia
Prediabetic state
Metformin
Glycated Hemoglobin
Overweight
Obesity
Diabetes Mellitus, Type 2
Diet, Healthy
Feasibility Studies
Mobile Applications
Body Weight
Hypoglycaemic agents
Fasting
Intermittent Fasting

For peer review only

SPIRIT CHECKLIST

Section/Item	Item no
Title	1
Trial Registration	2a
	2b
Protocol Version	3
Funding	4
Roles and responsibilities	5a
	5b
	5c
	5d
**Introduction**	
Background and rationale	6a
	6b
Objectives	7
Trial Design	8
**Methods: Participants, interventions, and outcomes**	
Study setting	9
Eligibility criteria	10
Interventions	11a

For peer review only

	11b
	11c
	11d
Outcomes	12
Participant Timeline	13
Sample Size	14
Recruitment	15
**Methods: Assignment of interventions (for controlled tri**	
Allocation	
Sequence generation	16a
Allocation concealment mechanism	16b
Implementation	16c

For peer review only

Blinding (masking)	17a
	17b
**Methods: Data collection, management, and analysis**	
Data collection methods	18a
	18b
Data management	19
Statistical methods	20a
	20b
	20c
**Methods: Monitoring**	
Data monitoring	21a

For peer review only

	21b
Harms	22
Auditing	23
**Ethics and dissemination**	
Research ethics approval	24
Protocol amendments	25
Consent or assent	26a
	26b
Confidentiality	27
Declaration of interests	28
Access to data	29
Ancillary and post-trial care	30
Dissemination policy	31a

For peer review only

	31b
	31c
**Appendices**	
Informed consent materials	32
Biological specimens	33

For peer review only

Description	Location
Descriptive title identifying the study design, population, interventions, and, if applicable, trial acronym	1
Trial identifier and registry name. If not yet registered, name of intended registry	3
All items from the World Health Organization Trial Registration Data Set	throughout
Date and version identifier	n/a
Sources and types of financial, material, and other support	23
Names, affiliations, and roles of protocol contributors	1
Name and contact information for the trial sponsor	name only, 23
Role of study sponsor and funders, if any, in study design; collection, management, analysis, and interpretation of data; writing of the report; and the decision to submit the report for publication, including whether they will have ultimate authority over any of these activities	23
Composition, roles, and responsibilities of the coordinating centre, steering committee, endpoint adjudication committee, data management team, and other individuals or groups overseeing the trial, if applicable (see Item 21a for data monitoring committee)	18
Description of research question and justification for undertaking the trial, including summary of relevant studies (published and unpublished) examining benefits and harms for each intervention	4-5
Explanation for choice of comparators	4-5
Specific objectives or hypotheses	13-14
Description of trial design including type of trial (eg, parallel group, crossover, factorial, single group), allocation ratio, and framework (eg, superiority, equivalence, noninferiority, exploratory)	6
Description of study settings (eg, community clinic, academic hospital) and list of countries where data will be collected. Reference to where list of study sites can be obtained	6
Inclusion and exclusion criteria for participants. If applicable, eligibility criteria for study centres and individuals who will perform the interventions (eg, surgeons, psychotherapists)	7
Interventions for each group with sufficient detail to allow replication, including how and when they will be administered	9

For peer review only

Criteria for discontinuing or modifying allocated interventions for a given trial participant (eg, drug dose change in response to harms, participant request, or improving/worsening disease)	n/a
Strategies to improve adherence to intervention protocols, and any procedures for monitoring adherence (eg, drug tablet return, laboratory tests)	13
Relevant concomitant care and interventions that are permitted or prohibited during the trial	7
Primary, secondary, and other outcomes, including the specific measurement variable (eg, systolic blood pressure), analysis metric (eg, change from baseline, final value, time to event), method of aggregation (eg, median, proportion), and time point for each outcome. Explanation of the clinical relevance of chosen efficacy and harm outcomes is strongly recommended	13-14
Time schedule of enrolment, interventions (including any run-ins and washouts), assessments, and visits for participants. A schematic diagram is highly recommended (see Figure)	11-12
Estimated number of participants needed to achieve study objectives and how it was determined, including clinical and statistical assumptions supporting any sample size calculations	8
Strategies for achieving adequate participant enrolment to reach target sample size	n/a
**als)**	
Method of generating the allocation sequence (eg, computer-generated random numbers), and list of any factors for stratification. To reduce predictability of a random sequence, details of any planned restriction (eg, blocking) should be provided in a separate document that is unavailable to those who enrol participants or assign interventions	8
Mechanism of implementing the allocation sequence (eg, central telephone; sequentially numbered, opaque, sealed envelopes), describing any steps to conceal the sequence until interventions are assigned	8
Who will generate the allocation sequence, who will enrol participants, and who will assign participants to interventions	7-8

For peer review only

Who will be blinded after assignment to interventions (eg, trial participants, care providers, outcome assessors, data analysts), and how	8
If blinded, circumstances under which unblinding is permissible, and procedure for revealing a participant's allocated intervention during the trial	8
Plans for assessment and collection of outcome, baseline, and other trial data, including any related processes to promote data quality (eg, duplicate measurements, training of assessors) and a description of study instruments (eg, questionnaires, laboratory tests) along with their reliability and validity, if known. Reference to where data collection forms can be found, if not in the protocol	9-15
Plans to promote participant retention and complete follow-up, including list of any outcome data to be collected for participants who discontinue or deviate from intervention protocols	supplementary PIS
Plans for data entry, coding, security, and storage, including any related processes to promote data quality (eg, double data entry; range checks for data values). Reference to where details of data management procedures can be found, if not in the protocol	16
Statistical methods for analysing primary and secondary outcomes. Reference to where other details of the statistical analysis plan can be found, if not in the protocol	16
Methods for any additional analyses (eg, subgroup and adjusted analyses)	16
Definition of analysis population relating to protocol non-adherence (eg, as randomised analysis), and any statistical methods to handle missing data (eg, multiple imputation)	16
Composition of data monitoring committee (DMC); summary of its role and reporting structure; statement of whether it is independent from the sponsor and competing interests; and reference to where further details about its charter can be found, if not in the protocol. Alternatively, an explanation of why a DMC is not needed	18

For peer review only

Description of any interim analyses and stopping guidelines, including who will have access to these interim results and make the final decision to terminate the trial	n/a
Plans for collecting, assessing, reporting, and managing solicited and spontaneously reported adverse events and other unintended effects of trial interventions or trial conduct	13, 16
Frequency and procedures for auditing trial conduct, if any, and whether the process will be independent from investigators and the sponsor	16
Plans for seeking research ethics committee/institutional review board (REC/IRB) approval	n/a
Plans for communicating important protocol modifications (eg, changes to eligibility criteria, outcomes, analyses) to relevant parties (eg, investigators, REC/IRBs, trial participants, trial registries, journals, regulators)	19
Who will obtain informed consent or assent from potential trial participants or authorised surrogates, and how (see Item 32)	7, supplementary consent
Additional consent provisions for collection and use of participant data and biological specimens in ancillary studies, if applicable	n/a
How personal information about potential and enrolled participants will be collected, shared, and maintained in order to protect confidentiality before, during, and after the trial	16
Financial and other competing interests for principal investigators for the overall trial and each study site	23
Statement of who will have access to the final trial dataset, and disclosure of contractual agreements that limit such access for investigators	19
Provisions, if any, for ancillary and post-trial care, and for compensation to those who suffer harm from trial participation	supplementary PIS
Plans for investigators and sponsor to communicate trial results to participants, healthcare professionals, the public, and other relevant groups (eg, via publication, reporting in results databases, or other data sharing arrangements), including any publication restrictions	19

For peer review only

Authorship eligibility guidelines and any intended use of professional writers	n/a
Plans, if any, for granting public access to the full protocol, participant- level dataset, and statistical code	19
Model consent form and other related documentation given to participants and authorised surrogates	supplementary materials
Plans for collection, laboratory evaluation, and storage of biological specimens for genetic or molecular analysis in the current trial and for future use in ancillary studies, if applicable	15

For peer review only

**Missing**

For peer review only

For peer review only

For peer review only

form

For peer review only

For peer review only

TIDieR
Template for Intervention Description and Replication

The TIDieR (Template for Intervention Description and Replication) Checklist*:

Information to include when describing an intervention and the location of the information

Item number	Item	Where located ** Primary paper (page or appendix number)	Other † (details)
	**BRIEF NAME**		
**1.**	Provide the name or a phrase that describes the intervention.	2	
	**WHY**		
**2.**	Describe any rationale, theory, or goal of the elements essential to the intervention.	4-5	______________
	**WHAT**		
**3.**	Materials: Describe any physical or informational materials used in the intervention, including those provided to participants or used in intervention delivery or in training of intervention providers. Provide information on where the materials can be accessed (e.g. online appendix, URL).	12, 15	_appendix____
**4.**	Procedures: Describe each of the procedures, activities, and/or processes used in the intervention, including any enabling or support activities.	6-12	______________
	**WHO PROVIDED**		
**5.**	For each category of intervention provider (e.g. psychologist, nursing assistant), describe their expertise, background and any specific training given.	1, 7-12, 17-18	______________
	**HOW**		

For peer review only - http://bmjopen.bmj.com/site/about/guidelines.xhtml

**6.**	Describe the modes of delivery (e.g. face-to-face or by some other mechanism, such as internet or telephone) of the intervention and whether it was provided individually or in a group.	6-18	______________
	**WHERE**		
**7.**	Describe the type(s) of location(s) where the intervention occurred, including any necessary infrastructure or relevant features.	6-18	______________
	**WHEN and HOW MUCH**		
**8.**	Describe the number of times the intervention was delivered and over what period of time including the number of sessions, their schedule, and their duration, intensity or dose.	6-18	______________
	**TAILORING**		
**9.**	If the intervention was planned to be personalised, titrated or adapted, then describe what, why, when, and how.	6-18	______________
	**MODIFICATIONS**		
**10.‡**	If the intervention was modified during the course of the study, describe the changes (what, why, when, and how).	N/A	______________
	**HOW WELL**		
**11.**	Planned: If intervention adherence or fidelity was assessed, describe how and by whom, and if any strategies were used to maintain or improve fidelity, describe them.	13-14	______________
**12.‡**	Actual: If intervention adherence or fidelity was assessed, describe the extent to which the intervention was delivered as planned.	N/A	______________

For peer review only

For peer review only - http://bmjopen.bmj.com/site/about/guidelines.xhtml

** **Authors** - use N/A if an item is not applicable for the intervention being described. **Reviewers** – use '?' if information about the element is not reported/not sufficiently reported.

† If the information is not provided in the primary paper, give details of where this information is available. This may include locations such as a published protocol or other published papers (provide citation details) or a website (provide the URL).

‡ If completing the TIDieR checklist for a protocol, these items are not relevant to the protocol and cannot be described until the study is complete.

* We strongly recommend using this checklist in conjunction with the TIDieR guide (see *BMJ* 2014;348:g1687) which contains an explanation and elaboration for each item.

* The focus of TIDieR is on reporting details of the intervention elements (and where relevant, comparison elements) of a study. Other elements and methodological features of studies are covered by other reporting statements and checklists and have not been duplicated as part of the TIDieR checklist. When a **randomised trial** is being reported, the TIDieR checklist should be used in conjunction with the CONSORT statement (see www.consort-statement.org) as an extension of **Item 5 of the CONSORT 2010 Statement.** When a **clinical trial protocol** is being reported, the TIDieR checklist should be used in conjunction with the SPIRIT statement as an extension of **Item 11 of the SPIRIT 2013 Statement** (see www.spirit-statement.org). For alternate study designs, TIDieR can be used in conjunction with the appropriate checklist for that study design (see www.equator-network.org).

For peer review only

For peer review only - http://bmjopen.bmj.com/site/about/guidelines.xhtml

BMJ Open

Manchester Intermittent Diet in Gestational Diabetes Acceptability Study (MIDDAS-GDM): A Two-Arm Randomised Feasibility Protocol Trial of an Intermittent Low-Energy Diet (ILED) in women with Gestational Diabetes and Obesity in Greater Manchester

Journal:	*BMJ Open*
Manuscript ID	bmjopen-2023-078264.R1
Article Type:	Protocol
Date Submitted by the Author:	09-Nov-2023

Complete List of Authors:	Dapre, Elizabeth; Wythenshawe Hospital<br>Issa, Basil; Manchester University NHS Foundation Trust, Department of Endocrinology and Diabetes<br>Harvie, Michelle; University Hospital of South Manchester NHS Foundation Trust, Genesis prevention centre<br>Su, Ting-Li; University of Manchester, Dentistry<br>McMillan, Brian; The University of Manchester, Centre for Primary Care and Health Services Research<br>Pilkington, A.; Wythenshawe Hospital<br>Hanna, F.; University Hospitals of North Midlands NHS Trust<br>Vyas, Avni; Manchester Metropolitan University Faculty of Health Psychology and Social Care, Health Professionals<br>Mackie, S.; Wythenshawe Hospital<br>Yates, James; Manchester University NHS Foundation Trust<br>Evans, Benjamin; Manchester University NHS Foundation Trust<br>Mubita, Womba; Manchester University NHS Foundation Trust, Department of Endocrinology and Diabetes<br>Lombardelli, Cheryl; Manchester University NHS Foundation Trust

**Primary Subject Heading**:	Diabetes and endocrinology
Secondary Subject Heading:	Nutrition and metabolism, Obstetrics and gynaecology, Reproductive medicine
Keywords:	Diabetes in pregnancy < DIABETES & ENDOCRINOLOGY, DIABETES & ENDOCRINOLOGY, Obesity, NUTRITION & DIETETICS, Feasibility Studies, Maternal medicine < OBSTETRICS

SCHOLARONE™
Manuscripts

Manchester Intermittent Diet in Gestational Diabetes Acceptability Study (MIDDAS-GDM): A Two-Arm Randomised Feasibility Protocol Trial of an Intermittent Low-Energy Diet (ILED) in women with Gestational Diabetes and Obesity in Greater Manchester

Authors: Dapre, E., Issa, B., Harvie, M., Su, T., McMillan, B., Hanna, F., Pilkington, A., Vyas, A., Yates, J., Mackie, S., Evans, B., Mubita, W., Lombardelli, C.

Dr Elizabeth Dapre (corresponding author)	elizabeth.dapre@hotmail.com<br>c/o Michelle Harvie, The Nightingale Centre, Wythenshawe Hospital, Southmoor Rd, Manchester M23 9LT
Dr Basil Issa	Diabetes and Endocrinology, Wythenshawe Hospital, Manchester, UK
Dr Michelle Harvie	The Nightingale Centre, Wythenshawe Hospital, Manchester, UK
Dr Ting-Li Su	Division of Dentistry, The University of Manchester, UK
Dr Brian McMillan	Division of Population Health, The University of Manchester, UK
Prof Fahmy Hanna	Diabetes and Endocrinology, University Hospitals of North Midlands, UK
Dr Andrea Pilkington	Obstetrics, St Mary's Wythenshawe Hospital, Manchester, UK
Avni Vyas	Division of Dietetics, Manchester Metropolitan University, UK
Benjamin Evans	Division of Clinical Trials, Manchester Foundation Trust, UK
James Yates	Division of Clinical Trials, Manchester Foundation Trust, UK
Sarah Mackie	Division of Clinical Trials, Manchester Foundation Trust, UK
Womba Mubita	Division of Clinical Trials, Manchester Foundation Trust, UK
Cheryl Lombardelli	The Nightingale Centre, Wythenshawe Hospital, Manchester, UK

Word Count: 3572

Submitted to: British Medical Journal Open

Start date of trial: 15th November 2022 (trial ongoing)

Date of submission:

For peer review only

<u>M</u>anchester <u>I</u>ntermittent <u>D</u>iet in Gestational <u>D</u>iabetes <u>A</u>cceptability <u>S</u>tudy (MIDDAS-GDM): A Two-Arm Randomised Feasibility Protocol Trial of an Intermittent Low-Energy Diet (ILED) in women with Gestational Diabetes and Obesity in Greater Manchester

Authors: Dapre, E., Issa, B., Harvie, M., Su, T., McMillan, B., Hanna, F., Pilkington, A., Vyas, A., Yates, J., Mackie, S., Evans, B., Mubita, W., Lombardelli, C.

Corresponding Author: elizabeth.dapre@nhs.net

For peer review only

Abstract (word count 298)

Introduction: The prevalence of gestational diabetes mellitus (GDM) is rising in the UK and is associated with maternal and neonatal complications. National Institute for Health and Care Excellence (NICE) guidance advises first line management with healthy eating and physical activity which is only moderately effective for achieving glycaemic targets. Approximately 30% of women require medication with metformin and/or insulin. There is currently no strong evidence base for any particular dietary regimen to improve outcomes in GDM. Intermittent low-energy diets (ILEDs) are associated with improved glycaemic control and reduced insulin resistance in type 2 diabetes (T2DM) and could be a viable option in the management of GDM. This study aims to test the safety, feasibility and acceptability of an ILED intervention amongst women with GDM compared to best National Health Service (NHS) care.

Method and analysis: We aim to recruit 48 women with GDM diagnosed between 24-28 weeks gestation from antenatal clinics at Wythenshawe and St Mary's hospitals, Manchester Foundation Trust, over 13 months starting in November 2022. Participants will be randomised (1:1) to ILED (2 low-energy diet days/week of 1000kcal and 5 days/week of the best NHS care healthy diet and physical activity advice) or best NHS care 7 days/week until delivery of their baby. Primary outcomes include uptake and retention of participants to the trial, and adherence to both dietary interventions. Safety outcomes will include birthweight, gestational age at delivery, neonatal hypoglycaemic episodes requiring intervention, neonatal hyperbilirubinaemia, admission to special care baby unit or neonatal intensive care unit, stillbirths, the percentage of women with hypoglycaemic episodes requiring

third-party assistance, and significant maternal ketonaemia (defined as ≥1.0mmol/L) Secondary outcomes will assess the fidelity of delivery of the interventions, and qualitative analysis of participant and healthcare professionals' experiences of the diet. Exploratory outcomes include the number of women requiring metformin and/or insulin.

For peer review only

Ethics and dissemination: Ethical approval has been granted by the Cambridge East Research Ethics Committee (22/EE/0119). Findings will be disseminated via publication in peer-reviewed journals, conference presentations, and shared with diabetes charitable bodies and organisations in the UK, such as Diabetes UK and the Association of British Clinical Diabetologists.

Trial Registration Number: NCT05344066

89

Strengths and limitations of this study
**Strengths**
• To the best of our knowledge this is the first study to look at intermittent diets in women with gestational diabetes
• This study adds to the limited literature of safety of low-calorie diets amongst women with gestational diabetes
• This study has been informed by an experienced patient and public involvement and engagement group
**Limitations**
• This study involves a small sample size and is not powered to show efficacy of the intervention
• Women joining this study are likely to be highly motivated and adherence may not reflect that seen in the wider general population

INTRODUCTION (word count: 3539)

Background

In the UK up to 16% of pregnant women develop gestational diabetes (GDM) and the incidence is rising, in part due to increasing rates of obesity and maternal age(1,2). GDM is associated with maternal and neonatal complications (the risk increases with poor glycaemic control), including macrosomia, shoulder dystocia, caesarean-sections, neonatal hypoglycaemia and/or hyperbilirubinaemia, preterm delivery, preeclampsia, and stillbirth(2). Women who have had GDM have an estimated seven to ten-fold risk of developing type 2 diabetes (T2DM) later in life, and their children have a higher risk of developing adult obesity and T2DM(2–4).

Excessive weight gain in pregnancy is a particular problem for women with GDM(5). Harper *et al* demonstrated that, in women with GDM, every additional 1lb/week gained following diagnosis of GDM resulted in a 36-83% increased risk of pre-eclampsia, caesarean-section, macrosomia, and large for gestational age babies(5). Such studies highlight the importance of adequate weight control throughout pregnancy in women with GDM in order to reduce both maternal and neonatal complications.

For peer review only

First-line therapy for GDM is diet and physical activity. National Institute for Health and Care Excellence (NICE) guidance encourages a healthy diet with increased fruit and vegetables, low-glycaemic index (GI) foods, reduced refined sugars, regular mealtimes and regular physical activity(6,7). These dietary measures fail to achieve glycaemic targets in ~30% of women who require medication with metformin and/or insulin(8). A range of dietary approaches have been studied including daily diets promoting low-GI diets (limiting refined and promoting complex carbohydrates), continuous modest energy-restriction (1800 Kcal/day), and low carbohydrate diets(9). There is currently no strong evidence base for any particular dietary regimen to improve outcomes in GDM.

Intermittent Low-Energy Diets (ILED)

The pathogenesis of GDM is strongly linked to obesity and chronic insulin resistance with many clinicians viewing GDM as a form of evolving T2DM. ILEDs typically include several days of a food based or meal replacement (e.g. drinks/bars) low-energy diet (650-1000kcal) diet, with a standard healthy (non-restrictive) diet recommended on the remaining days of the week. These diets are associated with significant reductions in weight, insulin resistance and hyperglycaemia in patients with prediabetes (HbA1c between 42-47mmol/mol, impaired glucose tolerance, or impaired fasting glycaemia), those with T2DM, and otherwise healthy subjects with overweight/obesity(10–17). These changes are equivalent to, or greater than, those achieved with standard daily energy restriction. A popular intermittent diet involves 2 consecutive or non-consecutive days/week of a low-energy diet (650-1000kcal) and 5 days of normal eating/week, known as the 5:2 diet. The Manchester Intermittent vs. Daily Diabetes App Study (MIDDAS), a study comparing an ILED and a continuous low-energy diet in T2D conducted in our unit, has shown the feasibility and safety of an ILED (800kcal for 2 days/week) in patients with T2DM and obesity, including those using insulin(18). At the end of the study approximately 70% of participants in the ILED group completed the study and achieved a 6% reduction of their baseline body weight. Forty two percent achieved an HbA1c of <48 mmol/mol(18). Given the strong overlap between GDM and T2DM, an ILED may be a promising dietary intervention for those with GDM.

For peer review only

A successful dietary approach to glycaemic control could empower women to take charge of the management of their GDM. Women with GDM are motivated to modify their diet driven by a desire to improve foetal outcomes(19–21).

Our Patient and Public Involvement and Engagement (PPIE) work indicates that women find the current National Institute for Health and Care Excellence (NICE) healthy eating guidance(6,7) confusing and vague. Our PPIE work has indicated that women are keen to try alternative dietary approaches, particularly if alternative diets are more effective in preventing the need to progress to medications such as metformin and insulin.

Aim

The aim of this trial is to test the safety, feasibility, and acceptability of an ILED in GDM to inform a future large-scale RCT.

METHODS

Trial Design

The study is a 28-week feasibility two-arm RCT in one NHS trust performed in patients with GDM and BMI ≥27.5 kg/m^2, or ≥25 kg/m^2 in high-risk minority ethnic groups (i.e. South Asian, Black African, African Caribbean) in Greater Manchester, between December 2022 and July 2024(22,23). There will be an embedded qualitative sub-study for participants and healthcare professionals. Due to the nature of the intervention, it will not be possible to blind the participants, clinicians, or study team to the treatment allocation after randomisation (the statistician and laboratory technicians will remain blinded).

Trial Setting and Recruitment

Participants will be recruited from antenatal clinics at Wythenshawe and St Mary's Hospitals, Manchester Foundation Trust (MFT) between November 2022 and December 2023. This is an urban area within Greater Manchester, and MFT serves patients from a wide range of minority ethnic and socio-demographic backgrounds. Women may self-refer to the antenatal clinic or be referred by their primary care team. Assessments will be carried out at MFT, or remotely if required by COVID-19 restrictions. The qualitative sub-study will be carried out at MFT, remotely, or at a location of the participant's choosing. We aim to recruit eligible participants over a

For peer review only

period of 13 months. Potential participants will be given written information about the study and the opportunity to ask questions about the study prior to providing written consent (figure 1).

Eligibility Criteria

<IMAGE ONE; figure1 inclusion exclusion jpg>

Participant Flow

Participants who fulfil the broad eligibility criteria will be notified about the trial by the GDM nurse/midwife at the time of their diagnosis. Those who are interested will be provided with a comprehensive patient information sheet (see appendix) and more detailed eligibility screening questions. They will be asked to attend their first appointment having fasted for at least 6 hours and complete a four-day food diary (in line with our departments usual care). On attending their first routine clinic appointment, interested participants will receive further information from the research team. They will have the opportunity to ask questions, have their eligibility confirmed, and will be asked for their written consent to take part. Baseline assessments will be taken and participants will be randomised to their allocated treatment group using an online randomisation platform. Participant flow through the study is demonstrated in figure 2.

Sample Size

We plan to recruit 24 participants per study arm (n=48) which, when considering an estimated attrition rate of 15%, will provide complete outcome data on 40 participants(24–26). It has been estimated that 24 participants per group will be sufficient to determine study outcomes, in line with sample size recommendations for feasibility studies(27–29).

This number will allow us to enable estimation of recruitment/retention parameters with sufficient precision. For example, based on 40 completed participants, it will enable recruitment rates in the region of 25% to be estimated with an error of +/-13.42% at most; retention of 85% will be estimated with error of +/-11.07% at most. It is also sufficient for estimation of variability (e.g. standard deviation) in gestational

For peer review only

weight gain and capillary glucose concentrations (proposed outcomes for the future definitive trial) with negligible bias (30).

Randomisation

The randomisation schedule will be independently set up and known only by the trial statistician. The trial statistician will be blinded to the participant's identity using "sealed envelope" software (https://www.sealedenvelope.com/). Randomisation will be carried out by generating an online pseudo-random list with random permuted blocks of varying size, known only to the statistician, and will be stratified for two variables:

- Age (18-35, >35 years)
- BMI (27.5-34.99kg/m^2 and >35kg/m2, >25-32.49kg/m^2 and >32.5kg/m^2 for high-risk minority ethnic groups (i.e. South Asian, Black African, African Caribbean)

These stratification variables have been chosen to reduce potential bias as we expect varying severity of GDM with increasing age and BMI, and possible differences in diet adherence(31).

Treatment to intervention and control groups will be allocated in a 1:1 ratio. A member of the research team who will be unaware of the randomisation algorithm (principal investigator, clinical research nurse, clinical research fellow or project manager) will trigger the randomisation procedure onsite; participants and clinicians will then be informed of the allocated treatment group. Clinicians will not be blinded due to the need to remain astute to safety, adherence, and side effects, requiring open and honest discussions with patients at each appointment. The statistician will remain blinded to treatment allocation until all outcome measures for all subjects have been collected.

Interventions

Study Arm 1: Best NHS Care Diet

All dietetic advice will be face to face or via video calls or the telephone. Participants will receive one to one personalised written and verbal advice from a dietitian to follow NICE diet and physical activity recommendations(6,7). Dietitians and midwives will receive training to ensure standardised delivery of information in clinic, and

standardised patient information leaflets will be supplied to include information about increased fruit/vegetable intake, low-glycaemic index foods, and a reduction in free sugars. Information will include advice about the importance of regular meals; dietary advice aims to ensure that participants include at least 70g protein/28g fibre, and predominantly mono- and polyunsaturated fats as per American Diabetes Association recommendations(32). Participants will be advised to be physically active, for example walking for 30 minutes after a meal. Participants will receive ongoing dietetic education and support every 2 weeks until delivery. This level of support is higher than typically provided in NHS GDM antenatal clinics due to limited resources but has been utilised to reflect best NHS care. They will receive suggested menus and recipes to follow the NICE recommended healthy diet for GDM. Participants will be asked to measure their capillary glucose four times each day and their ketones on two random (recorded) days of the week of their choosing (see appendix).

Study Arm 2: Intermittent Low-Energy Diet (ILED)

Participants will receive the same level of dietetic support as the best NHS care group. They will be given advice on adopting an ILED which involves 2 non-consecutive low-energy diet days/week (1000kcal to include 100g low-GI carbohydrate and 70g of protein) and 5 days/week of the NICE healthy eating low-GI diet and physical activity recommended for the best NHS care group. The low-energy days involve women selecting a set number of portions of protein, carbohydrate, fat, fruit, vegetables, and dairy/dairy alternatives as described in previous studies(33). Each low-energy day includes ~210g of lean protein foods, 3-4 portions of wholegrain carbohydrates, 1x7g portion of fat, 5 portions of vegetables, 2 of fruit, and 3 of dairy/dairy alternatives. Food and drink will be self-selected and not provided by the study team. Participants will be provided with comprehensive food lists, advice on portion sizes for the low-energy days and suggested menus and recipes to follow for both the low-energy and NICE recommended healthy diet days. Both diets can be successfully adapted for people of different ethnicities and those following omnivorous, vegetarian and vegan diets. Participants will be asked to measure their capillary glucose four times each day and their ketones on (and the morning after) the two low-energy days (see appendix).

For peer review only

<IMAGE 2: figure 2 flow jpg>

<IMAGE 3: figure 3 sched assessments jpg>

Outcomes

Primary outcomes

- Uptake rate measured as a percentage of eligible participants who consent to take part, including the proportion of women who were screened who did not meet the eligibility criteria, and the number of women who did not give consent to take part
- Recruitment rate measured as the number of eligible participants who consent to take part per month
- Retention rate measured as the number of randomised participants who complete the trial (those who attend the final visit) and the percentage of participants who attend all 8 visits
- Adherence to the dietary interventions assessed from self-reported adherence to the potential low-calorie days between randomisation and delivery
- Completion of self-assessed glucose and ketone readings assessed as a percentage of the required readings

- Safety outcomes:
    - Percentage of women following ILED/best NHS care with hypoglycaemia (episodes of blood glucose of <3.0mmol/mol) and hypoglycaemia requiring third-party assistance as measured by participants
    - Percentage of women who develop significant ketonaemia in both groups (defined as ≥1.0mmol/L) as measured by participants
    - Percentage of neonatal hypoglycaemic episodes requiring intervention (blood glucose checked 2- hours post-delivery and 2-hours thereafter for 12 hours according to local protocol), neonatal birth weight, gestational age at delivery, hyperbilirubinaemia/jaundice, and/or

For peer review only

admission to Special Care Baby Unit or neonatal intensive care, and stillbirths

- The incidence and rate of other adverse events (e.g. headaches, lethargy, constipation, or complications requiring hospital admission) between the start of the trial intervention and delivery recorded as mild, moderate and severe, as defined by Common Terminology Criteria for Adverse Events version 5 (CTCAEv5)(34). Hospital admission for routine labour and delivery will not be classified as an adverse event.

Secondary outcomes

- Completeness of collection of trial endpoints including the percentage of completed weight measurements, 4-day food diaries, and International Physical Activity Questionnaire (IPAQ) scores
- Fidelity of delivery of the interventions will be measured through the number and modality of completed planned patient contacts, electronic and paper food diaries, and self-reported capillary glucose and ketone measurements
- Qualitative analysis of the acceptability and implementation of the interventions will be explored amongst a subset of participants (~10 in each group) and healthcare professionals through in-depth interviews

Exploratory outcomes

The following outcomes will be explored without statistical inference.

1. Maternal outcomes:
   - The percentage of women requiring metformin and/or insulin
   - Four-point capillary glucose profiles during third trimester (four times daily until delivery)
   - Change in fasting blood test results between baseline measurements, 36-37 weeks' gestation, and 12 weeks post-delivery (including oral glucose tolerance tests (OGTT)
   - Mode of delivery, development of preeclampsia, polyhydramnios (maximum liquor volume pool depth ≥8 cm)
   - Quality of life and health status questionnaires (WHOQoL-BREF and SF-36 questionnaires)(35,36)
2. Foetal outcomes:

For peer review only

- Foetal weight
- Gestational age at delivery

Measurements

The full schedule of assessments can be found in figure 3.

Physical measurements

Height, weight and blood pressure will be measured using standardised calibrated equipment in antenatal clinic.

Blood samples

Fasting venous blood samples will be collected to assess maternal HbA1c, fasting glucose, insulin, beta-hydroxybutyrate, liver function tests, free fatty acids, thyroid function tests, and full blood count. A cord blood sample will be collected at the time of delivery to measure neonatal glucose, and insulin and C-peptide where collection is possible. At the end of the study all samples will be disposed of in accordance with the Human Tissue Act (2004).

Questionnaires

Participants will be asked to complete four questionnaires at four time points throughout the trial (self-reported). Quality of life and health status will be assessed using the World Health Organisation Quality of Life Questionnaire (brief version) and the 36-Item Short Form Survey respectively(35,36). Physical activity will be measured using the International Physical Activity Questionnaire – Short Form, and diet quality will be assessed using the UK Diabetes and Diet Questionnaire(37,38). These questionnaires are self-reported by participants and have been chosen as they are widely used and validated tools.

For peer review only

Food Diaries

4-day dietary records will be completed using Libro (Nutritics Mobile Application) or paper food diaries, which will be entered into Nutritics software (Nutritics, Dublin, Ireland)(39). Participants who wish to use Libro will receive one to one training to use this by the study dietitian. Diaries will provide the research team with information about the intake of energy, carbohydrate, fat, protein, fibre, glycaemic index, and the timing of meals for participants in both groups. Participants will be asked what other dietary modifications, if any, they have made at their fortnightly dietitian reviews to establish the adoption of any alternative dietary practices in the cohort.

Adverse Events

Participants in both groups will be asked about any adverse effects that they have experienced at each visit. These will include, but are not limited to, the potential effects of a low-energy diet, e.g. headache, lethargy, dizziness, constipation, indigestion, poor concentration, and hunger. Adverse events will be graded as per CTAEv5(34). Participants will be issued with a participation/emergency card with emergency contact details for the research team to be carried at all times and to be shown to the attending physician in case of emergency admission to hospital. All participants will be issued with clear instructions as to how to manage a hypoglycaemic and/or ketonaemic event (see appendix).

Data management

Participant data will be anonymised and will be stored in line with the Medicines for Human Use (Clinical Trials) Amended Regulations 2006 and the Data Protection Act (2018) and archived in line with the Medicines for Human Use (Clinical Trials) Amended Regulations (2006) as defined in the MFT Clinical Trials Office Archiving SOP (11; Retention of Data, Off-Site Archiving, and Destroying Documents). Deidentified data will be stored in a study-specific Research Electronic Data Capture (REDCap) database. The sponsor will periodically audit the site study file, a sample of the case report form, consent forms, and source data, and check accuracy of the study database to ensure satisfactory completion.

Statistical methods

A statistical analysis plan specifying the full details of the primary and secondary outcomes, other variables, and methods, will be produced prior to trial analysis. The

For peer review only

main analysis will be conducted via intention-to-treat population and will not undertake any significance tests. Descriptive, graphical (summary), and basic statistics (e.g. i. number, frequencies and percentages, ii. mean and standard deviation, or iii. median and quartiles as appropriate) will be presented as appropriate for each group respectively, for group difference jointly, and for each stratum. Per-protocol analysis will be considered as a secondary analysis. Levels of missing data will be investigated and used to inform future studies. No imputation will be used. The end of study questionnaire will be analysed using appropriate descriptive statistics for closed questions and key themes will be extracted without formal analysis from open questions to inform future research.

Progression Criterion

The success of the feasibility trial will be defined by the progression criteria as outlined in table 1. Any concerns regarding a low retention rate will be discussed with the PPIE group. Interviews will include those who withdraw from the study to address potential reasons for withdrawal with the aim to improve retention in future.

	Feasible (green)	Feasible with modification of the protocol (amber)	Not feasible (red)
**Recruitment**	≥4 patients/month	>2 patients/month	≤2 patients/month
**Uptake to the feasibility study**	≥15%	10-15%	<10%
**Retention to the feasibility study**	>70%	50-70%	<50%
**Adherence to the ILED intervention**	>50% of the low-energy days completed (2/week between weeks 24-28 and delivery)	30-50% of the low-energy days completed (2/week between weeks 24-28 and delivery)	<30% of the low-energy days completed (2/week between weeks 24-28 and delivery)

Table 1: Trial progression criterion

Qualitative sub-study

For peer review only

Participants will be invited to take part in an optional qualitative sub-study at 11-13 weeks post-partum. Healthcare professionals delivering the interventions will also be invited to take part in this study.

We will undertake 11-12 semi-structured interviews with a subset of women from each group (ILED n=10 and best NHS Care n=10) at around 12 weeks post-delivery. The final sample size will be contingent on obtaining data saturation. We will also interview a sample of healthcare professionals (HCPs) involved in the delivery of care to study participants, including dieticians, obstetricians and midwives, including those with leadership and clinical managerial roles. Sampling will be purposive, aiming to obtain women from a range of ethnic groups, ages, socioeconomic backgrounds, and self-reported engagement with the intervention. Participants and HCPs will be asked about their experiences and thoughts regarding the intervention, including motivating factors, and facilitators/barriers to engagement. Interviews will be conducted by a researcher from the University of Manchester/MFT who is independent from the research staff involved in the delivery and assessment of the programmes. Analysis will be conducted by two independent researchers at the University of Manchester/MFT using Braun and Clarke's thematic analysis approach to identify key issues around the acceptability, usefulness of the programmes, and feasibility of a subsequent trial(40). Analysis will be inductive: open-ended, exploratory, and driven by the data.

All participants will also be asked to complete an optional and anonymous end of study questionnaire developed by the study team at their post-partum visit (see appendix). This will give participants the opportunity to feedback on their experience and will enable the study team to identify improvements to the design of a possible follow-up study.

Trial Steering Committee (TSC)

The trial steering committee will include an independent consultant endocrinologist, obstetrician, dietitian, and the patient representative. The committee will oversee the trial to ensure that it is carried out to the expected standards. The TSC will liaise with the CI to develop a schedule of meetings, proposed to occur every four months, with meetings to occur no less than annually. Minutes will be taken at TSC meetings and

For peer review only

copies of the minutes will be filed in the Trial Master File; they will be shared with relevant stakeholders as appropriate.

Patient and public involvement

Patient and public involvement was actively sought throughout the planning and design of this trial and continues to form a key part of the trial as it progresses. The patient and public involvement and engagement (PPIE) group assisted in the development of all participant materials and provided valuable insight into the wording of participant information and acceptability of the proposed intervention. The PPIE group will be updated as the trial progresses and a further focus group will be held to advise on the interview schedule and wording for the qualitative sub-study. The group will also be invited to aid in the development of summarising key findings for dissemination to relevant patient groups.

Ethics and dissemination

This study has been approved by the Cambridge East Research Ethics Committee and is sponsored by MFT. Findings will be disseminated via publication in peer-reviewed journals, conference presentations, and shared with diabetes charitable bodies and organisations in the UK, such as Diabetes UK and the Association of British Clinical Diabetologists. Anonymised data will be available upon formal request once the principal results of the study have been published. Planned modifications to the protocol will be approved by the research ethics committee before they are adopted into the study. An audit trail of ethical amendments and documentation will allow monitoring by the research team and external regulatory bodies.

This is the first study to assess the feasibility and safety of an ILED in GDM as compared to best NHS care. Given the increasing incidence of GDM and associated health risks this research is both pertinent and important. The study is not powered to show differences between ILED and best NHS care, however the planned quantitative and qualitative assessments will inform the feasibility of the programme and a future definitive trial.

For peer review only

Full References

1. The British Diabetic Association. Diabetes UK. [cited 2019 Nov 27]. What is gestational diabetes? Available from: https://www.diabetes.org.uk/diabetes-the-basics/gestational-diabetes

2. Kim C. Gestational diabetes: risks, management, and treatment options. Int J Womens Health. 2010 Oct 7;2:339–51.

3. Kim SY, Sharma AJ, Callaghan WM. Gestational diabetes and childhood obesity: what is the link? Curr Opin Obstet Gynecol. 2012 Dec;24(6):376–81.

4. Vounzoulaki E, Khunti K, Abner SC, Tan BK, Davies MJ, Gillies CL. Progression to type 2 diabetes in women with a known history of gestational diabetes: systematic review and meta-analysis. BMJ. 2020 May 13;m1361.

5. Harper L, Tita A, Biggio J. The Institute of Medicine Guidelines for Gestational Weight Gain after a Diagnosis of Gestational Diabetes and Pregnancy Outcomes. Am J Perinatol. 2014 Jun 27;32(03):239–46.

6. Webber J, Charlton M, Johns N. Diabetes in pregnancy: management of diabetes and its complications from preconception to the postnatal period (NG3). Br J Diabetes. 2015 Sep 1;15(3):107.

7. NHS. NHS Treatment Gestational Diabetes [Internet]. 2019 [cited 2020 Jan 10]. Available from: https://www.nhs.uk/conditions/gestational-diabetes/treatment/

8. Mayo K, Melamed N, Vandenberghe H, Berger H. The impact of adoption of the international association of diabetes in pregnancy study group criteria for the screening and diagnosis of gestational diabetes. Am J Obstet Gynecol. 2015 Feb;212(2):224.e1-9.

For peer review only

9. Han S, Middleton P, Shepherd E, Van Ryswyk E, Crowther CA. Different types of dietary advice for women with gestational diabetes mellitus. Cochrane Database Syst Rev. 2017 25;2:CD009275.

10. Carter S, Clifton PM, Keogh JB. Effect of Intermittent Compared With Continuous Energy Restricted Diet on Glycemic Control in Patients With Type 2 Diabetes: A Randomized Noninferiority Trial. JAMA Netw Open. 2018 Jul 20;1(3):e180756.

11. Antoni R, Johnston KL, Collins AL, Robertson MD. Effects of intermittent fasting on glucose and lipid metabolism. Proc Nutr Soc. 2017 Aug;76(3):361–8.

12. Cioffi I, Evangelista A, Ponzo V, Ciccone G, Soldati L, Santarpia L, et al. Intermittent versus continuous energy restriction on weight loss and cardiometabolic outcomes: a systematic review and meta-analysis of randomized controlled trials. J Transl Med. 2018 Dec;16(1):371.

13. Harris L, Hamilton S, Azevedo LB, Olajide J, De Brún C, Waller G, et al. Intermittent fasting interventions for treatment of overweight and obesity in adults: a systematic review and meta-analysis. JBI Database Syst Rev Implement Rep. 2018 Feb;16(2):507–47.

14. Harvie M, Howell A. Potential Benefits and Harms of Intermittent Energy Restriction and Intermittent Fasting Amongst Obese, Overweight and Normal Weight Subjects—A Narrative Review of Human and Animal Evidence. Behav Sci. 2017 Jan 19;7(4):4.

15. Harvie MN, Pegington M, Mattson MP, Frystyk J, Dillon B, Evans G, et al. The effects of intermittent or continuous energy restriction on weight loss and metabolic disease risk markers: a randomized trial in young overweight women. Int J Obes. 2011 May;35(5):714–27.

16. Wang X, Li Q, Liu Y, Jiang H, Chen W. Intermittent fasting versus continuous energy-restricted diet for patients with type 2 diabetes mellitus and metabolic syndrome for glycemic control: A systematic review and meta-analysis of randomized controlled trials. Diabetes Res Clin Pract. 2021 Sep;179:109003.

17. He S, Wang J, Zhang J, Xu J. Intermittent Versus Continuous Energy Restriction for Weight Loss and Metabolic Improvement: A Meta-Analysis and Systematic Review. Obesity. 2021 Jan;29(1):108–15.

18. McDiarmid S, Harvie M, Johnson R, Vyas A, Aglan A, Moran J, et al. Manchester Intermittent versus Daily Diet App Study (MIDDAS): A pilot randomized controlled trial in patients with type 2 diabetes. Diabetes Obes Metab. 2022 Mar;24(3):432–41.

19. Carolan M. Women's experiences of gestational diabetes self-management: A qualitative study. Midwifery. 2013 Jun;29(6):637–45.

20. Gao F, Luo H, Jones K, Nicholson W, Bell RA. Gestational Diabetes and Health Behaviors Among Women: National Health and Nutrition Examination Survey, 2007–2014. Prev Chronic Dis. 2018 Oct 25;15:180094.

For peer review only

21. McMillan B, Easton K, Goyder E, Delaney B, Madhuvrata P, Abdelgalil R, et al. Reducing risk of type 2 diabetes after gestational diabetes: a qualitative study to explore the potential of technology in primary care. Br J Gen Pract. 2018 Apr;68(669):e260–7.

22. National Institute for Health and Care Excellence. Diabetes in pregnancy: management from preconception to the postnatal period. NICE guideline. [Internet]. 2020. Available from: https://www.nice.org.uk/guidance/ng3/resources/diabetes-in-pregnancy-management-from-preconception-to-the-postnatal-period-pdf-51038446021

23. National Institute for Health and Care Excellence. Obesity: identification, assessment and management. NICE guideline. [Internet]. 2014 Nov [cited 2023 Aug 30]. Available from: https://www.nice.org.uk/guidance/cg189/resources/obesity-identification-assessment-and-management-pdf-35109821097925

24. H. Al Wattar B, Dodds J, Placzek A, Beresford L, Spyreli E, Moore A, et al. Mediterranean-style diet in pregnant women with metabolic risk factors (ESTEEM): A pragmatic multicentre randomised trial. Persson LÅ, editor. PLOS Med. 2019 Jul 23;16(7):e1002857.

25. Moreno-Castilla C, Hernandez M, Bergua M, Alvarez MC, Arce MA, Rodriguez K, et al. Low-carbohydrate diet for the treatment of gestational diabetes mellitus: a randomized controlled trial. Diabetes Care. 2013 Aug;36(8):2233–8.

26. Rae A, Bond D, Evans S, North F, Roberman B, Walters B. A randomised controlled trial of dietary energy restriction in the management of obese women with gestational diabetes. Aust N Z J Obstet Gynaecol. 2000 Nov;40(4):416–22.

27. Julious SA. Sample size of 12 per group rule of thumb for a pilot study. Pharm Stat. 2005 Oct;4(4):287–91.

28. Sim J, Lewis M. The size of a pilot study for a clinical trial should be calculated in relation to considerations of precision and efficiency. J Clin Epidemiol. 2012 Mar;65(3):301–8.

29. Lancaster GA. Pilot and feasibility studies come of age! Pilot Feasibility Stud. 2015 Dec;1(1):1, 2055-5784-1–1.

30. Teare MD, Dimairo M, Shephard N, Hayman A, Whitehead A, Walters SJ. Sample size requirements to estimate key design parameters from external pilot randomised controlled trials: a simulation study. Trials. 2014 Dec;15(1):264.

31. Makgoba M, Savvidou M, Steer P. An analysis of the interrelationship between maternal age, body mass index and racial origin in the development of gestational diabetes mellitus: Gestational diabetes mellitus and risk factors. BJOG Int J Obstet Gynaecol. 2012 Feb;119(3):276–82.

32. American Diabetes Association. 13. Management of Diabetes in Pregnancy: *Standards of Medical Care in Diabetes—2018*. Diabetes Care. 2018 Jan;41(Supplement 1):S137–43.

For peer review only

33. Harvie M, Wright C, Pegington M, McMullan D, Mitchell E, Martin B, et al. The effect of intermittent energy and carbohydrate restriction *v* . daily energy restriction on weight loss and metabolic disease risk markers in overweight women. Br J Nutr. 2013 Oct 28;110(8):1534–47.

34. National Cancer Institute. Cancer Therapy Evaluation Program. 2017 [cited 2020 May 7]. Common Terminology Criteria for Adverse Events (CTCAE). Available from: https://ctep.cancer.gov/protocolDevelopment/electronic_applications/ctc.htm#ctc_50

35. World Health Organisation. WHOQOL: Measuring Quality of Life [Internet]. Available from: https://www.who.int/tools/whoqol

36. Nair R, Kachan P. Outcome tools for diabetes-specific quality of life: Study performed in a private family practice clinic. Can Fam Physician Med Fam Can. 2017 Jun;63(6):e310–5.

37. Ekelund U, Sepp H, Brage S, Becker W, Jakes R, Hennings M, et al. Criterion-related validity of the last 7-day, short form of the International Physical Activity Questionnaire in Swedish adults. Public Health Nutr. 2006 Apr;9(2):258–65.

38. England CY, Thompson JL, Jago R, Cooper AR, Andrews RC. Development of a brief, reliable and valid diet assessment tool for impaired glucose tolerance and diabetes: the UK Diabetes and Diet Questionnaire. Public Health Nutr. 2017 Feb;20(2):191–9.

39. Nutritics [Internet]. [cited 2020 Jul 12]. Available from: https://www.nutritics.com/p/home?utm_source=adwords&utm_campaign=Nutritics+Brand&utm_term=&utm_medium=ppc&hsa_grp=78385303355&hsa_mt=b&hsa_kw=&hsa_src=g&hsa_ad=380634402696&hsa_cam=1619635404&hsa_net=adwords&hsa_acc=2361676125&hsa_ver=3&hsa_tgt=dsa-814211721343&gclid=Cj0KCQjw6ar4BRDnARIsAITGzlAm_uy2YNRBU5N-egXXyehUQ_9CHKylLQI_LgCYJK9Jpet4oIvUM5saAkzREALw_wcB

40. Braun V, Clarke V. Successful qualitative research: a practical guide for beginners. Los Angeles: SAGE; 2013. 382 p.

Authors' contributions

Michelle Harvie, Basil G. Issa, Elizabeth Dapre, Brian McMillan, Ting-Li Su, Fahmy Hanna, Andrea Pilkington and Avni Vyas designed the study, wrote the protocol, and secured the funding. Elizabeth Dapre drafted the manuscript for publication, with input from Michelle Harvie, Basil G. Issa, Brian McMillan, and Ting-Li Su. All other authors have proofed and checked the manuscript.

Acknowledgments

With thanks to Mrs Rebecca Lumsden, our patient expert, whose insight has been invaluable in the design of the study. We are most grateful to Mrs Jodie Aspinall, and Miss Lisa Brew-Butler, specialist midwives who continue to help identify suitable candidates for the study.

Funding statement:

This trial is funded by the National Institute for Health Research (NIHR201944) and sponsored by Manchester University NHS Foundation Trust (MFT). The funders of the study had no role in the study design or writing of the report. Dr Dapre is an NIHR sponsored GP academic clinical fellow.

Competing interests statement.

Michelle Harvie has co-authored three self-help books for the public to follow intermittent diets. All author proceeds are paid directly to the charity Prevent Breast Cancer (registered charity number 1109839) to fund breast cancer research.

For peer review only

Appendix

1.0 Patient Information Sheet (**Supplementary file 1** _ Patient information Sheet MIDDAS GDM v3.0 02032023 clean)

2.0 Consent Form (**Supplementary file 2**_v2Consent Form MIDDASGDM v2.0 19102022 Clean Updated)

3.0 Self-monitoring schedule for capillary glucose and ketone monitoring

ILED		Best NHS Care	
**Ketones (low kcal days)**	**Glucose**	**Ketones (2 days/wk)**	**Glucose**
Fasting before breakfast the morning after each of the low-energy days	Fasting (morning)	Fasting before breakfast on 2 non-consecutive days / week	Fasting (morning)
1 hour post evening meal on each of the low-energy days	1hr post breakfast	1 hour post evening meal on 2 non-consecutive days / week	1hr post breakfast
	1hr post lunch		1hr post lunch
	1hr post dinner		1hr post dinner

4.0 Medical Management Protocols

Hypoglycaemia

Participants will be advised to take 15-20g of rapid acting carbohydrate in the event of hypoglycaemia, (defined as blood glucose <4 mmol/L) which is anticipated to raise blood glucose by 3 mmol/L. Examples of rapid acting carbohydrate include 170-225ml Lucozade Original (not Lucozade Sport), a small carton of fruit juice, 5-6 glucose tablets, 4/5 jelly babies, or a small tin of cola (150-200ml). Participants will be advised to repeat the treatment every 15 minutes until blood glucose is ≥4 mmol/l. The following table highlights when participants should consider taking additional follow-up slower acting carbohydrate:

Situation	Acceptable slow acting carbohydrate
Less than 1 hour before the next meal	Try and avoid
1-2 hour before the next meal	10g
More than 2 hours before the next meal	15-20g

Ketonaemia

Ketone levels ≥1.0 mmol/L on a fasting sample:

- Drink 1L fluids and repeat ketone levels after 4 hours.
- If ketone level has improved (<1.0mmol/L), no further action required.
- If ketone level has increased or remains the same, repeat ketone level after 2 hours.
- If ketone level is persistently increased, consume 40g carbohydrates and repeat in 2 hours.
- Continue to do this until ketone levels <1.0mmol/L.

If a participant experiences >2 episodes of the above throughout the course of the study their notes will be reviewed by the PI and their suitability for remaining in the trial will be assessed.

Guidance for the introduction of diabetes medication (week 24-delivery)

Diabetes medication will be introduced according to the following protocol:

- If ≥25% fasting blood glucose readings are >5 mmol/l and/or ≥25% of 1 hour postprandial glucose readings are >7 mmol/l in a 7 day period: commence Metformin MR 500 mg daily to be increased every 3 days by 500 mg to 1 gram BD if tolerated.

- If after reaching optimal or maximum tolerated dose Metformin ≥25% fasting blood glucose readings are >5 mmol/l in a 7-day period: commence bedtime isophane insulin 4 units and uptitrate the dose by 2 units every 3 days aiming for a fasting glucose of ≤5 mmol/l,

For peer review only

- and/or ≥25% of 1 hour postprandial glucose readings are >7 mmol/l in a 7 day period: commence prandial fast acting insulin analogue (Humalog or Novorapid) 2-4 units with the relevant meal. Uptitrate the dose by 2 units every 3 days aiming for a 1 hour postprandial glucose of ≤7 mmol/l.

- Medication adjustment will be made in accordance with the above guidance.

5.0 Intermittent Low Energy Diet Day Example (**Supplementary file 3** _ Example of 1 days meal plan for 1000kcal with 3 options 12.12.22)

6.0 End of Study Questionnaire (**Supplementary file 4** _ MIDDASGDM End of study Questionniare v1.0 28042022)

LEGENDS

- <image 1>: Figure 1: Inclusion and exclusion criteria
- <image 2>: Figure 2: Participant flow through trial
- <image 3>: Figure 3: Schedule of assessments

For peer review only

Inclusion Criteria

- Pregnant women ≥18 years
- BMI of ≥27.5kg/m2 or a BMI ≥25 kg/m^2 in high risk minority ethnic group (i.e. South Asian, Black African, African Caribbean) and <50 kg/m2 at booking appointment (8-12 weeks' gestation)
- Newly diagnosed GDM according to local diagnostic criteria (fasting glucose ≥5.3mmol/l and/or 2-hour postprandial glucose ≥8.5mmol/l in a 75g OGTT) scheduled to receive first line diet and physical activity (best NHS care)
- 24-30 weeks' pregnant at screening appointment

Exclusion Criteria

- Pregestational type 1 or type 2 diabetes.
- Fasting glucose of ≥7 or 2-hour postprandial of ≥11 on OGTT (immediate intervention with medication would be required in this group of women)
- Current multiple pregnancy
- Maturity Onset Diabetes of the Young (MODY)
- Significant comorbid disease that in PI's opinion would preclude participation in the study e.g. chronic kidney disease, significant cardiac disease, significant history of disordered eating or severe psychological problems.
- Current participation in a GDM medication treatment trial
- People who are not capable of providing informed consent or adhering to the monitoring and safety protocols
- People who have previously had bariatric surgery for weight loss including gastric bypass and sleeve gastrectomy, and/or those prescribed weight loss medications (e.g. orlistat).
- Medications at the time of the OGTT that may interfere with results (e.g. high dose oral steroids, immunosuppressants)
- Previous history of intrauterine growth restriction
- Women who have lost more than 5% of their weight from booking appointment to screening appointment.

Figure 1: Inclusion and exclusion criteria

159x144mm (220 x 220 DPI)

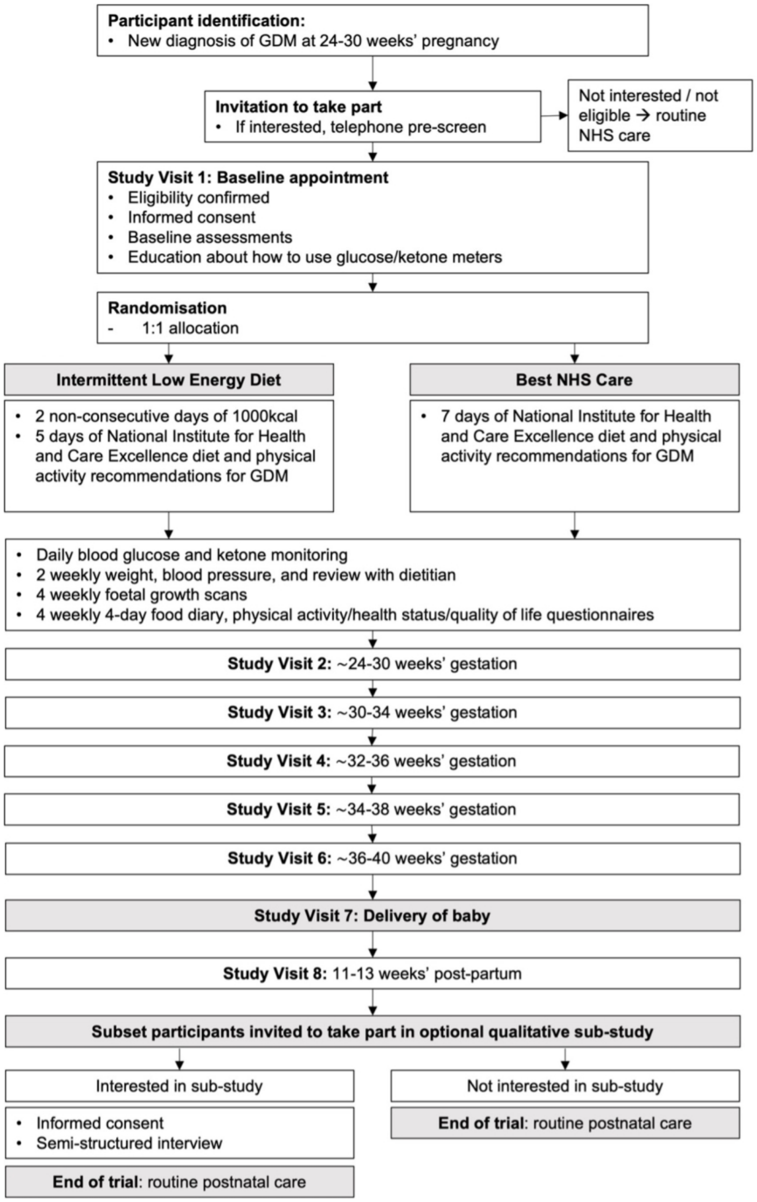

Figure 2: Participant flow through trial

154x245mm (220 x 220 DPI)

	Study Visit							
	1	2	3	4	5	6	7	8
Gestation (weeks)	~24-30	~24-30	~30-34	~32-36	~34-38	~36-40	delivery	11-13 post-partum
Eligibility confirmed	X							
Informed consent	X							
Randomisation	X							
Tailored dietitian review (face to face or remote)	X	X	X	X	X	X		X
Height	X							
Weight^	X	X	X	X	X	X	X	X
Blood Pressure^	X	X	X	X	X	X	X	X
Fasting blood sample*	X				X			X
Questionnairesǁ	X		X		X			X
4-day food diary			X		X			X
Foetal growth scan	X		X		X			
Review of glucose and ketone measurements		X	X	X	X	X		
Neonatal measurementsǂ							X	
Oral glucose tolerance test								X
Exit interview / end of study questionnaires								X
Invitation to optional qualitative sub-study$								X

^Frequency of assessment will be 2-4 weekly depending on whether appointment is face-to-face or virtual due to COVID-19 restrictions
*Fasting bloods: urea and electrolytes, liver function tests, bone profile, lipids, thyroid function tests, HbA1c, beta hydroxybutyrate, free fatty acids, full blood count, fasting glucose, insulin
ǁQuestionnaires: World Health Organisation Quality of Life (brief version), 36-Item Short Form Survey, International Physical Activity Questionnaire (short form), UK Diabetes and Diet Questionnaire
ǂNeonatal measurements include gestational age at delivery, mode of delivery, neonatal weight, cord blood glucose
$Sub-study involves semi-structured interviews exploring participants' thoughts and experiences of the trial

Figure 3: Schedule of assessments

154x236mm (72 x 72 DPI)

NHS
Manchester University
NHS Foundation Trust

MANCHESTER 1824
The University of Manchester

NIHR | National Institute for Health Research

Consultant Endocrinologist – Dr. Basil Issa
Research Dietitian – Dr. Michelle Harvie
Email: mft.middas.gdm@nhs.net
Tel: 07815987910

Nightingale Centre
Manchester University NHS Foundation Trust
Wythenshawe Hospital
Manchester
M23 9LT

MIDDAS-GDM

Manchester Intermittent Diet in Gestational Diabetes Acceptability Study

Participant information sheet

We would like to invite you to take part in a research study **that is testing two different diet programmes which aim to help people with gestational diabetes control their blood sugars.**

If you decide to take part:

- You will be assigned to one of two diet programmes for the duration of your pregnancy. One involves following the standard NHS healthy diet recommendations for pregnancy, and the other follows the standard NHS healthy diet for 5 days/week plus two non-consecutive calorie restricted days of 1,000 kcal per week (both groups will be encouraged to be physically active).

- You will be asked to attend your routine appointments at Wythenshawe or St Marys Hospital and will have fortnightly appointments until delivery of your baby (some appointments may be virtual depending on COVID-19 restrictions). You will be asked to attend the hospital for a blood test 12 weeks after having your baby.

- You will be supported by a diabetes specialist dietitian, midwife, consultant endocrinologist, and your obstetric team throughout the study to help manage your pregnancy and blood glucose safely.

- Throughout the study you will be asked to monitor your food intake via a smartphone/tablet app, or on paper if you prefer, and you will receive feedback on this during your dietary reviews. Comprehensive dietary advice and recipes will be provided.

- Throughout the study you will be asked to monitor your blood sugar using a blood sugar meter four times a day, and you will also be asked to monitor your ketone levels three times on two days of the week (ketones indicate how well your body is using sugar or fat as an energy source). You will be taught how to check your blood sugar and ketone levels.

- If you would like to take part, or you have any questions, then please contact mft.middas.gdm@nhs.net

For peer review only - http://bmjopen.bmj.com/site/about/guidelines.xhtml

Manchester University NHS Foundation Trust

MANCHESTER 1824 The University of Manchester

NIHR | National Institute for Health Research

This study is being carried out by a team of trained dietitians, doctors, nurses, midwives and researchers under the supervision of Dr. Basil Issa and Dr. Michelle Harvie at Wythenshawe and St Marys hospitals (Manchester University NHS Foundation Trust, MFT).

Before you decide if you would like to take part, it is important for you to understand why the research is being done and what taking part would involve for you. Please take your time to read the following information carefully. Discuss it with your friends, relatives, or GP if you wish to. Take time to consider whether or not you wish to take part.

Please ring the research team at the number at the top of the first page, or e-mail mft.middas.gdm@nhs.net if there is anything that is not clear, or if you would like more information. You can attend an information session about the diets and the study before agreeing to take part if you would like to.

Your participation in the study is entirely voluntary; you do not have to take part if you do not want to and you can opt out of the study at any time without giving a reason. Thank you for reading this information. We hope this research will be of interest to you.

Why are we doing this research?

Around 1 in 8 pregnant women can develop gestational diabetes. This condition causes risks to mother and baby from high blood sugar, high blood pressure, induced labours, caesarean-sections, and larger babies. Women often need medication to control blood sugar despite following recommended NHS healthy eating plans for pregnancy. Intermittent low-calorie diets (two non-consecutive days over the course of the week) improve blood sugar control and reduce the need for medication in patients with type 2 diabetes. We want to find out whether intermittent low-calorie diets might also improve blood sugar control in gestational diabetes and reduce the need for medication as it is a similar condition to type 2 diabetes.

What is the purpose of this research?

This study aims to assess the acceptability (to you) and safety of an intermittent low-calorie diet compared to the usual recommended NHS healthy eating and lifestyle plan for gestational diabetes. **A computer system will randomly allocate you to one of the two diets**. We want to find out which diet is most acceptable to women, whether there is any difference in the two diets' effect on blood sugar control, and any side effects experienced by women. The findings of this study will inform a larger study which will be designed to more closely compare the effect of the two diets on blood sugar control in women with gestational diabetes.

Why have I been asked to take part?

You have been invited to take part in this study because you have been diagnosed with gestational diabetes. We hope to recruit around 48 people to take part in this study.

For peer review only - http://bmjopen.bmj.com/site/about/guidelines.xhtml

Manchester University NHS Foundation Trust
MANCHESTER 1824 The University of Manchester
NIHR | National Institute for Health Research

What happens if you agree to take part?

If you agree to take part you will be randomly allocated via a computer system to one of two diet and lifestyle programmes for the remaining weeks of your pregnancy.

Best NHS Care Healthy Diet programme

You will receive personalised advice from a specialist dietician. Recommendations will include increased fruit/vegetable intake, low glycaemic index starchy foods (i.e starchy foods which are slowly absorbed and take a while to raise your blood sugar level), reducing refined sugar, and having regular mealtimes. You will be advised how to design your diet to include the right amount of protein, fats, carbohydrates, and fibre, and will be given meal plans and recipes. You will also be advised to try to complete 150 minutes of moderate intensity exercise a week in addition to strength exercises on at least two days of the week.

Intermittent Low-Calorie Diet programme

If you are allocated to this group you will receive personalised advice to follow a low-calorie diet of 1,000 kcal on two non-consecutive days of the week and the NHS healthy diet on the other five days of the week. The 1,000 kcal days include a set number of portions of protein, carbohydrates and fat foods, fruits, vegetables and dairy/dairy alternatives typically including ~210g (7 oz) of lean protein foods and 3-4 portions of wholegrain carbohydrates, 5 portions of vegetables, 2 of fruit, and 3 of dairy or dairy alternatives and a small amount of healthy fat. You will also be advised to try to complete 150 minutes of moderate intensity exercise a week in addition to strength exercises on at least two days of the week.

Monitoring

You will have all of your usual routine antenatal appointments including checks on your weight, blood pressure, blood tests and ultrasound scans. Extra blood tests will be done as part of the study and these will be added on to samples taken during your routine blood tests.

You will be asked to monitor your blood sugar at home four times a day until your baby is born, and your ketone levels on two days of the week (you will be taught how to do this using a finger prick machine). The results will be recorded when you attend clinic.

We would like to collect a sample of blood from the umbilical cord at delivery to check blood sugar and insulin levels, and your baby's birthweight will be documented. Guidelines currently recommend that wherever possible the umbilical cord is clamped after 1-2 minutes; this is referred to as 'delayed cord clamping' and means that babies receive as much blood as possible from the placenta. This is normal practice. The cord blood sample will not affect your ability to have delayed cord clamping and will not cause any harm to your baby. It will not be possible, however, to delay clamping of the cord for so long that all blood has left the cord (the sample needs to be taken whilst there is still some blood left in the cord).

When babies are born to mothers with gestational diabetes it is normal that their birth weight is recorded and that their blood sugar is monitored for 12 hours following delivery; these results will be recorded by the research team.

For peer review only - http://bmjopen.bmj.com/site/about/guidelines.xhtml

NHS Manchester University NHS Foundation Trust

MANCHESTER 1824 The University of Manchester

NIHR | National Institute for Health Research

You will be asked to attend an additional glucose tolerance test at the hospital 12 weeks after delivery to assess whether you have any residual diabetes (95% of women do not) and also to assess how sensitive your body is to insulin (an important risk factor for the development of diabetes in the future). You will be asked to attend at 9:00am having fasted (no food or drink apart from water) from midnight. A blood sample (around 10 mL/2 teaspoons) will be taken for glucose and insulin and you will be asked to drink a sugary drink with 75 grams of glucose. A further blood sample will be taken after 2 hours for glucose and insulin. You will need to remain in hospital during this time. The reason for this is to help us to understand whether there could be any difference in the body's ability to process sugar between the two diet groups, and also to find out whether any women still have signs of diabetes after pregnancy.

Any blood samples taken as part of the study will be identifiable only using your study identification number and will have none of your personal details. Part of the sample will be sent for immediate analysis and any remaining will be stored securely, accessible only by the research team. At the end of the study any left over samples will be disposed of in accordance with the Human Tissue Act (2004).

You will be asked to record your food intake via the Libro smartphone/tablet app or in a paper diary for four days during four weeks throughout the study. You will also be asked to complete three questionnaires to assess your wellbeing and level of physical activity in these weeks, and a final end of study questionnaire at the final appointment.

Ongoing support from a specialist team of healthcare professionals
Your specialist team includes a Consultant Endocrinologist, Consultant Obstetrician, diabetes specialist dietitian, midwives, and a GP trainee with a special interest in women's health. The specialist team work closely with the usual obstetric teams involved in your care. Reviews with the team will be either face to face when you attend clinic or remotely using video calls.

Mobile Applications and Glucose Meters
The study will use a smartphone application called 'Libro' to help you record information. Libro is a smartphone application which allows you to record your dietary intake during the study. We will ask you to record 4 days of food and drink intake during 4 weeks across the study. Your diaries will be viewed by your allocated dietitian who will provide personalised dietary feedback via the app. You are also free to record more days of your diet should you wish, which some people find helpful. If you do not want to use the mobile app you can use paper instead. You will be supported to set up and use the Nutritics Libro App at your appointments. You do not have to use the application to be part of the study.

Your blood sugar will be monitored using a glucose monitoring device which checks your blood sugar using a 'fingerprick' blood test. You will be shown how to do this yourself. With your permission the research team will make a note of your glucose readings at every visit, either by checking your glucose monitoring device, or by uploading your glucose meter readings onto the computer if you are using a mobile application.

The self-monitoring schedule is as follows:

For peer review only - http://bmjopen.bmj.com/site/about/guidelines.xhtml

NHS Manchester University NHS Foundation Trust

MANCHESTER 1824 The University of Manchester

NIHR | National Institute for Health Research

Intermittent low-energy diet monitoring		Best NHS care monitoring	
Ketones (low kcal days)	Glucose	Ketones (2 days/wk)	Glucose
Fasting before breakfast the morning after each of the low calorie days	Fasting (morning)	Fasting before breakfast on 2 non-consecutive days / week	Fasting (morning)
1 hour post evening meal on each of the low calorie days	1hr post breakfast	1 hour post evening meal on 2 non consecutive days / week	1hr post breakfast
	1hr post lunch		1hr post lunch
	1hr post dinner		1hr post dinner

What should I do if my blood glucose or ketones are out of range?

Low Blood Sugar

You are advised to take 15-20g of 'rapid acting' carbohydrate if your blood glucose is <4 mmol/L). Examples include 170-225ml Lucozade Original (not Lucozade Sport), a small carton of fruit juice, 5-6 glucose tablets, 4/5 jelly babies, or a small tin of cola (150-200ml). You will need to repeat the treatment every 15 minutes until your blood glucose is ≥4 mmol/l.

The following table highlights when you need to consider an additional slower acting carbohydrate:

Situation	Acceptable slow acting carbohydrate
Less than 1 hour before the next meal	Try and avoid
1-2 hour before the next meal	10g (eg half of one of the items below)
More than 2 hours before the next meal	15-20g (eg slice of toast, piece of fruit, small bowl of cereal, glass of milk)

Raised Ketones

If your ketone levels are ⩾1.0 mmol/L on a fasting sample:

- Drink 1L fluids and repeat ketone levels after 4 hours.
- If your ketone level has improved (<1.0mmol/L), no further action is required.
- If your ketone level has increased or remains the same, repeat your ketone level after 2 hours.
- If your ketone level is persistently increased, consume 40g carbohydrates (eg one bagel, bowl of cereal and a banana, small jacket potato), and repeat in 2 hours.
- Continue to do this until your ketone levels are <1.0mmol/L.

Make Immediate Contact with the research team if:

Blood glucose	• Your blood glucose is <3.0 mmol/l or you have symptoms requiring medical attention which are thought to be due to low blood glucose,

For peer review only - http://bmjopen.bmj.com/site/about/guidelines.xhtml

NHS
Manchester University
NHS Foundation Trust

MANCHESTER 1824
The University of Manchester

NIHR | National Institute for Health Research

	• Your fasting blood glucose is >5.2 mmol/L on more than a quarter of your measurements on two days in a row,<br>• Your 1 hour post-meal blood glucose is >7.7 mmol/L on more than a quarter of your measurements on two days in a row
Ketones	• Your blood ketones are >1.0 mmol/L

Will I need medications?
If your blood sugars are found to be high despite following the recommended diet and lifestyle programmes you may be advised to start medication to help control your blood sugar. You will be advised on changes to your medications by a diabetes specialist nurse/diabetes midwife and also a Consultant Endocrinologist if required. This is usual practice regardless of whether you are taking part in the study.

What care will I receive after the study has stopped?
At the end of the study, you will be provided appropriate ongoing dietary advice from the study dietitian following your final glucose tolerance test to follow the NHS healthy eating and lifestyle plan. You will receive routine postnatal care from your GP, hospital team, and dietitian if required. You will be advised to see your GP for an annual blood test to check your blood sugar levels (this is routine care for women with gestational diabetes). Approximately 5% of women with gestational diabetes have residual diabetes after delivery. This will be identified from your glucose tolerance test/HbA1c; if this is the case you and your GP will be informed. Your GP will take over the management of your diabetes as per routine care outside the study.

Interview sub study
Women in this study may be invited to take part in an interview at the end of the study . You will be asked about your views and experiences on trying to follow your allocated diet programme. This interview can be arranged at a time that suits you, either at Wythenshawe or St Marys Hospital, at your home, or over the telephone. There is no obligation to take part in this interview study.

<u>Frequently asked questions</u>

Do I have to take part?
No, you do not have to take part if you do not wish to and your decision will not affect any standard of care you receive at Wythenshawe or St Marys hospitals (Manchester University NHS Foundation Trust, MFT).

What happens if I change my mind?
It is OK if you agree to take part in the study but later change your mind. You do not need to give a reason and it will not affect the standard of care you receive. The study team may also choose to withdraw you if it is necessary for your health or safety due to unexpected findings during the study. If you decide to withdraw from the study, or the study is stopped for any reason, you will be asked whether or not you are happy for us to keep the data that may have already been collected. If you do withdraw from the study you will continue to be cared for by your usual specialist diabetes and obstetric teams for the duration of your pregnancy. You will

For peer review only - http://bmjopen.bmj.com/site/about/guidelines.xhtml

still have the option of completing the end of study questionnaire and/or interview to provide feedback; this is very useful for the research team to help us understand potential reasons you may have chosen to withdraw from the study.

You will also have the option that if you withdraw, researchers may still collect relevant information about your pregnancy and/or gestational diabetes from your medical records within the 18-month study duration. This will be an option on the consent form.

Are there any benefits from taking part?
You will receive frequent personalised advice and support to follow two diet and lifestyle programmes which may help to control your blood sugar levels throughout your pregnancy. The information gained from this study will also help inform the future NHS care of patients with gestational diabetes.

Are there any risks from taking part?
Research has found that diets consisting of two low-calorie days a week are very low risk. Pregnant women will develop slightly higher levels of ketones when following low calorie diets than women who are not pregnant. Ketones are produced naturally by the body when the body uses fat stores for energy (i.e. when we follow a low calorie diet or haven't eaten enough because we are ill).

Some research suggests that very high levels of ketones throughout pregnancy may cause a higher risk of babies being slightly smaller than average. It is very unlikely that you will develop high levels of ketones by following this diet. You will be provided with a ketone meter and you will be asked to check your ketone levels before your lunch and evening meal on your low-calorie days, and the following morning, to make sure that your ketone levels are normal.

On your low-calorie days you may feel slightly more hungry, or you may experience other effects such as increased nausea, light headedness, or tiredness. It is important that you eat regularly throughout the day to reduce the risk of this happening. You will be asked to report any side effects of following the diet to the team at each appointment.

What happens if my baby or I become unwell during the study?
The safety of you and your baby are of utmost importance and remain our priority. In the instance that either of you become unwell your case will be reviewed by our specialist team and your suitability for continuing in the trial will be decided. Although it remains exceptionally rare, were you to experience the unexpected loss of your baby you will be withdrawn from the trial and supported by the dedicated specialist bereavement team at the hospital. Any information which has been collected as part of the trial will be stored securely and once we have finished the study we will keep some of the data so we can check the results. We will write our reports in a way that no-one can work out that you took part in the study.

What will happen to blood samples which are taken?
Some blood samples taken as part of the study will be sent to the laboratory immediately for analysis and any remaining will be stored securely for the duration of the study. Only your 'study ID' will be used – the samples will have none of your personal details on them. At the end of

For peer review only - http://bmjopen.bmj.com/site/about/guidelines.xhtml

the study any remaining samples will be disposed of in accordance with the Human Tissue Act (2004).

What happens if something goes wrong?
If you have a concern about any aspect of this study, you should ask to speak with the lead researchers who will do their best to answer your questions (**Dr. Basil Issa** or **Dr. Michelle Harvie – via the study office – via michelle.harvie@manchester.ac.uk or telephone 0161 291 4410**). If you remain unhappy and wish to complain formally, you can do so through the NHS complaints procedure. Details can be obtained from the NHS Patient and Liaison Service (PALS) on **Tel:** 0161 276 8686 or contact the team by email pals@mft.nhs.uk.

The hospital is insured to carry out clinical research through the NHS Indemnity scheme. If something did go wrong and you were harmed or suffered deterioration in your health as a result of taking part in this study then you may have grounds for legal action or compensation.

Additional information about the study

Will my lifestyle be affected if I take part?
An essential aspect of this study is a change to your diet and physical activity patterns with support from a specialist team of healthcare professionals.

Payments
We are able to offer free parking at Wythenshawe/St Marys Hospitals for study visits and offer reimbursement for reasonable travel expenses (car, bus or tram) linked to visits for this study. There are no other payments for taking part.

Will my details be kept confidential?
Yes. The study team and any associated regulatory authorities follow strict ethical and legal guidance regarding participant confidentiality. Any information we have about you will be handled in confidence and will only be used for the purposes of this study. All data recorded will be coded and your name will remain anonymous.

During the study we will inform your GP via letter of your participation in the study and your ongoing results, including your weight, blood tests, any abnormal findings and any recommendations for treatment.

If you join the study, some relevant parts of your medical records may be looked at by authorised personnel at Wythenshawe or St Marys hospitals prior to starting the study. These records may also be looked at by an independent auditing body and regulatory authorities to check that the study is being carried out correctly. We will only access parts of your medical records that are relevant to this research and all information accessed will be kept strictly confidential.

How will we use information about you?

For peer review only - http://bmjopen.bmj.com/site/about/guidelines.xhtml

We will need to use information from you and from your medical records for this research project.

This information will include the following:

- Initials
- NHS number
- Name
- Contact details
- Medical History including test results

People will use this information to do the research or to check your records to make sure that the research is being done properly. People who do not need to know who you are will not be able to see your name or contact details. Your data will have a code number instead. We will keep all information about you safe and secure.

Once we have finished the study, we will keep some of the data so we can check the results. We will write our reports in a way that no-one can work out that you took part in the study. Other researchers from outside the Trust may ask to see this data for the purposes of furthering their research. We will only share this upon written request to the Trust. The external researchers will be asked to sign a Confidentiality Agreement before any data is shared.

What are your choices about how your information is used?
You can stop being part of the study at any time, without giving a reason, but we will keep information about you that we already have. If you choose to stop taking part in the study, we would like to continue collecting information about your health during pregnancy from your hospital records. If you do not want this to happen, tell us and we will stop. We need to manage your records in specific ways for the research to be reliable. This means that we won't be able to let you see or change the data we hold about you.

Where can you find out more about how your information is used?
You can find out more about how we use your information

- at https://research.cmft.nhs.uk/getting-involved/gdpr-and-research
- by asking one of the research team
- by sending an email to mft.middas.gdm@nhs.net or
- by ringing us on 07815987910

How will my details be used to access the Mobile Applications?
None of your personal details (other than the telephone number from which an application is downloaded) will be needed to access the mobile applications. Once you have given your consent to take part in the study you will be issued with a 'dummy' e-mail and password under a pseudonym (fake name). Only the research team will know the dummy e-mail address you have been assigned to, in order to be able to review your data. The application will not contain your identifiable data. If you choose to use a mobile application to monitor your blood sugar

For peer review only - http://bmjopen.bmj.com/site/about/guidelines.xhtml

levels the relevant terms of service for the app and the app developers privacy policy will apply. It will be your responsibility to read and understand these prior to download.

Will my insurance be affected if I take part in this study?

It is unlikely that your insurance premiums will be affected by participation in this study as the study has the potential to improve your diabetic control and reduce your risk of ill health. However, if you are at all concerned, then we advise that you contact your insurers and seek expert advice before agreeing to participate.

Who has reviewed this study?

Research in the NHS is looked at by an independent group of people called a Research Ethics Committee (REC). The REC is made up of experts, non-experts and members of the general public. Together they review research applications to ensure your safety, rights, wellbeing and dignity are protected at all times. This study has been reviewed and given favourable opinion by REC.

What will happen to the study results?

It is intended that the results of this study will be presented at conferences and published in medical journals so that we can explain to the medical community what our research results have shown. To do this our study information is double-checked by other professionals in research and healthcare. There is a possibility that the study and its results may be publicised for example on radio, television, magazines, books and websites. **You will not be identified in any publicity, reports or publication arising from this study.** If you would like a general summary of the results of the study you can select this on the consent form or please contact the research team.

Who is organising and funding the research?

Researchers from Wythenshawe hospital, have designed this study and will be carrying out this research. This study has been funded by the National Institute of Health and Research.

<u>Further information and contact details</u>

For further information about this study, please contact **mft.middas.gdm@nhs.net or 07815987910**

Thank you for taking the time to read this information sheet.
We hope it has been of interest to you.

For peer review only - http://bmjopen.bmj.com/site/about/guidelines.xhtml

Manchester University NHS Foundation Trust

MANCHESTER 1824 The University of Manchester

NIHR | National Institute for Health Research

Participant pathway for the study

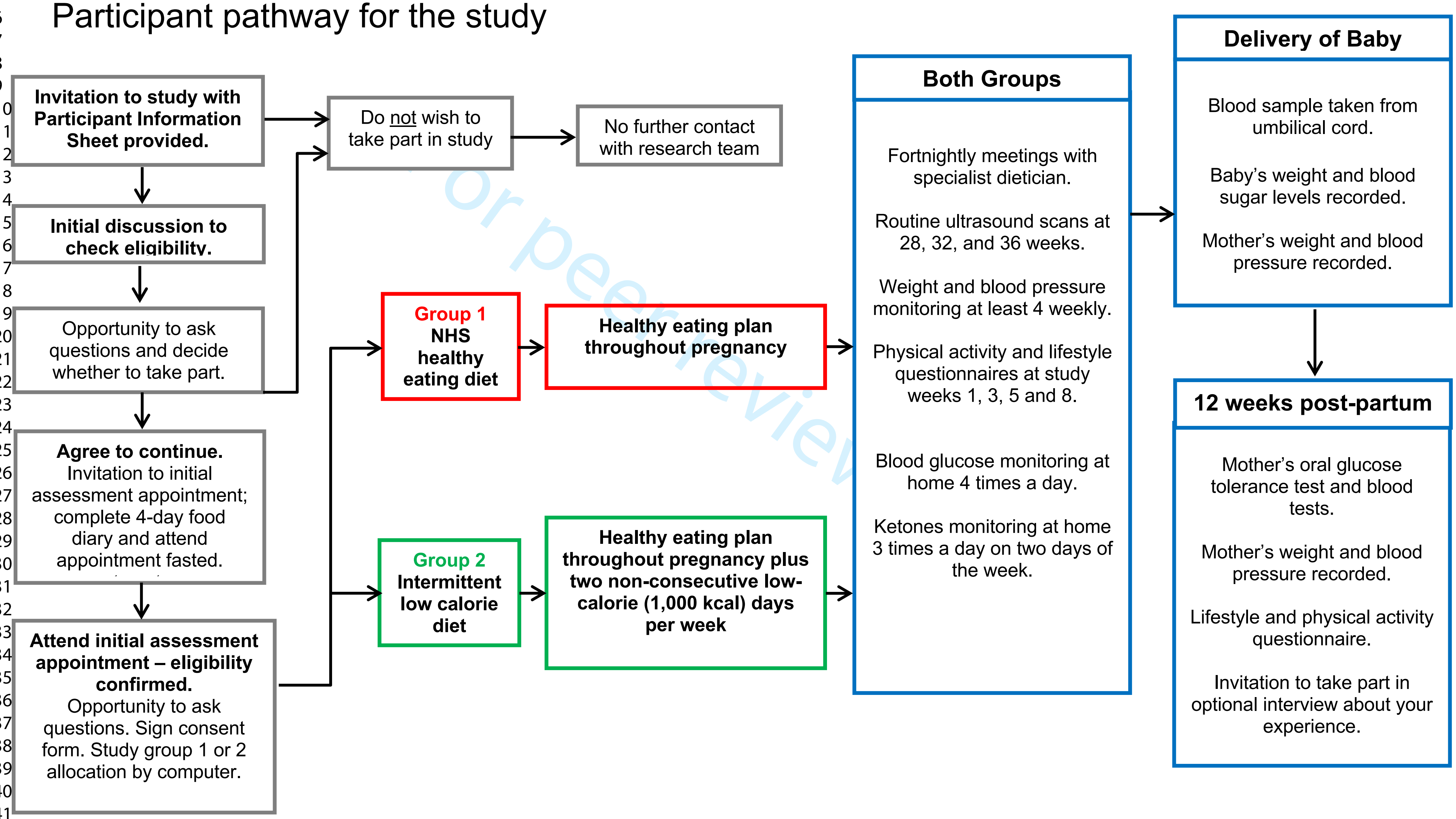

For peer review only - http://bmjopen.bmj.com/site/about/guidelines.xhtml

Manchester University NHS Foundation Trust

Consultant Endocrinologist – Dr. Basil Issa
Tel: 0161 291 7070
Research Dietitian – Dr. Michelle Harvie
Tel: 07815987910
Email: mft.middas.gdm@nhs.net

Nightingale Centre
Manchester University NHS Foundation Trust
Wythenshawe Hospital
Manchester
M23 9LT

MIDDAS-GDM

Manchester Intermittent Diet in Gestational Diabetes Acceptability Study

Participant Informed Consent Form

Participant Identification Number:

Please **initial** box

1. I confirm that I have read and understand the participant information sheet (version 3.0) for the above study. I have had the opportunity to consider the information and ask questions, and have had these answered satisfactorily. ☐

2. I understand that my participation is voluntary and that I am free to withdraw at any time, without giving any reason, without my medical care or legal rights being affected. ☐

3. I understand that relevant sections of my medical notes and data collected during the study may be looked at by individuals from Manchester University NHS Foundation Trust and regulatory authorities, where it is relevant to my taking part in the research. I give permission for these individuals to have access to my records. ☐

4. I consent to the collection of blood samples to be collected as described in the participant information sheet. ☐

5. I consent to the collection of cord blood to be collected at the time of delivery as described in the participant information sheet. ☐

6. I understand that the information collected about me will be used to support other research in the future and may be shared anonymously with other researchers. ☐

7. I agree that my blood sugar readings can be recorded by the study team. ☐

8. I agree to my GP being informed of my participation in this study and changes to my weight, body measurements, blood results, questionnaire results and medications as required. ☐

For peer review only - http://bmjopen.bmj.com/site/about/guidelines.xhtml

Manchester University NHS Foundation Trust
The University of Manchester
NIHR | National Institute for Health Research

9. I understand that to use the mobile applications as described in the Patient Information Sheet, I will need to provide my personal mobile number to be able to access the app. ☐

10. I understand that the information I provide to mobile applications as described in the Patient Information Sheet will be treated in line with the relevant terms of service and the app developers privacy policy at the time of downloading the application. ☐

11. I have informed the study team of any health issues, including those which may affect my ability to follow the diet, and I will inform the study team of any unusual symptoms that occur during the diet. I will inform the study team of changes to my health status during the study. ☐

12. I have informed the study team of any health issues, including those which may affect my ability to exercise, and I will inform the study team of changes to my health status during the study. ☐

13. I consent to the storage of personal information (including electronic) for the purposes of this study. I understand that any information that could identify me will be kept strictly confidential and that no personal information will be included in the study report or other publication. ☐

14. I agree to take part in the above study. ☐

15. I agree that relevant information about my pregnancy and/or gestational diabetes can be obtained from my medical records within the 18-month study duration if I withdraw from the study early. ☐

16. I am aware that my non-identifiable trial data may be shared with other researchers for the purposes of research. ☐

Optional (delete as appropriate)

17. I agree to be approached to take part in sub-study 1 (interview study), and understand that I will be approached to take part in the sub-study regardless of whether I withdraw from the main study — YES/NO

18. I would like to receive a summary of the final study results — YES/NO

19. I agree to be contacted regarding future research opportunities — YES/NO

My preferred contact (*please tick and include email if preferred*)

Do not contact ☐ Post ☐ Email ☐ ______________________

For peer review only - http://bmjopen.bmj.com/site/about/guidelines.xhtml

Manchester University
NHS Foundation Trust

MANCHESTER 1824
The University of Manchester

NIHR | National Institute for Health Research

Signatures

………………………..………....	…………………...	……………..……………
Name of participant	Date	Signature
…………………………………....	……………………	……………..……………
Name of person taking consent	Date	Signature

When completed: 1 for participant; 1 for patient file; 1 for medical notes; 1 for GP; 1 (original) for site file

For peer review only

For peer review only - http://bmjopen.bmj.com/site/about/guidelines.xhtml

Example of 1 day meal plan for Diet Day

The diet days aim to limit the calories to 1000 calories per day. You are aiming to include 2 (not consecutive) diet days each week. The other 5 days, follow the Mediterranean diet as described earlier. To keep the calories to 1000, the diet day will look like this:

**Mixed diet**		**Vegetarian/ vegan diet**
**4**	Carbohydrate portions	3
**6**	Protein portions	7
**5**	Vegetable portions	5
**2**	Fruit portions	2
**3**	Dairy portions	3
**1**	Fat portions	1

Below are some examples of meals that can be used to help you follow a 1000 calorie diet.. There are options for a mixed diet or vegan or vegetarian options, if you feel you wanted to try meat free days. Filling up on vegetables will make you feel less hungry

For peer review only

Mixed diet options

Breakfast	Portion	Dairy	Protein	Carb	Veg	Fruit	Fat
Grilled lean bacon	1 rasher	0	1	0	0	0	0
Grilled tomatoes	7 cherry tomatoes	0	0	0	1	0	0
Tea/ coffee	1 mug	0	0	0	0	0	0
Midmorning							
Diet or natural yogurt	1 small carton	1	0	0	0	0	0
Lunch							
Wholegrain bread	2 medium slices	0	0	2	0	0	0
Tuna	⅓ of a 120g can	0	1	0	0	0	0
Green salad	Cereal bowl full / 80 g with oil-free dressing	0	0	0	1	0	0
Satsumas	2	0	0	0	0	1	0
Mid afternoon							
Low fat cheese	30g / match box size	1	0	0	0	0	0
Apple slices	I medium apple (80g)	0	0	0	0	1	0
Tea/ coffee		0	0	0	0	0	0
Evening							
Vegetable rice	4 tablespoons cooked rice 160g of mix vegetables	0	0	2	2	0	0
Chicken curry	90g /average chicken breast (no skin) & ½ can tomatoes, 1 desertspoon oil	0	3	0	1	0	1
Bedtime							
Low fat houmous	1 level tablespoon	0	1	0	0	0	0
Pepper sticks	½ red pepper	0	0	0	1	0	0
Milk	1 small glass	1	0	0	0	0	0
**Total portions a day**		**3 portions**	**6 portions**	**4 portions**	**5 portions**	**2 portions**	**1 portion**

For peer review only

Vegetarian option

Breakfast		Dairy	Protein	Carb	Veg	Fruit	Fat
Egg	2 poached	0	2	0	0	0	0
Mushrooms	2 cupped handfuls / 80g	0	0	0	1	0	0
Tea/ coffee	1 mug	0	0	0	0	0	0
Midmorning							
Cheddar cheese	1 match box size / 30g	1	0	0	0	0	0
Cucumber	Sliced handful	0	0	0	1	0	0
Lunch							
Baked beans	2 tablespoons	0	1	0	0	0	0
Seeded bread toasted	1 medium sliced	0	0	1	0	0	0
Blueberries	1 handful	0	0	0	0	1	0
Mid afternoon							
Meat free ham	2 slice small	0	1	0	0	0	0
Pepper	½ sliced	0	0	0	1	0	0
Avocado	¼	0	0	0	0	0	1
Tea/ coffee	1 mug	0	0	0	0	0	0
Evening							
Vegetarian sausage casserole Jacket potato (100g)	1 grilled sausage 2 cereal bowls vegetables 1 ½ egg sized (100 g)	0	2	1	2	0	0
Bedtime							
Pear	1 medium	0	0	0	0	1	0
Low fat cream cheese	1 tablespoon	1	0	0	0	0	0
Whole wheat cracker	2 biscuits	0	0	1	0	0	0
Milk	1 small glass	1	1	0	0	0	0
**Total portions a day**		**3 portions**	**7 portions**	**3 portions**	**5 portions**	**2 portions**	**1 portions**

For peer review only

Vegan options

Breakfast		Dairy equivalent	Protein	Carb	Veg	Fruit	Fat
Branflakes	3 tablespoons	0	0	1	0	0	0
Milk- soya	200 ml	1	0	0	0	0	0
Tea/ coffee	1 mug	0	0	0	0	0	0
Midmorning							
Soya yogurt	3 tablespoons	1	0	0	0	0	0
Lunch							
Kidney bean & Vegetable chilli<br><br>Wholemeal pitta	3 tablespoons of beans 60g with 1 cereal bowl mixed vegetables & 1/2 can chopped tomatoes<br>½ pitta	0	2	1	2	0	0
Banana	1 medium	0	0	0	0	1	0
Mid afternoon							
Low fat hummus	2 level tablespoon	0	2	0	0	0	0
1 carrot	I medium carrot (80g)	0	0	0	1	0	0
Tea/ coffee	1 mug	0	0	0	0	0	0
Evening							
Quinoa	2 tablespoon cooked	0	0	1	0	0	0
Tofu	4 matchbox	0	2	0	0	0	0
Mixed salad with edamame beans	2 x Cereal bowl full with oil free dressing & 1 tablespoons of edamame	0	1	0	2	0	0
Bedtime							
Peanut butter	1 heaped teaspoon	0	0	0	0	0	1
Apple	1 medium sliced	0	0	0	0	1	0
Milk	1 small glass	1	0	0	0	0	0
**Total portions a day**		**3 portions**	**7 portions**	**2 portions**	**5 portions**	**2 portions**	**1 portions**

For peer review only

To help with estimation of portions the following tables outline weight and measures of the different food groups. Where possible household measures are given to make things a little easier. Use these to help you plan your 2 days in the week of 1000 calories.

**Carbohydrate**<br>**4 portions - mixed diet**<br>**3 portions - vegan/vegetarian**	**Equal to**
Wholewheat or oat breakfast cereal, e.g. wholewheat biscuit, malted wholewheat squares, Grapenuts, bran flakes, fruit & fibre	24g or 3 tablespoons or<br>1 whole wheat biscuit
Porridge oats or no-added sugar muesli	20g or 1 heaped tablespoon
Wholegrain, wholemeal, rye, granary bread	36g or medium slice of bread (other than rye), 1½ slices of rye, or ½ roll
Wholemeal or multigrain pitta bread or tortilla wrap, chapatti made without fat	60g or 1x 8” tortilla<br>or 1 standard pitta<br>or small thin chapatti
Rye crispbread, crackers, oak cakes	22g or 2 crispbreads/ 2 oatcakes
Wholegrain rice cake	16g or 2 rice cakes
Wholewheat pasta or rice - cooked amount<br><br>Cous cous, Bulgar wheat, Quinoa, Pearl barley	1 tablespoon uncooked 2 tablespoons cooked<br><br>30g- raw weight or 60g cooked
Lasagne (wholemeal if possible)	20g raw weight or<br>1 large sheet or 1½ smaller sheets
Noodles (wholemeal if possible)	25g raw weight or ½ block/nest
Baked or boiled potato (in skin), cassava, sweet potato	1½ egg sized potatoes or<br>100g raw weight
Wholemeal pizza base (topping is from other food groups)	35g or $^{1}/_{6}$ of thin 10” pizza base
Unsweetened popcorn	20g or 2 handfuls

For peer review only

**Protein**<br>**6 portions – mixed diet**<br>**7 portions – vegan/vegetarian**	**Equal to**
Fresh or smoked white fish (e.g. haddock or cod)	60g or 2oz<br>2 fish finger size
Seafood, e.g. prawns, mussels, crab	45g or 1½oz
Canned tuna or salmon in brine or spring water	45g or 1½oz<br>⅓ standard tin (120g)
Oily fish (fresh or tinned in tomato sauce or olive oil - drained), e.g. mackerel, sardines, salmon, fresh tuna, kippers, smoked salmon or trout	30g or 1oz or<br>¼ standard tin (120g) or<br>¼ fillet of salmon
Chicken, turkey, duck, pheasant (cooked without skin)<br>Lean beef, pork, lamb, rabbit, venison, offal (fat removed)<br>**Quorn fillets, steak, mince or pieces**<br>**Vegetarian mince frozen**	30g or 1oz or<br>1 slice size of playing card
Lean grilled bacon<br>**Quorn ham**	25g or ¾oz or<br>1 rasher
Lean ham<br>**Quorn bacon rashers (not slices)**	30g or 1oz or<br>1 medium, 2 small or 4 wafer thin slices
Eggs	60 g or 2 oz or<br>1 egg
Tofu	50g or $1^{2}/_{3}$ oz or<br>Size of 2 match boxes
Tempeh	25 g or 1 oz or<br>Size of 1 match box
Baked beans (reduced sugar)	60 g or 2 oz or<br>2 tablespoons
Lentils, chickpeas and kidney beans, mung beans, black eye beans, puy lentils, toor dahl, urad dahl,<br>Raw weight<br><br>Cooked or tinned weight	20g or ⅔ oz or<br>1 tablespoon raw<br><br>65g or 2oz or<br>1½ tablespoons cooked /tinned or 1 cupped handful
Soya beans (frozen or cooked) or edamame beans	30g or 1oz or<br>1 tablespoon
Vegetarian sausage	25g or ¾ oz or ½ sausage
Textured vegetable protein (TVP)	10g or ⅓ oz uncooked or<br>1 heaped tablespoon uncooked
Low fat hummus	30g or 1oz or<br>1 level tablespoon

For peer review only - http://bmjopen.bmj.com/site/about/guidelines.xhtml

Vegetables – min 5 portions<br>1 portion = 80g or 2⅔oz	1 portion is equal to
Asparagus, Aubergines, Broccoli, Brussel sprouts, Carrots, Cabbage, Cauliflower, Chinese leaves, Courgettes, Cucumber, Curly kale, Green beans, Lettuce (mixed leaves), Mange tout, Methi, Mushrooms, Okra, Pak choi, Peas, Sugar snap, Spinach, Spring greens cooked, Sweetcorn, Tomatoes, Watercress fresh	80g or 2 ⅔ oz or 2 spears of broccoli, 8 cauliflower florets. 3 heaped tablespoons of vegetables or large cereal bowl of salad.

Fruit - 2 portions.<br>1 portion = 80g or 2⅔oz (30g or 1oz dried fruits)	1 portion is equal to
Berries (e.g. blackberries, blueberries, redcurrants, raspberries, strawberries)<br>Cherries or grapes	80g or 2⅔oz<br>1 handful
Grapefruit, guava and mango	80g or 2⅔oz or<br>½ a whole fruit
Large fruit (e.g. melon, pineapple, papaya)	80g or 2⅔oz or<br>1 medium slice
Medium fruits (e.g. apple, pear, nectarine, orange, peach, banana, pomegranate)	80g or 2⅔oz<br>1 fruit
Small fruit (e.g. fresh apricots, kiwi, clementine, passion fruit, plums)	80g or 2⅔oz or<br>2 fruits
Any stewed fruit—unsweetened or with calorie-free sweetener e.g. apple, rhubarb	80g or 2⅔oz or<br>3 tablespoons
Kumquats, lychees, physalis	5 fruits
Dried fruits (raisins, currants, apricots)	30g or 1oz or<br>1 tablespoon

Milk and dairy foods - 3 portions	Equal to
Milk (semi-skimmed or skimmed)<br>Alternative 'milks' with added calcium, e.g. soya **	⅓ pint or 200ml or<br>1 small glass
Diet yoghurts, Low fat/fat-free Greek or Greek Style or natural yoghurts, fromage frais or plain soya yoghurt, high protein yogurt	120-150g or 4-5oz or<br>1 small pot or<br>3 tablespoons
Whole milk natural yogurt	80g or 1 ⅔ oz or<br>2 tablespoons
Cottage cheese	75g or 1½oz or<br>¼ pot, 2 tablespoons
Cream cheese (light or extra light)	30g or 1oz or<br>1 tablespoon
Lower fat hard cheeses e.g.:<br>Reduced fat cheddar, Edam,<br>Bavarian smoked, feta, ricotta, mozzarella, reduced fat halloumi, paneer made from semi-skimmed milk	30g or 1oz or Matchbox size<br>No more than 180g or 6oz a week

** we recommend soya milk as coconut, oat and almond milks are lower in protein and calcium

For peer review only

Fat – 2 portions only	Equal to
Margarine or low-fat spread (avoid the buttery types)<br>Olive oil or other oil<br>Oil based dressing<br>Pesto<br>Mayonnaise	8g or 1 teaspoon<br>1 dessertspoon of oil
Seeds (e.g. linseed, pumpkin, sunflower, sesame, chia, hemp)	7g or 1 dessertspoon
Unsalted or salted or dry roasted nuts	7g or 1 dessertspoon<br>3 walnut halves, 3 Brazil,<br>4 almonds, 8 peanuts,<br>10 cashews or pistachios
Olives	50g or 10 olives
Low fat mayonnaise, Curry paste or Harissa paste	15g or ½ oz or 1 tablespoon
Peanut butter (without palm oil)	11g or ⅓ oz or<br>1 heaped teaspoon
Cocoa powder	12 g or ⅓ oz or<br>2 heaped teaspoons
Avocado	40g or 1⅓ oz or<br>1/4 of an average pear
Low fat guacamole	40g or 1⅓ oz or 2 tablespoons

For peer review only

Manchester University
NHS Foundation Trust

MIDDAS-GDM End of Study Questionnaire

Thank you for taking part in the MIDDAS-GDM Study.

This is one of the first studies of its kind. We hope to learn as much as possible from this study, in particular the views of people who have taken part. We are inviting you to provide your views on different aspects of the study and following the diet, and how we can improve our programmes and research studies in future.

Please complete the following questions and return this questionnaire to the MIDDAS-GDM study team in the envelope provided. If there is anything else you would like to say about your experiences of the study, please use the section at the end. Your answers to the questions below will remain anonymous.

1. **What were your reasons for joining the study?**

……………………………………………………………………………………………………
……………………………………………………………………………………………………
……………………………………………………………………………………………………
……………………………………………………………………………………………………
……………………………………………………………………………………………………

2. **How satisfied were you with study overall (recruitment, appointments etc)?** *(circle)*

1	2	3	4	5	6	7	8	9	10
Not at all satisfied		Slightly satisfied		Quite satisfied		Very satisfied			Extremely satisfied

Comments:……………………………………………………………………………………
……………………………………………………………………………………………………
……………………………………………………………………………………………………

Study Appointments

3. **You were asked to attend ……….. additional face to face appointments at the hospital by the study team** *(please fill in the number)*

For peer review only - http://bmjopen.bmj.com/site/about/guidelines.xhtml

Manchester University
NHS Foundation Trust

4. **You were asked to attend ……….. additional virtual (i.e. video call or telephone) appointments by the study team** *(please fill in the number)*

5. **How do you feel about the number of additional appointments you were asked to attend?** *(tick)*

☐ I was happy with the number of appointments
☐ I would have preferred fewer face to face appointments
☐ I would have preferred more face to face appointments
☐ I would have preferred fewer virtual appointments
☐ I would have preferred more virtual appointments

Comments:………………………………………………………………………………………
………………………………………………………………………………………………

6. **How do you feel about virtual (i.e. video call or telephone) appointments?**

☐ I prefer virtual appointments to face to face appointments (please explain why below)
☐ I prefer face to face appointments to virtual appointments (please explain why below)

Comments:………………………………………………………………………………………
………………………………………………………………………………………………
………………………………………………………………………………………………
………………………………………………………………………………………………

Diet

7. **Which diet were you asked to follow? (*please tick*)**

☐ Best NHS Care (i.e. increased fruit/vegetable intake, low-GI foods, reduction in free sugars, regular meals)
☐ Intermittent low energy diet (5 days of the best NHS care diet plus 2 non-consecutive days of 1000 kcal each week)

8. **Was the diet easy to follow?**

1	2	3	4	5	6	7	8	9	10
Not at all			Slightly	Moderately		Very			Extremely

For peer review only - http://bmjopen.bmj.com/site/about/guidelines.xhtml

Manchester University
NHS Foundation Trust

Comments:……………………………………………………………………………………………
……………………………………………………………………………………………………

9. Did you enjoy following the diet plan?

1	2	3	4	5	6	7	8	9	10
Not at all		Slightly		Moderately		Very			Extremely

Comments:……………………………………………………………………………………………
……………………………………………………………………………………………………

10. Would you make any changes to the written information (i.e. diet booklets, recipes) you were given on how to follow the diet plan?

☐ Yes

☐ No

If yes, what changes would you make?

Comments:……………………………………………………………………………………………
……………………………………………………………………………………………………

11. How was your first appointment with the dietitian? *(tick all that apply)***?**

☐ The amount of information I received was OK

☐ The amount of information I received was too little

☐ The amount of information I received was too much

☐ I was happy with the advice I received

☐ The advice I received could be improved (specify below)

Comments:……………………………………………………………………………………………
……………………………………………………………………………………………………

For peer review only - http://bmjopen.bmj.com/site/about/guidelines.xhtml

Manchester University
NHS Foundation Trust

12. Would you make any changes to the reviews (calls/face to face appointments) you had with the dietitian during your pregnancy ?

☐ Yes

☐ No

Comments:………………………………………………………………………………………………
………

13. How useful was your final appointment with the dietitian at 12 weeks post-delivery?

1	2	3	4	5	6	7	8	9	10
Not at all		Slightly		Quite		Very			Extremely

Comments:………………………………………………………………………………………………
………

14. Did you feel confident to exercise whilst on the diet plan?

1	2	3	4	5	6	7	8	9	10
Not at all confident		Slightly confident		Quite confident		Very confident			Extremely confident

Comments:………………………………………………………………………………………………
………

Additional support

15. Would any of the following have been useful & if so how often?

	No	Yes	Preferred method of contact	How often
Additional support from dietitian	☐	☐	Face to face / phone	
Additional support from the midwife	☐	☐	Face to face / phone	

For peer review only - http://bmjopen.bmj.com/site/about/guidelines.xhtml

Manchester University
NHS Foundation Trust

Additional support from the doctors in the clinic	☐	☐	Face to face / phone	
More contact with other women in the study following the diets	☐	☐	Face to face / phone	
Other, please specify:				

For peer review only

16. Did you receive any support outside of the study team help to keep you on track as you progressed through the study?

☐ No

☐ Yes

If yes, what support did you receive?

…………………………………………………………………………………………………

…………………………………………………………………………………………………

Record keeping

17. How did you find the finger prick testing requirements on the study? *(tick all those that apply)*

☐ Challenging but on the whole achievable

☐ Challenging and not achievable

☐ Not challenging at all

☐ I felt it was necessary to test this often to ensure my safety

☐ I felt it was unnecessary to test this often to ensure my safety

Comments:…………………………………………………………………………………………

…………………………………………………………………………………………………

18. How did you find the ketone testing requirements on the study? *(tick all those that apply)*

☐ Challenging but on the whole achievable

☐ Challenging and not achievable

For peer review only - http://bmjopen.bmj.com/site/about/guidelines.xhtml

Manchester University NHS Foundation Trust

☐ Not challenging at all

☐ I felt it was <u>necessary</u> to test this often to ensure my safety

☐ I felt it was <u>unnecessary</u> to test this often to ensure my safety

Comments:……………………………………………………………………………………………

……………………………………………………………………………………………………

19. How did you find using Diasend software?

☐ Straightforward

☐ Challenging but on the whole <u>achievable</u>

☐ Challenging and <u>not achievable</u>

☐ I felt uncomfortable using computer software to keep track of my medical details

☐ I felt comfortable using computer software to keep track of my medical details

Comments:……………………………………………………………………………………………

……………………………………………………………………………………………………

20. How did you find completing the food diary during the study? *(tick all those that apply)*

☐ Challenging but on the whole <u>achievable</u>

☐ Challenging and <u>not achievable</u>

☐ Not challenging at all

Comments:……………………………………………………………………………………………

……………………………………………………………………………………………………

21. How did you find the physical activity questionnaires on the study? *(tick all those that apply)*

☐ Challenging but on the whole <u>achievable</u>

☐ Challenging and <u>not achievable</u>

☐ Not challenging at all

Comments:……………………………………………………………………………………………

……………………………………………………………………………………………………

For peer review only

For peer review only - http://bmjopen.bmj.com/site/about/guidelines.xhtml

Manchester University
NHS Foundation Trust

22. How did you find the quality of life questionnaires on the study? *(tick all those that apply)*

- ☐ Challenging but on the whole achievable
- ☐ Challenging and not achievable
- ☐ Not challenging at all

Comments:…………………………………………………………………………………………

……………………………………………………………………………………………………

Libro® app

23. Did you use the Libro® app?

☐ Yes

☐ No (please move to question 25)

24. Did you find the App helpful?

1	2	3	4	5	6	7	8	9	10
Not at all		Slightly		Moderately		Very			Extremely

Comments………………………………………………………………………………………

……………………………………………………………………………………………………

25. What did you like about the App?

……………………………………………………………………………………………………

……………………………………………………………………………………………………

……………………………………………………………………………………………………

26. What did you dislike about the App and could be improved?

……………………………………………………………………………………………………

……………………………………………………………………………………………………

27. If you didn't use the app what were the reasons for this? *(tick all that apply)*

☐ Would prefer not to say ☐ Already using other health apps

For peer review only - http://bmjopen.bmj.com/site/about/guidelines.xhtml

- ☐ Don't like using apps in general
- ☐ Labour intensive / time consuming
- ☐ Find mobile devices challenging
- ☐ Lack of regular internet access
- ☐ Other (provide details below)
- ☐ Not user friendly
- ☐ Prefer to use pen and paper

……………………………………………………………………………………………

……………………………………………………………………………………………

……………………………………………………………………………………………

For peer review only

Diasend Software

28. Did you find the Diasend software helpful?

1	2	3	4	5	6	7	8	9	10
Not at all		Slightly		Moderately		Very			Extremely

Comments……………………………………………………………………………………

……………………………………………………………………………………………

29. What did you like about the Diasend software?

……………………………………………………………………………………………

……………………………………………………………………………………………

30. What did you dislike about the Diasend software and could be improved?

……………………………………………………………………………………………

……………………………………………………………………………………………

Study Improvements

31. What did you enjoy most about the study?

……………………………………………………………………………………………

……………………………………………………………………………………………

……………………………………………………………………………………………

……………………………………………………………………………………………

For peer review only - http://bmjopen.bmj.com/site/about/guidelines.xhtml

Manchester University
NHS Foundation Trust

32. What did you enjoy least about the study and could be improved?

……………………………………………………………………………………………………
……………………………………………………………………………………………………
……………………………………………………………………………………………………
……………………………………………………………………………………………………
……………………………………………………………………………………………………

Any other comments about the study

……………………………………………………………………………………………………
……………………………………………………………………………………………………
……………………………………………………………………………………………………
……………………………………………………………………………………………………
……………………………………………………………………………………………………
……………………………………………………………………………………………………
……………………………………………………………………………………………………
……………………………………………………………………………………………………

Thank you for completing this questionnaire, please return to:

MIDDAS-GDM Study Team, Nightingale Centre,

Wythenshawe Hospital, Manchester, M23 9LT

For peer review only

For peer review only - http://bmjopen.bmj.com/site/about/guidelines.xhtml

The TIDieR (Template for Intervention Description and Replication) Checklist*:

Information to include when describing an intervention and the location of the information

Item number	Item	Where located ** Primary paper (page or appendix number)	Other † (details)
	**BRIEF NAME**		
**1.**	Provide the name or a phrase that describes the intervention.	2	
	**WHY**		
**2.**	Describe any rationale, theory, or goal of the elements essential to the intervention.	4-5	______________
	**WHAT**		
**3.**	Materials: Describe any physical or informational materials used in the intervention, including those provided to participants or used in intervention delivery or in training of intervention providers. Provide information on where the materials can be accessed (e.g. online appendix, URL).	12, 15	_appendix____
**4.**	Procedures: Describe each of the procedures, activities, and/or processes used in the intervention, including any enabling or support activities.	6-12	______________
	**WHO PROVIDED**		
**5.**	For each category of intervention provider (e.g. psychologist, nursing assistant), describe their expertise, background and any specific training given.	1, 7-12, 17-18	______________
	**HOW**		

For peer review only - http://bmjopen.bmj.com/site/about/guidelines.xhtml

**6.**	Describe the modes of delivery (e.g. face-to-face or by some other mechanism, such as internet or telephone) of the intervention and whether it was provided individually or in a group.	6-18	______________
	**WHERE**		
**7.**	Describe the type(s) of location(s) where the intervention occurred, including any necessary infrastructure or relevant features.	6-18	______________
	**WHEN and HOW MUCH**		
**8.**	Describe the number of times the intervention was delivered and over what period of time including the number of sessions, their schedule, and their duration, intensity or dose.	6-18	______________
	**TAILORING**		
**9.**	If the intervention was planned to be personalised, titrated or adapted, then describe what, why, when, and how.	6-18	______________
	**MODIFICATIONS**		
**10.‡**	If the intervention was modified during the course of the study, describe the changes (what, why, when, and how).	N/A	______________
	**HOW WELL**		
**11.**	Planned: If intervention adherence or fidelity was assessed, describe how and by whom, and if any strategies were used to maintain or improve fidelity, describe them.	13-14	______________
**12.‡**	Actual: If intervention adherence or fidelity was assessed, describe the extent to which the intervention was delivered as planned.	N/A	______________

For peer review only

For peer review only - http://bmjopen.bmj.com/site/about/guidelines.xhtml

** **Authors** - use N/A if an item is not applicable for the intervention being described. **Reviewers** – use '?' if information about the element is not reported/not sufficiently reported.

† If the information is not provided in the primary paper, give details of where this information is available. This may include locations such as a published protocol or other published papers (provide citation details) or a website (provide the URL).

‡ If completing the TIDieR checklist for a protocol, these items are not relevant to the protocol and cannot be described until the study is complete.

* We strongly recommend using this checklist in conjunction with the TIDieR guide (see *BMJ* 2014;348:g1687) which contains an explanation and elaboration for each item.

* The focus of TIDieR is on reporting details of the intervention elements (and where relevant, comparison elements) of a study. Other elements and methodological features of studies are covered by other reporting statements and checklists and have not been duplicated as part of the TIDieR checklist. When a **randomised trial** is being reported, the TIDieR checklist should be used in conjunction with the CONSORT statement (see www.consort-statement.org) as an extension of **Item 5 of the CONSORT 2010 Statement.** When a **clinical trial protocol** is being reported, the TIDieR checklist should be used in conjunction with the SPIRIT statement as an extension of **Item 11 of the SPIRIT 2013 Statement** (see www.spirit-statement.org). For alternate study designs, TIDieR can be used in conjunction with the appropriate checklist for that study design (see www.equator-network.org).

For peer review only

For peer review only - http://bmjopen.bmj.com/site/about/guidelines.xhtml

SPIRIT CHECKLIST

Section/Item	Item no
Title	1
Trial Registration	2a
	2b
Protocol Version	3
Funding	4
Roles and responsibilities	5a
	5b
	5c
	5d
**Introduction**	
Background and rationale	6a
	6b
Objectives	7
Trial Design	8
**Methods: Participants, interventions, and outcomes**	
Study setting	9
Eligibility criteria	10
Interventions	11a

For peer review only

	11b
	11c
	11d
Outcomes	12
Participant Timeline	13
Sample Size	14
Recruitment	15
**Methods: Assignment of interventions (for controlled tri**	
Allocation	
Sequence generation	16a
Allocation concealment mechanism	16b
Implementation	16c

For peer review only

Blinding (masking)	17a
	17b
**Methods: Data collection, management, and analysis**	
Data collection methods	18a
	18b
Data management	19
Statistical methods	20a
	20b
	20c
**Methods: Monitoring**	
Data monitoring	21a

For peer review only

	21b
Harms	22
Auditing	23
**Ethics and dissemination**	
Research ethics approval	24
Protocol amendments	25
Consent or assent	26a
	26b
Confidentiality	27
Declaration of interests	28
Access to data	29
Ancillary and post-trial care	30
Dissemination policy	31a

For peer review only

	31b
	31c
**Appendices**	
Informed consent materials	32
Biological specimens	33

For peer review only

Description	Location
Descriptive title identifying the study design, population, interventions, and, if applicable, trial acronym	1
Trial identifier and registry name. If not yet registered, name of intended registry	3
All items from the World Health Organization Trial Registration Data Set	throughout
Date and version identifier	n/a
Sources and types of financial, material, and other support	23
Names, affiliations, and roles of protocol contributors	1
Name and contact information for the trial sponsor	name only, 23
Role of study sponsor and funders, if any, in study design; collection, management, analysis, and interpretation of data; writing of the report; and the decision to submit the report for publication, including whether they will have ultimate authority over any of these activities	23
Composition, roles, and responsibilities of the coordinating centre, steering committee, endpoint adjudication committee, data management team, and other individuals or groups overseeing the trial, if applicable (see Item 21a for data monitoring committee)	18
Description of research question and justification for undertaking the trial, including summary of relevant studies (published and unpublished) examining benefits and harms for each intervention	4-5
Explanation for choice of comparators	4-5
Specific objectives or hypotheses	13-14
Description of trial design including type of trial (eg, parallel group, crossover, factorial, single group), allocation ratio, and framework (eg, superiority, equivalence, noninferiority, exploratory)	6
Description of study settings (eg, community clinic, academic hospital) and list of countries where data will be collected. Reference to where list of study sites can be obtained	6
Inclusion and exclusion criteria for participants. If applicable, eligibility criteria for study centres and individuals who will perform the interventions (eg, surgeons, psychotherapists)	7
Interventions for each group with sufficient detail to allow replication, including how and when they will be administered	9

For peer review only

Criteria for discontinuing or modifying allocated interventions for a given trial participant (eg, drug dose change in response to harms, participant request, or improving/worsening disease)	n/a
Strategies to improve adherence to intervention protocols, and any procedures for monitoring adherence (eg, drug tablet return, laboratory tests)	13
Relevant concomitant care and interventions that are permitted or prohibited during the trial	7
Primary, secondary, and other outcomes, including the specific measurement variable (eg, systolic blood pressure), analysis metric (eg, change from baseline, final value, time to event), method of aggregation (eg, median, proportion), and time point for each outcome. Explanation of the clinical relevance of chosen efficacy and harm outcomes is strongly recommended	13-14
Time schedule of enrolment, interventions (including any run-ins and washouts), assessments, and visits for participants. A schematic diagram is highly recommended (see Figure)	11-12
Estimated number of participants needed to achieve study objectives and how it was determined, including clinical and statistical assumptions supporting any sample size calculations	8
Strategies for achieving adequate participant enrolment to reach target sample size	n/a
**als)**	
Method of generating the allocation sequence (eg, computer-generated random numbers), and list of any factors for stratification. To reduce predictability of a random sequence, details of any planned restriction (eg, blocking) should be provided in a separate document that is unavailable to those who enrol participants or assign interventions	8
Mechanism of implementing the allocation sequence (eg, central telephone; sequentially numbered, opaque, sealed envelopes), describing any steps to conceal the sequence until interventions are assigned	8
Who will generate the allocation sequence, who will enrol participants, and who will assign participants to interventions	7-8

For peer review only

Who will be blinded after assignment to interventions (eg, trial participants, care providers, outcome assessors, data analysts), and how	8
If blinded, circumstances under which unblinding is permissible, and procedure for revealing a participant's allocated intervention during the trial	8
Plans for assessment and collection of outcome, baseline, and other trial data, including any related processes to promote data quality (eg, duplicate measurements, training of assessors) and a description of study instruments (eg, questionnaires, laboratory tests) along with their reliability and validity, if known. Reference to where data collection forms can be found, if not in the protocol	9-15
Plans to promote participant retention and complete follow-up, including list of any outcome data to be collected for participants who discontinue or deviate from intervention protocols	supplementary PIS
Plans for data entry, coding, security, and storage, including any related processes to promote data quality (eg, double data entry; range checks for data values). Reference to where details of data management procedures can be found, if not in the protocol	16
Statistical methods for analysing primary and secondary outcomes. Reference to where other details of the statistical analysis plan can be found, if not in the protocol	16
Methods for any additional analyses (eg, subgroup and adjusted analyses)	16
Definition of analysis population relating to protocol non-adherence (eg, as randomised analysis), and any statistical methods to handle missing data (eg, multiple imputation)	16
Composition of data monitoring committee (DMC); summary of its role and reporting structure; statement of whether it is independent from the sponsor and competing interests; and reference to where further details about its charter can be found, if not in the protocol. Alternatively, an explanation of why a DMC is not needed	18

Description of any interim analyses and stopping guidelines, including who will have access to these interim results and make the final decision to terminate the trial	n/a
Plans for collecting, assessing, reporting, and managing solicited and spontaneously reported adverse events and other unintended effects of trial interventions or trial conduct	13, 16
Frequency and procedures for auditing trial conduct, if any, and whether the process will be independent from investigators and the sponsor	16
Plans for seeking research ethics committee/institutional review board (REC/IRB) approval	n/a
Plans for communicating important protocol modifications (eg, changes to eligibility criteria, outcomes, analyses) to relevant parties (eg, investigators, REC/IRBs, trial participants, trial registries, journals, regulators)	19
Who will obtain informed consent or assent from potential trial participants or authorised surrogates, and how (see Item 32)	7, supplementary consent
Additional consent provisions for collection and use of participant data and biological specimens in ancillary studies, if applicable	n/a
How personal information about potential and enrolled participants will be collected, shared, and maintained in order to protect confidentiality before, during, and after the trial	16
Financial and other competing interests for principal investigators for the overall trial and each study site	23
Statement of who will have access to the final trial dataset, and disclosure of contractual agreements that limit such access for investigators	19
Provisions, if any, for ancillary and post-trial care, and for compensation to those who suffer harm from trial participation	supplementary PIS
Plans for investigators and sponsor to communicate trial results to participants, healthcare professionals, the public, and other relevant groups (eg, via publication, reporting in results databases, or other data sharing arrangements), including any publication restrictions	19

For peer review only

Authorship eligibility guidelines and any intended use of professional writers	n/a
Plans, if any, for granting public access to the full protocol, participant- level dataset, and statistical code	19
Model consent form and other related documentation given to participants and authorised surrogates	supplementary materials
Plans for collection, laboratory evaluation, and storage of biological specimens for genetic or molecular analysis in the current trial and for future use in ancillary studies, if applicable	15

For peer review only

Missing

For peer review only

For peer review only

For peer review only

form

For peer review only

For peer review only

MeSH Descriptors

Infant, newborn
Pregnancy
Glucose Intolerance
Diabetes, Gestational
Insulin
Hyperglycaemia
Prediabetic state
Metformin
Glycated Hemoglobin
Overweight
Obesity
Diabetes Mellitus, Type 2
Diet, Healthy
Feasibility Studies
Mobile Applications
Body Weight
Hypoglycaemic agents
Fasting
Intermittent Fasting

For peer review only

BMJ Open

Manchester Intermittent Diet in Gestational Diabetes Acceptability Study (MIDDAS-GDM): A Two-Arm Randomised Feasibility Protocol Trial of an Intermittent Low-Energy Diet (ILED) in women with Gestational Diabetes and Obesity in Greater Manchester

Journal:	*BMJ Open*
Manuscript ID	bmjopen-2023-078264.R2
Article Type:	Protocol
Date Submitted by the Author:	07-Dec-2023
Complete List of Authors:	Dapre, Elizabeth; Wythenshawe Hospital<br>Issa, Basil; Manchester University NHS Foundation Trust, Department of Endocrinology and Diabetes<br>Harvie, Michelle; University Hospital of South Manchester NHS Foundation Trust, Genesis prevention centre<br>Su, Ting-Li; University of Manchester, Dentistry<br>McMillan, Brian; The University of Manchester, Centre for Primary Care and Health Services Research<br>Pilkington, A.; Wythenshawe Hospital<br>Hanna, F.; University Hospitals of North Midlands NHS Trust<br>Vyas, Avni; Manchester Metropolitan University Faculty of Health Psychology and Social Care, Health Professionals<br>Mackie, S.; Wythenshawe Hospital<br>Yates, James; Manchester University NHS Foundation Trust<br>Evans, Benjamin; Manchester University NHS Foundation Trust<br>Mubita, Womba; Manchester University NHS Foundation Trust, Department of Endocrinology and Diabetes<br>Lombardelli, Cheryl; Manchester University NHS Foundation Trust
**Primary Subject Heading**:	Diabetes and endocrinology
Secondary Subject Heading:	Nutrition and metabolism, Obstetrics and gynaecology, Reproductive medicine
Keywords:	Diabetes in pregnancy < DIABETES & ENDOCRINOLOGY, DIABETES & ENDOCRINOLOGY, Obesity, NUTRITION & DIETETICS, Feasibility Studies, Maternal medicine < OBSTETRICS

SCHOLARONE™ Manuscripts

Manchester Intermittent Diet in Gestational Diabetes Acceptability Study (MIDDAS-GDM): A Two-Arm Randomised Feasibility Protocol Trial of an Intermittent Low-Energy Diet (ILED) in women with Gestational Diabetes and Obesity in Greater Manchester

Authors: Dapre, E., Issa, B., Harvie, M., Su, T., McMillan, B., Hanna, F., Pilkington, A., Vyas, A., Yates, J., Mackie, S., Evans, B., Mubita, W., Lombardelli, C.

Dr Elizabeth Dapre (corresponding author)	elizabeth.dapre@hotmail.com<br>c/o Michelle Harvie, The Nightingale Centre, Wythenshawe Hospital, Southmoor Rd, Manchester M23 9LT
Dr Basil Issa	Diabetes and Endocrinology, Wythenshawe Hospital, Manchester, UK
Dr Michelle Harvie	The Nightingale Centre, Wythenshawe Hospital, Manchester, UK
Dr Ting-Li Su	Division of Dentistry, The University of Manchester, UK
Dr Brian McMillan	Division of Population Health, The University of Manchester, UK
Prof Fahmy Hanna	Diabetes and Endocrinology, University Hospitals of North Midlands, UK
Dr Andrea Pilkington	Obstetrics, St Mary's Wythenshawe Hospital, Manchester, UK
Avni Vyas	Division of Dietetics, Manchester Metropolitan University, UK
Benjamin Evans	Division of Clinical Trials, Manchester Foundation Trust, UK
James Yates	Division of Clinical Trials, Manchester Foundation Trust, UK
Sarah Mackie	Division of Clinical Trials, Manchester Foundation Trust, UK
Womba Mubita	Division of Clinical Trials, Manchester Foundation Trust, UK
Cheryl Lombardelli	The Nightingale Centre, Wythenshawe Hospital, Manchester, UK

Word Count: 3572

Submitted to: British Medical Journal Open

Start date of trial: 15th November 2022 (trial ongoing)

Date of submission:

For peer review only

Manchester Intermittent Diet in Gestational Diabetes Acceptability Study (MIDDAS-GDM): A Two-Arm Randomised Feasibility Protocol Trial of an Intermittent Low-Energy Diet (ILED) in women with Gestational Diabetes and Obesity in Greater Manchester

Authors: Dapre, E., Issa, B., Harvie, M., Su, T., McMillan, B., Hanna, F., Pilkington, A., Vyas, A., Yates, J., Mackie, S., Evans, B., Mubita, W., Lombardelli, C.

Corresponding Author: elizabeth.dapre@nhs.net

Abstract (word count 298)

Introduction: The prevalence of gestational diabetes mellitus (GDM) is rising in the UK and is associated with maternal and neonatal complications. National Institute for Health and Care Excellence (NICE) guidance advises first line management with healthy eating and physical activity which is only moderately effective for achieving glycaemic targets. Approximately 30% of women require medication with metformin and/or insulin. There is currently no strong evidence base for any particular dietary regimen to improve outcomes in GDM. Intermittent low-energy diets (ILEDs) are associated with improved glycaemic control and reduced insulin resistance in type 2 diabetes (T2DM) and could be a viable option in the management of GDM. This study aims to test the safety, feasibility and acceptability of an ILED intervention amongst women with GDM compared to best National Health Service (NHS) care.

Method and analysis: We aim to recruit 48 women with GDM diagnosed between 24-28 weeks gestation from antenatal clinics at Wythenshawe and St Mary's hospitals, Manchester Foundation Trust, over 13 months starting in November 2022. Participants will be randomised (1:1) to ILED (2 low-energy diet days/week of 1000kcal and 5 days/week of the best NHS care healthy diet and physical activity advice) or best NHS care 7 days/week until delivery of their baby. Primary outcomes include uptake and retention of participants to the trial, and adherence to both dietary interventions. Safety outcomes will include birthweight, gestational age at delivery, neonatal hypoglycaemic episodes requiring intervention, neonatal hyperbilirubinaemia, admission to special care baby unit or neonatal intensive care unit, stillbirths, the percentage of women with hypoglycaemic episodes requiring

For peer review only

third-party assistance, and significant maternal ketonaemia (defined as ≥1.0mmol/L) Secondary outcomes will assess the fidelity of delivery of the interventions, and qualitative analysis of participant and healthcare professionals' experiences of the diet. Exploratory outcomes include the number of women requiring metformin and/or insulin.

For peer review only

Ethics and dissemination: Ethical approval has been granted by the Cambridge East Research Ethics Committee (22/EE/0119). Findings will be disseminated via publication in peer-reviewed journals, conference presentations, and shared with diabetes charitable bodies and organisations in the UK, such as Diabetes UK and the Association of British Clinical Diabetologists.

Trial Registration Number: NCT05344066

Strengths and limitations of this study
**Strengths**
• This study adds to the limited literature of safety of low-calorie diets amongst women with gestational diabetes
• This study has been informed by an experienced patient and public involvement and engagement group
**Limitations**
• This study involves a small sample size and is not powered to show efficacy of the intervention
• Women joining this study are likely to be highly motivated and adherence may not reflect that seen in the wider general population

INTRODUCTION (word count: 3539)

Background

In the UK up to 16% of pregnant women develop gestational diabetes (GDM) and the incidence is rising, in part due to increasing rates of obesity and maternal age(1,2). GDM is associated with maternal and neonatal complications (the risk increases with poor glycaemic control), including macrosomia, shoulder dystocia, caesarean-sections, neonatal hypoglycaemia and/or hyperbilirubinaemia, preterm delivery, preeclampsia, and stillbirth(2). Women who have had GDM have an estimated seven to ten-fold risk of developing type 2 diabetes (T2DM) later in life, and their children have a higher risk of developing adult obesity and T2DM(2–4).

Excessive weight gain in pregnancy is a particular problem for women with GDM(5). Harper *et al* demonstrated that, in women with GDM, every additional 1lb/week gained following diagnosis of GDM resulted in a 36-83% increased risk of pre-eclampsia, caesarean-section, macrosomia, and large for gestational age babies(5). Such studies highlight the importance of adequate weight control throughout pregnancy in women with GDM in order to reduce both maternal and neonatal complications.

First-line therapy for GDM is diet and physical activity. National Institute for Health and Care Excellence (NICE) guidance encourages a healthy diet with increased fruit

For peer review only

and vegetables, low-glycaemic index (GI) foods, reduced refined sugars, regular mealtimes and regular physical activity(6,7). These dietary measures fail to achieve glycaemic targets in ~30% of women who require medication with metformin and/or insulin(8). A range of dietary approaches have been studied including daily diets promoting low-GI diets (limiting refined and promoting complex carbohydrates), continuous modest energy-restriction (1800 Kcal/day), and low carbohydrate diets(9). There is currently no strong evidence base for any particular dietary regimen to improve outcomes in GDM.

Intermittent Low-Energy Diets (ILED)

The pathogenesis of GDM is strongly linked to obesity and chronic insulin resistance with many clinicians viewing GDM as a form of evolving T2DM. ILEDs typically include several days of a food based or meal replacement (e.g. drinks/bars) low-energy diet (650-1000kcal) diet, with a standard healthy (non-restrictive) diet recommended on the remaining days of the week. These diets are associated with significant reductions in weight, insulin resistance and hyperglycaemia in patients with prediabetes (HbA1c between 42-47mmol/mol, impaired glucose tolerance, or impaired fasting glycaemia), those with T2DM, and otherwise healthy subjects with overweight/obesity(10–17). These changes are equivalent to, or greater than, those achieved with standard daily energy restriction. A popular intermittent diet involves 2 consecutive or non-consecutive days/week of a low-energy diet (650-1000kcal) and 5 days of normal eating/week, known as the 5:2 diet. The Manchester Intermittent vs. Daily Diabetes App Study (MIDDAS), a study comparing an ILED and a continuous low-energy diet in T2D conducted in our unit, has shown the feasibility and safety of an ILED (800kcal for 2 days/week) in patients with T2DM and obesity, including those using insulin(18). At the end of the study approximately 70% of participants in the ILED group completed the study and achieved a 6% reduction of their baseline body weight. Forty two percent achieved an HbA1c of <48 mmol/mol(18). Given the strong overlap between GDM and T2DM, an ILED may be a promising dietary intervention for those with GDM.

A successful dietary approach to glycaemic control could empower women to take charge of the management of their GDM. Women with GDM are motivated to modify their diet driven by a desire to improve foetal outcomes(19–21).

For peer review only

Our Patient and Public Involvement and Engagement (PPIE) work indicates that women find the current National Institute for Health and Care Excellence (NICE) healthy eating guidance(6,7) confusing and vague. Our PPIE work has indicated that women are keen to try alternative dietary approaches, particularly if alternative diets are more effective in preventing the need to progress to medications such as metformin and insulin.

Aim

The aim of this trial is to test the safety, feasibility, and acceptability of an ILED in GDM to inform a future large-scale RCT.

METHODS

Trial Design

The study is a 28-week feasibility two-arm RCT in one NHS trust performed in patients with GDM and BMI ≥27.5 kg/m^2, or ≥25 kg/m^2 in high-risk minority ethnic groups (i.e. South Asian, Black African, African Caribbean) in Greater Manchester, between December 2022 and July 2024(22,23). There will be an embedded qualitative sub-study for participants and healthcare professionals. Due to the nature of the intervention, it will not be possible to blind the participants, clinicians, or study team to the treatment allocation after randomisation (the statistician and laboratory technicians will remain blinded).

Trial Setting and Recruitment

Participants will be recruited from antenatal clinics at Wythenshawe and St Mary's Hospitals, Manchester Foundation Trust (MFT) between November 2022 and December 2023. This is an urban area within Greater Manchester, and MFT serves patients from a wide range of minority ethnic and socio-demographic backgrounds. Women may self-refer to the antenatal clinic or be referred by their primary care team. Assessments will be carried out at MFT, or remotely if required by COVID-19 restrictions. The qualitative sub-study will be carried out at MFT, remotely, or at a location of the participant's choosing. We aim to recruit eligible participants over a period of 13 months. Potential participants will be given written information about the study and the opportunity to ask questions about the study prior to providing written consent (figure 1).

For peer review only

Eligibility Criteria

<IMAGE ONE; figure1 inclusion exclusion jpg>

Participant Flow

Participants who fulfil the broad eligibility criteria will be notified about the trial by the GDM nurse/midwife at the time of their diagnosis. Those who are interested will be provided with a comprehensive patient information sheet (see appendix) and more detailed eligibility screening questions. They will be asked to attend their first appointment having fasted for at least 6 hours and complete a four-day food diary (in line with our departments usual care). On attending their first routine clinic appointment, interested participants will receive further information from the research team. They will have the opportunity to ask questions, have their eligibility confirmed, and will be asked for their written consent to take part. Baseline assessments will be taken and participants will be randomised to their allocated treatment group using an online randomisation platform. Participant flow through the study is demonstrated in figure 2.

Sample Size

We plan to recruit 24 participants per study arm (n=48) which, when considering an estimated attrition rate of 15%, will provide complete outcome data on 40 participants(24–26). It has been estimated that 24 participants per group will be sufficient to determine study outcomes, in line with sample size recommendations for feasibility studies(27–29).

This number will allow us to enable estimation of recruitment/retention parameters with sufficient precision. For example, based on 40 completed participants, it will enable recruitment rates in the region of 25% to be estimated with an error of +/-13.42% at most; retention of 85% will be estimated with error of +/-11.07% at most. It is also sufficient for estimation of variability (e.g. standard deviation) in gestational weight gain and capillary glucose concentrations (proposed outcomes for the future definitive trial) with negligible bias (30).

For peer review only

Randomisation

The randomisation schedule will be independently set up and known only by the trial statistician. The trial statistician will be blinded to the participant's identity using "sealed envelope" software (https://www.sealedenvelope.com/). Randomisation will be carried out by generating an online pseudo-random list with random permuted blocks of varying size, known only to the statistician, and will be stratified for two variables:

- Age (18-35, >35 years)
- BMI (27.5-34.99kg/m^2 and >35kg/m2, >25-32.49kg/m^2 and >32.5kg/m^2 for high-risk minority ethnic groups (i.e. South Asian, Black African, African Caribbean)

These stratification variables have been chosen to reduce potential bias as we expect varying severity of GDM with increasing age and BMI, and possible differences in diet adherence(31).

Treatment to intervention and control groups will be allocated in a 1:1 ratio. A member of the research team who will be unaware of the randomisation algorithm (principal investigator, clinical research nurse, clinical research fellow or project manager) will trigger the randomisation procedure onsite; participants and clinicians will then be informed of the allocated treatment group. Clinicians will not be blinded due to the need to remain astute to safety, adherence, and side effects, requiring open and honest discussions with patients at each appointment. The statistician will remain blinded to treatment allocation until all outcome measures for all subjects have been collected.

Interventions

Study Arm 1: Best NHS Care Diet

All dietetic advice will be face to face or via video calls or the telephone. Participants will receive one to one personalised written and verbal advice from a dietitian to follow NICE diet and physical activity recommendations(6,7). Dietitians and midwives will receive training to ensure standardised delivery of information in clinic, and standardised patient information leaflets will be supplied to include information about increased fruit/vegetable intake, low-glycaemic index foods, and a reduction in free sugars. Information will include advice about the importance of regular meals; dietary advice aims to ensure that participants include at least 70g protein/28g fibre, and

For peer review only - http://bmjopen.bmj.com/site/about/guidelines.xhtml

predominantly mono- and polyunsaturated fats as per American Diabetes Association recommendations(32). Participants will be advised to be physically active, for example walking for 30 minutes after a meal. Participants will receive ongoing dietetic education and support every 2 weeks until delivery. This level of support is higher than typically provided in NHS GDM antenatal clinics due to limited resources but has been utilised to reflect best NHS care. They will receive suggested menus and recipes to follow the NICE recommended healthy diet for GDM. Participants will be asked to measure their capillary glucose four times each day and their ketones on two random (recorded) days of the week of their choosing (see appendix).

Study Arm 2: Intermittent Low-Energy Diet (ILED)

Participants will receive the same level of dietetic support as the best NHS care group. They will be given advice on adopting an ILED which involves 2 non-consecutive low-energy diet days/week (1000kcal to include 100g low-GI carbohydrate and 70g of protein) and 5 days/week of the NICE healthy eating low-GI diet and physical activity recommended for the best NHS care group. The low-energy days involve women selecting a set number of portions of protein, carbohydrate, fat, fruit, vegetables, and dairy/dairy alternatives as described in previous studies(33). Each low-energy day includes ~210g of lean protein foods, 3-4 portions of wholegrain carbohydrates, 1x7g portion of fat, 5 portions of vegetables, 2 of fruit, and 3 of dairy/dairy alternatives. Food and drink will be self-selected and not provided by the study team. Participants will be provided with comprehensive food lists, advice on portion sizes for the low-energy days and suggested menus and recipes to follow for both the low-energy and NICE recommended healthy diet days. Both diets can be successfully adapted for people of different ethnicities and those following omnivorous, vegetarian and vegan diets. Participants will be asked to measure their capillary glucose four times each day and their ketones on (and the morning after) the two low-energy days (see appendix).

<IMAGE 2: figure 2 flow jpg>

<IMAGE 3: figure 3 sched assessments jpg>

For peer review only

Outcomes

Primary outcomes

- Uptake rate measured as a percentage of eligible participants who consent to take part, including the proportion of women who were screened who did not meet the eligibility criteria, and the number of women who did not give consent to take part
- Recruitment rate measured as the number of eligible participants who consent to take part per month
- Retention rate measured as the number of randomised participants who complete the trial (those who attend the final visit) and the percentage of participants who attend all 8 visits
- Adherence to the dietary interventions assessed from self-reported adherence to the potential low-calorie days between randomisation and delivery
- Completion of self-assessed glucose and ketone readings assessed as a percentage of the required readings

- Safety outcomes:
  - Percentage of women following ILED/best NHS care with hypoglycaemia (episodes of blood glucose of <3.0mmol/mol) and hypoglycaemia requiring third-party assistance as measured by participants
  - Percentage of women who develop significant ketonaemia in both groups (defined as ≥1.0mmol/L) as measured by participants
  - Percentage of neonatal hypoglycaemic episodes requiring intervention (blood glucose checked 2- hours post-delivery and 2-hours thereafter for 12 hours according to local protocol), neonatal birth weight, gestational age at delivery, hyperbilirubinaemia/jaundice, and/or admission to Special Care Baby Unit or neonatal intensive care, and stillbirths
  - The incidence and rate of other adverse events (e.g. headaches, lethargy, constipation, or complications requiring hospital admission)

For peer review only

between the start of the trial intervention and delivery recorded as mild, moderate and severe, as defined by Common Terminology Criteria for Adverse Events version 5 (CTCAEv5)(34). Hospital admission for routine labour and delivery will not be classified as an adverse event.

Secondary outcomes

- Completeness of collection of trial endpoints including the percentage of completed weight measurements, 4-day food diaries, and International Physical Activity Questionnaire (IPAQ) scores
- Fidelity of delivery of the interventions will be measured through the number and modality of completed planned patient contacts, electronic and paper food diaries, and self-reported capillary glucose and ketone measurements
- Qualitative analysis of the acceptability and implementation of the interventions will be explored amongst a subset of participants (~10 in each group) and healthcare professionals through in-depth interviews

Exploratory outcomes

The following outcomes will be explored without statistical inference.

1. Maternal outcomes:
    - The percentage of women requiring metformin and/or insulin
    - Four-point capillary glucose profiles during third trimester (four times daily until delivery)
    - Change in fasting blood test results between baseline measurements, 36-37 weeks' gestation, and 12 weeks post-delivery (including oral glucose tolerance tests (OGTT)
    - Mode of delivery, development of preeclampsia, polyhydramnios (maximum liquor volume pool depth ≥8 cm)
    - Quality of life and health status questionnaires (WHOQoL-BREF and SF-36 questionnaires)(35,36)
2. Foetal outcomes:
    - Foetal weight
    - Gestational age at delivery

For peer review only

Measurements

The full schedule of assessments can be found in figure 3.

Physical measurements

Height, weight and blood pressure will be measured using standardised calibrated equipment in antenatal clinic.

Blood samples

Fasting venous blood samples will be collected to assess maternal HbA1c, fasting glucose, insulin, beta-hydroxybutyrate, liver function tests, free fatty acids, thyroid function tests, and full blood count. A cord blood sample will be collected at the time of delivery to measure neonatal glucose, and insulin and C-peptide where collection is possible. At the end of the study all samples will be disposed of in accordance with the Human Tissue Act (2004).

Questionnaires

Participants will be asked to complete four questionnaires at four time points throughout the trial (self-reported). Quality of life and health status will be assessed using the World Health Organisation Quality of Life Questionnaire (brief version) and the 36-Item Short Form Survey respectively(35,36). Physical activity will be measured using the International Physical Activity Questionnaire – Short Form, and diet quality will be assessed using the UK Diabetes and Diet Questionnaire(37,38). These questionnaires are self-reported by participants and have been chosen as they are widely used and validated tools.

Food Diaries

4-day dietary records will be completed using Libro (Nutritics Mobile Application) or paper food diaries, which will be entered into Nutritics software (Nutritics, Dublin, Ireland)(39). Participants who wish to use Libro will receive one to one training to use this by the study dietitian. Diaries will provide the research team with information

For peer review only

about the intake of energy, carbohydrate, fat, protein, fibre, glycaemic index, and the timing of meals for participants in both groups. Participants will be asked what other dietary modifications, if any, they have made at their fortnightly dietitian reviews to establish the adoption of any alternative dietary practices in the cohort.

Adverse Events

Participants in both groups will be asked about any adverse effects that they have experienced at each visit. These will include, but are not limited to, the potential effects of a low-energy diet, e.g. headache, lethargy, dizziness, constipation, indigestion, poor concentration, and hunger. Adverse events will be graded as per CTAEv5(34). Participants will be issued with a participation/emergency card with emergency contact details for the research team to be carried at all times and to be shown to the attending physician in case of emergency admission to hospital. All participants will be issued with clear instructions as to how to manage a hypoglycaemic and/or ketonaemic event (see appendix).

Data management

Participant data will be anonymised and will be stored in line with the Medicines for Human Use (Clinical Trials) Amended Regulations 2006 and the Data Protection Act (2018) and archived in line with the Medicines for Human Use (Clinical Trials) Amended Regulations (2006) as defined in the MFT Clinical Trials Office Archiving SOP (11; Retention of Data, Off-Site Archiving, and Destroying Documents). Deidentified data will be stored in a study-specific Research Electronic Data Capture (REDCap) database. The sponsor will periodically audit the site study file, a sample of the case report form, consent forms, and source data, and check accuracy of the study database to ensure satisfactory completion.

Statistical methods

A statistical analysis plan specifying the full details of the primary and secondary outcomes, other variables, and methods, will be produced prior to trial analysis. The main analysis will be conducted via intention-to-treat population and will not undertake any significance tests. Descriptive, graphical (summary), and basic statistics (e.g. i. number, frequencies and percentages, ii. mean and standard deviation, or iii. median and quartiles as appropriate) will be presented as appropriate for each group respectively, for group difference jointly, and for each

For peer review only

stratum. Per-protocol analysis will be considered as a secondary analysis. Levels of missing data will be investigated and used to inform future studies. No imputation will be used. The end of study questionnaire will be analysed using appropriate descriptive statistics for closed questions and key themes will be extracted without formal analysis from open questions to inform future research.

Progression Criterion

The success of the feasibility trial will be defined by the progression criteria as outlined in table 1. Any concerns regarding a low retention rate will be discussed with the PPIE group. Interviews will include those who withdraw from the study to address potential reasons for withdrawal with the aim to improve retention in future.

	Feasible (green)	Feasible with modification of the protocol (amber)	Not feasible (red)
**Recruitment**	≥4 patients/month	>2 patients/month	≤2 patients/month
**Uptake to the feasibility study**	≥15%	10-15%	<10%
**Retention to the feasibility study**	>70%	50-70%	<50%
**Adherence to the ILED intervention**	>50% of the low-energy days completed (2/week between weeks 24-28 and delivery)	30-50% of the low-energy days completed (2/week between weeks 24-28 and delivery)	<30% of the low-energy days completed (2/week between weeks 24-28 and delivery)

Table 1: Trial progression criterion

Qualitative sub-study

Participants will be invited to take part in an optional qualitative sub-study at 11-13 weeks post-partum. Healthcare professionals delivering the interventions will also be invited to take part in this study.

We will undertake 11-12 semi-structured interviews with a subset of women from each group (ILED n=10 and best NHS Care n=10) at around 12 weeks post-delivery. The final sample size will be contingent on obtaining data saturation. We will also

For peer review only - http://bmjopen.bmj.com/site/about/guidelines.xhtml

interview a sample of healthcare professionals (HCPs) involved in the delivery of care to study participants, including dieticians, obstetricians and midwives, including those with leadership and clinical managerial roles. Sampling will be purposive, aiming to obtain women from a range of ethnic groups, ages, socioeconomic backgrounds, and self-reported engagement with the intervention. Participants and HCPs will be asked about their experiences and thoughts regarding the intervention, including motivating factors, and facilitators/barriers to engagement. Interviews will be conducted by a researcher from the University of Manchester/MFT who is independent from the research staff involved in the delivery and assessment of the programmes. Analysis will be conducted by two independent researchers at the University of Manchester/MFT using Braun and Clarke's thematic analysis approach to identify key issues around the acceptability, usefulness of the programmes, and feasibility of a subsequent trial(40). Analysis will be inductive: open-ended, exploratory, and driven by the data.

All participants will also be asked to complete an optional and anonymous end of study questionnaire developed by the study team at their post-partum visit (see appendix). This will give participants the opportunity to feedback on their experience and will enable the study team to identify improvements to the design of a possible follow-up study.

Trial Steering Committee (TSC)

The trial steering committee will include an independent consultant endocrinologist, obstetrician, dietitian, and the patient representative. The committee will oversee the trial to ensure that it is carried out to the expected standards. The TSC will liaise with the CI to develop a schedule of meetings, proposed to occur every four months, with meetings to occur no less than annually. Minutes will be taken at TSC meetings and copies of the minutes will be filed in the Trial Master File; they will be shared with relevant stakeholders as appropriate.

Patient and public involvement

Patient and public involvement was actively sought throughout the planning and design of this trial and continues to form a key part of the trial as it progresses. The patient and public involvement and engagement (PPIE) group assisted in the

For peer review only

development of all participant materials and provided valuable insight into the wording of participant information and acceptability of the proposed intervention. The PPIE group will be updated as the trial progresses and a further focus group will be held to advise on the interview schedule and wording for the qualitative sub-study. The group will also be invited to aid in the development of summarising key findings for dissemination to relevant patient groups.

Ethics and dissemination

This study has been approved by the Cambridge East Research Ethics Committee and is sponsored by MFT. Findings will be disseminated via publication in peer-reviewed journals, conference presentations, and shared with diabetes charitable bodies and organisations in the UK, such as Diabetes UK and the Association of British Clinical Diabetologists. Anonymised data will be available upon formal request once the principal results of the study have been published. Planned modifications to the protocol will be approved by the research ethics committee before they are adopted into the study. An audit trail of ethical amendments and documentation will allow monitoring by the research team and external regulatory bodies.

This is the first study to assess the feasibility and safety of an ILED in GDM as compared to best NHS care. Given the increasing incidence of GDM and associated health risks this research is both pertinent and important. The study is not powered to show differences between ILED and best NHS care, however the planned quantitative and qualitative assessments will inform the feasibility of the programme and a future definitive trial.

For peer review only

Full References

1. The British Diabetic Association. Diabetes UK. [cited 2019 Nov 27]. What is gestational diabetes? Available from: https://www.diabetes.org.uk/diabetes-the-basics/gestational-diabetes

2. Kim C. Gestational diabetes: risks, management, and treatment options. Int J Womens Health. 2010 Oct 7;2:339–51.

3. Kim SY, Sharma AJ, Callaghan WM. Gestational diabetes and childhood obesity: what is the link? Curr Opin Obstet Gynecol. 2012 Dec;24(6):376–81.

4. Vounzoulaki E, Khunti K, Abner SC, Tan BK, Davies MJ, Gillies CL. Progression to type 2 diabetes in women with a known history of gestational diabetes: systematic review and meta-analysis. BMJ. 2020 May 13;m1361.

5. Harper L, Tita A, Biggio J. The Institute of Medicine Guidelines for Gestational Weight Gain after a Diagnosis of Gestational Diabetes and Pregnancy Outcomes. Am J Perinatol. 2014 Jun 27;32(03):239–46.

6. Webber J, Charlton M, Johns N. Diabetes in pregnancy: management of diabetes and its complications from preconception to the postnatal period (NG3). Br J Diabetes. 2015 Sep 1;15(3):107.

7. NHS. NHS Treatment Gestational Diabetes [Internet]. 2019 [cited 2020 Jan 10]. Available from: https://www.nhs.uk/conditions/gestational-diabetes/treatment/

8. Mayo K, Melamed N, Vandenberghe H, Berger H. The impact of adoption of the international association of diabetes in pregnancy study group criteria for the screening and diagnosis of gestational diabetes. Am J Obstet Gynecol. 2015 Feb;212(2):224.e1-9.

9. Han S, Middleton P, Shepherd E, Van Ryswyk E, Crowther CA. Different types of dietary advice for women with gestational diabetes mellitus. Cochrane Database Syst Rev. 2017 25;2:CD009275.

10. Carter S, Clifton PM, Keogh JB. Effect of Intermittent Compared With Continuous Energy Restricted Diet on Glycemic Control in Patients With Type 2 Diabetes: A Randomized Noninferiority Trial. JAMA Netw Open. 2018 Jul 20;1(3):e180756.

For peer review only

11. Antoni R, Johnston KL, Collins AL, Robertson MD. Effects of intermittent fasting on glucose and lipid metabolism. Proc Nutr Soc. 2017 Aug;76(3):361–8.

12. Cioffi I, Evangelista A, Ponzo V, Ciccone G, Soldati L, Santarpia L, et al. Intermittent versus continuous energy restriction on weight loss and cardiometabolic outcomes: a systematic review and meta-analysis of randomized controlled trials. J Transl Med. 2018 Dec;16(1):371.

13. Harris L, Hamilton S, Azevedo LB, Olajide J, De Brún C, Waller G, et al. Intermittent fasting interventions for treatment of overweight and obesity in adults: a systematic review and meta-analysis. JBI Database Syst Rev Implement Rep. 2018 Feb;16(2):507–47.

14. Harvie M, Howell A. Potential Benefits and Harms of Intermittent Energy Restriction and Intermittent Fasting Amongst Obese, Overweight and Normal Weight Subjects—A Narrative Review of Human and Animal Evidence. Behav Sci. 2017 Jan 19;7(4):4.

15. Harvie MN, Pegington M, Mattson MP, Frystyk J, Dillon B, Evans G, et al. The effects of intermittent or continuous energy restriction on weight loss and metabolic disease risk markers: a randomized trial in young overweight women. Int J Obes. 2011 May;35(5):714–27.

16. Wang X, Li Q, Liu Y, Jiang H, Chen W. Intermittent fasting versus continuous energy-restricted diet for patients with type 2 diabetes mellitus and metabolic syndrome for glycemic control: A systematic review and meta-analysis of randomized controlled trials. Diabetes Res Clin Pract. 2021 Sep;179:109003.

17. He S, Wang J, Zhang J, Xu J. Intermittent Versus Continuous Energy Restriction for Weight Loss and Metabolic Improvement: A Meta-Analysis and Systematic Review. Obesity. 2021 Jan;29(1):108–15.

18. McDiarmid S, Harvie M, Johnson R, Vyas A, Aglan A, Moran J, et al. Manchester Intermittent versus Daily Diet App Study (MIDDAS): A pilot randomized controlled trial in patients with type 2 diabetes. Diabetes Obes Metab. 2022 Mar;24(3):432–41.

19. Carolan M. Women's experiences of gestational diabetes self-management: A qualitative study. Midwifery. 2013 Jun;29(6):637–45.

20. Gao F, Luo H, Jones K, Nicholson W, Bell RA. Gestational Diabetes and Health Behaviors Among Women: National Health and Nutrition Examination Survey, 2007–2014. Prev Chronic Dis. 2018 Oct 25;15:180094.

21. McMillan B, Easton K, Goyder E, Delaney B, Madhuvrata P, Abdelgalil R, et al. Reducing risk of type 2 diabetes after gestational diabetes: a qualitative study to explore the potential of technology in primary care. Br J Gen Pract. 2018 Apr;68(669):e260–7.

22. National Institute for Health and Care Excellence. Diabetes in pregnancy: management from preconception to the postnatal period. NICE guideline. [Internet]. 2020. Available from: https://www.nice.org.uk/guidance/ng3/resources/diabetes-in-pregnancy-management-from-preconception-to-the-postnatal-period-pdf-51038446021

For peer review only

23. National Institute for Health and Care Excellence. Obesity: identification, assessment and management. NICE guideline. [Internet]. 2014 Nov [cited 2023 Aug 30]. Available from: https://www.nice.org.uk/guidance/cg189/resources/obesity-identification-assessment-and-management-pdf-35109821097925

24. H. Al Wattar B, Dodds J, Placzek A, Beresford L, Spyreli E, Moore A, et al. Mediterranean-style diet in pregnant women with metabolic risk factors (ESTEEM): A pragmatic multicentre randomised trial. Persson LÅ, editor. PLOS Med. 2019 Jul 23;16(7):e1002857.

25. Moreno-Castilla C, Hernandez M, Bergua M, Alvarez MC, Arce MA, Rodriguez K, et al. Low-carbohydrate diet for the treatment of gestational diabetes mellitus: a randomized controlled trial. Diabetes Care. 2013 Aug;36(8):2233–8.

26. Rae A, Bond D, Evans S, North F, Roberman B, Walters B. A randomised controlled trial of dietary energy restriction in the management of obese women with gestational diabetes. Aust N Z J Obstet Gynaecol. 2000 Nov;40(4):416–22.

27. Julious SA. Sample size of 12 per group rule of thumb for a pilot study. Pharm Stat. 2005 Oct;4(4):287–91.

28. Sim J, Lewis M. The size of a pilot study for a clinical trial should be calculated in relation to considerations of precision and efficiency. J Clin Epidemiol. 2012 Mar;65(3):301–8.

29. Lancaster GA. Pilot and feasibility studies come of age! Pilot Feasibility Stud. 2015 Dec;1(1):1, 2055-5784-1–1.

30. Teare MD, Dimairo M, Shephard N, Hayman A, Whitehead A, Walters SJ. Sample size requirements to estimate key design parameters from external pilot randomised controlled trials: a simulation study. Trials. 2014 Dec;15(1):264.

31. Makgoba M, Savvidou M, Steer P. An analysis of the interrelationship between maternal age, body mass index and racial origin in the development of gestational diabetes mellitus: Gestational diabetes mellitus and risk factors. BJOG Int J Obstet Gynaecol. 2012 Feb;119(3):276–82.

32. American Diabetes Association. 13. Management of Diabetes in Pregnancy: *Standards of Medical Care in Diabetes—2018*. Diabetes Care. 2018 Jan;41(Supplement 1):S137–43.

33. Harvie M, Wright C, Pegington M, McMullan D, Mitchell E, Martin B, et al. The effect of intermittent energy and carbohydrate restriction *v* . daily energy restriction on weight loss and metabolic disease risk markers in overweight women. Br J Nutr. 2013 Oct 28;110(8):1534–47.

34. National Cancer Institute. Cancer Therapy Evaluation Program. 2017 [cited 2020 May 7]. Common Terminology Criteria for Adverse Events (CTCAE). Available from: https://ctep.cancer.gov/protocolDevelopment/electronic_applications/ctc.htm#ctc_50

For peer review only

For peer review only - http://bmjopen.bmj.com/site/about/guidelines.xhtml

35. World Health Organisation. WHOQOL: Measuring Quality of Life [Internet]. Available from: https://www.who.int/tools/whoqol

36. Nair R, Kachan P. Outcome tools for diabetes-specific quality of life: Study performed in a private family practice clinic. Can Fam Physician Med Fam Can. 2017 Jun;63(6):e310–5.

37. Ekelund U, Sepp H, Brage S, Becker W, Jakes R, Hennings M, et al. Criterion-related validity of the last 7-day, short form of the International Physical Activity Questionnaire in Swedish adults. Public Health Nutr. 2006 Apr;9(2):258–65.

38. England CY, Thompson JL, Jago R, Cooper AR, Andrews RC. Development of a brief, reliable and valid diet assessment tool for impaired glucose tolerance and diabetes: the UK Diabetes and Diet Questionnaire. Public Health Nutr. 2017 Feb;20(2):191–9.

39. Nutritics [Internet]. [cited 2020 Jul 12]. Available from: https://www.nutritics.com/p/home?utm_source=adwords&utm_campaign=Nutritics+Brand&utm_term=&utm_medium=ppc&hsa_grp=78385303355&hsa_mt=b&hsa_kw=&hsa_src=g&hsa_ad=380634402696&hsa_cam=1619635404&hsa_net=adwords&hsa_acc=2361676125&hsa_ver=3&hsa_tgt=dsa-814211721343&gclid=Cj0KCQjw6ar4BRDnARIsAITGzlAm_uy2YNRBU5N-egXXyehUQ_9CHKylLQI_LgCYJK9Jpet4oIvUM5saAkzREALw_wcB

40. Braun V, Clarke V. Successful qualitative research: a practical guide for beginners. Los Angeles: SAGE; 2013. 382 p.

Authors' contributions

Michelle Harvie, Basil Issa and Elizabeth Dapre designed the study, wrote the protocol and secured funding. Brian McMillan designed and worded the qualitative sub-study. Ting-Li Su developed the statistical analysis plan. Fahmy Hanna reviewed and advised on overall study design. Andrea Pilkington provided expert obstetric guidance for the protocol design. Avni Vyas, Cheryl Lombardelli and Michelle Harvie conducted dietetic reviews. Womba Mubita helped with recruitment and review of participants. James Yates and Benjamin Evans were responsible for project management and data reporting. Sarah Mackie coordinated the clinical trial. Elizabeth Dapre drafted the manuscript for publication, with input from Michelle Harvie, Basil Issa, Brian McMillan and Ting-Li Su. All authors have proofed and checked the manuscript.

For peer review only

Acknowledgments

With thanks to Mrs Rebecca Lumsden, our patient expert, whose insight has been invaluable in the design of the study. We are most grateful to Mrs Jodie Aspinall, and Miss Lisa Brew-Butler, specialist midwives who continue to help identify suitable candidates for the study, and Jessy Athirampuzha who has played a critical role in the recruitment and follow up of participants throughout the trial.

Funding statement:

This trial is funded by the National Institute for Health Research (NIHR201944) and sponsored by Manchester University NHS Foundation Trust (MFT). The funders of the study had no role in the study design or writing of the report. Dr Dapre is an NIHR sponsored GP academic clinical fellow.

Competing interests statement.

Michelle Harvie has co-authored three self-help books for the public to follow intermittent diets. All author proceeds are paid directly to the charity Prevent Breast Cancer (registered charity number 1109839) to fund breast cancer research.

For peer review only

Appendix

1.0 Patient Information Sheet (supplementary document 1)

2.0 Consent Form (supplementary document 2)

3.0 Self-monitoring schedule for capillary glucose and ketone monitoring (supplementary document 3)

4.0 Medical Management Protocols (supplementary document 4)

5.0 Intermittent Low Energy Diet Day Example (supplementary document 5)

6.0 End of Study Questionnaire (supplementary document 6)

LEGENDS

- <image 1>: Figure 1: Inclusion and exclusion criteria
- <image 2>: Figure 2: Participant flow through trial
- <image 3>: Figure 3: Schedule of assessments

For peer review only

Inclusion Criteria

- Pregnant women ≥18 years
- BMI of ≥27.5kg/m2 or a BMI ≥25 kg/m^2 in high risk minority ethnic group (i.e. South Asian, Black African, African Caribbean) and <50 kg/m2 at booking appointment (8-12 weeks' gestation)
- Newly diagnosed GDM according to local diagnostic criteria (fasting glucose ≥5.3mmol/l and/or 2-hour postprandial glucose ≥8.5mmol/l in a 75g OGTT) scheduled to receive first line diet and physical activity (best NHS care)
- 24-30 weeks' pregnant at screening appointment

Exclusion Criteria

- Pregestational type 1 or type 2 diabetes.
- Fasting glucose of ≥7 or 2-hour postprandial of ≥11 on OGTT (immediate intervention with medication would be required in this group of women)
- Current multiple pregnancy
- Maturity Onset Diabetes of the Young (MODY)
- Significant comorbid disease that in PI's opinion would preclude participation in the study e.g. chronic kidney disease, significant cardiac disease, significant history of disordered eating or severe psychological problems.
- Current participation in a GDM medication treatment trial
- People who are not capable of providing informed consent or adhering to the monitoring and safety protocols
- People who have previously had bariatric surgery for weight loss including gastric bypass and sleeve gastrectomy, and/or those prescribed weight loss medications (e.g. orlistat).
- Medications at the time of the OGTT that may interfere with results (e.g. high dose oral steroids, immunosuppressants)
- Previous history of intrauterine growth restriction
- Women who have lost more than 5% of their weight from booking appointment to screening appointment.

Figure 1: Inclusion and exclusion criteria

159x144mm (220 x 220 DPI)

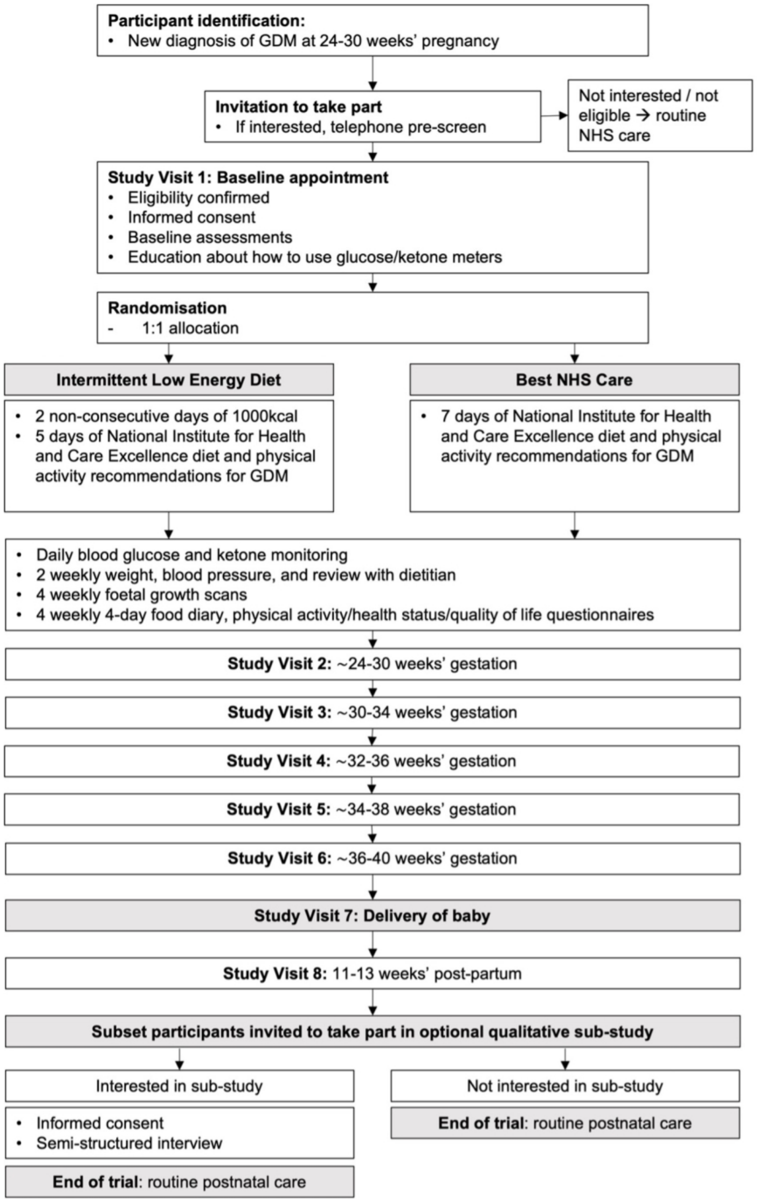

Figure 2: Participant flow through trial

154x245mm (220 x 220 DPI)

	Study Visit							
	1	2	3	4	5	6	7	8
Gestation (weeks)	~24-30	~24-30	~30-34	~32-36	~34-38	~36-40	delivery	11-13 post-partum
Eligibility confirmed	X							
Informed consent	X							
Randomisation	X							
Tailored dietitian review (face to face or remote)	X	X	X	X	X	X		X
Height	X							
Weight^	X	X	X	X	X	X	X	X
Blood Pressure^	X	X	X	X	X	X	X	X
Fasting blood sample*	X				X			X
Questionnaires‡	X		X		X			X
4-day food diary			X		X			X
Foetal growth scan	X		X		X			
Review of glucose and ketone measurements		X	X	X	X	X		
Neonatal measurements⁑							X	
Oral glucose tolerance test								X
Exit interview / end of study questionnaires								X
Invitation to optional qualitative sub-study$								X

^Frequency of assessment will be 2-4 weekly depending on whether appointment is face-to-face or virtual due to COVID-19 restrictions

*Fasting bloods: urea and electrolytes, liver function tests, bone profile, lipids, thyroid function tests, HbA1c, beta hydroxybutyrate, free fatty acids, full blood count, fasting glucose, insulin

‡Questionnaires: World Health Organisation Quality of Life (brief version), 36-Item Short Form Survey, International Physical Activity Questionnaire (short form), UK Diabetes and Diet Questionnaire

⁑Neonatal measurements include gestational age at delivery, mode of delivery, neonatal weight, cord blood glucose

$Sub-study involves semi-structured interviews exploring participants' thoughts and experiences of the trial

Figure 3: Schedule of assessments

154x236mm (72 x 72 DPI)

Manchester University NHS Foundation Trust

The University of Manchester

NIHR | National Institute for Health Research

Consultant Endocrinologist – Dr. Basil Issa
Research Dietitian – Dr. Michelle Harvie
Email: mft.middas.gdm@nhs.net
Tel: 07815987910

Nightingale Centre
Manchester University NHS Foundation Trust
Wythenshawe Hospital
Manchester
M23 9LT

MIDDAS-GDM

Manchester Intermittent Diet in Gestational Diabetes Acceptability Study

Participant information sheet

We would like to invite you to take part in a research study **that is testing two different diet programmes which aim to help people with gestational diabetes control their blood sugars.**

If you decide to take part:

- You will be assigned to one of two diet programmes for the duration of your pregnancy. One involves following the standard NHS healthy diet recommendations for pregnancy, and the other follows the standard NHS healthy diet for 5 days/week plus two non-consecutive calorie restricted days of 1,000 kcal per week (both groups will be encouraged to be physically active).

- You will be asked to attend your routine appointments at Wythenshawe or St Marys Hospital and will have fortnightly appointments until delivery of your baby (some appointments may be virtual depending on COVID-19 restrictions). You will be asked to attend the hospital for a blood test 12 weeks after having your baby.

- You will be supported by a diabetes specialist dietitian, midwife, consultant endocrinologist, and your obstetric team throughout the study to help manage your pregnancy and blood glucose safely.

- Throughout the study you will be asked to monitor your food intake via a smartphone/tablet app, or on paper if you prefer, and you will receive feedback on this during your dietary reviews. Comprehensive dietary advice and recipes will be provided.

- Throughout the study you will be asked to monitor your blood sugar using a blood sugar meter four times a day, and you will also be asked to monitor your ketone levels three times on two days of the week (ketones indicate how well your body is using sugar or fat as an energy source). You will be taught how to check your blood sugar and ketone levels.

- If you would like to take part, or you have any questions, then please contact mft.middas.gdm@nhs.net

For peer review only - http://bmjopen.bmj.com/site/about/guidelines.xhtml

This study is being carried out by a team of trained dietitians, doctors, nurses, midwives and researchers under the supervision of Dr. Basil Issa and Dr. Michelle Harvie at Wythenshawe and St Marys hospitals (Manchester University NHS Foundation Trust, MFT).

Before you decide if you would like to take part, it is important for you to understand why the research is being done and what taking part would involve for you. Please take your time to read the following information carefully. Discuss it with your friends, relatives, or GP if you wish to. Take time to consider whether or not you wish to take part.

Please ring the research team at the number at the top of the first page, or e-mail mft.middas.gdm@nhs.net if there is anything that is not clear, or if you would like more information. You can attend an information session about the diets and the study before agreeing to take part if you would like to.

Your participation in the study is entirely voluntary; you do not have to take part if you do not want to and you can opt out of the study at any time without giving a reason. Thank you for reading this information. We hope this research will be of interest to you.

Why are we doing this research?

Around 1 in 8 pregnant women can develop gestational diabetes. This condition causes risks to mother and baby from high blood sugar, high blood pressure, induced labours, caesarean-sections, and larger babies. Women often need medication to control blood sugar despite following recommended NHS healthy eating plans for pregnancy. Intermittent low-calorie diets (two non-consecutive days over the course of the week) improve blood sugar control and reduce the need for medication in patients with type 2 diabetes. We want to find out whether intermittent low-calorie diets might also improve blood sugar control in gestational diabetes and reduce the need for medication as it is a similar condition to type 2 diabetes.

What is the purpose of this research?

This study aims to assess the acceptability (to you) and safety of an intermittent low-calorie diet compared to the usual recommended NHS healthy eating and lifestyle plan for gestational diabetes. **A computer system will randomly allocate you to one of the two diets**. We want to find out which diet is most acceptable to women, whether there is any difference in the two diets' effect on blood sugar control, and any side effects experienced by women. The findings of this study will inform a larger study which will be designed to more closely compare the effect of the two diets on blood sugar control in women with gestational diabetes.

Why have I been asked to take part?

You have been invited to take part in this study because you have been diagnosed with gestational diabetes. We hope to recruit around 48 people to take part in this study.

For peer review only - http://bmjopen.bmj.com/site/about/guidelines.xhtml

Manchester University NHS Foundation Trust

The University of Manchester

NIHR | National Institute for Health Research

What happens if you agree to take part?

If you agree to take part you will be randomly allocated via a computer system to one of two diet and lifestyle programmes for the remaining weeks of your pregnancy.

Best NHS Care Healthy Diet programme

You will receive personalised advice from a specialist dietician. Recommendations will include increased fruit/vegetable intake, low glycaemic index starchy foods (i.e starchy foods which are slowly absorbed and take a while to raise your blood sugar level), reducing refined sugar, and having regular mealtimes. You will be advised how to design your diet to include the right amount of protein, fats, carbohydrates, and fibre, and will be given meal plans and recipes. You will also be advised to try to complete 150 minutes of moderate intensity exercise a week in addition to strength exercises on at least two days of the week.

Intermittent Low-Calorie Diet programme

If you are allocated to this group you will receive personalised advice to follow a low-calorie diet of 1,000 kcal on two non-consecutive days of the week and the NHS healthy diet on the other five days of the week. The 1,000 kcal days include a set number of portions of protein, carbohydrates and fat foods, fruits, vegetables and dairy/dairy alternatives typically including ~210g (7 oz) of lean protein foods and 3-4 portions of wholegrain carbohydrates, 5 portions of vegetables, 2 of fruit, and 3 of dairy or dairy alternatives and a small amount of healthy fat. You will also be advised to try to complete 150 minutes of moderate intensity exercise a week in addition to strength exercises on at least two days of the week.

Monitoring

You will have all of your usual routine antenatal appointments including checks on your weight, blood pressure, blood tests and ultrasound scans. Extra blood tests will be done as part of the study and these will be added on to samples taken during your routine blood tests.

You will be asked to monitor your blood sugar at home four times a day until your baby is born, and your ketone levels on two days of the week (you will be taught how to do this using a finger prick machine). The results will be recorded when you attend clinic.

We would like to collect a sample of blood from the umbilical cord at delivery to check blood sugar and insulin levels, and your baby's birthweight will be documented. Guidelines currently recommend that wherever possible the umbilical cord is clamped after 1-2 minutes; this is referred to as 'delayed cord clamping' and means that babies receive as much blood as possible from the placenta. This is normal practice. The cord blood sample will not affect your ability to have delayed cord clamping and will not cause any harm to your baby. It will not be possible, however, to delay clamping of the cord for so long that all blood has left the cord (the sample needs to be taken whilst there is still some blood left in the cord).

When babies are born to mothers with gestational diabetes it is normal that their birth weight is recorded and that their blood sugar is monitored for 12 hours following delivery; these results will be recorded by the research team.

For peer review only - http://bmjopen.bmj.com/site/about/guidelines.xhtml

Manchester University NHS Foundation Trust

MANCHESTER 1824 The University of Manchester

NIHR | National Institute for Health Research

You will be asked to attend an additional glucose tolerance test at the hospital 12 weeks after delivery to assess whether you have any residual diabetes (95% of women do not) and also to assess how sensitive your body is to insulin (an important risk factor for the development of diabetes in the future). You will be asked to attend at 9:00am having fasted (no food or drink apart from water) from midnight. A blood sample (around 10 mL/2 teaspoons) will be taken for glucose and insulin and you will be asked to drink a sugary drink with 75 grams of glucose. A further blood sample will be taken after 2 hours for glucose and insulin. You will need to remain in hospital during this time. The reason for this is to help us to understand whether there could be any difference in the body's ability to process sugar between the two diet groups, and also to find out whether any women still have signs of diabetes after pregnancy.

Any blood samples taken as part of the study will be identifiable only using your study identification number and will have none of your personal details. Part of the sample will be sent for immediate analysis and any remaining will be stored securely, accessible only by the research team. At the end of the study any left over samples will be disposed of in accordance with the Human Tissue Act (2004).

You will be asked to record your food intake via the Libro smartphone/tablet app or in a paper diary for four days during four weeks throughout the study. You will also be asked to complete three questionnaires to assess your wellbeing and level of physical activity in these weeks, and a final end of study questionnaire at the final appointment.

Ongoing support from a specialist team of healthcare professionals
Your specialist team includes a Consultant Endocrinologist, Consultant Obstetrician, diabetes specialist dietitian, midwives, and a GP trainee with a special interest in women's health. The specialist team work closely with the usual obstetric teams involved in your care. Reviews with the team will be either face to face when you attend clinic or remotely using video calls.

Mobile Applications and Glucose Meters
The study will use a smartphone application called 'Libro' to help you record information. Libro is a smartphone application which allows you to record your dietary intake during the study. We will ask you to record 4 days of food and drink intake during 4 weeks across the study. Your diaries will be viewed by your allocated dietitian who will provide personalised dietary feedback via the app. You are also free to record more days of your diet should you wish, which some people find helpful. If you do not want to use the mobile app you can use paper instead. You will be supported to set up and use the Nutritics Libro App at your appointments. You do not have to use the application to be part of the study.

Your blood sugar will be monitored using a glucose monitoring device which checks your blood sugar using a 'fingerprick' blood test. You will be shown how to do this yourself. With your permission the research team will make a note of your glucose readings at every visit, either by checking your glucose monitoring device, or by uploading your glucose meter readings onto the computer if you are using a mobile application.

The self-monitoring schedule is as follows:

For peer review only - http://bmjopen.bmj.com/site/about/guidelines.xhtml

Manchester University NHS Foundation Trust

MANCHESTER 1824 The University of Manchester

NIHR | National Institute for Health Research

Intermittent low-energy diet monitoring		Best NHS care monitoring	
Ketones (low kcal days)	Glucose	Ketones (2 days/wk)	Glucose
Fasting before breakfast the morning after each of the low calorie days	Fasting (morning)	Fasting before breakfast on 2 non-consecutive days / week	Fasting (morning)
1 hour post evening meal on each of the low calorie days	1hr post breakfast	1 hour post evening meal on 2 non consecutive days / week	1hr post breakfast
	1hr post lunch		1hr post lunch
	1hr post dinner		1hr post dinner

What should I do if my blood glucose or ketones are out of range?

Low Blood Sugar

You are advised to take 15-20g of 'rapid acting' carbohydrate if your blood glucose is <4 mmol/L). Examples include 170-225ml Lucozade Original (not Lucozade Sport), a small carton of fruit juice, 5-6 glucose tablets, 4/5 jelly babies, or a small tin of cola (150-200ml). You will need to repeat the treatment every 15 minutes until your blood glucose is ≥4 mmol/l.

The following table highlights when you need to consider an additional slower acting carbohydrate:

Situation	Acceptable slow acting carbohydrate
Less than 1 hour before the next meal	Try and avoid
1-2 hour before the next meal	10g (eg half of one of the items below)
More than 2 hours before the next meal	15-20g (eg slice of toast, piece of fruit, small bowl of cereal, glass of milk)

Raised Ketones

If your ketone levels are ≥1.0 mmol/L on a fasting sample:

- Drink 1L fluids and repeat ketone levels after 4 hours.
- If your ketone level has improved (<1.0mmol/L), no further action is required.
- If your ketone level has increased or remains the same, repeat your ketone level after 2 hours.
- If your ketone level is persistently increased, consume 40g carbohydrates (eg one bagel, bowl of cereal and a banana, small jacket potato), and repeat in 2 hours.
- Continue to do this until your ketone levels are <1.0mmol/L.

Make Immediate Contact with the research team if:

Blood glucose	• Your blood glucose is <3.0 mmol/l or you have symptoms requiring medical attention which are thought to be due to low blood glucose,

For peer review only - http://bmjopen.bmj.com/site/about/guidelines.xhtml

	• Your fasting blood glucose is >5.2 mmol/L on more than a quarter of your measurements on two days in a row,<br>• Your 1 hour post-meal blood glucose is >7.7 mmol/L on more than a quarter of your measurements on two days in a row
Ketones	• Your blood ketones are >1.0 mmol/L

Will I need medications?
If your blood sugars are found to be high despite following the recommended diet and lifestyle programmes you may be advised to start medication to help control your blood sugar. You will be advised on changes to your medications by a diabetes specialist nurse/diabetes midwife and also a Consultant Endocrinologist if required. This is usual practice regardless of whether you are taking part in the study.

What care will I receive after the study has stopped?
At the end of the study, you will be provided appropriate ongoing dietary advice from the study dietitian following your final glucose tolerance test to follow the NHS healthy eating and lifestyle plan. You will receive routine postnatal care from your GP, hospital team, and dietitian if required. You will be advised to see your GP for an annual blood test to check your blood sugar levels (this is routine care for women with gestational diabetes). Approximately 5% of women with gestational diabetes have residual diabetes after delivery. This will be identified from your glucose tolerance test/HbA1c; if this is the case you and your GP will be informed. Your GP will take over the management of your diabetes as per routine care outside the study.

Interview sub study
Women in this study may be invited to take part in an interview at the end of the study . You will be asked about your views and experiences on trying to follow your allocated diet programme. This interview can be arranged at a time that suits you, either at Wythenshawe or St Marys Hospital, at your home, or over the telephone. There is no obligation to take part in this interview study.

Frequently asked questions

Do I have to take part?
No, you do not have to take part if you do not wish to and your decision will not affect any standard of care you receive at Wythenshawe or St Marys hospitals (Manchester University NHS Foundation Trust, MFT).

What happens if I change my mind?
It is OK if you agree to take part in the study but later change your mind. You do not need to give a reason and it will not affect the standard of care you receive. The study team may also choose to withdraw you if it is necessary for your health or safety due to unexpected findings during the study. If you decide to withdraw from the study, or the study is stopped for any reason, you will be asked whether or not you are happy for us to keep the data that may have already been collected. If you do withdraw from the study you will continue to be cared for by your usual specialist diabetes and obstetric teams for the duration of your pregnancy. You will

For peer review only - http://bmjopen.bmj.com/site/about/guidelines.xhtml

still have the option of completing the end of study questionnaire and/or interview to provide feedback; this is very useful for the research team to help us understand potential reasons you may have chosen to withdraw from the study.

You will also have the option that if you withdraw, researchers may still collect relevant information about your pregnancy and/or gestational diabetes from your medical records within the 18-month study duration. This will be an option on the consent form.

Are there any benefits from taking part?
You will receive frequent personalised advice and support to follow two diet and lifestyle programmes which may help to control your blood sugar levels throughout your pregnancy. The information gained from this study will also help inform the future NHS care of patients with gestational diabetes.

Are there any risks from taking part?
Research has found that diets consisting of two low-calorie days a week are very low risk. Pregnant women will develop slightly higher levels of ketones when following low calorie diets than women who are not pregnant. Ketones are produced naturally by the body when the body uses fat stores for energy (i.e. when we follow a low calorie diet or haven't eaten enough because we are ill).

Some research suggests that very high levels of ketones throughout pregnancy may cause a higher risk of babies being slightly smaller than average. It is very unlikely that you will develop high levels of ketones by following this diet. You will be provided with a ketone meter and you will be asked to check your ketone levels before your lunch and evening meal on your low-calorie days, and the following morning, to make sure that your ketone levels are normal.

On your low-calorie days you may feel slightly more hungry, or you may experience other effects such as increased nausea, light headedness, or tiredness. It is important that you eat regularly throughout the day to reduce the risk of this happening. You will be asked to report any side effects of following the diet to the team at each appointment.

What happens if my baby or I become unwell during the study?
The safety of you and your baby are of utmost importance and remain our priority. In the instance that either of you become unwell your case will be reviewed by our specialist team and your suitability for continuing in the trial will be decided. Although it remains exceptionally rare, were you to experience the unexpected loss of your baby you will be withdrawn from the trial and supported by the dedicated specialist bereavement team at the hospital. Any information which has been collected as part of the trial will be stored securely and once we have finished the study we will keep some of the data so we can check the results. We will write our reports in a way that no-one can work out that you took part in the study.

What will happen to blood samples which are taken?
Some blood samples taken as part of the study will be sent to the laboratory immediately for analysis and any remaining will be stored securely for the duration of the study. Only your 'study ID' will be used – the samples will have none of your personal details on them. At the end of

For peer review only - http://bmjopen.bmj.com/site/about/guidelines.xhtml

NHS Manchester University NHS Foundation Trust

MANCHESTER 1824 The University of Manchester

NIHR | National Institute for Health Research

the study any remaining samples will be disposed of in accordance with the Human Tissue Act (2004).

What happens if something goes wrong?
If you have a concern about any aspect of this study, you should ask to speak with the lead researchers who will do their best to answer your questions (**Dr. Basil Issa** or **Dr. Michelle Harvie – via the study office – via michelle.harvie@manchester.ac.uk or telephone 0161 291 4410**). If you remain unhappy and wish to complain formally, you can do so through the NHS complaints procedure. Details can be obtained from the NHS Patient and Liaison Service (PALS) on **Tel:** 0161 276 8686 or contact the team by email pals@mft.nhs.uk.

The hospital is insured to carry out clinical research through the NHS Indemnity scheme. If something did go wrong and you were harmed or suffered deterioration in your health as a result of taking part in this study then you may have grounds for legal action or compensation.

Additional information about the study

Will my lifestyle be affected if I take part?
An essential aspect of this study is a change to your diet and physical activity patterns with support from a specialist team of healthcare professionals.

Payments
We are able to offer free parking at Wythenshawe/St Marys Hospitals for study visits and offer reimbursement for reasonable travel expenses (car, bus or tram) linked to visits for this study. There are no other payments for taking part.

Will my details be kept confidential?
Yes. The study team and any associated regulatory authorities follow strict ethical and legal guidance regarding participant confidentiality. Any information we have about you will be handled in confidence and will only be used for the purposes of this study. All data recorded will be coded and your name will remain anonymous.

During the study we will inform your GP via letter of your participation in the study and your ongoing results, including your weight, blood tests, any abnormal findings and any recommendations for treatment.

If you join the study, some relevant parts of your medical records may be looked at by authorised personnel at Wythenshawe or St Marys hospitals prior to starting the study. These records may also be looked at by an independent auditing body and regulatory authorities to check that the study is being carried out correctly. We will only access parts of your medical records that are relevant to this research and all information accessed will be kept strictly confidential.

How will we use information about you?

For peer review only - http://bmjopen.bmj.com/site/about/guidelines.xhtml

We will need to use information from you and from your medical records for this research project.

This information will include the following:

- Initials
- NHS number
- Name
- Contact details
- Medical History including test results

People will use this information to do the research or to check your records to make sure that the research is being done properly. People who do not need to know who you are will not be able to see your name or contact details. Your data will have a code number instead. We will keep all information about you safe and secure.

Once we have finished the study, we will keep some of the data so we can check the results. We will write our reports in a way that no-one can work out that you took part in the study. Other researchers from outside the Trust may ask to see this data for the purposes of furthering their research. We will only share this upon written request to the Trust. The external researchers will be asked to sign a Confidentiality Agreement before any data is shared.

What are your choices about how your information is used?
You can stop being part of the study at any time, without giving a reason, but we will keep information about you that we already have. If you choose to stop taking part in the study, we would like to continue collecting information about your health during pregnancy from your hospital records. If you do not want this to happen, tell us and we will stop. We need to manage your records in specific ways for the research to be reliable. This means that we won't be able to let you see or change the data we hold about you.

Where can you find out more about how your information is used?
You can find out more about how we use your information

- at https://research.cmft.nhs.uk/getting-involved/gdpr-and-research
- by asking one of the research team
- by sending an email to mft.middas.gdm@nhs.net or
- by ringing us on 07815987910

How will my details be used to access the Mobile Applications?
None of your personal details (other than the telephone number from which an application is downloaded) will be needed to access the mobile applications. Once you have given your consent to take part in the study you will be issued with a 'dummy' e-mail and password under a pseudonym (fake name). Only the research team will know the dummy e-mail address you have been assigned to, in order to be able to review your data. The application will not contain your identifiable data. If you choose to use a mobile application to monitor your blood sugar

For peer review only - http://bmjopen.bmj.com/site/about/guidelines.xhtml

levels the relevant terms of service for the app and the app developers privacy policy will apply. It will be your responsibility to read and understand these prior to download.

Will my insurance be affected if I take part in this study?
It is unlikely that your insurance premiums will be affected by participation in this study as the study has the potential to improve your diabetic control and reduce your risk of ill health. However, if you are at all concerned, then we advise that you contact your insurers and seek expert advice before agreeing to participate.

Who has reviewed this study?
Research in the NHS is looked at by an independent group of people called a Research Ethics Committee (REC). The REC is made up of experts, non-experts and members of the general public. Together they review research applications to ensure your safety, rights, wellbeing and dignity are protected at all times. This study has been reviewed and given favourable opinion by REC.

What will happen to the study results?
It is intended that the results of this study will be presented at conferences and published in medical journals so that we can explain to the medical community what our research results have shown. To do this our study information is double-checked by other professionals in research and healthcare. There is a possibility that the study and its results may be publicised for example on radio, television, magazines, books and websites. **You will not be identified in any publicity, reports or publication arising from this study.** If you would like a general summary of the results of the study you can select this on the consent form or please contact the research team.

Who is organising and funding the research?
Researchers from Wythenshawe hospital, have designed this study and will be carrying out this research. This study has been funded by the National Institute of Health and Research.

Further information and contact details
For further information about this study, please contact **mft.middas.gdm@nhs.net** **or 07815987910**

Thank you for taking the time to read this information sheet.
We hope it has been of interest to you.

For peer review only - http://bmjopen.bmj.com/site/about/guidelines.xhtml

Manchester University NHS Foundation Trust

The University of Manchester

NIHR | National Institute for Health Research

Participant pathway for the study

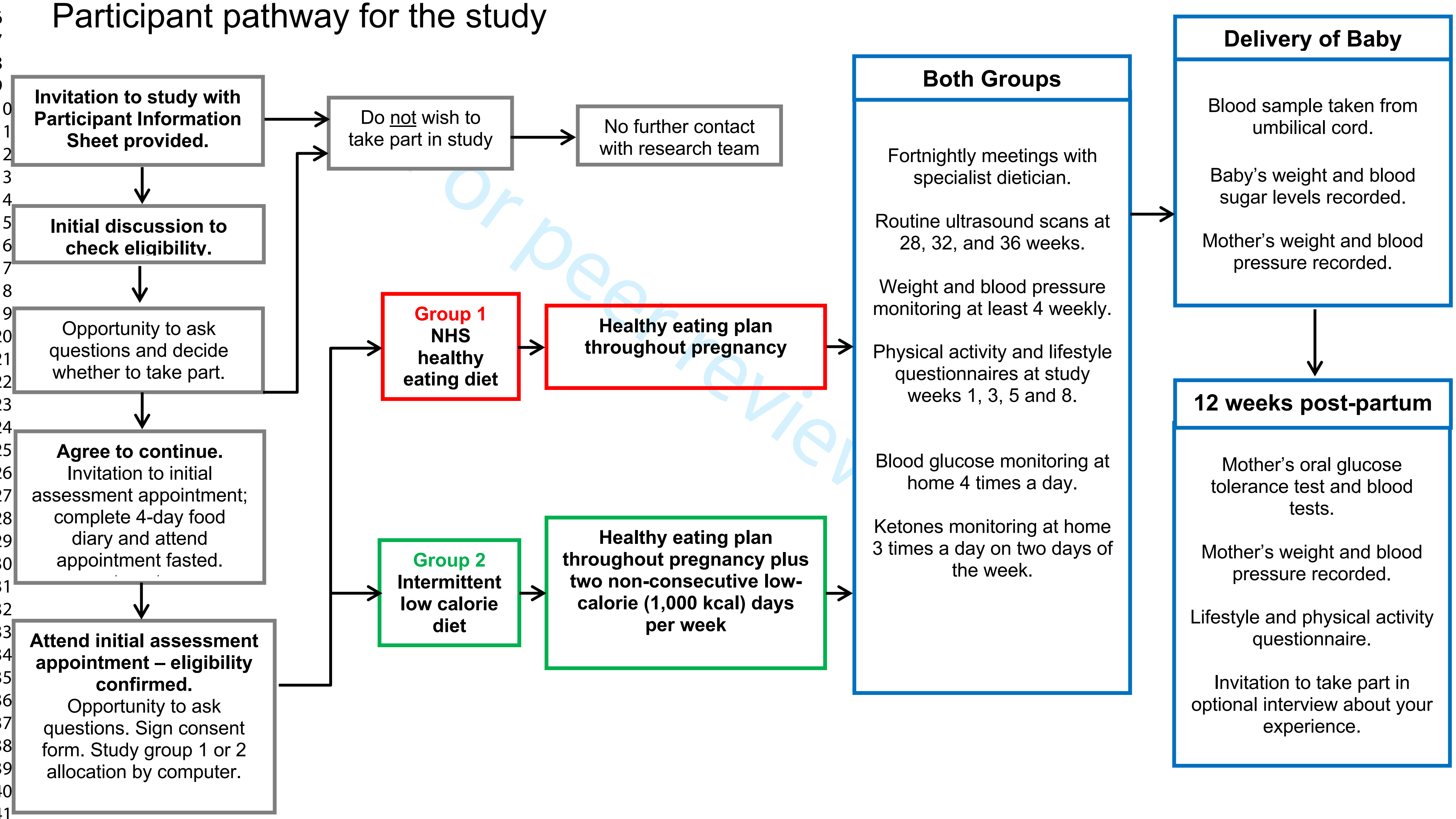

For peer review only - http://bmjopen.bmj.com/site/about/guidelines.xhtml

NHS Manchester University NHS Foundation Trust

MANCHESTER 1824 The University of Manchester

NIHR | National Institute for Health Research

Consultant Endocrinologist – Dr. Basil Issa
Tel: 0161 291 7070
Research Dietitian – Dr. Michelle Harvie
Tel: 07815987910
Email: mft.middas.gdm@nhs.net

Nightingale Centre
Manchester University NHS Foundation Trust
Wythenshawe Hospital
Manchester
M23 9LT

MIDDAS-GDM

Manchester Intermittent Diet in Gestational Diabetes Acceptability Study

Participant Informed Consent Form

Participant Identification Number:

Please **initial** box

1.	I confirm that I have read and understand the participant information sheet (version 3.0) for the above study. I have had the opportunity to consider the information and ask questions, and have had these answered satisfactorily.	☐
2.	I understand that my participation is voluntary and that I am free to withdraw at any time, without giving any reason, without my medical care or legal rights being affected.	☐
3.	I understand that relevant sections of my medical notes and data collected during the study may be looked at by individuals from Manchester University NHS Foundation Trust and regulatory authorities, where it is relevant to my taking part in the research. I give permission for these individuals to have access to my records.	☐
4.	I consent to the collection of blood samples to be collected as described in the participant information sheet.	☐
5.	I consent to the collection of cord blood to be collected at the time of delivery as described in the participant information sheet.	☐
6.	I understand that the information collected about me will be used to support other research in the future and may be shared anonymously with other researchers.	☐
7.	I agree that my blood sugar readings can be recorded by the study team.	☐
8.	I agree to my GP being informed of my participation in this study and changes to my weight, body measurements, blood results, questionnaire results and medications as required.	☐

For peer review only - http://bmjopen.bmj.com/site/about/guidelines.xhtml

Manchester University NHS Foundation Trust

MANCHESTER 1824 The University of Manchester

NIHR | National Institute for Health Research

9. I understand that to use the mobile applications as described in the Patient Information Sheet, I will need to provide my personal mobile number to be able to access the app. ☐

10. I understand that the information I provide to mobile applications as described in the Patient Information Sheet will be treated in line with the relevant terms of service and the app developers privacy policy at the time of downloading the application. ☐

11. I have informed the study team of any health issues, including those which may affect my ability to follow the diet, and I will inform the study team of any unusual symptoms that occur during the diet. I will inform the study team of changes to my health status during the study. ☐

12. I have informed the study team of any health issues, including those which may affect my ability to exercise, and I will inform the study team of changes to my health status during the study. ☐

13. I consent to the storage of personal information (including electronic) for the purposes of this study. I understand that any information that could identify me will be kept strictly confidential and that no personal information will be included in the study report or other publication. ☐

14. I agree to take part in the above study. ☐

15. I agree that relevant information about my pregnancy and/or gestational diabetes can be obtained from my medical records within the 18-month study duration if I withdraw from the study early. ☐

16. I am aware that my non-identifiable trial data may be shared with other researchers for the purposes of research. ☐

Optional (delete as appropriate)

17. I agree to be approached to take part in sub-study 1 (interview study), and understand that I will be approached to take part in the sub-study regardless of whether I withdraw from the main study YES/NO

18. I would like to receive a summary of the final study results YES/NO

19. I agree to be contacted regarding future research opportunities YES/NO

My preferred contact (*please tick and include email if preferred*)

Do not contact ☐ Post ☐ Email ☐ ____________________

For peer review only - http://bmjopen.bmj.com/site/about/guidelines.xhtml

Manchester University
NHS Foundation Trust

MANCHESTER 1824
The University of Manchester

NIHR | National Institute for Health Research

Signatures

…………………………..………....	…………………..	……………..………………
Name of participant	Date	Signature
……………………………………....	……………………	……………..……………
Name of person taking consent	Date	Signature

When completed: 1 for participant; 1 for patient file; 1 for medical notes; 1 for GP; 1 (original) for site file

For peer review only - http://bmjopen.bmj.com/site/about/guidelines.xhtml

Self-monitoring schedule for capillary glucose and ketone monitoring

ILED		Best NHS Care	
**Ketones (low kcal days)**	**Glucose**	**Ketones (2 days/wk)**	**Glucose**
Fasting before breakfast the morning after each of the low-energy days	Fasting (morning)	Fasting before breakfast on 2 non-consecutive days / week	Fasting (morning)
1 hour post evening meal on each of the low-energy days	1hr post breakfast	1 hour post evening meal on 2 non-consecutive days / week	1hr post breakfast
	1hr post lunch		1hr post lunch
	1hr post dinner		1hr post dinner

peer review only

Medical Management Protocols

Hypoglycaemia

Participants will be advised to take 15-20g of rapid acting carbohydrate in the event of hypoglycaemia, (defined as blood glucose <4 mmol/L) which is anticipated to raise blood glucose by 3 mmol/L. Examples of rapid acting carbohydrate include 170-225ml Lucozade Original (not Lucozade Sport), a small carton of fruit juice, 5-6 glucose tablets, 4/5 jelly babies, or a small tin of cola (150-200ml). Participants will be advised to repeat the treatment every 15 minutes until blood glucose is ≥4 mmol/l. The following table highlights when participants should consider taking additional follow-up slower acting carbohydrate:

Situation	Acceptable slow acting carbohydrate
Less than 1 hour before the next meal	Try and avoid
1-2 hour before the next meal	10g
More than 2 hours before the next meal	15-20g

Ketonaemia

Ketone levels ≥1.0 mmol/L on a fasting sample:

- Drink 1L fluids and repeat ketone levels after 4 hours.
- If ketone level has improved (<1.0mmol/L), no further action required.
- If ketone level has increased or remains the same, repeat ketone level after 2 hours.
- If ketone level is persistently increased, consume 40g carbohydrates and repeat in 2 hours.
- Continue to do this until ketone levels <1.0mmol/L.

If a participant experiences >2 episodes of the above throughout the course of the study their notes will be reviewed by the PI and their suitability for remaining in the trial will be assessed.

For peer review only

Guidance for the introduction of diabetes medication (week 24-delivery)

Diabetes medication will be introduced according to the following protocol:

- If ≥25% fasting blood glucose readings are >5 mmol/l and/or ≥25% of 1 hour postprandial glucose readings are >7 mmol/l in a 7 day period: commence Metformin MR 500 mg daily to be increased every 3 days by 500 mg to 1 gram BD if tolerated.

- If after reaching optimal or maximum tolerated dose Metformin ≥25% fasting blood glucose readings are >5 mmol/l in a 7-day period: commence bedtime isophane insulin 4 units and uptitrate the dose by 2 units every 3 days aiming for a fasting glucose of ≤5 mmol/l,

- and/or ≥25% of 1 hour postprandial glucose readings are >7 mmol/l in a 7 day period: commence prandial fast acting insulin analogue (Humalog or Novorapid) 2-4 units with the relevant meal. Uptitrate the dose by 2 units every 3 days aiming for a 1 hour postprandial glucose of ≤7 mmol/l.

- Medication adjustment will be made in accordance with the above guidance.

For peer review only

Example of 1 day meal plan for Diet Day

The diet days aim to limit the calories to 1000 calories per day. You are aiming to include 2 (not consecutive) diet days each week. The other 5 days, follow the Mediterranean diet as described earlier. To keep the calories to 1000, the diet day will look like this:

Mixed diet		Vegetarian/ vegan diet
**4**	Carbohydrate portions	3
**6**	Protein portions	7
**5**	Vegetable portions	5
**2**	Fruit portions	2
**3**	Dairy portions	3
**1**	Fat portions	1

Below are some examples of meals that can be used to help you follow a 1000 calorie diet.. There are options for a mixed diet or vegan or vegetarian options, if you feel you wanted to try meat free days. Filling up on vegetables will make you feel less hungry

For peer review only

Mixed diet options

Breakfast	Portion	Dairy	Protein	Carb	Veg	Fruit	Fat
Grilled lean bacon	1 rasher	0	1	0	0	0	0
Grilled tomatoes	7 cherry tomatoes	0	0	0	1	0	0
Tea/ coffee	1 mug	0	0	0	0	0	0
Midmorning							
Diet or natural yogurt	1 small carton	1	0	0	0	0	0
Lunch							
Wholegrain bread	2 medium slices	0	0	2	0	0	0
Tuna	⅓ of a 120g can	0	1	0	0	0	0
Green salad	Cereal bowl full / 80 g with oil-free dressing	0	0	0	1	0	0
Satsumas	2	0	0	0	0	1	0
Mid afternoon							
Low fat cheese	30g / match box size	1	0	0	0	0	0
Apple slices	I medium apple (80g)	0	0	0	0	1	0
Tea/ coffee		0	0	0	0	0	0
Evening							
Vegetable rice	4 tablespoons cooked rice 160g of mix vegetables	0	0	2	2	0	0
Chicken curry	90g /average chicken breast (no skin) & ½ can tomatoes, 1 desertspoon oil	0	3	0	1	0	1
Bedtime							
Low fat houmous	1 level tablespoon	0	1	0	0	0	0
Pepper sticks	½ red pepper	0	0	0	1	0	0
Milk	1 small glass	1	0	0	0	0	0
**Total portions a day**		**3 portions**	**6 portions**	**4 portions**	**5 portions**	**2 portions**	**1 portion**

For peer review only

Vegetarian option

Breakfast		Dairy	Protein	Carb	Veg	Fruit	Fat
Egg	2 poached	0	2	0	0	0	0
Mushrooms	2 cupped handfuls / 80g	0	0	0	1	0	0
Tea/ coffee	1 mug	0	0	0	0	0	0
Midmorning							
Cheddar cheese	1 match box size / 30g	1	0	0	0	0	0
Cucumber	Sliced handful	0	0	0	1	0	0
Lunch							
Baked beans	2 tablespoons	0	1	0	0	0	0
Seeded bread toasted	1 medium sliced	0	0	1	0	0	0
Blueberries	1 handful	0	0	0	0	1	0
Mid afternoon							
Meat free ham	2 slice small	0	1	0	0	0	0
Pepper	½ sliced	0	0	0	1	0	0
Avocado	¼	0	0	0	0	0	1
Tea/ coffee	1 mug	0	0	0	0	0	0
Evening							
Vegetarian sausage casserole Jacket potato (100g)	1 grilled sausage 2 cereal bowls vegetables 1 ½ egg sized (100 g)	0	2	1	2	0	0
Bedtime							
Pear	1 medium	0	0	0	0	1	0
Low fat cream cheese	1 tablespoon	1	0	0	0	0	0
Whole wheat cracker	2 biscuits	0	0	1	0	0	0
Milk	1 small glass	1	1	0	0	0	0
**Total portions a day**		**3 portions**	**7 portions**	**3 portions**	**5 portions**	**2 portions**	**1 portions**

For peer review only

Vegan options

Breakfast		Dairy equivalent	Protein	Carb	Veg	Fruit	Fat
Branflakes	3 tablespoons	0	0	1	0	0	0
Milk- soya	200 ml	1	0	0	0	0	0
Tea/ coffee	1 mug	0	0	0	0	0	0
Midmorning							
Soya yogurt	3 tablespoons	1	0	0	0	0	0
Lunch							
Kidney bean & Vegetable chilli<br><br>Wholemeal pitta	3 tablespoons of beans 60g with 1 cereal bowl mixed vegetables & 1/2 can chopped tomatoes<br>½ pitta	0	2	1	2	0	0
Banana	1 medium	0	0	0	0	1	0
Mid afternoon							
Low fat hummus	2 level tablespoon	0	2	0	0	0	0
1 carrot	I medium carrot (80g)	0	0	0	1	0	0
Tea/ coffee	1 mug	0	0	0	0	0	0
Evening							
Quinoa	2 tablespoon cooked	0	0	1	0	0	0
Tofu	4 matchbox	0	2	0	0	0	0
Mixed salad with edamame beans	2 x Cereal bowl full with oil free dressing & 1 tablespoons of edamame	0	1	0	2	0	0
Bedtime							
Peanut butter	1 heaped teaspoon	0	0	0	0	0	1
Apple	1 medium sliced	0	0	0	0	1	0
Milk	1 small glass	1	0	0	0	0	0
**Total portions a day**		**3 portions**	**7 portions**	**2 portions**	**5 portions**	**2 portions**	**1 portions**

For peer review only

To help with estimation of portions the following tables outline weight and measures of the different food groups. Where possible household measures are given to make things a little easier. Use these to help you plan your 2 days in the week of 1000 calories.

**Carbohydrate**<br>**4 portions - mixed diet**<br>**3 portions - vegan/vegetarian**	**Equal to**
Wholewheat or oat breakfast cereal, e.g. wholewheat biscuit, malted wholewheat squares, Grapenuts, bran flakes, fruit & fibre	24g or 3 tablespoons or<br>1 whole wheat biscuit
Porridge oats or no-added sugar muesli	20g or 1 heaped tablespoon
Wholegrain, wholemeal, rye, granary bread	36g or medium slice of bread (other than rye), 1½ slices of rye, or ½ roll
Wholemeal or multigrain pitta bread or tortilla wrap, chapatti made without fat	60g or 1x 8” tortilla<br>or 1 standard pitta<br>or small thin chapatti
Rye crispbread, crackers, oak cakes	22g or 2 crispbreads/ 2 oatcakes
Wholegrain rice cake	16g or 2 rice cakes
Wholewheat pasta or rice - cooked amount<br><br>Cous cous, Bulgar wheat, Quinoa, Pearl barley	1 tablespoon uncooked 2 tablespoons cooked<br><br>30g- raw weight or 60g cooked
Lasagne (wholemeal if possible)	20g raw weight or<br>1 large sheet or 1½ smaller sheets
Noodles (wholemeal if possible)	25g raw weight or ½ block/nest
Baked or boiled potato (in skin), cassava, sweet potato	1½ egg sized potatoes or<br>100g raw weight
Wholemeal pizza base (topping is from other food groups)	35g or $^{1}/_{6}$ of thin 10” pizza base
Unsweetened popcorn	20g or 2 handfuls

For peer review only

**Protein**<br>**6 portions – mixed diet**<br>**7 portions – vegan/vegetarian**	**Equal to**
Fresh or smoked white fish (e.g. haddock or cod)	60g or 2oz<br>2 fish finger size
Seafood, e.g. prawns, mussels, crab	45g or 1½oz
Canned tuna or salmon in brine or spring water	45g or 1½oz<br>⅓ standard tin (120g)
Oily fish (fresh or tinned in tomato sauce or olive oil - drained), e.g. mackerel, sardines, salmon, fresh tuna, kippers, smoked salmon or trout	30g or 1oz or<br>¼ standard tin (120g) or<br>¼ fillet of salmon
Chicken, turkey, duck, pheasant (cooked without skin)<br>Lean beef, pork, lamb, rabbit, venison, offal (fat removed)<br>**Quorn fillets, steak, mince or pieces**<br>**Vegetarian mince frozen**	30g or 1oz or<br>1 slice size of playing card
Lean grilled bacon<br>**Quorn ham**	25g or ¾oz or<br>1 rasher
Lean ham<br>**Quorn bacon rashers (not slices)**	30g or 1oz or<br>1 medium, 2 small or 4 wafer thin slices
Eggs	60 g or 2 oz or<br>1 egg
Tofu	50g or $1^2/_3$ oz or<br>Size of 2 match boxes
Tempeh	25 g or 1 oz or<br>Size of 1 match box
Baked beans (reduced sugar)	60 g or 2 oz or<br>2 tablespoons
Lentils, chickpeas and kidney beans, mung beans, black eye beans, puy lentils, toor dahl, urad dahl,<br>Raw weight<br><br>Cooked or tinned weight	20g or ⅔ oz or<br>1 tablespoon raw<br><br>65g or 2oz or<br>1½ tablespoons cooked /tinned or 1 cupped handful
Soya beans (frozen or cooked) or edamame beans	30g or 1oz or<br>1 tablespoon
Vegetarian sausage	25g or ¾ oz or ½ sausage
Textured vegetable protein (TVP)	10g or ⅓ oz uncooked or<br>1 heaped tablespoon uncooked
Low fat hummus	30g or 1oz or<br>1 level tablespoon

Vegetables – min 5 portions<br>1 portion = 80g or 2⅔oz	1 portion is equal to
Asparagus, Aubergines, Broccoli, Brussel sprouts, Carrots, Cabbage, Cauliflower, Chinese leaves, Courgettes, Cucumber, Curly kale, Green beans, Lettuce (mixed leaves), Mange tout, Methi, Mushrooms, Okra, Pak choi, Peas, Sugar snap, Spinach, Spring greens cooked, Sweetcorn, Tomatoes, Watercress fresh	80g or 2 ⅔ oz or 2 spears of broccoli, 8 cauliflower florets. 3 heaped tablespoons of vegetables or large cereal bowl of salad.

Fruit - 2 portions.<br>1 portion = 80g or 2⅔oz (30g or 1oz dried fruits)	1 portion is equal to
Berries (e.g. blackberries, blueberries, redcurrants, raspberries, strawberries)<br>Cherries or grapes	80g or 2⅔oz<br>1 handful
Grapefruit, guava and mango	80g or 2⅔oz or<br>½ a whole fruit
Large fruit (e.g. melon, pineapple, papaya)	80g or 2⅔oz or<br>1 medium slice
Medium fruits (e.g. apple, pear, nectarine, orange, peach, banana, pomegranate)	80g or 2⅔oz<br>1 fruit
Small fruit (e.g. fresh apricots, kiwi, clementine, passion fruit, plums)	80g or 2⅔oz or<br>2 fruits
Any stewed fruit—unsweetened or with calorie-free sweetener e.g. apple, rhubarb	80g or 2⅔oz or<br>3 tablespoons
Kumquats, lychees, physalis	5 fruits
Dried fruits (raisins, currants, apricots)	30g or 1oz or<br>1 tablespoon

Milk and dairy foods - 3 portions	Equal to
Milk (semi-skimmed or skimmed)<br>Alternative ‘milks’ with added calcium, e.g. soya **	⅓ pint or 200ml or<br>1 small glass
Diet yoghurts, Low fat/fat-free Greek or Greek Style or natural yoghurts, fromage frais or plain soya yoghurt, high protein yogurt	120-150g or 4-5oz or<br>1 small pot or<br>3 tablespoons
Whole milk natural yogurt	80g or 1 ⅔ oz or<br>2 tablespoons
Cottage cheese	75g or 1½oz or<br>¼ pot, 2 tablespoons
Cream cheese (light or extra light)	30g or 1oz or<br>1 tablespoon
Lower fat hard cheeses e.g.:<br>Reduced fat cheddar, Edam,<br>Bavarian smoked, feta, ricotta, mozzarella, reduced fat halloumi, paneer made from semi-skimmed milk	30g or 1oz or Matchbox size<br>No more than 180g or 6oz a week

** we recommend soya milk as coconut, oat and almond milks are lower in protein and calcium

For peer review only

**Fat – 2 portions only**	**Equal to**
Margarine or low-fat spread (avoid the buttery types)<br>Olive oil or other oil<br>Oil based dressing<br>Pesto<br>Mayonnaise	8g or 1 teaspoon<br>1 dessertspoon of oil
Seeds (e.g. linseed, pumpkin, sunflower, sesame, chia, hemp)	7g or 1 dessertspoon
Unsalted or salted or dry roasted nuts	7g or 1 dessertspoon<br>3 walnut halves, 3 Brazil,<br>4 almonds, 8 peanuts,<br>10 cashews or pistachios
Olives	50g or 10 olives
Low fat mayonnaise, Curry paste or Harissa paste	15g or ½ oz or 1 tablespoon
Peanut butter (without palm oil)	11g or ⅓ oz or<br>1 heaped teaspoon
Cocoa powder	12 g or ⅓ oz or<br>2 heaped teaspoons
Avocado	40g or 1⅓ oz or<br>1/4 of an average pear
Low fat guacamole	40g or 1⅓ oz or 2 tablespoons

For peer review only

NHS
Manchester University
NHS Foundation Trust

MIDDAS-GDM End of Study Questionnaire

Thank you for taking part in the MIDDAS-GDM Study.

This is one of the first studies of its kind. We hope to learn as much as possible from this study, in particular the views of people who have taken part. We are inviting you to provide your views on different aspects of the study and following the diet, and how we can improve our programmes and research studies in future.

Please complete the following questions and return this questionnaire to the MIDDAS-GDM study team in the envelope provided. If there is anything else you would like to say about your experiences of the study, please use the section at the end. Your answers to the questions below will remain anonymous.

1. What were your reasons for joining the study?

………

………

………

………

………

2. How satisfied were you with study overall (recruitment, appointments etc)? *(circle)*

1	2	3	4	5	6	7	8	9	10
Not at all satisfied		Slightly satisfied		Quite satisfied		Very satisfied			Extremely satisfied

Comments:……………………………………………………………………………………………………

………

………

Study Appointments

3. You were asked to attend ……….. additional face to face appointments at the hospital by the study team *(please fill in the number)*

For peer review only - http://bmjopen.bmj.com/site/about/guidelines.xhtml

NHS
Manchester University
NHS Foundation Trust

4. **You were asked to attend ……….. additional virtual (i.e. video call or telephone) appointments by the study team** *(please fill in the number)*

5. **How do you feel about the number of additional appointments you were asked to attend?** *(tick)*

☐ I was happy with the number of appointments
☐ I would have preferred fewer face to face appointments
☐ I would have preferred more face to face appointments
☐ I would have preferred fewer virtual appointments
☐ I would have preferred more virtual appointments

Comments:……………………………………………………………………………………………
……………………………………………………………………………………………………

6. **How do you feel about virtual (i.e. video call or telephone) appointments?**

☐ I prefer virtual appointments to face to face appointments (please explain why below)
☐ I prefer face to face appointments to virtual appointments (please explain why below)

Comments:……………………………………………………………………………………………
……………………………………………………………………………………………………
……………………………………………………………………………………………………
……………………………………………………………………………………………………

For peer review only

Diet

7. **Which diet were you asked to follow? (*please tick*)**

☐ Best NHS Care (i.e. increased fruit/vegetable intake, low-GI foods, reduction in free sugars, regular meals)
☐ Intermittent low energy diet (5 days of the best NHS care diet plus 2 non-consecutive days of 1000 kcal each week)

8. **Was the diet easy to follow?**

1	2	3	4	5	6	7	8	9	10
Not at all		Slightly		Moderately		Very			Extremely

For peer review only - http://bmjopen.bmj.com/site/about/guidelines.xhtml

NHS Manchester University NHS Foundation Trust

Comments:……………………………………………………………………………………………

……………………………………………………………………………………………………

9. Did you enjoy following the diet plan?

1	2	3	4	5	6	7	8	9	10
Not at all		Slightly		Moderately		Very			Extremely

Comments:……………………………………………………………………………………………

……………………………………………………………………………………………………

10. Would you make any changes to the written information (i.e. diet booklets, recipes) you were given on how to follow the diet plan?

☐ Yes

☐ No

If yes, what changes would you make?

Comments:……………………………………………………………………………………………

……………………………………………………………………………………………………

11. How was your first appointment with the dietitian? *(tick all that apply)***?**

☐ The amount of information I received was OK

☐ The amount of information I received was too little

☐ The amount of information I received was too much

☐ I was happy with the advice I received

☐ The advice I received could be improved (specify below)

Comments:……………………………………………………………………………………………

……………………………………………………………………………………………………

For peer review only - http://bmjopen.bmj.com/site/about/guidelines.xhtml

Manchester University
NHS Foundation Trust

12. Would you make any changes to the reviews (calls/face to face appointments) you had with the dietitian during your pregnancy ?

☐ Yes

☐ No

Comments:……………………………………………………………………………………………

……

13. How useful was your final appointment with the dietitian at 12 weeks post-delivery?

1	2	3	4	5	6	7	8	9	10
Not at all		Slightly		Quite		Very			Extremely

Comments:……………………………………………………………………………………………

……

14. Did you feel confident to exercise whilst on the diet plan?

1	2	3	4	5	6	7	8	9	10
Not at all confident		Slightly confident		Quite confident		Very confident			Extremely confident

Comments:……………………………………………………………………………………………

……

For peer review only

Additional support

15. Would any of the following have been useful & if so how often?

	No	Yes	Preferred method of contact	How often
Additional support from dietitian	☐	☐	Face to face / phone	
Additional support from the midwife	☐	☐	Face to face / phone	

For peer review only - http://bmjopen.bmj.com/site/about/guidelines.xhtml

Manchester University NHS Foundation Trust

Additional support from the doctors in the clinic	☐	☐	Face to face / phone	
More contact with other women in the study following the diets	☐	☐	Face to face / phone	
Other, please specify:				

16. Did you receive any support outside of the study team help to keep you on track as you progressed through the study?

☐ No

☐ Yes

If yes, what support did you receive?

…………………………………………………………………………………………………

…………………………………………………………………………………………………

Record keeping

17. How did you find the finger prick testing requirements on the study? *(tick all those that apply)*

☐ Challenging but on the whole achievable

☐ Challenging and not achievable

☐ Not challenging at all

☐ I felt it was necessary to test this often to ensure my safety

☐ I felt it was unnecessary to test this often to ensure my safety

Comments:………………………………………………………………………………………

…………………………………………………………………………………………………

18. How did you find the ketone testing requirements on the study? *(tick all those that apply)*

☐ Challenging but on the whole achievable

☐ Challenging and not achievable

For peer review only

For peer review only - http://bmjopen.bmj.com/site/about/guidelines.xhtml

Manchester University
NHS Foundation Trust

☐ Not challenging at all

☐ I felt it was necessary to test this often to ensure my safety

☐ I felt it was unnecessary to test this often to ensure my safety

Comments:…………………………………………………………………………………………

…………………………………………………………………………………………………...............

19. How did you find using Diasend software?

☐ Straightforward

☐ Challenging but on the whole achievable

☐ Challenging and not achievable

☐ I felt uncomfortable using computer software to keep track of my medical details

☐ I felt comfortable using computer software to keep track of my medical details

Comments:…………………………………………………………………………………………

…………………………………………………………………………………………………...............

20. How did you find completing the food diary during the study? *(tick all those that apply)*

☐ Challenging but on the whole achievable

☐ Challenging and not achievable

☐ Not challenging at all

Comments:…………………………………………………………………………………………

…………………………………………………………………………………………………...............

21. How did you find the physical activity questionnaires on the study? *(tick all those that apply)*

☐ Challenging but on the whole achievable

☐ Challenging and not achievable

☐ Not challenging at all

Comments:…………………………………………………………………………………………

…………………………………………………………………………………………………...............

For peer review only - http://bmjopen.bmj.com/site/about/guidelines.xhtml

NHS
Manchester University
NHS Foundation Trust

22. How did you find the quality of life questionnaires on the study? *(tick all those that apply)*

☐ Challenging but on the whole achievable

☐ Challenging and not achievable

☐ Not challenging at all

Comments:……………………………………………………………………………………………

……

Libro® app

23. Did you use the Libro® app?

☐ Yes

☐ No (please move to question 25)

24. Did you find the App helpful?

1	2	3	4	5	6	7	8	9	10
Not at all		Slightly		Moderately		Very			Extremely

Comments……………………………………………………………………………………………

……

25. What did you like about the App?

……

……

……

26. What did you dislike about the App and could be improved?

……

……

27. If you didn't use the app what were the reasons for this? *(tick all that apply)*

☐ Would prefer not to say ☐ Already using other health apps

For peer review only - http://bmjopen.bmj.com/site/about/guidelines.xhtml

Manchester University
NHS Foundation Trust

☐ Don't like using apps in general
☐ Not user friendly
☐ Labour intensive / time consuming
☐ Prefer to use pen and paper
☐ Find mobile devices challenging
☐ Lack of regular internet access
☐ Other (provide details below)

……………………………………………………………………………………………………

……………………………………………………………………………………………………

……………………………………………………………………………………………………

Diasend Software

28. Did you find the Diasend software helpful?

1	2	3	4	5	6	7	8	9	10
Not at all		Slightly		Moderately		Very			Extremely

Comments……………………………………………………………………………………………

……………………………………………………………………………………………………

29. What did you like about the Diasend software?

……………………………………………………………………………………………………

……………………………………………………………………………………………………

30. What did you dislike about the Diasend software and could be improved?

……………………………………………………………………………………………………

……………………………………………………………………………………………………

Study Improvements

31. What did you enjoy most about the study?

……………………………………………………………………………………………………

……………………………………………………………………………………………………

……………………………………………………………………………………………………

……………………………………………………………………………………………………

For peer review only

For peer review only - http://bmjopen.bmj.com/site/about/guidelines.xhtml

Manchester University
NHS Foundation Trust

32. What did you enjoy least about the study and could be improved?

……………………………………………………………………………………………………
……………………………………………………………………………………………………
……………………………………………………………………………………………………
……………………………………………………………………………………………………
……………………………………………………………………………………………………

<u>Any other comments about the study</u>

……………………………………………………………………………………………………
……………………………………………………………………………………………………
……………………………………………………………………………………………………
……………………………………………………………………………………………………
……………………………………………………………………………………………………
……………………………………………………………………………………………………
……………………………………………………………………………………………………
……………………………………………………………………………………………………

For peer review only

Thank you for completing this questionnaire, please return to:

MIDDAS-GDM Study Team, Nightingale Centre,

Wythenshawe Hospital, Manchester, M23 9LT

For peer review only - http://bmjopen.bmj.com/site/about/guidelines.xhtml

TIDieR
Template for Intervention Description and Replication

The TIDieR (Template for Intervention Description and Replication) Checklist*:

Information to include when describing an intervention and the location of the information

Item number	Item	Where located ** Primary paper (page or appendix number)	Other † (details)
	**BRIEF NAME**		
**1.**	Provide the name or a phrase that describes the intervention.	2	
	**WHY**		
**2.**	Describe any rationale, theory, or goal of the elements essential to the intervention.	4-5	______________
	**WHAT**		
**3.**	Materials: Describe any physical or informational materials used in the intervention, including those provided to participants or used in intervention delivery or in training of intervention providers. Provide information on where the materials can be accessed (e.g. online appendix, URL).	12, 15	_appendix____
**4.**	Procedures: Describe each of the procedures, activities, and/or processes used in the intervention, including any enabling or support activities.	6-12	______________
	**WHO PROVIDED**		
**5.**	For each category of intervention provider (e.g. psychologist, nursing assistant), describe their expertise, background and any specific training given.	1, 7-12, 17-18	______________
	**HOW**		

For peer review only

For peer review only - http://bmjopen.bmj.com/site/about/guidelines.xhtml

**6.**	Describe the modes of delivery (e.g. face-to-face or by some other mechanism, such as internet or telephone) of the intervention and whether it was provided individually or in a group.	6-18	______________
	**WHERE**		
**7.**	Describe the type(s) of location(s) where the intervention occurred, including any necessary infrastructure or relevant features.	6-18	______________
	**WHEN and HOW MUCH**		
**8.**	Describe the number of times the intervention was delivered and over what period of time including the number of sessions, their schedule, and their duration, intensity or dose.	6-18	______________
	**TAILORING**		
**9.**	If the intervention was planned to be personalised, titrated or adapted, then describe what, why, when, and how.	6-18	______________
	**MODIFICATIONS**		
**10.‡**	If the intervention was modified during the course of the study, describe the changes (what, why, when, and how).	N/A	______________
	**HOW WELL**		
**11.**	Planned: If intervention adherence or fidelity was assessed, describe how and by whom, and if any strategies were used to maintain or improve fidelity, describe them.	13-14	______________
**12.‡**	Actual: If intervention adherence or fidelity was assessed, describe the extent to which the intervention was delivered as planned.	N/A	______________

For peer review only

For peer review only - http://bmjopen.bmj.com/site/about/guidelines.xhtml

** **Authors** - use N/A if an item is not applicable for the intervention being described. **Reviewers** – use '?' if information about the element is not reported/not sufficiently reported.

† If the information is not provided in the primary paper, give details of where this information is available. This may include locations such as a published protocol or other published papers (provide citation details) or a website (provide the URL).

‡ If completing the TIDieR checklist for a protocol, these items are not relevant to the protocol and cannot be described until the study is complete.

* We strongly recommend using this checklist in conjunction with the TIDieR guide (see *BMJ* 2014;348:g1687) which contains an explanation and elaboration for each item.

* The focus of TIDieR is on reporting details of the intervention elements (and where relevant, comparison elements) of a study. Other elements and methodological features of studies are covered by other reporting statements and checklists and have not been duplicated as part of the TIDieR checklist. When a **randomised trial** is being reported, the TIDieR checklist should be used in conjunction with the CONSORT statement (see www.consort-statement.org) as an extension of **Item 5 of the CONSORT 2010 Statement.** When a **clinical trial protocol** is being reported, the TIDieR checklist should be used in conjunction with the SPIRIT statement as an extension of **Item 11 of the SPIRIT 2013 Statement** (see www.spirit-statement.org). For alternate study designs, TIDieR can be used in conjunction with the appropriate checklist for that study design (see www.equator-network.org).

For peer review only

For peer review only - http://bmjopen.bmj.com/site/about/guidelines.xhtml

SPIRIT CHECKLIST

Section/Item	Item no
Title	1
Trial Registration	2a
	2b
Protocol Version	3
Funding	4
Roles and responsibilities	5a
	5b
	5c
	5d
**Introduction**	
Background and rationale	6a
	6b
Objectives	7
Trial Design	8
**Methods: Participants, interventions, and outcomes**	
Study setting	9
Eligibility criteria	10
Interventions	11a

For peer review only

	11b
	11c
	11d
Outcomes	12
Participant Timeline	13
Sample Size	14
Recruitment	15
**Methods: Assignment of interventions (for controlled tri**	
Allocation	
Sequence generation	16a
Allocation concealment mechanism	16b
Implementation	16c

For peer review only

Blinding (masking)	17a
	17b
**Methods: Data collection, management, and analysis**	
Data collection methods	18a
	18b
Data management	19
Statistical methods	20a
	20b
	20c
**Methods: Monitoring**	
Data monitoring	21a

For peer review only

	21b
Harms	22
Auditing	23
**Ethics and dissemination**	
Research ethics approval	24
Protocol amendments	25
Consent or assent	26a
	26b
Confidentiality	27
Declaration of interests	28
Access to data	29
Ancillary and post-trial care	30
Dissemination policy	31a

For peer review only

	31b
	31c
**Appendices**	
Informed consent materials	32
Biological specimens	33

For peer review only

Description	Location
Descriptive title identifying the study design, population, interventions, and, if applicable, trial acronym	1
Trial identifier and registry name. If not yet registered, name of intended registry	3
All items from the World Health Organization Trial Registration Data Set	throughout
Date and version identifier	n/a
Sources and types of financial, material, and other support	23
Names, affiliations, and roles of protocol contributors	1
Name and contact information for the trial sponsor	name only, 23
Role of study sponsor and funders, if any, in study design; collection, management, analysis, and interpretation of data; writing of the report; and the decision to submit the report for publication, including whether they will have ultimate authority over any of these activities	23
Composition, roles, and responsibilities of the coordinating centre, steering committee, endpoint adjudication committee, data management team, and other individuals or groups overseeing the trial, if applicable (see Item 21a for data monitoring committee)	18
Description of research question and justification for undertaking the trial, including summary of relevant studies (published and unpublished) examining benefits and harms for each intervention	4-5
Explanation for choice of comparators	4-5
Specific objectives or hypotheses	13-14
Description of trial design including type of trial (eg, parallel group, crossover, factorial, single group), allocation ratio, and framework (eg, superiority, equivalence, noninferiority, exploratory)	6
Description of study settings (eg, community clinic, academic hospital) and list of countries where data will be collected. Reference to where list of study sites can be obtained	6
Inclusion and exclusion criteria for participants. If applicable, eligibility criteria for study centres and individuals who will perform the interventions (eg, surgeons, psychotherapists)	7
Interventions for each group with sufficient detail to allow replication, including how and when they will be administered	9

For peer review only

Criteria for discontinuing or modifying allocated interventions for a given trial participant (eg, drug dose change in response to harms, participant request, or improving/worsening disease)	n/a
Strategies to improve adherence to intervention protocols, and any procedures for monitoring adherence (eg, drug tablet return, laboratory tests)	13
Relevant concomitant care and interventions that are permitted or prohibited during the trial	7
Primary, secondary, and other outcomes, including the specific measurement variable (eg, systolic blood pressure), analysis metric (eg, change from baseline, final value, time to event), method of aggregation (eg, median, proportion), and time point for each outcome. Explanation of the clinical relevance of chosen efficacy and harm outcomes is strongly recommended	13-14
Time schedule of enrolment, interventions (including any run-ins and washouts), assessments, and visits for participants. A schematic diagram is highly recommended (see Figure)	11-12
Estimated number of participants needed to achieve study objectives and how it was determined, including clinical and statistical assumptions supporting any sample size calculations	8
Strategies for achieving adequate participant enrolment to reach target sample size	n/a
**als)**	
Method of generating the allocation sequence (eg, computer-generated random numbers), and list of any factors for stratification. To reduce predictability of a random sequence, details of any planned restriction (eg, blocking) should be provided in a separate document that is unavailable to those who enrol participants or assign interventions	8
Mechanism of implementing the allocation sequence (eg, central telephone; sequentially numbered, opaque, sealed envelopes), describing any steps to conceal the sequence until interventions are assigned	8
Who will generate the allocation sequence, who will enrol participants, and who will assign participants to interventions	7-8

For peer review only

Who will be blinded after assignment to interventions (eg, trial participants, care providers, outcome assessors, data analysts), and how	8
If blinded, circumstances under which unblinding is permissible, and procedure for revealing a participant's allocated intervention during the trial	8
Plans for assessment and collection of outcome, baseline, and other trial data, including any related processes to promote data quality (eg, duplicate measurements, training of assessors) and a description of study instruments (eg, questionnaires, laboratory tests) along with their reliability and validity, if known. Reference to where data collection forms can be found, if not in the protocol	9-15
Plans to promote participant retention and complete follow-up, including list of any outcome data to be collected for participants who discontinue or deviate from intervention protocols	supplementary PIS
Plans for data entry, coding, security, and storage, including any related processes to promote data quality (eg, double data entry; range checks for data values). Reference to where details of data management procedures can be found, if not in the protocol	16
Statistical methods for analysing primary and secondary outcomes. Reference to where other details of the statistical analysis plan can be found, if not in the protocol	16
Methods for any additional analyses (eg, subgroup and adjusted analyses)	16
Definition of analysis population relating to protocol non-adherence (eg, as randomised analysis), and any statistical methods to handle missing data (eg, multiple imputation)	16
Composition of data monitoring committee (DMC); summary of its role and reporting structure; statement of whether it is independent from the sponsor and competing interests; and reference to where further details about its charter can be found, if not in the protocol. Alternatively, an explanation of why a DMC is not needed	18

For peer review only

Description of any interim analyses and stopping guidelines, including who will have access to these interim results and make the final decision to terminate the trial	n/a
Plans for collecting, assessing, reporting, and managing solicited and spontaneously reported adverse events and other unintended effects of trial interventions or trial conduct	13, 16
Frequency and procedures for auditing trial conduct, if any, and whether the process will be independent from investigators and the sponsor	16
Plans for seeking research ethics committee/institutional review board (REC/IRB) approval	n/a
Plans for communicating important protocol modifications (eg, changes to eligibility criteria, outcomes, analyses) to relevant parties (eg, investigators, REC/IRBs, trial participants, trial registries, journals, regulators)	19
Who will obtain informed consent or assent from potential trial participants or authorised surrogates, and how (see Item 32)	7, supplementary consent
Additional consent provisions for collection and use of participant data and biological specimens in ancillary studies, if applicable	n/a
How personal information about potential and enrolled participants will be collected, shared, and maintained in order to protect confidentiality before, during, and after the trial	16
Financial and other competing interests for principal investigators for the overall trial and each study site	23
Statement of who will have access to the final trial dataset, and disclosure of contractual agreements that limit such access for investigators	19
Provisions, if any, for ancillary and post-trial care, and for compensation to those who suffer harm from trial participation	supplementary PIS
Plans for investigators and sponsor to communicate trial results to participants, healthcare professionals, the public, and other relevant groups (eg, via publication, reporting in results databases, or other data sharing arrangements), including any publication restrictions	19

For peer review only

Authorship eligibility guidelines and any intended use of professional writers	n/a
Plans, if any, for granting public access to the full protocol, participant- level dataset, and statistical code	19
Model consent form and other related documentation given to participants and authorised surrogates	supplementary materials
Plans for collection, laboratory evaluation, and storage of biological specimens for genetic or molecular analysis in the current trial and for future use in ancillary studies, if applicable	15

For peer review only

Missing

For peer review only

For peer review only

For peer review only

form

For peer review only

For peer review only

MeSH Descriptors

Infant, newborn
Pregnancy
Glucose Intolerance
Diabetes, Gestational
Insulin
Hyperglycaemia
Prediabetic state
Metformin
Glycated Hemoglobin
Overweight
Obesity
Diabetes Mellitus, Type 2
Diet, Healthy
Feasibility Studies
Mobile Applications
Body Weight
Hypoglycaemic agents
Fasting
Intermittent Fasting

For peer review only

BMJ Open

Manchester Intermittent Diet in Gestational Diabetes Acceptability Study (MIDDAS-GDM): A Two-Arm Randomised Feasibility Protocol Trial of an Intermittent Low-Energy Diet (ILED) in women with Gestational Diabetes and Obesity in Greater Manchester

Journal:	*BMJ Open*
Manuscript ID	bmjopen-2023-078264.R3
Article Type:	Protocol
Date Submitted by the Author:	05-Jan-2024
Complete List of Authors:	Dapre, Elizabeth; Wythenshawe Hospital<br>Issa, Basil; Manchester University NHS Foundation Trust, Department of Endocrinology and Diabetes<br>Harvie, Michelle; University Hospital of South Manchester NHS Foundation Trust, Genesis prevention centre<br>Su, Ting-Li; University of Manchester, Dentistry<br>McMillan, Brian; The University of Manchester, Centre for Primary Care and Health Services Research<br>Pilkington, A.; Wythenshawe Hospital<br>Hanna, F.; University Hospitals of North Midlands NHS Trust<br>Vyas, Avni; Manchester Metropolitan University Faculty of Health Psychology and Social Care, Health Professionals<br>Mackie, S.; Wythenshawe Hospital<br>Yates, James; Manchester University NHS Foundation Trust<br>Evans, Benjamin; Manchester University NHS Foundation Trust<br>Mubita, Womba; Manchester University NHS Foundation Trust, Department of Endocrinology and Diabetes<br>Lombardelli, Cheryl; Manchester University NHS Foundation Trust
**Primary Subject Heading**:	Diabetes and endocrinology
Secondary Subject Heading:	Nutrition and metabolism, Obstetrics and gynaecology, Reproductive medicine
Keywords:	Diabetes in pregnancy < DIABETES & ENDOCRINOLOGY, DIABETES & ENDOCRINOLOGY, Obesity, NUTRITION & DIETETICS, Feasibility Studies, Maternal medicine < OBSTETRICS

SCHOLARONE™
Manuscripts

For peer review only - http://bmjopen.bmj.com/site/about/guidelines.xhtml

Manchester Intermittent Diet in Gestational Diabetes Acceptability Study (MIDDAS-GDM): A Two-Arm Randomised Feasibility Protocol Trial of an Intermittent Low-Energy Diet (ILED) in women with Gestational Diabetes and Obesity in Greater Manchester

Authors: Dapre, E., Issa, B., Harvie, M., Su, T., McMillan, B., Hanna, F., Pilkington, A., Vyas, A., Yates, J., Mackie, S., Evans, B., Mubita, W., Lombardelli, C.

Dr Elizabeth Dapre (corresponding author)	elizabeth.dapre@hotmail.com<br>c/o Michelle Harvie, The Nightingale Centre, Wythenshawe Hospital, Southmoor Rd, Manchester M23 9LT
Dr Basil Issa	Diabetes and Endocrinology, Wythenshawe Hospital, Manchester, UK
Dr Michelle Harvie	The Nightingale Centre, Wythenshawe Hospital, Manchester, UK
Dr Ting-Li Su	Division of Dentistry, The University of Manchester, UK
Dr Brian McMillan	Division of Population Health, The University of Manchester, UK
Prof Fahmy Hanna	Diabetes and Endocrinology, University Hospitals of North Midlands, UK
Dr Andrea Pilkington	Obstetrics, St Mary's Wythenshawe Hospital, Manchester, UK
Avni Vyas	Division of Dietetics, Manchester Metropolitan University, UK
Benjamin Evans	Division of Clinical Trials, Manchester Foundation Trust, UK
James Yates	Division of Clinical Trials, Manchester Foundation Trust, UK
Sarah Mackie	Division of Clinical Trials, Manchester Foundation Trust, UK
Womba Mubita	Division of Clinical Trials, Manchester Foundation Trust, UK
Cheryl Lombardelli	The Nightingale Centre, Wythenshawe Hospital, Manchester, UK

Word Count: 3572

Submitted to: British Medical Journal Open

Start date of trial: 15th November 2022 (trial ongoing)

Date of submission:

For peer review only

Manchester **I**ntermittent **D**iet in Gestational **D**iabetes **A**cceptability **S**tudy (MIDDAS-GDM): A Two-Arm Randomised Feasibility Protocol Trial of an Intermittent Low-Energy Diet (ILED) in women with Gestational Diabetes and Obesity in Greater Manchester

Authors: Dapre, E., Issa, B., Harvie, M., Su, T., McMillan, B., Hanna, F., Pilkington, A., Vyas, A., Yates, J., Mackie, S., Evans, B., Mubita, W., Lombardelli, C.

Corresponding Author: elizabeth.dapre@nhs.net

Abstract (word count 298)

Introduction: The prevalence of gestational diabetes mellitus (GDM) is rising in the UK and is associated with maternal and neonatal complications. National Institute for Health and Care Excellence (NICE) guidance advises first line management with healthy eating and physical activity which is only moderately effective for achieving glycaemic targets. Approximately 30% of women require medication with metformin and/or insulin. There is currently no strong evidence base for any particular dietary regimen to improve outcomes in GDM. Intermittent low-energy diets (ILEDs) are associated with improved glycaemic control and reduced insulin resistance in type 2 diabetes (T2DM) and could be a viable option in the management of GDM. This study aims to test the safety, feasibility and acceptability of an ILED intervention amongst women with GDM compared to best National Health Service (NHS) care.

Method and analysis: We aim to recruit 48 women with GDM diagnosed between 24-28 weeks gestation from antenatal clinics at Wythenshawe and St Mary's hospitals, Manchester Foundation Trust, over 13 months starting in November 2022. Participants will be randomised (1:1) to ILED (2 low-energy diet days/week of 1000kcal and 5 days/week of the best NHS care healthy diet and physical activity advice) or best NHS care 7 days/week until delivery of their baby. Primary outcomes include uptake and retention of participants to the trial, and adherence to both dietary interventions. Safety outcomes will include birthweight, gestational age at delivery, neonatal hypoglycaemic episodes requiring intervention, neonatal hyperbilirubinaemia, admission to special care baby unit or neonatal intensive care unit, stillbirths, the percentage of women with hypoglycaemic episodes requiring

For peer review only

third-party assistance, and significant maternal ketonaemia (defined as ≥1.0mmol/L) Secondary outcomes will assess the fidelity of delivery of the interventions, and qualitative analysis of participant and healthcare professionals' experiences of the diet. Exploratory outcomes include the number of women requiring metformin and/or insulin.

Ethics and dissemination: Ethical approval has been granted by the Cambridge East Research Ethics Committee (22/EE/0119). Findings will be disseminated via publication in peer-reviewed journals, conference presentations, and shared with diabetes charitable bodies and organisations in the UK, such as Diabetes UK and the Association of British Clinical Diabetologists.

Trial Registration Number: NCT05344066

For peer review only

Strengths and limitations of this study

Strengths

- This study adds to the limited literature of safety of low-calorie diets amongst women with gestational diabetes
- This study has been informed by an experienced patient and public involvement and engagement group

Limitations

- This study involves a small sample size and is not powered to show efficacy of the intervention
- Women joining this study are likely to be highly motivated and adherence may not reflect that seen in the wider general population

INTRODUCTION (word count: 3539)

Background

In the UK up to 16% of pregnant women develop gestational diabetes (GDM) and the incidence is rising, in part due to increasing rates of obesity and maternal age(1,2). GDM is associated with maternal and neonatal complications (the risk increases with poor glycaemic control), including macrosomia, shoulder dystocia, caesarean-sections, neonatal hypoglycaemia and/or hyperbilirubinaemia, preterm delivery, preeclampsia, and stillbirth(2). Women who have had GDM have an estimated seven to ten-fold risk of developing type 2 diabetes (T2DM) later in life, and their children have a higher risk of developing adult obesity and T2DM(2–4).

Excessive weight gain in pregnancy is a particular problem for women with GDM(5). Harper *et al* demonstrated that, in women with GDM, every additional 1lb/week gained following diagnosis of GDM resulted in a 36-83% increased risk of pre-eclampsia, caesarean-section, macrosomia, and large for gestational age babies(5). Such studies highlight the importance of adequate weight control throughout pregnancy in women with GDM in order to reduce both maternal and neonatal complications.

First-line therapy for GDM is diet and physical activity. National Institute for Health and Care Excellence (NICE) guidance encourages a healthy diet with increased fruit

For peer review only

and vegetables, low-glycaemic index (GI) foods, reduced refined sugars, regular mealtimes and regular physical activity(6,7). These dietary measures fail to achieve glycaemic targets in ~30% of women who require medication with metformin and/or insulin(8). A range of dietary approaches have been studied including daily diets promoting low-GI diets (limiting refined and promoting complex carbohydrates), continuous modest energy-restriction (1800 Kcal/day), and low carbohydrate diets(9). There is currently no strong evidence base for any particular dietary regimen to improve outcomes in GDM.

Intermittent Low-Energy Diets (ILED)

The pathogenesis of GDM is strongly linked to obesity and chronic insulin resistance with many clinicians viewing GDM as a form of evolving T2DM. ILEDs typically include several days of a food based or meal replacement (e.g. drinks/bars) low-energy diet (650-1000kcal) diet, with a standard healthy (non-restrictive) diet recommended on the remaining days of the week. These diets are associated with significant reductions in weight, insulin resistance and hyperglycaemia in patients with prediabetes (HbA1c between 42-47mmol/mol, impaired glucose tolerance, or impaired fasting glycaemia), those with T2DM, and otherwise healthy subjects with overweight/obesity(10–17). These changes are equivalent to, or greater than, those achieved with standard daily energy restriction. A popular intermittent diet involves 2 consecutive or non-consecutive days/week of a low-energy diet (650-1000kcal) and 5 days of normal eating/week, known as the 5:2 diet. The Manchester Intermittent vs. Daily Diabetes App Study (MIDDAS), a study comparing an ILED and a continuous low-energy diet in T2D conducted in our unit, has shown the feasibility and safety of an ILED (800kcal for 2 days/week) in patients with T2DM and obesity, including those using insulin(18). At the end of the study approximately 70% of participants in the ILED group completed the study and achieved a 6% reduction of their baseline body weight. Forty two percent achieved an HbA1c of <48 mmol/mol(18). Given the strong overlap between GDM and T2DM, an ILED may be a promising dietary intervention for those with GDM.

A successful dietary approach to glycaemic control could empower women to take charge of the management of their GDM. Women with GDM are motivated to modify their diet driven by a desire to improve foetal outcomes(19–21).

For peer review only

Our Patient and Public Involvement and Engagement (PPIE) work indicates that women find the current National Institute for Health and Care Excellence (NICE) healthy eating guidance(6,7) confusing and vague. Our PPIE work has indicated that women are keen to try alternative dietary approaches, particularly if alternative diets are more effective in preventing the need to progress to medications such as metformin and insulin.

Aim

The aim of this trial is to test the safety, feasibility, and acceptability of an ILED in GDM to inform a future large-scale RCT.

METHODS

Trial Design

The study is a 28-week feasibility two-arm RCT in one NHS trust performed in patients with GDM and BMI ≥27.5 kg/m^2, or ≥25 kg/m^2 in high-risk minority ethnic groups (i.e. South Asian, Black African, African Caribbean) in Greater Manchester, between December 2022 and July 2024(22,23). There will be an embedded qualitative sub-study for participants and healthcare professionals. Due to the nature of the intervention, it will not be possible to blind the participants, clinicians, or study team to the treatment allocation after randomisation (the statistician and laboratory technicians will remain blinded).

Trial Setting and Recruitment

Participants will be recruited from antenatal clinics at Wythenshawe and St Mary's Hospitals, Manchester Foundation Trust (MFT) between November 2022 and December 2023. This is an urban area within Greater Manchester, and MFT serves patients from a wide range of minority ethnic and socio-demographic backgrounds. Women may self-refer to the antenatal clinic or be referred by their primary care team. Assessments will be carried out at MFT, or remotely if required by COVID-19 restrictions. The qualitative sub-study will be carried out at MFT, remotely, or at a location of the participant's choosing. We aim to recruit eligible participants over a period of 13 months. Potential participants will be given written information about the study and the opportunity to ask questions about the study prior to providing written consent (supplementary files 1 and 2).

For peer review only

Eligibility Criteria

<IMAGE ONE; figure1 inclusion exclusion jpg>

Participant Flow

Participants who fulfil the broad eligibility criteria will be notified about the trial by the GDM nurse/midwife at the time of their diagnosis. Those who are interested will be provided with a comprehensive patient information sheet (supplementary file 1) and more detailed eligibility screening questions. They will be asked to attend their first appointment having fasted for at least 6 hours and complete a four-day food diary (in line with our department's usual care). On attending their first routine clinic appointment, interested participants will receive further information from the research team. They will have the opportunity to ask questions, have their eligibility confirmed, and will be asked for their written consent to take part. Baseline assessments will be taken and participants will be randomised to their allocated treatment group using an online randomisation platform. Participant flow through the study is demonstrated in figure 2.

For peer review only

Sample Size

We plan to recruit 24 participants per study arm (n=48) which, when considering an estimated attrition rate of 15%, will provide complete outcome data on 40 participants(24–26). It has been estimated that 24 participants per group will be sufficient to determine study outcomes, in line with sample size recommendations for feasibility studies(27–29).

This number will allow us to enable estimation of recruitment/retention parameters with sufficient precision. For example, based on 40 completed participants, it will enable recruitment rates in the region of 25% to be estimated with an error of +/-13.42% at most; retention of 85% will be estimated with error of +/-11.07% at most. It is also sufficient for estimation of variability (e.g. standard deviation) in gestational weight gain and capillary glucose concentrations (proposed outcomes for the future definitive trial) with negligible bias (30).

Randomisation

The randomisation schedule will be independently set up and known only by the trial statistician. The trial statistician will be blinded to the participant's identity using "sealed envelope" software (https://www.sealedenvelope.com/). Randomisation will be carried out by generating an online pseudo-random list with random permuted blocks of varying size, known only to the statistician, and will be stratified for two variables:

- Age (18-35, >35 years)
- BMI (27.5-34.99kg/m^2 and >35kg/m2, >25-32.49kg/m^2 and >32.5kg/m^2 for high-risk minority ethnic groups (i.e. South Asian, Black African, African Caribbean)

These stratification variables have been chosen to reduce potential bias as we expect varying severity of GDM with increasing age and BMI, and possible differences in diet adherence(31).

Treatment to intervention and control groups will be allocated in a 1:1 ratio. A member of the research team who will be unaware of the randomisation algorithm (principal investigator, clinical research nurse, clinical research fellow or project manager) will trigger the randomisation procedure onsite; participants and clinicians will then be informed of the allocated treatment group. Clinicians will not be blinded due to the need to remain astute to safety, adherence, and side effects, requiring open and honest discussions with patients at each appointment. The statistician will remain blinded to treatment allocation until all outcome measures for all subjects have been collected.

Interventions

Study Arm 1: Best NHS Care Diet

All dietetic advice will be face to face or via video calls or the telephone. Participants will receive one to one personalised written and verbal advice from a dietitian to follow NICE diet and physical activity recommendations(6,7). Dietitians and midwives will receive training to ensure standardised delivery of information in clinic, and standardised patient information leaflets will be supplied to include information about increased fruit/vegetable intake, low-glycaemic index foods, and a reduction in free sugars. Information will include advice about the importance of regular meals; dietary advice aims to ensure that participants include at least 70g protein/28g fibre, and

predominantly mono- and polyunsaturated fats as per American Diabetes Association recommendations(32). Participants will be advised to be physically active, for example walking for 30 minutes after a meal. Participants will receive ongoing dietetic education and support every 2 weeks until delivery. This level of support is higher than typically provided in NHS GDM antenatal clinics due to limited resources but has been utilised to reflect best NHS care. They will receive suggested menus and recipes to follow the NICE recommended healthy diet for GDM. Participants will be asked to measure their capillary glucose four times each day and their ketones on two random (recorded) days of the week of their choosing (supplementary file 3).

Study Arm 2: Intermittent Low-Energy Diet (ILED)

Participants will receive the same level of dietetic support as the best NHS care group. They will be given advice on adopting an ILED which involves 2 non-consecutive low-energy diet days/week (1000kcal to include 100g low-GI carbohydrate and 70g of protein) and 5 days/week of the NICE healthy eating low-GI diet and physical activity recommended for the best NHS care group. The low-energy days involve women selecting a set number of portions of protein, carbohydrate, fat, fruit, vegetables, and dairy/dairy alternatives as described in previous studies(33). Each low-energy day includes ~210g of lean protein foods, 3-4 portions of wholegrain carbohydrates, 1x7g portion of fat, 5 portions of vegetables, 2 of fruit, and 3 of dairy/dairy alternatives. Food and drink will be self-selected and not provided by the study team. Participants will be provided with comprehensive food lists, advice on portion sizes for the low-energy days and suggested menus and recipes to follow for both the low-energy and NICE recommended healthy diet days (supplementary file 4). Both diets can be successfully adapted for people of different ethnicities and those following omnivorous, vegetarian and vegan diets. Participants will be asked to measure their capillary glucose four times each day and their ketones on (and the morning after) the two low-energy days (supplementary file 3).

<IMAGE 2: figure 2 flow jpg>

<IMAGE 3: figure 3 sched assessments jpg>

For peer review only

Outcomes

Primary outcomes

- Uptake rate measured as a percentage of eligible participants who consent to take part, including the proportion of women who were screened who did not meet the eligibility criteria, and the number of women who did not give consent to take part
- Recruitment rate measured as the number of eligible participants who consent to take part per month
- Retention rate measured as the number of randomised participants who complete the trial (those who attend the final visit) and the percentage of participants who attend all 8 visits
- Adherence to the dietary interventions assessed from self-reported adherence to the potential low-calorie days between randomisation and delivery
- Completion of self-assessed glucose and ketone readings assessed as a percentage of the required readings

- Safety outcomes:
    - Percentage of women following ILED/best NHS care with hypoglycaemia (episodes of blood glucose of <3.0mmol/mol) and hypoglycaemia requiring third-party assistance as measured by participants
    - Percentage of women who develop significant ketonaemia in both groups (defined as ≥1.0mmol/L) as measured by participants
    - Percentage of neonatal hypoglycaemic episodes requiring intervention (blood glucose checked 2- hours post-delivery and 2-hours thereafter for 12 hours according to local protocol), neonatal birth weight, gestational age at delivery, hyperbilirubinaemia/jaundice, and/or admission to Special Care Baby Unit or neonatal intensive care, and stillbirths
    - The incidence and rate of other adverse events (e.g. headaches, lethargy, constipation, or complications requiring hospital admission)

For peer review only - http://bmjopen.bmj.com/site/about/guidelines.xhtml

between the start of the trial intervention and delivery recorded as mild, moderate and severe, as defined by Common Terminology Criteria for Adverse Events version 5 (CTCAEv5)(34). Hospital admission for routine labour and delivery will not be classified as an adverse event.

Secondary outcomes

- Completeness of collection of trial endpoints including the percentage of completed weight measurements, 4-day food diaries, and International Physical Activity Questionnaire (IPAQ) scores
- Fidelity of delivery of the interventions will be measured through the number and modality of completed planned patient contacts, electronic and paper food diaries, and self-reported capillary glucose and ketone measurements
- Qualitative analysis of the acceptability and implementation of the interventions will be explored amongst a subset of participants (~10 in each group) and healthcare professionals through in-depth interviews

Exploratory outcomes

The following outcomes will be explored without statistical inference.

1. Maternal outcomes:
    - The percentage of women requiring metformin and/or insulin
    - Four-point capillary glucose profiles during third trimester (four times daily until delivery)
    - Change in fasting blood test results between baseline measurements, 36-37 weeks' gestation, and 12 weeks post-delivery (including oral glucose tolerance tests (OGTT)
    - Mode of delivery, development of preeclampsia, polyhydramnios (maximum liquor volume pool depth ≥8 cm)
    - Quality of life and health status questionnaires (WHOQoL-BREF and SF-36 questionnaires)(35,36)
2. Foetal outcomes:
    - Foetal weight
    - Gestational age at delivery

For peer review only

Measurements

The full schedule of assessments can be found in figure 3.

Physical measurements

Height, weight and blood pressure will be measured using standardised calibrated equipment in antenatal clinic.

Blood samples

Fasting venous blood samples will be collected to assess maternal HbA1c, fasting glucose, insulin, beta-hydroxybutyrate, liver function tests, free fatty acids, thyroid function tests, and full blood count. At the end of the study all samples will be disposed of in accordance with the Human Tissue Act (2004).

Questionnaires

Participants will be asked to complete four questionnaires at four time points throughout the trial (self-reported). Quality of life and health status will be assessed using the World Health Organisation Quality of Life Questionnaire (brief version) and the 36-Item Short Form Survey respectively(35,36). Physical activity will be measured using the International Physical Activity Questionnaire – Short Form, and diet quality will be assessed using the UK Diabetes and Diet Questionnaire(37,38). These questionnaires are self-reported by participants and have been chosen as they are widely used and validated tools.

Food Diaries

4-day dietary records will be completed using Libro (Nutritics Mobile Application) or paper food diaries, which will be entered into Nutritics software (Nutritics, Dublin, Ireland)(39). Participants who wish to use Libro will receive one to one training to use this by the study dietitian. Diaries will provide the research team with information about the intake of energy, carbohydrate, fat, protein, fibre, glycaemic index, and the timing of meals for participants in both groups. Participants will be asked what other

For peer review only

dietary modifications, if any, they have made at their fortnightly dietitian reviews to establish the adoption of any alternative dietary practices in the cohort.

Adverse Events

Participants in both groups will be asked about any adverse effects that they have experienced at each visit. These will include, but are not limited to, the potential effects of a low-energy diet, e.g. headache, lethargy, dizziness, constipation, indigestion, poor concentration, and hunger. Adverse events will be graded as per CTAEv5(34). Participants will be issued with a participation/emergency card with emergency contact details for the research team to be carried at all times and to be shown to the attending physician in case of emergency admission to hospital. All participants will be issued with clear instructions as to how to manage a hypoglycaemic and/or ketonaemic event (supplementary file 5).

Data management

Participant data will be anonymised and will be stored in line with the Medicines for Human Use (Clinical Trials) Amended Regulations 2006 and the Data Protection Act (2018) and archived in line with the Medicines for Human Use (Clinical Trials) Amended Regulations (2006) as defined in the MFT Clinical Trials Office Archiving SOP (11; Retention of Data, Off-Site Archiving, and Destroying Documents). Deidentified data will be stored in a study-specific Research Electronic Data Capture (REDCap) database. The sponsor will periodically audit the site study file, a sample of the case report form, consent forms, and source data, and check accuracy of the study database to ensure satisfactory completion.

Statistical methods

A statistical analysis plan specifying the full details of the primary and secondary outcomes, other variables, and methods, will be produced prior to trial analysis. The main analysis will be conducted via intention-to-treat population and will not undertake any significance tests. Descriptive, graphical (summary), and basic statistics (e.g. i. number, frequencies and percentages, ii. mean and standard deviation, or iii. median and quartiles as appropriate) will be presented as appropriate for each group respectively, for group difference jointly, and for each stratum. Per-protocol analysis will be considered as a secondary analysis. Levels of missing data will be investigated and used to inform future studies. No imputation will

For peer review only

be used. The end of study questionnaire will be analysed using appropriate descriptive statistics for closed questions and key themes will be extracted without formal analysis from open questions to inform future research.

Progression Criterion

The success of the feasibility trial will be defined by the progression criteria as outlined in table 1. Any concerns regarding a low retention rate will be discussed with the PPIE group. Interviews will include those who withdraw from the study to address potential reasons for withdrawal with the aim to improve retention in future.

	**Feasible (green)**	**Feasible with modification of the protocol (amber)**	**Not feasible (red)**
**Recruitment**	≥4 patients/month	>2 patients/month	≤2 patients/month
**Uptake to the feasibility study**	≥15%	10-15%	<10%
**Retention to the feasibility study**	>70%	50-70%	<50%
**Adherence to the ILED intervention**	>50% of the low-energy days completed (2/week between weeks 24-28 and delivery)	30-50% of the low-energy days completed (2/week between weeks 24-28 and delivery)	<30% of the low-energy days completed (2/week between weeks 24-28 and delivery)

Table 1: Trial progression criterion

Qualitative sub-study

Participants will be invited to take part in an optional qualitative sub-study at 11-13 weeks post-partum. Healthcare professionals delivering the interventions will also be invited to take part in this study.

We will undertake 11-12 semi-structured interviews with a subset of women from each group (ILED n=10 and best NHS Care n=10) at around 12 weeks post-delivery. The final sample size will be contingent on obtaining data saturation. We will also interview a sample of healthcare professionals (HCPs) involved in the delivery of care to study participants, including dieticians, obstetricians and midwives, including

For peer review only

those with leadership and clinical managerial roles. Sampling will be purposive, aiming to obtain women from a range of ethnic groups, ages, socioeconomic backgrounds, and self-reported engagement with the intervention. Participants and HCPs will be asked about their experiences and thoughts regarding the intervention, including motivating factors, and facilitators/barriers to engagement. Interviews will be conducted by a researcher from the University of Manchester/MFT who is independent from the research staff involved in the delivery and assessment of the programmes. Analysis will be conducted by two independent researchers at the University of Manchester/MFT using Braun and Clarke's thematic analysis approach to identify key issues around the acceptability, usefulness of the programmes, and feasibility of a subsequent trial(40). Analysis will be inductive: open-ended, exploratory, and driven by the data.

All participants will also be asked to complete an optional and anonymous end of study questionnaire developed by the study team at their post-partum visit (supplementary file 6). This will give participants the opportunity to feedback on their experience and will enable the study team to identify improvements to the design of a possible follow-up study.

Trial Steering Committee (TSC)

The trial steering committee will include an independent consultant endocrinologist, obstetrician, dietitian, and the patient representative. The committee will oversee the trial to ensure that it is carried out to the expected standards. The TSC will liaise with the CI to develop a schedule of meetings, proposed to occur every four months, with meetings to occur no less than annually. Minutes will be taken at TSC meetings and copies of the minutes will be filed in the Trial Master File; they will be shared with relevant stakeholders as appropriate.

Patient and public involvement

Patient and public involvement was actively sought throughout the planning and design of this trial and continues to form a key part of the trial as it progresses. The patient and public involvement and engagement (PPIE) group assisted in the development of all participant materials and provided valuable insight into the wording of participant information and acceptability of the proposed intervention. The

For peer review only

PPIE group will be updated as the trial progresses and a further focus group will be held to advise on the interview schedule and wording for the qualitative sub-study. The group will also be invited to aid in the development of summarising key findings for dissemination to relevant patient groups.

Ethics and dissemination

This study has been approved by the Cambridge East Research Ethics Committee and is sponsored by MFT. Findings will be disseminated via publication in peer-reviewed journals, conference presentations, and shared with diabetes charitable bodies and organisations in the UK, such as Diabetes UK and the Association of British Clinical Diabetologists. Anonymised data will be available upon formal request once the principal results of the study have been published. Planned modifications to the protocol will be approved by the research ethics committee before they are adopted into the study. An audit trail of ethical amendments and documentation will allow monitoring by the research team and external regulatory bodies.

This is the first study to assess the feasibility and safety of an ILED in GDM as compared to best NHS care. Given the increasing incidence of GDM and associated health risks this research is both pertinent and important. The study is not powered to show differences between ILED and best NHS care, however the planned quantitative and qualitative assessments will inform the feasibility of the programme and a future definitive trial.

For peer review only

Full References

1. The British Diabetic Association. Diabetes UK. [cited 2019 Nov 27]. What is gestational diabetes? Available from: https://www.diabetes.org.uk/diabetes-the-basics/gestational-diabetes

2. Kim C. Gestational diabetes: risks, management, and treatment options. Int J Womens Health. 2010 Oct 7;2:339–51.

3. Kim SY, Sharma AJ, Callaghan WM. Gestational diabetes and childhood obesity: what is the link? Curr Opin Obstet Gynecol. 2012 Dec;24(6):376–81.

4. Vounzoulaki E, Khunti K, Abner SC, Tan BK, Davies MJ, Gillies CL. Progression to type 2 diabetes in women with a known history of gestational diabetes: systematic review and meta-analysis. BMJ. 2020 May 13;m1361.

5. Harper L, Tita A, Biggio J. The Institute of Medicine Guidelines for Gestational Weight Gain after a Diagnosis of Gestational Diabetes and Pregnancy Outcomes. Am J Perinatol. 2014 Jun 27;32(03):239–46.

6. Webber J, Charlton M, Johns N. Diabetes in pregnancy: management of diabetes and its complications from preconception to the postnatal period (NG3). Br J Diabetes. 2015 Sep 1;15(3):107.

7. NHS. NHS Treatment Gestational Diabetes [Internet]. 2019 [cited 2020 Jan 10]. Available from: https://www.nhs.uk/conditions/gestational-diabetes/treatment/

8. Mayo K, Melamed N, Vandenberghe H, Berger H. The impact of adoption of the international association of diabetes in pregnancy study group criteria for the screening and diagnosis of gestational diabetes. Am J Obstet Gynecol. 2015 Feb;212(2):224.e1-9.

9. Han S, Middleton P, Shepherd E, Van Ryswyk E, Crowther CA. Different types of dietary advice for women with gestational diabetes mellitus. Cochrane Database Syst Rev. 2017 25;2:CD009275.

10. Carter S, Clifton PM, Keogh JB. Effect of Intermittent Compared With Continuous Energy Restricted Diet on Glycemic Control in Patients With Type 2 Diabetes: A Randomized Noninferiority Trial. JAMA Netw Open. 2018 Jul 20;1(3):e180756.

11. Antoni R, Johnston KL, Collins AL, Robertson MD. Effects of intermittent fasting on glucose and lipid metabolism. Proc Nutr Soc. 2017 Aug;76(3):361–8.

For peer review only

12. Cioffi I, Evangelista A, Ponzo V, Ciccone G, Soldati L, Santarpia L, et al. Intermittent versus continuous energy restriction on weight loss and cardiometabolic outcomes: a systematic review and meta-analysis of randomized controlled trials. J Transl Med. 2018 Dec;16(1):371.

13. Harris L, Hamilton S, Azevedo LB, Olajide J, De Brún C, Waller G, et al. Intermittent fasting interventions for treatment of overweight and obesity in adults: a systematic review and meta-analysis. JBI Database Syst Rev Implement Rep. 2018 Feb;16(2):507–47.

14. Harvie M, Howell A. Potential Benefits and Harms of Intermittent Energy Restriction and Intermittent Fasting Amongst Obese, Overweight and Normal Weight Subjects—A Narrative Review of Human and Animal Evidence. Behav Sci. 2017 Jan 19;7(4):4.

15. Harvie MN, Pegington M, Mattson MP, Frystyk J, Dillon B, Evans G, et al. The effects of intermittent or continuous energy restriction on weight loss and metabolic disease risk markers: a randomized trial in young overweight women. Int J Obes. 2011 May;35(5):714–27.

16. Wang X, Li Q, Liu Y, Jiang H, Chen W. Intermittent fasting versus continuous energy-restricted diet for patients with type 2 diabetes mellitus and metabolic syndrome for glycemic control: A systematic review and meta-analysis of randomized controlled trials. Diabetes Res Clin Pract. 2021 Sep;179:109003.

17. He S, Wang J, Zhang J, Xu J. Intermittent Versus Continuous Energy Restriction for Weight Loss and Metabolic Improvement: A Meta-Analysis and Systematic Review. Obesity. 2021 Jan;29(1):108–15.

18. McDiarmid S, Harvie M, Johnson R, Vyas A, Aglan A, Moran J, et al. Manchester Intermittent versus Daily Diet App Study (MIDDAS): A pilot randomized controlled trial in patients with type 2 diabetes. Diabetes Obes Metab. 2022 Mar;24(3):432–41.

19. Carolan M. Women's experiences of gestational diabetes self-management: A qualitative study. Midwifery. 2013 Jun;29(6):637–45.

20. Gao F, Luo H, Jones K, Nicholson W, Bell RA. Gestational Diabetes and Health Behaviors Among Women: National Health and Nutrition Examination Survey, 2007–2014. Prev Chronic Dis. 2018 Oct 25;15:180094.

21. McMillan B, Easton K, Goyder E, Delaney B, Madhuvrata P, Abdelgalil R, et al. Reducing risk of type 2 diabetes after gestational diabetes: a qualitative study to explore the potential of technology in primary care. Br J Gen Pract. 2018 Apr;68(669):e260–7.

22. National Institute for Health and Care Excellence. Diabetes in pregnancy: management from preconception to the postnatal period. NICE guideline. [Internet]. 2020. Available from: https://www.nice.org.uk/guidance/ng3/resources/diabetes-in-pregnancy-management-from-preconception-to-the-postnatal-period-pdf-51038446021

23. National Institute for Health and Care Excellence. Obesity: identification, assessment and management. NICE guideline. [Internet]. 2014 Nov [cited 2023 Aug 30]. Available

For peer review only

For peer review only - http://bmjopen.bmj.com/site/about/guidelines.xhtml

from: https://www.nice.org.uk/guidance/cg189/resources/obesity-identification-assessment-and-management-pdf-35109821097925

24. H. Al Wattar B, Dodds J, Placzek A, Beresford L, Spyreli E, Moore A, et al. Mediterranean-style diet in pregnant women with metabolic risk factors (ESTEEM): A pragmatic multicentre randomised trial. Persson LÅ, editor. PLOS Med. 2019 Jul 23;16(7):e1002857.

25. Moreno-Castilla C, Hernandez M, Bergua M, Alvarez MC, Arce MA, Rodriguez K, et al. Low-carbohydrate diet for the treatment of gestational diabetes mellitus: a randomized controlled trial. Diabetes Care. 2013 Aug;36(8):2233–8.

26. Rae A, Bond D, Evans S, North F, Roberman B, Walters B. A randomised controlled trial of dietary energy restriction in the management of obese women with gestational diabetes. Aust N Z J Obstet Gynaecol. 2000 Nov;40(4):416–22.

27. Julious SA. Sample size of 12 per group rule of thumb for a pilot study. Pharm Stat. 2005 Oct;4(4):287–91.

28. Sim J, Lewis M. The size of a pilot study for a clinical trial should be calculated in relation to considerations of precision and efficiency. J Clin Epidemiol. 2012 Mar;65(3):301–8.

29. Lancaster GA. Pilot and feasibility studies come of age! Pilot Feasibility Stud. 2015 Dec;1(1):1, 2055-5784-1–1.

30. Teare MD, Dimairo M, Shephard N, Hayman A, Whitehead A, Walters SJ. Sample size requirements to estimate key design parameters from external pilot randomised controlled trials: a simulation study. Trials. 2014 Dec;15(1):264.

31. Makgoba M, Savvidou M, Steer P. An analysis of the interrelationship between maternal age, body mass index and racial origin in the development of gestational diabetes mellitus: Gestational diabetes mellitus and risk factors. BJOG Int J Obstet Gynaecol. 2012 Feb;119(3):276–82.

32. American Diabetes Association. 13. Management of Diabetes in Pregnancy: *Standards of Medical Care in Diabetes—2018*. Diabetes Care. 2018 Jan;41(Supplement 1):S137–43.

33. Harvie M, Wright C, Pegington M, McMullan D, Mitchell E, Martin B, et al. The effect of intermittent energy and carbohydrate restriction *v* . daily energy restriction on weight loss and metabolic disease risk markers in overweight women. Br J Nutr. 2013 Oct 28;110(8):1534–47.

34. National Cancer Institute. Cancer Therapy Evaluation Program. 2017 [cited 2020 May 7]. Common Terminology Criteria for Adverse Events (CTCAE). Available from: https://ctep.cancer.gov/protocolDevelopment/electronic_applications/ctc.htm#ctc_50

35. World Health Organisation. WHOQOL: Measuring Quality of Life [Internet]. Available from: https://www.who.int/tools/whoqol

For peer review only

36. Nair R, Kachan P. Outcome tools for diabetes-specific quality of life: Study performed in a private family practice clinic. Can Fam Physician Med Fam Can. 2017 Jun;63(6):e310–5.

37. Ekelund U, Sepp H, Brage S, Becker W, Jakes R, Hennings M, et al. Criterion-related validity of the last 7-day, short form of the International Physical Activity Questionnaire in Swedish adults. Public Health Nutr. 2006 Apr;9(2):258–65.

38. England CY, Thompson JL, Jago R, Cooper AR, Andrews RC. Development of a brief, reliable and valid diet assessment tool for impaired glucose tolerance and diabetes: the UK Diabetes and Diet Questionnaire. Public Health Nutr. 2017 Feb;20(2):191–9.

39. Nutritics [Internet]. [cited 2020 Jul 12]. Available from: https://www.nutritics.com/p/home?utm_source=adwords&utm_campaign=Nutritics+Brand&utm_term=&utm_medium=ppc&hsa_grp=78385303355&hsa_mt=b&hsa_kw=&hsa_src=g&hsa_ad=380634402696&hsa_cam=1619635404&hsa_net=adwords&hsa_acc=2361676125&hsa_ver=3&hsa_tgt=dsa-814211721343&gclid=Cj0KCQjw6ar4BRDnARIsAITGzlAm_uy2YNRBU5N-egXXyehUQ_9CHKylLQI_LgCYJK9Jpet4oIvUM5saAkzREALw_wcB

40. Braun V, Clarke V. Successful qualitative research: a practical guide for beginners. Los Angeles: SAGE; 2013. 382 p.

Authors' contributions

Michelle Harvie, Basil Issa and Elizabeth Dapre designed the study, wrote the protocol and secured funding. Brian McMillan designed and worded the qualitative sub-study. Ting-Li Su developed the statistical analysis plan. Fahmy Hanna reviewed and advised on overall study design. Andrea Pilkington provided expert obstetric guidance for the protocol design. Avni Vyas, Cheryl Lombardelli and Michelle Harvie conducted dietetic reviews. Womba Mubita helped with recruitment and review of participants. James Yates and Benjamin Evans were responsible for project management and data reporting. Sarah Mackie coordinated the clinical trial. Elizabeth Dapre drafted the manuscript for publication, with input from Michelle Harvie, Basil Issa, Brian McMillan and Ting-Li Su. All authors have proofed and checked the manuscript.

Acknowledgments

For peer review only

With thanks to Mrs Rebecca Lumsden, our patient expert, whose insight has been invaluable in the design of the study. We are most grateful to Mrs Jodie Aspinall, and Miss Lisa Brew-Butler, specialist midwives who continue to help identify suitable candidates for the study, and Jessy Athirampuzha who has played a critical role in the recruitment and follow up of participants throughout the trial.

Funding statement:

This trial is funded by the National Institute for Health Research (NIHR201944) and sponsored by Manchester University NHS Foundation Trust (MFT). The funders of the study had no role in the study design or writing of the report. Dr Dapre is an NIHR sponsored GP academic clinical fellow.

Competing interests statement.

Michelle Harvie has co-authored three self-help books for the public to follow intermittent diets. All author proceeds are paid directly to the charity Prevent Breast Cancer (registered charity number 1109839) to fund breast cancer research.

For peer review only

Appendix

1.0 Patient Information Sheet (supplementary document 1)

2.0 Consent Form (supplementary document 2)

3.0 Self-monitoring schedule for capillary glucose and ketone monitoring (supplementary document 3)

4.0 Intermittent Low Energy Diet Day Example (supplementary document 4)

5.0 Medical Management Protocols (supplementary document 5)

6.0 End of Study Questionnaire (supplementary document 6)

For peer review only

LEGENDS

- <image 1>: Figure 1: Inclusion and exclusion criteria
- <image 2>: Figure 2: Participant flow through trial
- <image 3>: Figure 3: Schedule of assessments

Inclusion Criteria

- Pregnant women ≥18 years
- BMI of ≥27.5kg/m2 or a BMI ≥25 kg/m^2 in high risk minority ethnic group (i.e. South Asian, Black African, African Caribbean) and <50 kg/m2 at booking appointment (8-12 weeks' gestation)
- Newly diagnosed GDM according to local diagnostic criteria (fasting glucose ≥5.3mmol/l and/or 2-hour postprandial glucose ≥8.5mmol/l in a 75g OGTT) scheduled to receive first line diet and physical activity (best NHS care)
- 24-30 weeks' pregnant at screening appointment

Exclusion Criteria

- Pregestational type 1 or type 2 diabetes.
- Fasting glucose of ≥7 or 2-hour postprandial of ≥11 on OGTT (immediate intervention with medication would be required in this group of women)
- Current multiple pregnancy
- Maturity Onset Diabetes of the Young (MODY)
- Significant comorbid disease that in PI's opinion would preclude participation in the study e.g. chronic kidney disease, significant cardiac disease, significant history of disordered eating or severe psychological problems.
- Current participation in a GDM medication treatment trial
- People who are not capable of providing informed consent or adhering to the monitoring and safety protocols
- People who have previously had bariatric surgery for weight loss including gastric bypass and sleeve gastrectomy, and/or those prescribed weight loss medications (e.g. orlistat).
- Medications at the time of the OGTT that may interfere with results (e.g. high dose oral steroids, immunosuppressants)
- Previous history of intrauterine growth restriction
- Women who have lost more than 5% of their weight from booking appointment to screening appointment.

Figure 1: Inclusion and exclusion criteria

159x144mm (220 x 220 DPI)

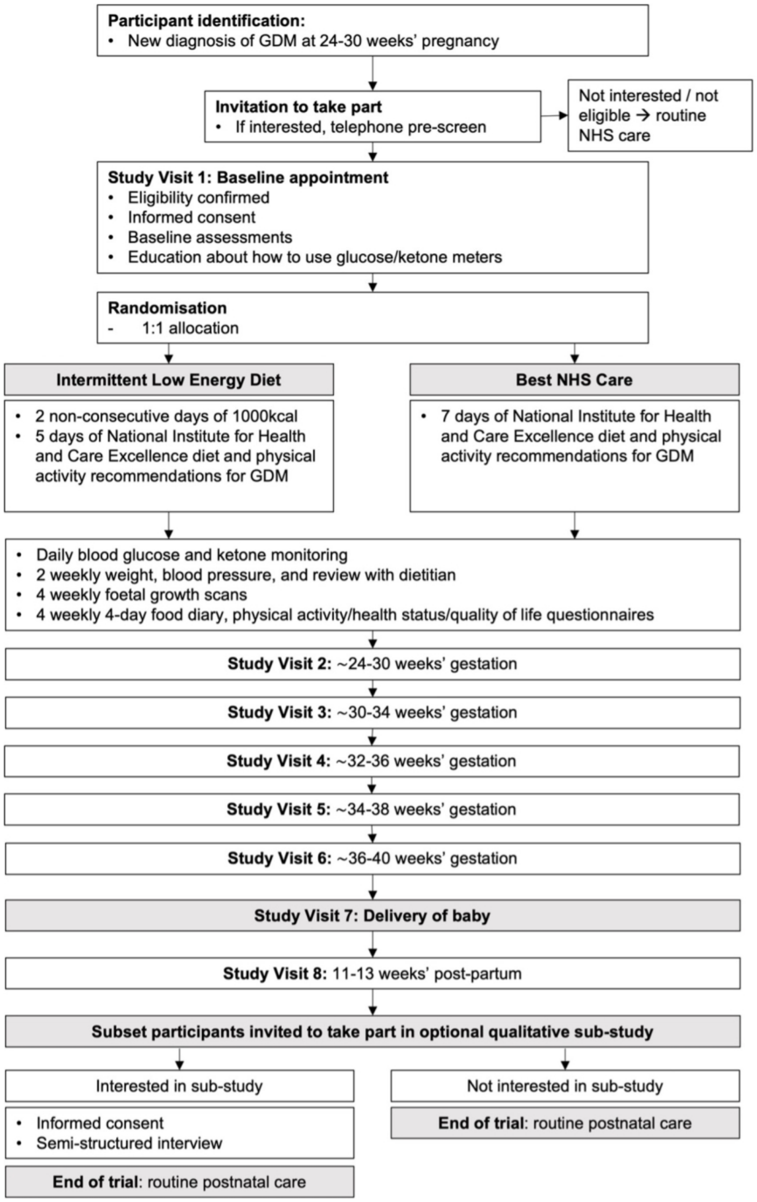

Figure 2: Participant flow through trial

154x245mm (220 x 220 DPI)

	Study Visit							
	1	2	3	4	5	6	7	8
Gestation (weeks)	~24-30	~24-30	~30-34	~32-36	~34-38	~36-40	delivery	11-13 post-partum
Eligibility confirmed	X							
Informed consent	X							
Randomisation	X							
Tailored dietitian review (face to face or remote)	X	X	X	X	X	X		X
Height	X							
Weight ^	X	X	X	X	X	X	X	X
Blood Pressure ^	X	X	X	X	X	X	X	X
Fasting blood sample *	X				X			X
Questionnaires #	X		X		X			X
4-day food diary		X			X			X
Foetal growth scan	X		X		X			
Review of glucose and ketone measurements		X	X	X	X	X		
Neonatal measurements §							X	
Oral glucose tolerance test								X
Exit interview / end of study questionnaires								X
Invitation to optional qualitative sub-study $								X

^ Frequency of assessment will be 2-4 weekly depending on whether appointment is face-to-face or virtual due to COVID-19
* Fasting bloods: urea and electrolytes, liver function tests, bone profile, lipids, thyroid function tests, HbA1c, beta-hydroxybutyrate, free fatty acids, full blood count, fasting glucose, insulin
# Questionnaires: World Health Organisation Quality of Life (brief version), 36-Item Short Form Survey, International Physical Activity Questionnaire (short form), UK Diabetes and Diet Questionnaire
§ Neonatal measurements include gestational age at delivery, mode of delivery, and neonatal weight
$ Sub-study involves semi-structured interviews exploring thoughts and experiences of the trial

159x239mm (72 x 72 DPI)

NHS
Manchester University
NHS Foundation Trust

MANCHESTER 1824
The University of Manchester

NIHR | National Institute for Health Research

Consultant Endocrinologist – Dr. Basil Issa
Research Dietitian – Dr. Michelle Harvie
Email: mft.middas.gdm@nhs.net
Tel: 07815987910

1st Floor Education & Research Centre
Manchester University NHS Foundation Trust
Wythenshawe Hospital
Manchester
M23 9LT

MIDDAS-GDM

Manchester Intermittent Diet in Gestational Diabetes Acceptability Study

Participant information sheet

We would like to invite you to take part in a research study **that is testing two different diet programmes which aim to help people with gestational diabetes control their blood sugars.**

If you decide to take part:

- You will be assigned to one of two diet programmes for the duration of your pregnancy. One involves following the standard NHS healthy diet recommendations for pregnancy, and the other follows the standard NHS healthy diet for 5 days/week plus two non-consecutive calorie restricted days of 1,000 kcal per week (both groups will be encouraged to be physically active).

- You will be asked to attend your routine appointments at Wythenshawe or St Marys Hospital and will have fortnightly appointments until delivery of your baby (some appointments may be virtual depending on COVID-19 restrictions). You will be asked to attend the hospital for a blood test 12 weeks after having your baby.

- You will be supported by a diabetes specialist dietitian, midwife, consultant endocrinologist, and your obstetric team throughout the study to help manage your pregnancy and blood glucose safely.

- Throughout the study you will be asked to monitor your food intake via a smartphone/tablet app, or on paper if you prefer, and you will receive feedback on this during your dietary reviews. Comprehensive dietary advice and recipes will be provided.

- Throughout the study you will be asked to monitor your blood sugar using a blood sugar meter four times a day, and you will also be asked to monitor your ketone levels two times on two days of the week (ketones indicate how well your body is using sugar or fat as an energy source). You will be taught how to check your blood sugar and ketone levels.

- If you would like to take part, or you have any questions, then please contact **mft.middas.gdm@nhs.uk**

For peer review only - http://bmjopen.bmj.com/site/about/guidelines.xhtml

This study is being carried out by a team of trained dietitians, doctors, nurses, midwives and researchers under the supervision of Dr. Basil Issa and Dr. Michelle Harvie at Wythenshawe and St Marys hospitals (Manchester University NHS Foundation Trust, MFT).

Before you decide if you would like to take part, it is important for you to understand why the research is being done and what taking part would involve for you. Please take your time to read the following information carefully. Discuss it with your friends, relatives, or GP if you wish to. Take time to consider whether or not you wish to take part.

Please ring the research team at the number at the top of the first page, or e-mail mft.middas.gdm@nhs.net if there is anything that is not clear, or if you would like more information. You can attend an information session about the diets and the study before agreeing to take part if you would like to.

Your participation in the study is entirely voluntary; you do not have to take part if you do not want to and you can opt out of the study at any time without giving a reason. Thank you for reading this information. We hope this research will be of interest to you.

Why are we doing this research?
Around 1 in 8 pregnant women can develop gestational diabetes. This condition causes risks to mother and baby from high blood sugar, high blood pressure, induced labours, caesarean-sections, and larger babies. Women often need medication to control blood sugar despite following recommended NHS healthy eating plans for pregnancy. Intermittent low-calorie diets (two non-consecutive days over the course of the week) improve blood sugar control and reduce the need for medication in patients with type 2 diabetes. We want to find out whether intermittent low-calorie diets might also improve blood sugar control in gestational diabetes and reduce the need for medication as it is a similar condition to type 2 diabetes.

What is the purpose of this research?
This study aims to assess the acceptability (to you) and safety of an intermittent low-calorie diet compared to the usual recommended NHS healthy eating and lifestyle plan for gestational diabetes. **A computer system will randomly allocate you to one of the two diets**. We want to find out which diet is most acceptable to women, whether there is any difference in the two diets' effect on blood sugar control, and any side effects experienced by women. The findings of this study will inform a larger study which will be designed to more closely compare the effect of the two diets on blood sugar control in women with gestational diabetes.

Why have I been asked to take part?
You have been invited to take part in this study because you have been diagnosed with gestational diabetes. We hope to recruit around 48 people to take part in this study.

For peer review only - http://bmjopen.bmj.com/site/about/guidelines.xhtml

NHS Manchester University NHS Foundation Trust

MANCHESTER 1824 The University of Manchester

NIHR | National Institute for Health Research

<u>What happens if you agree to take part?</u>
If you agree to take part you will be randomly allocated via a computer system to one of two diet and lifestyle programmes for the remaining weeks of your pregnancy.

Best NHS Care Healthy Diet programme
You will receive personalised advice from a specialist dietician. Recommendations will include increased fruit/vegetable intake, low glycaemic index starchy foods (i.e starchy foods which are slowly absorbed and take a while to raise your blood sugar level), reducing refined sugar, and having regular mealtimes. You will be advised how to design your diet to include the right amount of protein, fats, carbohydrates, and fibre, and will be given meal plans and recipes. You will also be advised to try to complete 150 minutes of moderate intensity exercise a week.

Intermittent Low-Calorie Diet programme
If you are allocated to this group you will receive personalised advice to follow a low-calorie diet of 1,000 kcal on two non-consecutive days of the week and the NHS healthy diet on the other five days of the week. The 1,000 kcal days include a set number of portions of protein, carbohydrates and fat foods, fruits, vegetables and dairy/dairy alternatives typically including ~210g (7 oz) of lean protein foods and 3-4 portions of wholegrain carbohydrates, 5 portions of vegetables, 2 of fruit, and 3 of dairy or dairy alternatives and a small amount of healthy fat. You will also be advised to try to complete 150 minutes of moderate intensity exercise a week.

Monitoring
You will have all your usual routine antenatal appointments including checks on your weight, blood pressure, blood tests and ultrasound scans. Extra blood tests will be done as part of the study, and these will be added on to samples taken during your routine blood tests.

You will be asked to monitor your blood sugar at home four times a day until your baby is born which is usual care in the clinic. In addition, you will be asked to record ketone levels on two days of the week (you will be taught how to do this using a finger prick machine). The results will be recorded when you attend clinic.

When babies are born to mothers with gestational diabetes it is normal that their birth weight is recorded and that their blood sugar is monitored for 12 hours following delivery; these results will be recorded by the research team.

You will be asked to attend an additional glucose tolerance test at the hospital 12 weeks after delivery to assess whether you have any residual diabetes (95% of women do not) and also to assess how sensitive your body is to insulin (an important risk factor for the development of diabetes in the future). You will be asked to attend at 9:00am having fasted (no food or drink apart from water) from midnight. A blood sample (around 10 mL/2 teaspoons) will be taken for glucose and insulin and you will be asked to drink a sugary drink with 75 grams of glucose. A further blood sample will be taken after 2 hours for glucose and insulin. You will need to

For peer review only - http://bmjopen.bmj.com/site/about/guidelines.xhtml

Manchester University NHS Foundation Trust

MANCHESTER 1824 The University of Manchester

NIHR | National Institute for Health Research

remain in hospital during this time. The reason for this is to help us to understand whether there could be any difference in the body's ability to process sugar between the two diet groups, and also to find out whether any women still have signs of diabetes after pregnancy.

Any blood samples taken as part of the study will be identifiable only using your study identification number and will have none of your personal details. Part of the sample will be sent for immediate analysis and any remaining will be stored securely, accessible only by the research team. At the end of the study any left over samples will be disposed of in accordance with the Human Tissue Act (2004).

You will be asked to record your food intake via the Libro smartphone/tablet app or in a paper diary for four days once a month throughout the study. You will also be asked to complete three questionnaires to assess your wellbeing and level of physical activity in these weeks, and a final end of study questionnaire at the final appointment.

Ongoing support from a specialist team of healthcare professionals

Your specialist team includes a Consultant Endocrinologist, Consultant Obstetrician, diabetes specialist dietitian, midwives, and a GP trainee with a special interest in women's health. The specialist team work closely with the usual obstetric teams involved in your care. Reviews with the team will be either face to face when you attend clinic or remotely using video calls.

Mobile Applications and Glucose Meters

You will be given the option of using a smartphone application called 'Libro' to record your dietary intake during the study. We will ask you to record 4 days of food and drink intake once a month across the study. Your diaries will be viewed by your allocated dietitian who will provide personalised dietary feedback via the app. You are also free to record more days of your diet should you wish, which some people find helpful. If you do not want to use the mobile app you can use paper instead. You will be supported to set up and use the Nutritics Libro App at your appointments. You do not have to use the application to be part of the study.

Your blood sugar will be monitored using a glucose monitoring device which checks your blood sugar using a 'fingerprick' blood test. You will be shown how to do this yourself. With your permission the research team will make a note of your glucose readings at every visit, either by checking your glucose monitoring device, or by uploading your glucose meter readings onto the computer if you are using a mobile application.

The self-monitoring schedule is as follows:

**Intermittent low-energy diet monitoring**		**Best NHS care monitoring**	
Ketones (low kcal days)	Glucose	Ketones (2 days/wk)	Glucose
Fasting before breakfast the morning after each of the low-calorie days	Fasting (morning)	Fasting before breakfast on 2 non-consecutive days/weeks	Fasting (morning)
	1hr post breakfast		1hr post breakfast
	1hr post lunch		1hr post lunch

For peer review only - http://bmjopen.bmj.com/site/about/guidelines.xhtml

NHS Manchester University NHS Foundation Trust
MANCHESTER 1824 The University of Manchester
NIHR | National Institute for Health Research

1 hour post evening meal on each of the low-calorie days	1hr post dinner	1 hour post evening meal on 2 non-consecutive days/week	1hr post dinner

What should I do if my blood glucose or ketones are out of range?

Low Blood Sugar

It is unlikely that your blood sugar levels will drop too low as a result of being on the intermittent low calorie or the NHS standard diets. You are advised to take 15-20g of 'rapid acting' carbohydrate if your blood glucose is <4 mmol/L). Examples include 170-225ml Lucozade Original (not Lucozade Sport), a small carton of fruit juice, 5-6 glucose tablets, 4/5 jelly babies, or a small tin of cola (150-200ml). You will need to repeat the treatment every 15 minutes until your blood glucose is ≥4 mmol/l.

The following table highlights when you need to consider an additional slower acting carbohydrate:

Situation	Acceptable slow acting carbohydrate
Less than 1 hour before the next meal	Try and avoid
1-2 hour before the next meal	10g (eg half of one of the items below)
More than 2 hours before the next meal	15-20g (eg slice of toast, piece of fruit, small bowl of cereal, glass of milk)

Raised Ketones

It is unlikely that your ketone levels will rise significantly as a result of being on the intermittent low calorie or NHS standard diets.

If your ketone levels are ⩾1.0 mmol/L on a fasting sample:

- Drink 1L fluids and repeat ketone levels after 4 hours.
- If your ketone level has improved (<1.0mmol/L), no further action is required.
- If your ketone level has increased or remains the same, repeat your ketone level after 2 hours.
- If your ketone level is persistently increased, consume 40g carbohydrates (eg one bagel, bowl of cereal and a banana, small jacket potato), and repeat in 2 hours.
- Continue to do this until your ketone levels are <1.0mmol/L.

Make Immediate Contact with the research team if:

Blood glucose	• Your blood glucose is <3.0 mmol/l or you have symptoms requiring medical attention which are thought to be due to low blood glucose,<br>• Your fasting blood glucose is >5.2 mmol/L on more than a quarter of your measurements on two days in a row,<br>• Your 1 hour post-meal blood glucose is >7.7 mmol/L on more than a quarter of your measurements on two days in a row

For peer review only - http://bmjopen.bmj.com/site/about/guidelines.xhtml

Manchester University NHS Foundation Trust

MANCHESTER 1824 The University of Manchester

NIHR | National Institute for Health Research

Ketones	• Your blood ketones are >1.0 mmol/L

Will I need medications?
If your blood sugars are found to be high despite following the recommended diet and lifestyle programmes you may be advised to start medication to help control your blood sugar. You will be advised on changes to your medications by a diabetes specialist nurse/diabetes midwife and also a Consultant Endocrinologist if required. This is usual practice regardless of whether you are taking part in the study.

What care will I receive after the study has stopped?
At the end of the study, you will be provided appropriate ongoing dietary advice from the study dietitian following your final glucose tolerance test to follow the NHS healthy eating and lifestyle plan. You will receive routine postnatal care from your GP, hospital team, and dietitian if required. You will be advised to see your GP for an annual blood test to check your blood sugar levels (this is routine care for women with gestational diabetes). Approximately 5% of women with gestational diabetes have residual diabetes after delivery. This will be identified from your glucose tolerance test/HbA1c; if this is the case you and your GP will be informed. Your GP will take over the management of your diabetes as per routine care outside the study.

Interview sub study
Women in this study may be invited to take part in an interview at the end of the study . You will be asked about your views and experiences on trying to follow your allocated diet programme. This interview can be arranged at a time that suits you, either at Wythenshawe or St Marys Hospital, at your home, or over the telephone. There is no obligation to take part in this interview study.

<u>Frequently asked questions</u>

Do I have to take part?
No, you do not have to take part if you do not wish to and your decision will not affect any standard of care you receive at Wythenshawe or St Marys hospitals (Manchester University NHS Foundation Trust, MFT).

What happens if I change my mind?
It is OK if you agree to take part in the study but later change your mind. You do not need to give a reason and it will not affect the standard of care you receive. The study team may also choose to withdraw you if it is necessary for your health or safety due to unexpected findings during the study. If you decide to withdraw from the study, or the study is stopped for any reason, you will be asked whether or not you are happy for us to keep the data that may have already been collected. If you do withdraw from the study you will continue to be cared for by your usual specialist diabetes and obstetric teams for the duration of your pregnancy. You will still have the option of completing the end of study questionnaire and/or interview to provide feedback; this is very useful for the research team to help us understand potential reasons you may have chosen to withdraw from the study.

For peer review only - http://bmjopen.bmj.com/site/about/guidelines.xhtml

You will also have the option that if you withdraw, researchers may still collect relevant information about your pregnancy and/or gestational diabetes from your medical records within the 18-month study duration. This will be an option on the consent form.

Are there any benefits from taking part?
You will receive frequent personalised advice and support to follow two diet and lifestyle programmes which may help to control your blood sugar levels throughout your pregnancy. The information gained from this study will also help inform the future NHS care of patients with gestational diabetes.

Are there any risks from taking part?
Research has found that diets consisting of two low-calorie days a week are very low risk. Pregnant women will develop slightly higher levels of ketones when following low calorie diets than women who are not pregnant. Ketones are produced naturally by the body when the body uses fat stores for energy (i.e. when we follow a low calorie diet or haven't eaten enough because we are ill).

Some research suggests that very high levels of ketones throughout pregnancy may cause a higher risk of babies being slightly smaller than average. It is very unlikely that you will develop high levels of ketones by following this diet. You will be provided with a ketone meter, and you will be asked to check your ketone levels after an evening meal on your low-calorie day, and the following morning, to make sure that your ketone levels are normal.

On your low-calorie days you may feel slightly more hungry, or you may experience other effects such as increased nausea, light headedness, or tiredness. It is important that you eat regularly throughout the day to reduce the risk of this happening. You will be asked to report any side effects of following the diet to the team at each appointment.

What happens if my baby or I become unwell during the study?
The safety of you and your baby are of utmost importance and remain our priority. In the instance that either of you become unwell your case will be reviewed by our specialist team and your suitability for continuing in the trial will be decided. Although it remains exceptionally rare, were you to experience the unexpected loss of your baby you will be withdrawn from the trial and supported by the dedicated specialist bereavement team at the hospital. Any information which has been collected as part of the trial will be stored securely and once we have finished the study we will keep some of the data so we can check the results. We will write our reports in a way that no-one can work out that you took part in the study.

What will happen to blood samples which are taken?
Some blood samples taken as part of the study will be sent to the laboratory immediately for analysis and any remaining will be stored securely for the duration of the study. Only your 'study ID' will be used – the samples will have none of your personal details on them. At the end of the study any remaining samples will be disposed of in accordance with the Human Tissue Act (2004).

For peer review only - http://bmjopen.bmj.com/site/about/guidelines.xhtml

Manchester University NHS Foundation Trust

MANCHESTER 1824 The University of Manchester

NIHR | National Institute for Health Research

What happens if something goes wrong?
If you have a concern about any aspect of this study, you should ask to speak with the lead researchers who will do their best to answer your questions (**Dr. Basil Issa** or **Dr. Michelle Harvie – via the study office – via michelle.harvie@manchester.ac.uk or telephone 0161 291 4410**). If you remain unhappy and wish to complain formally, you can do so through the NHS complaints procedure. Details can be obtained from the NHS Patient and Liaison Service (PALS) on **Tel:** 0161 276 8686 or contact the team by email pals@mft.nhs.uk.

The hospital is insured to carry out clinical research through the NHS Indemnity scheme. If something did go wrong and you were harmed or suffered deterioration in your health as a result of taking part in this study then you may have grounds for legal action or compensation.

Additional information about the study

Will my lifestyle be affected if I take part?
An essential aspect of this study is a change to your diet and physical activity patterns with support from a specialist team of healthcare professionals.

Payments
We are able to offer free parking at Wythenshawe/St Marys Hospitals for study visits and offer reimbursement for reasonable travel expenses (car, bus or tram) linked to visits for this study. There are no other payments for taking part.

Will my details be kept confidential?
Yes. The study team and any associated regulatory authorities follow strict ethical and legal guidance regarding participant confidentiality. Any information we have about you will be handled in confidence and will only be used for the purposes of this study. All data recorded will be coded and your name will remain anonymous.

During the study we will inform your GP via letter of your participation in the study and your ongoing results, including your weight, blood tests, any abnormal findings and any recommendations for treatment.

If you join the study, some relevant parts of your medical records may be looked at by authorised personnel at Wythenshawe or St Marys hospitals prior to starting the study. These records may also be looked at by an independent auditing body and regulatory authorities to check that the study is being carried out correctly. We will only access parts of your medical records that are relevant to this research and all information accessed will be kept strictly confidential.

How will we use information about you?
We will need to use information from you and from your medical records for this research project.

For peer review only - http://bmjopen.bmj.com/site/about/guidelines.xhtml

This information will include the following:

- Initials
- NHS number
- Name
- Contact details
- Medical History including test results
- Demographic details

People will use this information to do the research or to check your records to make sure that the research is being done properly. People who do not need to know who you are will not be able to see your name or contact details. Your data will have a code number instead. We will keep all information about you safe and secure.

Once we have finished the study, we will keep some of the data so we can check the results. We will write our reports in a way that no-one can work out that you took part in the study. Other researchers from outside the Trust may ask to see this data for the purposes of furthering their research. We will only share this upon written request to the Trust. The external researchers will be asked to sign a Confidentiality Agreement before any data is shared.

What are your choices about how your information is used?
You can stop being part of the study at any time, without giving a reason, but we will keep information about you that we already have. If you choose to stop taking part in the study, we would like to continue collecting information about your health during pregnancy from your hospital records. If you do not want this to happen, tell us and we will stop. We need to manage your records in specific ways for the research to be reliable. This means that we won't be able to let you see or change the data we hold about you.

Where can you find out more about how your information is used?
You can find out more about how we use your information

- at https://research.cmft.nhs.uk/getting-involved/gdpr-and-research
- by asking one of the research team
- by sending an email to **mft.middas.gdm@nhs.net** or
- by ringing us on **07815987910**

How will my details be used to access the Mobile Applications?
None of your personal details (other than email from which an application is downloaded) will be needed to access the nutritics mobile applications. You can opt to have a 'dummy' e-mail and password under a pseudonym (fake name). Only the research team will know the dummy e-mail address you have been assigned to, in order to be able to review your data. The application will not contain your identifiable data. If you choose to use a mobile application to monitor your blood sugar levels the relevant terms of service for the app and the app developers privacy policy will apply. It will be your responsibility to read and understand these prior to download.

Will my insurance be affected if I take part in this study?

For peer review only - http://bmjopen.bmj.com/site/about/guidelines.xhtml

It is unlikely that your insurance premiums will be affected by participation in this study as the study has the potential to improve your diabetic control and reduce your risk of ill health. However, if you are at all concerned, then we advise that you contact your insurers and seek expert advice before agreeing to participate.

Who has reviewed this study?

Research in the NHS is looked at by an independent group of people called a Research Ethics Committee (REC). The REC is made up of experts, non-experts and members of the general public. Together they review research applications to ensure your safety, rights, wellbeing and dignity are protected at all times. This study has been reviewed and given favourable opinion by REC.

What will happen to the study results?

It is intended that the results of this study will be presented at conferences and published in medical journals so that we can explain to the medical community what our research results have shown. To do this our study information is double-checked by other professionals in research and healthcare. There is a possibility that the study and its results may be publicised for example on radio, television, magazines, books and websites. **You will not be identified in any publicity, reports or publication arising from this study.** If you would like a general summary of the results of the study you can select this on the consent form or please contact the research team.

Who is organising and funding the research?

Researchers from Wythenshawe hospital have designed this study and will be carrying out this research. This study has been funded by the National Institute of Health and Research.

<u>Further information and contact details</u>

For further information about this study, please contact **mft.middas.gdm@nhs.net** or **07815987910.**

Thank you for taking the time to read this information sheet.
We hope it has been of interest to you.

For peer review only - http://bmjopen.bmj.com/site/about/guidelines.xhtml

NHS
Manchester University
NHS Foundation Trust

MANCHESTER 1824
The University of Manchester

NIHR | National Institute for Health Research

Participant pathway for the study

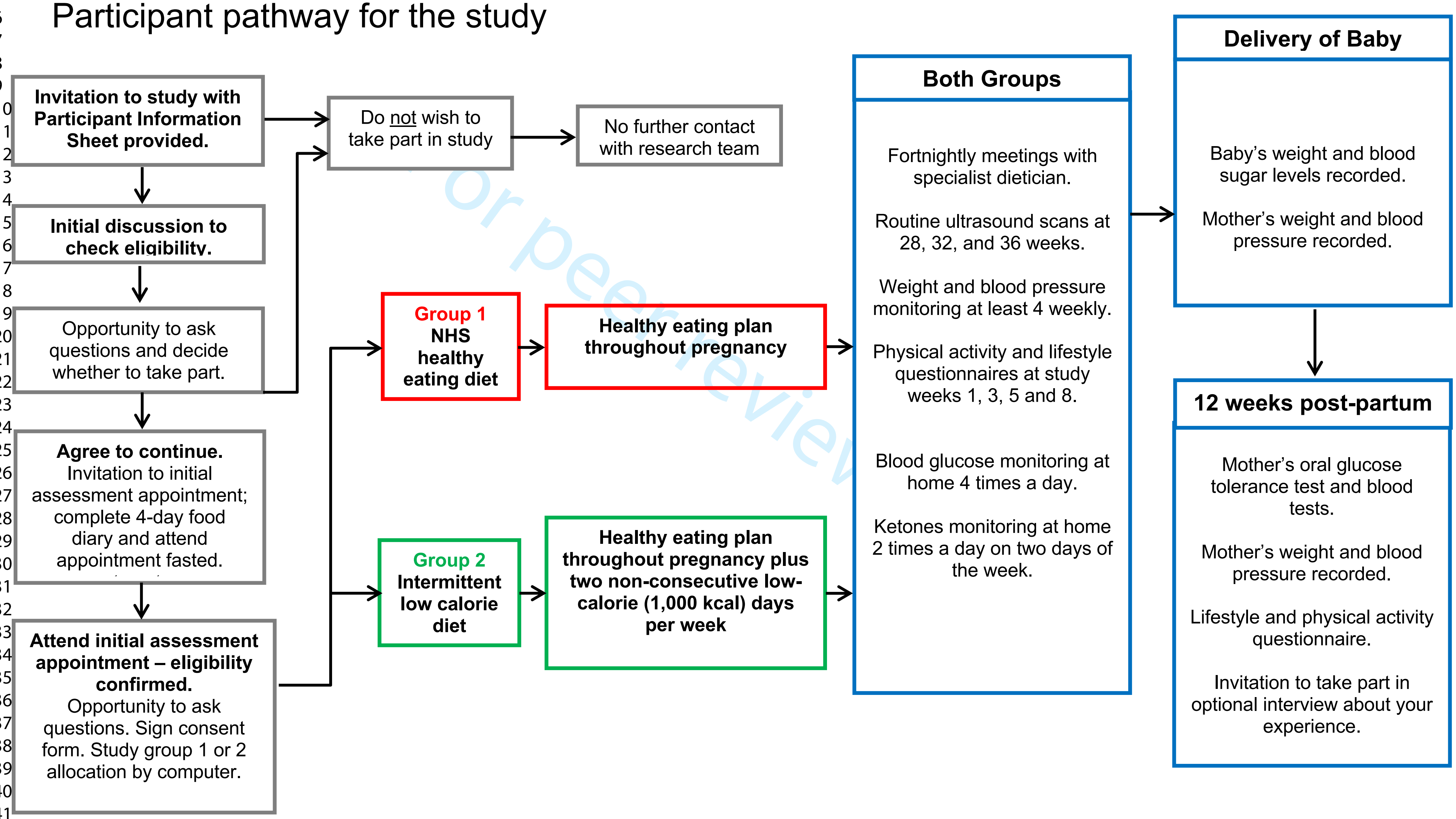

For peer review only - http://bmjopen.bmj.com/site/about/guidelines.xhtml

Manchester University NHS Foundation Trust

Consultant Endocrinologist – Dr. Basil Issa
Tel: 0161 291 7070
Research Dietitian – Dr. Michelle Harvie
Tel: 07815987910
Email: mft.middas.gdm@nhs.net

1st Floor Education and Research Centre
Manchester University NHS Foundation Trust
Wythenshawe Hospital
Manchester
M23 9LT

MIDDAS-GDM

Manchester Intermittent Diet in Gestational Diabetes Acceptability Study

Participant Informed Consent Form

Participant Identification Number:

Please **initial** box

1. I confirm that I have read and understand the participant information sheet (version …..) for the above study. I have had the opportunity to consider the information and ask questions, and have had these answered satisfactorily. ☐

2. I understand that my participation is voluntary and that I am free to withdraw at any time, without giving any reason, without my medical care or legal rights being affected. ☐

3. I understand that relevant sections of my medical notes and data collected during the study may be looked at by individuals from Manchester University NHS Foundation Trust and regulatory authorities, where it is relevant to my taking part in the research. I give permission for these individuals to have access to my records. ☐

4. I consent to the collection of blood samples to be collected as described in the participant information sheet. ☐

5. I understand that the information collected about me will be used to support other research in the future and may be shared anonymously with other researchers. ☐

6. I agree that my blood sugar readings can be recorded by the study team. ☐

7. I agree to my GP being informed of my participation in this study and changes to my weight, body measurements, blood results, questionnaire results and medications as required ☐

8. I understand that the information I provide to mobile applications as described in the Patient Information Sheet will be treated in line with the relevant terms of service and the app developers privacy policy at the time of downloading the application. ☐

For peer review only - http://bmjopen.bmj.com/site/about/guidelines.xhtml

NHS Manchester University NHS Foundation Trust

MANCHESTER 1824 The University of Manchester

NIHR | National Institute for Health Research

9. I have informed the study team of any health issues, including those which may affect my ability to follow the diet, and I will inform the study team of any unusual symptoms that occur during the diet. I will inform the study team of changes to my health status during the study. ☐

10. I have informed the study team of any health issues, including those which may affect my ability to exercise, and I will inform the study team of changes to my health status during the study. ☐

11. I consent to the storage of personal information (including electronic) for the purposes of this study. I understand that any information that could identify me will be kept strictly confidential and that no personal information will be included in the study report or other publication. ☐

12. I agree to take part in the above study. ☐

13. I agree that relevant information about my pregnancy and/or gestational diabetes can be obtained from my medical records within the 18-month study duration if I withdraw from the study early. ☐

14. I am aware that my non-identifiable trial data may be shared with other researchers for the purposes of research. ☐

Optional (delete as appropriate)

15. I agree to be approached to take part in sub-study 1 (interview study), and understand that I will be approached to take part in the sub-study regardless of whether I withdraw from the main study YES/NO

16. I would like to receive a summary of the final study results YES/NO

17. I agree to be contacted regarding future research opportunities YES/NO

My preferred contact (*please tick and include email if preferred*)

Do not contact ☐ Post ☐ Email ☐ ______________________________

For peer review only - http://bmjopen.bmj.com/site/about/guidelines.xhtml

Manchester University
NHS Foundation Trust

The University of Manchester

NIHR | National Institute for Health Research

Signatures

……………………………..………… …………………….. ……………..……………….
Name of participant Date Signature

…………………………………..……… …………………… ……………...……………….
Name of person taking consent Date Signature

When completed: 1 for participant; 1 for patient file; 1 for medical notes;; 1 (original) for site file

For peer review only - http://bmjopen.bmj.com/site/about/guidelines.xhtml

Self-monitoring schedule for capillary glucose and ketone monitoring

ILED		Best NHS Care	
**Ketones (low kcal days)**	**Glucose**	**Ketones (2 days/wk)**	**Glucose**
Fasting before breakfast the morning after each of the low-energy days	Fasting (morning)	Fasting before breakfast on 2 non-consecutive days / week	Fasting (morning)
1 hour post evening meal on each of the low-energy days	1hr post breakfast	1 hour post evening meal on 2 non-consecutive days / week	1hr post breakfast
	1hr post lunch		1hr post lunch
	1hr post dinner		1hr post dinner

For peer review only

Example of 1 day meal plan for Diet Day

The diet days aim to limit the calories to 1000 calories per day. You are aiming to include 2 (not consecutive) diet days each week. The other 5 days, follow the Mediterranean diet as described earlier. To keep the calories to 1000, the diet day will look like this:

Mixed diet		Vegetarian/ vegan diet
**4**	Carbohydrate portions	3
**6**	Protein portions	7
**5**	Vegetable portions	5
**2**	Fruit portions	2
**3**	Dairy portions	3
**1**	Fat portions	1

Below are some examples of meals that can be used to help you follow a 1000 calorie diet.. There are options for a mixed diet or vegan or vegetarian options, if you feel you wanted to try meat free days. Filling up on vegetables will make you feel less hungry

For peer review only

Mixed diet options

Breakfast	Portion	Dairy	Protein	Carb	Veg	Fruit	Fat
Grilled lean bacon	1 rasher	0	1	0	0	0	0
Grilled tomatoes	7 cherry tomatoes	0	0	0	1	0	0
Tea/ coffee	1 mug	0	0	0	0	0	0
Midmorning							
Diet or natural yogurt	1 small carton	1	0	0	0	0	0
Lunch							
Wholegrain bread	2 medium slices	0	0	2	0	0	0
Tuna	⅓ of a 120g can	0	1	0	0	0	0
Green salad	Cereal bowl full / 80 g with oil-free dressing	0	0	0	1	0	0
Satsumas	2	0	0	0	0	1	0
Mid afternoon							
Low fat cheese	30g / match box size	1	0	0	0	0	0
Apple slices	I medium apple (80g)	0	0	0	0	1	0
Tea/ coffee		0	0	0	0	0	0
Evening							
Vegetable rice	4 tablespoons cooked rice 160g of mix vegetables	0	0	2	2	0	0
Chicken curry	90g /average chicken breast (no skin) & ½ can tomatoes, 1 desertspoon oil	0	3	0	1	0	1
Bedtime							
Low fat houmous	1 level tablespoon	0	1	0	0	0	0
Pepper sticks	½ red pepper	0	0	0	1	0	0
Milk	1 small glass	1	0	0	0	0	0
**Total portions a day**		**3 portions**	**6 portions**	**4 portions**	**5 portions**	**2 portions**	**1 portion**

Vegetarian option

Breakfast		Dairy	Protein	Carb	Veg	Fruit	Fat
Egg	2 poached	0	2	0	0	0	0
Mushrooms	2 cupped handfuls / 80g	0	0	0	1	0	0
Tea/ coffee	1 mug	0	0	0	0	0	0
Midmorning							
Cheddar cheese	1 match box size / 30g	1	0	0	0	0	0
Cucumber	Sliced handful	0	0	0	1	0	0
Lunch							
Baked beans	2 tablespoons	0	1	0	0	0	0
Seeded bread toasted	1 medium sliced	0	0	1	0	0	0
Blueberries	1 handful	0	0	0	0	1	0
Mid afternoon							
Meat free ham	2 slice small	0	1	0	0	0	0
Pepper	½ sliced	0	0	0	1	0	0
Avocado	¼	0	0	0	0	0	1
Tea/ coffee	1 mug	0	0	0	0	0	0
Evening							
Vegetarian sausage casserole Jacket potato (100g)	1 grilled sausage 2 cereal bowls vegetables 1 ½ egg sized (100 g)	0	2	1	2	0	0
Bedtime							
Pear	1 medium	0	0	0	0	1	0
Low fat cream cheese	1 tablespoon	1	0	0	0	0	0
Whole wheat cracker	2 biscuits	0	0	1	0	0	0
Milk	1 small glass	1	1	0	0	0	0
**Total portions a day**		**3 portions**	**7 portions**	**3 portions**	**5 portions**	**2 portions**	**1 portions**

For peer review only

Vegan options

Breakfast		Dairy equivalent	Protein	Carb	Veg	Fruit	Fat
Branflakes	3 tablespoons	0	0	1	0	0	0
Milk- soya	200 ml	1	0	0	0	0	0
Tea/ coffee	1 mug	0	0	0	0	0	0
Midmorning							
Soya yogurt	3 tablespoons	1	0	0	0	0	0
Lunch							
Kidney bean & Vegetable chilli<br><br>Wholemeal pitta	3 tablespoons of beans 60g with 1 cereal bowl mixed vegetables & 1/2 can chopped tomatoes<br>½ pitta	0	2	1	2	0	0
Banana	1 medium	0	0	0	0	1	0
Mid afternoon							
Low fat hummus	2 level tablespoon	0	2	0	0	0	0
1 carrot	I medium carrot (80g)	0	0	0	1	0	0
Tea/ coffee	1 mug	0	0	0	0	0	0
Evening							
Quinoa	2 tablespoon cooked	0	0	1	0	0	0
Tofu	4 matchbox	0	2	0	0	0	0
Mixed salad with edamame beans	2 x Cereal bowl full with oil free dressing & 1 tablespoons of edamame	0	1	0	2	0	0
Bedtime							
Peanut butter	1 heaped teaspoon	0	0	0	0	0	1
Apple	1 medium sliced	0	0	0	0	1	0
Milk	1 small glass	1	0	0	0	0	0
**Total portions a day**		**3 portions**	**7 portions**	**2 portions**	**5 portions**	**2 portions**	**1 portions**

To help with estimation of portions the following tables outline weight and measures of the different food groups. Where possible household measures are given to make things a little easier. Use these to help you plan your 2 days in the week of 1000 calories.

**Carbohydrate**<br>**4 portions - mixed diet**<br>**3 portions - vegan/vegetarian**	**Equal to**
Wholewheat or oat breakfast cereal, e.g. wholewheat biscuit, malted wholewheat squares, Grapenuts, bran flakes, fruit & fibre	24g or 3 tablespoons or<br>1 whole wheat biscuit
Porridge oats or no-added sugar muesli	20g or 1 heaped tablespoon
Wholegrain, wholemeal, rye, granary bread	36g or medium slice of bread (other than rye), 1½ slices of rye, or ½ roll
Wholemeal or multigrain pitta bread or tortilla wrap, chapatti made without fat	60g or 1x 8” tortilla<br>or 1 standard pitta<br>or small thin chapatti
Rye crispbread, crackers, oak cakes	22g or 2 crispbreads/ 2 oatcakes
Wholegrain rice cake	16g or 2 rice cakes
Wholewheat pasta or rice - cooked amount<br><br>Cous cous, Bulgar wheat, Quinoa, Pearl barley	1 tablespoon uncooked 2 tablespoons cooked<br><br>30g- raw weight or 60g cooked
Lasagne (wholemeal if possible)	20g raw weight or<br>1 large sheet or 1½ smaller sheets
Noodles (wholemeal if possible)	25g raw weight or ½ block/nest
Baked or boiled potato (in skin), cassava, sweet potato	1½ egg sized potatoes or<br>100g raw weight
Wholemeal pizza base (topping is from other food groups)	35g or $^{1}/_{6}$ of thin 10” pizza base
Unsweetened popcorn	20g or 2 handfuls

For peer review only

**Protein**<br>**6 portions – mixed diet**<br>**7 portions – vegan/vegetarian**	**Equal to**
Fresh or smoked white fish (e.g. haddock or cod)	60g or 2oz<br>2 fish finger size
Seafood, e.g. prawns, mussels, crab	45g or 1½oz
Canned tuna or salmon in brine or spring water	45g or 1½oz<br>⅓ standard tin (120g)
Oily fish (fresh or tinned in tomato sauce or olive oil - drained), e.g. mackerel, sardines, salmon, fresh tuna, kippers, smoked salmon or trout	30g or 1oz or<br>¼ standard tin (120g) or<br>¼ fillet of salmon
Chicken, turkey, duck, pheasant (cooked without skin)<br>Lean beef, pork, lamb, rabbit, venison, offal (fat removed)<br>**Quorn fillets, steak, mince or pieces**<br>**Vegetarian mince frozen**	30g or 1oz or<br>1 slice size of playing card
Lean grilled bacon<br>**Quorn ham**	25g or ¾oz or<br>1 rasher
Lean ham<br>**Quorn bacon rashers (not slices)**	30g or 1oz or<br>1 medium, 2 small or 4 wafer thin slices
Eggs	60 g or 2 oz or<br>1 egg
Tofu	50g or $1^{2}/_{3}$ oz or<br>Size of 2 match boxes
Tempeh	25 g or 1 oz or<br>Size of 1 match box
Baked beans (reduced sugar)	60 g or 2 oz or<br>2 tablespoons
Lentils, chickpeas and kidney beans, mung beans, black eye beans, puy lentils, toor dahl, urad dahl,<br>Raw weight<br><br>Cooked or tinned weight	20g or ⅔ oz or<br>1 tablespoon raw<br><br>65g or 2oz or<br>1½ tablespoons cooked /tinned or 1 cupped handful
Soya beans (frozen or cooked) or edamame beans	30g or 1oz or<br>1 tablespoon
Vegetarian sausage	25g or ¾ oz or ½ sausage
Textured vegetable protein (TVP)	10g or ⅓ oz uncooked or<br>1 heaped tablespoon uncooked
Low fat hummus	30g or 1oz or<br>1 level tablespoon

For peer review only - http://bmjopen.bmj.com/site/about/guidelines.xhtml

Vegetables – min 5 portions<br>1 portion = 80g or 2⅔oz	1 portion is equal to
Asparagus, Aubergines, Broccoli, Brussel sprouts, Carrots, Cabbage, Cauliflower, Chinese leaves, Courgettes, Cucumber, Curly kale, Green beans, Lettuce (mixed leaves), Mange tout, Methi, Mushrooms, Okra, Pak choi, Peas, Sugar snap, Spinach, Spring greens cooked, Sweetcorn, Tomatoes, Watercress fresh	80g or 2 ⅔ oz or 2 spears of broccoli, 8 cauliflower florets. 3 heaped tablespoons of vegetables or large cereal bowl of salad.

Fruit - 2 portions.<br>1 portion = 80g or 2⅔oz (30g or 1oz dried fruits)	1 portion is equal to
Berries (e.g. blackberries, blueberries, redcurrants, raspberries, strawberries)<br>Cherries or grapes	80g or 2⅔oz<br>1 handful
Grapefruit, guava and mango	80g or 2⅔oz or<br>½ a whole fruit
Large fruit (e.g. melon, pineapple, papaya)	80g or 2⅔oz or<br>1 medium slice
Medium fruits (e.g. apple, pear, nectarine, orange, peach, banana, pomegranate)	80g or 2⅔oz<br>1 fruit
Small fruit (e.g. fresh apricots, kiwi, clementine, passion fruit, plums)	80g or 2⅔oz or<br>2 fruits
Any stewed fruit—unsweetened or with calorie-free sweetener e.g. apple, rhubarb	80g or 2⅔oz or<br>3 tablespoons
Kumquats, lychees, physalis	5 fruits
Dried fruits (raisins, currants, apricots)	30g or 1oz or<br>1 tablespoon

Milk and dairy foods - 3 portions	Equal to
Milk (semi-skimmed or skimmed)<br>Alternative 'milks' with added calcium, e.g. soya **	⅓ pint or 200ml or<br>1 small glass
Diet yoghurts, Low fat/fat-free Greek or Greek Style or natural yoghurts, fromage frais or plain soya yoghurt, high protein yogurt	120-150g or 4-5oz or<br>1 small pot or<br>3 tablespoons
Whole milk natural yogurt	80g or 1 ⅔ oz or<br>2 tablespoons
Cottage cheese	75g or 1½oz or<br>¼ pot, 2 tablespoons
Cream cheese (light or extra light)	30g or 1oz or<br>1 tablespoon
Lower fat hard cheeses e.g.:<br>Reduced fat cheddar, Edam,<br>Bavarian smoked, feta, ricotta, mozzarella, reduced fat halloumi, paneer made from semi-skimmed milk	30g or 1oz or Matchbox size<br>No more than 180g or 6oz a week

** we recommend soya milk as coconut, oat and almond milks are lower in protein and calcium

**Fat – 2 portions only**	**Equal to**
Margarine or low-fat spread (avoid the buttery types)<br>Olive oil or other oil<br>Oil based dressing<br>Pesto<br>Mayonnaise	8g or 1 teaspoon<br>1 dessertspoon of oil
Seeds (e.g. linseed, pumpkin, sunflower, sesame, chia, hemp)	7g or 1 dessertspoon
Unsalted or salted or dry roasted nuts	7g or 1 dessertspoon<br>3 walnut halves, 3 Brazil,<br>4 almonds, 8 peanuts,<br>10 cashews or pistachios
Olives	50g or 10 olives
Low fat mayonnaise, Curry paste or Harissa paste	15g or ½ oz or 1 tablespoon
Peanut butter (without palm oil)	11g or ⅓ oz or<br>1 heaped teaspoon
Cocoa powder	12 g or ⅓ oz or<br>2 heaped teaspoons
Avocado	40g or 1⅓ oz or<br>1/4 of an average pear
Low fat guacamole	40g or 1⅓ oz or 2 tablespoons

For peer review only

Medical Management Protocols

Hypoglycaemia

Participants will be advised to take 15-20g of rapid acting carbohydrate in the event of hypoglycaemia, (defined as blood glucose <4 mmol/L) which is anticipated to raise blood glucose by 3 mmol/L. Examples of rapid acting carbohydrate include 170-225ml Lucozade Original (not Lucozade Sport), a small carton of fruit juice, 5-6 glucose tablets, 4/5 jelly babies, or a small tin of cola (150-200ml). Participants will be advised to repeat the treatment every 15 minutes until blood glucose is ≥4 mmol/l. The following table highlights when participants should consider taking additional follow-up slower acting carbohydrate:

Situation	Acceptable slow acting carbohydrate
Less than 1 hour before the next meal	Try and avoid
1-2 hour before the next meal	10g
More than 2 hours before the next meal	15-20g

Ketonaemia

Ketone levels ≥1.0 mmol/L on a fasting sample:

- Drink 1L fluids and repeat ketone levels after 4 hours.
- If ketone level has improved (<1.0mmol/L), no further action required.
- If ketone level has increased or remains the same, repeat ketone level after 2 hours.
- If ketone level is persistently increased, consume 40g carbohydrates and repeat in 2 hours.
- Continue to do this until ketone levels <1.0mmol/L.

If a participant experiences >2 episodes of the above throughout the course of the study their notes will be reviewed by the PI and their suitability for remaining in the trial will be assessed.

For peer review only

Guidance for the introduction of diabetes medication (week 24-delivery)

Diabetes medication will be introduced according to the following protocol:

- If ≥25% fasting blood glucose readings are >5 mmol/l and/or ≥25% of 1 hour postprandial glucose readings are >7 mmol/l in a 7 day period: commence Metformin MR 500 mg daily to be increased every 3 days by 500 mg to 1 gram BD if tolerated.

- If after reaching optimal or maximum tolerated dose Metformin ≥25% fasting blood glucose readings are >5 mmol/l in a 7-day period: commence bedtime isophane insulin 4 units and uptitrate the dose by 2 units every 3 days aiming for a fasting glucose of ≤5 mmol/l,

- and/or ≥25% of 1 hour postprandial glucose readings are >7 mmol/l in a 7 day period: commence prandial fast acting insulin analogue (Humalog or Novorapid) 2-4 units with the relevant meal. Uptitrate the dose by 2 units every 3 days aiming for a 1 hour postprandial glucose of ≤7 mmol/l.

- Medication adjustment will be made in accordance with the above guidance.

For peer review only

NHS
Manchester University
NHS Foundation Trust

MIDDAS-GDM End of Study Questionnaire

Thank you for taking part in the MIDDAS-GDM Study.

This is one of the first studies of its kind. We hope to learn as much as possible from this study, in particular the views of people who have taken part. We are inviting you to provide your views on different aspects of the study and following the diet, and how we can improve our programmes and research studies in future.

Please complete the following questions and return this questionnaire to the MIDDAS-GDM study team in the envelope provided. If there is anything else you would like to say about your experiences of the study, please use the section at the end. Your answers to the questions below will remain anonymous.

1. What were your reasons for joining the study?

……

……

……

……

……

2. How satisfied were you with study overall (recruitment, appointments etc)? *(circle)*

1	2	3	4	5	6	7	8	9	10
Not at all satisfied		Slightly satisfied		Quite satisfied		Very satisfied			Extremely satisfied

Comments:……………………………………………………………………………………………

……

……

Study Appointments

3. You were asked to attend ………. additional face to face appointments at the hospital by the study team *(please fill in the number)*

For peer review only - http://bmjopen.bmj.com/site/about/guidelines.xhtml

Manchester University
NHS Foundation Trust

4. You were asked to attend ……….. additional virtual (i.e. video call or telephone) appointments by the study team *(please fill in the number)*

5. How do you feel about the number of additional appointments you were asked to attend? *(tick)*

☐ I was happy with the number of appointments

☐ I would have preferred fewer face to face appointments

☐ I would have preferred more face to face appointments

☐ I would have preferred fewer virtual appointments

☐ I would have preferred more virtual appointments

Comments:…………………………………………………………………………………………

……………………………………………………………………………………………………

6. How do you feel about virtual (i.e. video call or telephone) appointments?

☐ I prefer virtual appointments to face to face appointments (please explain why below)

☐ I prefer face to face appointments to virtual appointments (please explain why below)

Comments:…………………………………………………………………………………………

……………………………………………………………………………………………………

……………………………………………………………………………………………………

……………………………………………………………………………………………………

Diet

7. Which diet were you asked to follow? (*please tick*)

☐ Best NHS Care (i.e. increased fruit/vegetable intake, low-GI foods, reduction in free sugars, regular meals)

☐ Intermittent low energy diet (5 days of the best NHS care diet plus 2 non-consecutive days of 1000 kcal each week)

8. Was the diet easy to follow?

1	2	3	4	5	6	7	8	9	10
Not at all		Slightly		Moderately		Very			Extremely

For peer review only - http://bmjopen.bmj.com/site/about/guidelines.xhtml

NHS Manchester University NHS Foundation Trust

Comments:…………………………………………………………………………………………

…………………………………………………………………………………………………

9. Did you enjoy following the diet plan?

1	2	3	4	5	6	7	8	9	10
Not at all		Slightly		Moderately		Very			Extremely

Comments:…………………………………………………………………………………………

…………………………………………………………………………………………………

10. Would you make any changes to the written information (i.e. diet booklets, recipes) you were given on how to follow the diet plan?

☐ Yes

☐ No

If yes, what changes would you make?

Comments:…………………………………………………………………………………………

…………………………………………………………………………………………………

11. How was your first appointment with the dietitian? *(tick all that apply)***?**

☐ The amount of information I received was OK

☐ The amount of information I received was too little

☐ The amount of information I received was too much

☐ I was happy with the advice I received

☐ The advice I received could be improved (specify below)

Comments:…………………………………………………………………………………………

…………………………………………………………………………………………………

For peer review only - http://bmjopen.bmj.com/site/about/guidelines.xhtml

Manchester University
NHS Foundation Trust

12. Would you make any changes to the reviews (calls/face to face appointments) you had with the dietitian during your pregnancy ?

☐ Yes

☐ No

Comments:……………………………………………………………………………………………

……………………………………………………………………………………………………

13. How useful was your final appointment with the dietitian at 12 weeks post-delivery?

1	2	3	4	5	6	7	8	9	10
Not at all		Slightly		Quite		Very			Extremely

Comments:……………………………………………………………………………………………

……………………………………………………………………………………………………

14. Did you feel confident to exercise whilst on the diet plan?

1	2	3	4	5	6	7	8	9	10
Not at all confident		Slightly confident		Quite confident		Very confident			Extremely confident

Comments:……………………………………………………………………………………………

……………………………………………………………………………………………………

Additional support

15. Would any of the following have been useful & if so how often?

	No	Yes	Preferred method of contact	How often
Additional support from dietitian	☐	☐	Face to face / phone	
Additional support from the midwife	☐	☐	Face to face / phone	

For peer review only - http://bmjopen.bmj.com/site/about/guidelines.xhtml

NHS Manchester University NHS Foundation Trust

Additional support from the doctors in the clinic	☐	☐	Face to face / phone	
More contact with other women in the study following the diets	☐	☐	Face to face / phone	
Other, please specify:				

For peer review only

16. Did you receive any support outside of the study team help to keep you on track as you progressed through the study?

☐ No

☐ Yes

If yes, what support did you receive?

……………………………………………………………………………………………………

……………………………………………………………………………………………………

Record keeping

17. How did you find the finger prick testing requirements on the study? *(tick all those that apply)*

☐ Challenging but on the whole achievable

☐ Challenging and not achievable

☐ Not challenging at all

☐ I felt it was necessary to test this often to ensure my safety

☐ I felt it was unnecessary to test this often to ensure my safety

Comments:…………………………………………………………………………………………

……………………………………………………………………………………………………

18. How did you find the ketone testing requirements on the study? *(tick all those that apply)*

☐ Challenging but on the whole achievable

☐ Challenging and not achievable

For peer review only - http://bmjopen.bmj.com/site/about/guidelines.xhtml

Manchester University
NHS Foundation Trust

☐ Not challenging at all

☐ I felt it was <u>necessary</u> to test this often to ensure my safety

☐ I felt it was <u>unnecessary</u> to test this often to ensure my safety

Comments:…………………………………………………………………………………………

……

19. How did you find using Diasend software?

☐ Straightforward

☐ Challenging but on the whole <u>achievable</u>

☐ Challenging and <u>not achievable</u>

☐ I felt uncomfortable using computer software to keep track of my medical details

☐ I felt comfortable using computer software to keep track of my medical details

Comments:…………………………………………………………………………………………

……

20. How did you find completing the food diary during the study? *(tick all those that apply)*

☐ Challenging but on the whole <u>achievable</u>

☐ Challenging and <u>not achievable</u>

☐ Not challenging at all

Comments:…………………………………………………………………………………………

……

21. How did you find the physical activity questionnaires on the study? *(tick all those that apply)*

☐ Challenging but on the whole <u>achievable</u>

☐ Challenging and <u>not achievable</u>

☐ Not challenging at all

Comments:…………………………………………………………………………………………

……

For peer review only

For peer review only - http://bmjopen.bmj.com/site/about/guidelines.xhtml

NHS
Manchester University
NHS Foundation Trust

22. How did you find the quality of life questionnaires on the study? *(tick all those that apply)*

☐ Challenging but on the whole achievable

☐ Challenging and not achievable

☐ Not challenging at all

Comments:…………………………………………………………………………………………

……………………………………………………………………………………………………

Libro® app

23. Did you use the Libro® app?

☐ Yes

☐ No (please move to question 25)

24. Did you find the App helpful?

1	2	3	4	5	6	7	8	9	10
Not at all		Slightly		Moderately		Very			Extremely

Comments…………………………………………………………………………………………

……………………………………………………………………………………………………

25. What did you like about the App?

……………………………………………………………………………………………………

……………………………………………………………………………………………………

……………………………………………………………………………………………………

26. What did you dislike about the App and could be improved?

……………………………………………………………………………………………………

……………………………………………………………………………………………………

27. If you didn't use the app what were the reasons for this? *(tick all that apply)*

☐ Would prefer not to say ☐ Already using other health apps

For peer review only

For peer review only - http://bmjopen.bmj.com/site/about/guidelines.xhtml

- ☐ Don't like using apps in general
- ☐ Not user friendly
- ☐ Labour intensive / time consuming
- ☐ Prefer to use pen and paper
- ☐ Find mobile devices challenging
- ☐ Lack of regular internet access
- ☐ Other (provide details below)

……

……

……

Diasend Software

28. Did you find the Diasend software helpful?

1	2	3	4	5	6	7	8	9	10
Not at all		Slightly		Moderately		Very			Extremely

Comments…………………………………………………………………………………………………

……

29. What did you like about the Diasend software?

……

……

30. What did you dislike about the Diasend software and could be improved?

……

……

Study Improvements

31. What did you enjoy most about the study?

……

……

……

……

For peer review only - http://bmjopen.bmj.com/site/about/guidelines.xhtml

Manchester University
NHS Foundation Trust

32. What did you enjoy least about the study and could be improved?

....
....
....
....
....

<u>Any other comments about the study</u>

....
....
....
....
....
....
....
....

Thank you for completing this questionnaire, please return to:

MIDDAS-GDM Study Team, Nightingale Centre,

Wythenshawe Hospital, Manchester, M23 9LT

For peer review only

For peer review only - http://bmjopen.bmj.com/site/about/guidelines.xhtml

MeSH Descriptors

Infant, newborn
Pregnancy
Glucose Intolerance
Diabetes, Gestational
Insulin
Hyperglycaemia
Prediabetic state
Metformin
Glycated Hemoglobin
Overweight
Obesity
Diabetes Mellitus, Type 2
Diet, Healthy
Feasibility Studies
Mobile Applications
Body Weight
Hypoglycaemic agents
Fasting
Intermittent Fasting

For peer review only

SPIRIT CHECKLIST

Section/Item	Item no
Title	1
Trial Registration	2a
	2b
Protocol Version	3
Funding	4
Roles and responsibilities	5a
	5b
	5c
	5d
**Introduction**	
Background and rationale	6a
	6b
Objectives	7
Trial Design	8
**Methods: Participants, interventions, and outcomes**	
Study setting	9
Eligibility criteria	10
Interventions	11a

For peer review only

	11b
	11c
	11d
Outcomes	12
Participant Timeline	13
Sample Size	14
Recruitment	15
**Methods: Assignment of interventions (for controlled tri**	
Allocation	
Sequence generation	16a
Allocation concealment mechanism	16b
Implementation	16c

For peer review only

Blinding (masking)	17a
	17b
**Methods: Data collection, management, and analysis**	
Data collection methods	18a
	18b
Data management	19
Statistical methods	20a
	20b
	20c
**Methods: Monitoring**	
Data monitoring	21a

For peer review only

	21b
Harms	22
Auditing	23
**Ethics and dissemination**	
Research ethics approval	24
Protocol amendments	25
Consent or assent	26a
	26b
Confidentiality	27
Declaration of interests	28
Access to data	29
Ancillary and post-trial care	30
Dissemination policy	31a

For peer review only

	31b
	31c
**Appendices**	
Informed consent materials	32
Biological specimens	33

For peer review only

Description	Location
Descriptive title identifying the study design, population, interventions, and, if applicable, trial acronym	1
Trial identifier and registry name. If not yet registered, name of intended registry	3
All items from the World Health Organization Trial Registration Data Set	throughout
Date and version identifier	n/a
Sources and types of financial, material, and other support	23
Names, affiliations, and roles of protocol contributors	1
Name and contact information for the trial sponsor	name only, 23
Role of study sponsor and funders, if any, in study design; collection, management, analysis, and interpretation of data; writing of the report; and the decision to submit the report for publication, including whether they will have ultimate authority over any of these activities	23
Composition, roles, and responsibilities of the coordinating centre, steering committee, endpoint adjudication committee, data management team, and other individuals or groups overseeing the trial, if applicable (see Item 21a for data monitoring committee)	18
Description of research question and justification for undertaking the trial, including summary of relevant studies (published and unpublished) examining benefits and harms for each intervention	4-5
Explanation for choice of comparators	4-5
Specific objectives or hypotheses	13-14
Description of trial design including type of trial (eg, parallel group, crossover, factorial, single group), allocation ratio, and framework (eg, superiority, equivalence, noninferiority, exploratory)	6
Description of study settings (eg, community clinic, academic hospital) and list of countries where data will be collected. Reference to where list of study sites can be obtained	6
Inclusion and exclusion criteria for participants. If applicable, eligibility criteria for study centres and individuals who will perform the interventions (eg, surgeons, psychotherapists)	7
Interventions for each group with sufficient detail to allow replication, including how and when they will be administered	9

For peer review only

Criteria for discontinuing or modifying allocated interventions for a given trial participant (eg, drug dose change in response to harms, participant request, or improving/worsening disease)	n/a
Strategies to improve adherence to intervention protocols, and any procedures for monitoring adherence (eg, drug tablet return, laboratory tests)	13
Relevant concomitant care and interventions that are permitted or prohibited during the trial	7
Primary, secondary, and other outcomes, including the specific measurement variable (eg, systolic blood pressure), analysis metric (eg, change from baseline, final value, time to event), method of aggregation (eg, median, proportion), and time point for each outcome. Explanation of the clinical relevance of chosen efficacy and harm outcomes is strongly recommended	13-14
Time schedule of enrolment, interventions (including any run-ins and washouts), assessments, and visits for participants. A schematic diagram is highly recommended (see Figure)	11-12
Estimated number of participants needed to achieve study objectives and how it was determined, including clinical and statistical assumptions supporting any sample size calculations	8
Strategies for achieving adequate participant enrolment to reach target sample size	n/a
**als)**	
Method of generating the allocation sequence (eg, computer-generated random numbers), and list of any factors for stratification. To reduce predictability of a random sequence, details of any planned restriction (eg, blocking) should be provided in a separate document that is unavailable to those who enrol participants or assign interventions	8
Mechanism of implementing the allocation sequence (eg, central telephone; sequentially numbered, opaque, sealed envelopes), describing any steps to conceal the sequence until interventions are assigned	8
Who will generate the allocation sequence, who will enrol participants, and who will assign participants to interventions	7-8

For peer review only

Who will be blinded after assignment to interventions (eg, trial participants, care providers, outcome assessors, data analysts), and how	8
If blinded, circumstances under which unblinding is permissible, and procedure for revealing a participant's allocated intervention during the trial	8
Plans for assessment and collection of outcome, baseline, and other trial data, including any related processes to promote data quality (eg, duplicate measurements, training of assessors) and a description of study instruments (eg, questionnaires, laboratory tests) along with their reliability and validity, if known. Reference to where data collection forms can be found, if not in the protocol	9-15
Plans to promote participant retention and complete follow-up, including list of any outcome data to be collected for participants who discontinue or deviate from intervention protocols	supplementary PIS
Plans for data entry, coding, security, and storage, including any related processes to promote data quality (eg, double data entry; range checks for data values). Reference to where details of data management procedures can be found, if not in the protocol	16
Statistical methods for analysing primary and secondary outcomes. Reference to where other details of the statistical analysis plan can be found, if not in the protocol	16
Methods for any additional analyses (eg, subgroup and adjusted analyses)	16
Definition of analysis population relating to protocol non-adherence (eg, as randomised analysis), and any statistical methods to handle missing data (eg, multiple imputation)	16
Composition of data monitoring committee (DMC); summary of its role and reporting structure; statement of whether it is independent from the sponsor and competing interests; and reference to where further details about its charter can be found, if not in the protocol. Alternatively, an explanation of why a DMC is not needed	18

For peer review only

Description of any interim analyses and stopping guidelines, including who will have access to these interim results and make the final decision to terminate the trial	n/a
Plans for collecting, assessing, reporting, and managing solicited and spontaneously reported adverse events and other unintended effects of trial interventions or trial conduct	13, 16
Frequency and procedures for auditing trial conduct, if any, and whether the process will be independent from investigators and the sponsor	16
Plans for seeking research ethics committee/institutional review board (REC/IRB) approval	n/a
Plans for communicating important protocol modifications (eg, changes to eligibility criteria, outcomes, analyses) to relevant parties (eg, investigators, REC/IRBs, trial participants, trial registries, journals, regulators)	19
Who will obtain informed consent or assent from potential trial participants or authorised surrogates, and how (see Item 32)	7, supplementary consent
Additional consent provisions for collection and use of participant data and biological specimens in ancillary studies, if applicable	n/a
How personal information about potential and enrolled participants will be collected, shared, and maintained in order to protect confidentiality before, during, and after the trial	16
Financial and other competing interests for principal investigators for the overall trial and each study site	23
Statement of who will have access to the final trial dataset, and disclosure of contractual agreements that limit such access for investigators	19
Provisions, if any, for ancillary and post-trial care, and for compensation to those who suffer harm from trial participation	supplementary PIS
Plans for investigators and sponsor to communicate trial results to participants, healthcare professionals, the public, and other relevant groups (eg, via publication, reporting in results databases, or other data sharing arrangements), including any publication restrictions	19

For peer review only

Authorship eligibility guidelines and any intended use of professional writers	n/a
Plans, if any, for granting public access to the full protocol, participant- level dataset, and statistical code	19
Model consent form and other related documentation given to participants and authorised surrogates	supplementary materials
Plans for collection, laboratory evaluation, and storage of biological specimens for genetic or molecular analysis in the current trial and for future use in ancillary studies, if applicable	15

For peer review only

Missing

For peer review only

For peer review only

For peer review only

form

For peer review only

For peer review only

TIDieR
Template for Intervention Description and Replication

The TIDieR (Template for Intervention Description and Replication) Checklist*:

Information to include when describing an intervention and the location of the information

Item number	Item	Where located ** Primary paper (page or appendix number)	Other † (details)
	**BRIEF NAME**		
**1.**	Provide the name or a phrase that describes the intervention.	2	
	**WHY**		
**2.**	Describe any rationale, theory, or goal of the elements essential to the intervention.	4-5	______________
	**WHAT**		
**3.**	Materials: Describe any physical or informational materials used in the intervention, including those provided to participants or used in intervention delivery or in training of intervention providers. Provide information on where the materials can be accessed (e.g. online appendix, URL).	12, 15	_appendix____
**4.**	Procedures: Describe each of the procedures, activities, and/or processes used in the intervention, including any enabling or support activities.	6-12	______________
	**WHO PROVIDED**		
**5.**	For each category of intervention provider (e.g. psychologist, nursing assistant), describe their expertise, background and any specific training given.	1, 7-12, 17-18	______________
	**HOW**		

For peer review only

**6.**	Describe the modes of delivery (e.g. face-to-face or by some other mechanism, such as internet or telephone) of the intervention and whether it was provided individually or in a group.	6-18	______________
	**WHERE**		
**7.**	Describe the type(s) of location(s) where the intervention occurred, including any necessary infrastructure or relevant features.	6-18	______________
	**WHEN and HOW MUCH**		
**8.**	Describe the number of times the intervention was delivered and over what period of time including the number of sessions, their schedule, and their duration, intensity or dose.	6-18	______________
	**TAILORING**		
**9.**	If the intervention was planned to be personalised, titrated or adapted, then describe what, why, when, and how.	6-18	______________
	**MODIFICATIONS**		
**10.‡**	If the intervention was modified during the course of the study, describe the changes (what, why, when, and how).	N/A	______________
	**HOW WELL**		
**11.**	Planned: If intervention adherence or fidelity was assessed, describe how and by whom, and if any strategies were used to maintain or improve fidelity, describe them.	13-14	______________
**12.‡**	Actual: If intervention adherence or fidelity was assessed, describe the extent to which the intervention was delivered as planned.	N/A	______________

For peer review only

** **Authors** - use N/A if an item is not applicable for the intervention being described. **Reviewers** – use '?' if information about the element is not reported/not sufficiently reported.

† If the information is not provided in the primary paper, give details of where this information is available. This may include locations such as a published protocol or other published papers (provide citation details) or a website (provide the URL).

‡ If completing the TIDieR checklist for a protocol, these items are not relevant to the protocol and cannot be described until the study is complete.

* We strongly recommend using this checklist in conjunction with the TIDieR guide (see *BMJ* 2014;348:g1687) which contains an explanation and elaboration for each item.

* The focus of TIDieR is on reporting details of the intervention elements (and where relevant, comparison elements) of a study. Other elements and methodological features of studies are covered by other reporting statements and checklists and have not been duplicated as part of the TIDieR checklist. When a **randomised trial** is being reported, the TIDieR checklist should be used in conjunction with the CONSORT statement (see www.consort-statement.org) as an extension of **Item 5 of the CONSORT 2010 Statement.** When a **clinical trial protocol** is being reported, the TIDieR checklist should be used in conjunction with the SPIRIT statement as an extension of **Item 11 of the SPIRIT 2013 Statement** (see www.spirit-statement.org). For alternate study designs, TIDieR can be used in conjunction with the appropriate checklist for that study design (see www.equator-network.org).

For peer review only

For peer review only - http://bmjopen.bmj.com/site/about/guidelines.xhtml

BMJ Open

Manchester Intermittent Diet in Gestational Diabetes Acceptability Study (MIDDAS-GDM): A Two-Arm Randomised Feasibility Protocol Trial of an Intermittent Low-Energy Diet (ILED) in women with Gestational Diabetes and Obesity in Greater Manchester

Journal:	*BMJ Open*
Manuscript ID	bmjopen-2023-078264.R4
Article Type:	Protocol
Date Submitted by the Author:	11-Jan-2024
Complete List of Authors:	Dapre, Elizabeth; Wythenshawe Hospital<br>Issa, Basil; Manchester University NHS Foundation Trust, Department of Endocrinology and Diabetes<br>Harvie, Michelle; University Hospital of South Manchester NHS Foundation Trust, Genesis prevention centre<br>Su, Ting-Li; University of Manchester, Dentistry<br>McMillan, Brian; The University of Manchester, Centre for Primary Care and Health Services Research<br>Pilkington, A.; Wythenshawe Hospital<br>Hanna, F.; University Hospitals of North Midlands NHS Trust<br>Vyas, Avni; Manchester Metropolitan University Faculty of Health Psychology and Social Care, Health Professionals<br>Mackie, S.; Wythenshawe Hospital<br>Yates, James; Manchester University NHS Foundation Trust<br>Evans, Benjamin; Manchester University NHS Foundation Trust<br>Mubita, Womba; Manchester University NHS Foundation Trust, Department of Endocrinology and Diabetes<br>Lombardelli, Cheryl; Manchester University NHS Foundation Trust
**Primary Subject Heading**:	Diabetes and endocrinology
Secondary Subject Heading:	Nutrition and metabolism, Obstetrics and gynaecology, Reproductive medicine
Keywords:	Diabetes in pregnancy < DIABETES & ENDOCRINOLOGY, DIABETES & ENDOCRINOLOGY, Obesity, NUTRITION & DIETETICS, Feasibility Studies, Maternal medicine < OBSTETRICS

SCHOLARONE™
Manuscripts

Manchester Intermittent Diet in Gestational Diabetes Acceptability Study (MIDDAS-GDM): A Two-Arm Randomised Feasibility Protocol Trial of an Intermittent Low-Energy Diet (ILED) in women with Gestational Diabetes and Obesity in Greater Manchester

Authors: Dapre, E., Issa, B., Harvie, M., Su, T., McMillan, B., Hanna, F., Pilkington, A., Vyas, A., Yates, J., Mackie, S., Evans, B., Mubita, W., Lombardelli, C.

Dr Elizabeth Dapre (corresponding author)	elizabeth.dapre@hotmail.com<br>c/o Michelle Harvie, The Nightingale Centre, Wythenshawe Hospital, Southmoor Rd, Manchester M23 9LT
Dr Basil Issa	Diabetes and Endocrinology, Wythenshawe Hospital, Manchester, UK
Dr Michelle Harvie	The Nightingale Centre, Wythenshawe Hospital, Manchester, UK
Dr Ting-Li Su	Division of Dentistry, The University of Manchester, UK
Dr Brian McMillan	Division of Population Health, The University of Manchester, UK
Prof Fahmy Hanna	Diabetes and Endocrinology, University Hospitals of North Midlands, UK
Dr Andrea Pilkington	Obstetrics, St Mary's Wythenshawe Hospital, Manchester, UK
Avni Vyas	Division of Dietetics, Manchester Metropolitan University, UK
Benjamin Evans	Division of Clinical Trials, Manchester Foundation Trust, UK
James Yates	Division of Clinical Trials, Manchester Foundation Trust, UK
Sarah Mackie	Division of Clinical Trials, Manchester Foundation Trust, UK
Womba Mubita	Division of Clinical Trials, Manchester Foundation Trust, UK
Cheryl Lombardelli	The Nightingale Centre, Wythenshawe Hospital, Manchester, UK

Word Count: 3572

Submitted to: British Medical Journal Open

Start date of trial: 15th November 2022 (trial ongoing)

Date of submission:

For peer review only

Manchester Intermittent Diet in Gestational Diabetes Acceptability Study (MIDDAS-GDM): A Two-Arm Randomised Feasibility Protocol Trial of an Intermittent Low-Energy Diet (ILED) in women with Gestational Diabetes and Obesity in Greater Manchester

Authors: Dapre, E., Issa, B., Harvie, M., Su, T., McMillan, B., Hanna, F., Pilkington, A., Vyas, A., Yates, J., Mackie, S., Evans, B., Mubita, W., Lombardelli, C.

Corresponding Author: elizabeth.dapre@nhs.net

Abstract (word count 298)

Introduction: The prevalence of gestational diabetes mellitus (GDM) is rising in the UK and is associated with maternal and neonatal complications. National Institute for Health and Care Excellence (NICE) guidance advises first line management with healthy eating and physical activity which is only moderately effective for achieving glycaemic targets. Approximately 30% of women require medication with metformin and/or insulin. There is currently no strong evidence base for any particular dietary regimen to improve outcomes in GDM. Intermittent low-energy diets (ILEDs) are associated with improved glycaemic control and reduced insulin resistance in type 2 diabetes (T2DM) and could be a viable option in the management of GDM. This study aims to test the safety, feasibility and acceptability of an ILED intervention amongst women with GDM compared to best National Health Service (NHS) care.

Method and analysis: We aim to recruit 48 women with GDM diagnosed between 24-28 weeks gestation from antenatal clinics at Wythenshawe and St Mary's hospitals, Manchester Foundation Trust, over 13 months starting in November 2022. Participants will be randomised (1:1) to ILED (2 low-energy diet days/week of 1000kcal and 5 days/week of the best NHS care healthy diet and physical activity advice) or best NHS care 7 days/week until delivery of their baby. Primary outcomes include uptake and retention of participants to the trial, and adherence to both dietary interventions. Safety outcomes will include birthweight, gestational age at delivery, neonatal hypoglycaemic episodes requiring intervention, neonatal hyperbilirubinaemia, admission to special care baby unit or neonatal intensive care unit, stillbirths, the percentage of women with hypoglycaemic episodes requiring

For peer review only

third-party assistance, and significant maternal ketonaemia (defined as ≥1.0mmol/L) Secondary outcomes will assess the fidelity of delivery of the interventions, and qualitative analysis of participant and healthcare professionals' experiences of the diet. Exploratory outcomes include the number of women requiring metformin and/or insulin.

Ethics and dissemination: Ethical approval has been granted by the Cambridge East Research Ethics Committee (22/EE/0119). Findings will be disseminated via publication in peer-reviewed journals, conference presentations, and shared with diabetes charitable bodies and organisations in the UK, such as Diabetes UK and the Association of British Clinical Diabetologists.

Trial Registration Number: NCT05344066

For peer review only

Strengths and limitations of this study

Strengths

- This mixed methods feasibility study includes both quantitative and qualitative evaluation of the acceptability of the dietary intervention
- This study has been informed by an experienced patient and public involvement and engagement group

Limitations

- This study involves a small sample size and is not powered to show efficacy of the intervention
- Women joining this study are likely to be highly motivated and adherence may not reflect that seen in the wider general population

INTRODUCTION (word count: 3539)

Background

In the UK up to 16% of pregnant women develop gestational diabetes (GDM) and the incidence is rising, in part due to increasing rates of obesity and maternal age(1,2). GDM is associated with maternal and neonatal complications (the risk increases with poor glycaemic control), including macrosomia, shoulder dystocia, caesarean-sections, neonatal hypoglycaemia and/or hyperbilirubinaemia, preterm delivery, preeclampsia, and stillbirth(2). Women who have had GDM have an estimated seven to ten-fold risk of developing type 2 diabetes (T2DM) later in life, and their children have a higher risk of developing adult obesity and T2DM(2–4).

Excessive weight gain in pregnancy is a particular problem for women with GDM(5). Harper *et al* demonstrated that, in women with GDM, every additional 1lb/week gained following diagnosis of GDM resulted in a 36-83% increased risk of pre-eclampsia, caesarean-section, macrosomia, and large for gestational age babies(5). Such studies highlight the importance of adequate weight control throughout pregnancy in women with GDM in order to reduce both maternal and neonatal complications.

First-line therapy for GDM is diet and physical activity. National Institute for Health and Care Excellence (NICE) guidance encourages a healthy diet with increased fruit

For peer review only

and vegetables, low-glycaemic index (GI) foods, reduced refined sugars, regular mealtimes and regular physical activity(6,7). These dietary measures fail to achieve glycaemic targets in ~30% of women who require medication with metformin and/or insulin(8). A range of dietary approaches have been studied including daily diets promoting low-GI diets (limiting refined and promoting complex carbohydrates), continuous modest energy-restriction (1800 Kcal/day), and low carbohydrate diets(9). There is currently no strong evidence base for any particular dietary regimen to improve outcomes in GDM.

Intermittent Low-Energy Diets (ILED)

The pathogenesis of GDM is strongly linked to obesity and chronic insulin resistance with many clinicians viewing GDM as a form of evolving T2DM. ILEDs typically include several days of a food based or meal replacement (e.g. drinks/bars) low-energy diet (650-1000kcal) diet, with a standard healthy (non-restrictive) diet recommended on the remaining days of the week. These diets are associated with significant reductions in weight, insulin resistance and hyperglycaemia in patients with prediabetes (HbA1c between 42-47mmol/mol, impaired glucose tolerance, or impaired fasting glycaemia), those with T2DM, and otherwise healthy subjects with overweight/obesity(10–17). These changes are equivalent to, or greater than, those achieved with standard daily energy restriction. A popular intermittent diet involves 2 consecutive or non-consecutive days/week of a low-energy diet (650-1000kcal) and 5 days of normal eating/week, known as the 5:2 diet. The Manchester Intermittent vs. Daily Diabetes App Study (MIDDAS), a study comparing an ILED and a continuous low-energy diet in T2D conducted in our unit, has shown the feasibility and safety of an ILED (800kcal for 2 days/week) in patients with T2DM and obesity, including those using insulin(18). At the end of the study approximately 70% of participants in the ILED group completed the study and achieved a 6% reduction of their baseline body weight. Forty two percent achieved an HbA1c of <48 mmol/mol(18). Given the strong overlap between GDM and T2DM, an ILED may be a promising dietary intervention for those with GDM.

A successful dietary approach to glycaemic control could empower women to take charge of the management of their GDM. Women with GDM are motivated to modify their diet driven by a desire to improve foetal outcomes(19–21).

For peer review only

Our Patient and Public Involvement and Engagement (PPIE) work indicates that women find the current National Institute for Health and Care Excellence (NICE) healthy eating guidance(6,7) confusing and vague. Our PPIE work has indicated that women are keen to try alternative dietary approaches, particularly if alternative diets are more effective in preventing the need to progress to medications such as metformin and insulin.

Aim

The aim of this trial is to test the safety, feasibility, and acceptability of an ILED in GDM to inform a future large-scale RCT.

METHODS

Trial Design

The study is a 28-week feasibility two-arm RCT in one NHS trust performed in patients with GDM and BMI ≥27.5 kg/m^2, or ≥25 kg/m^2 in high-risk minority ethnic groups (i.e. South Asian, Black African, African Caribbean) in Greater Manchester, between December 2022 and July 2024(22,23). There will be an embedded qualitative sub-study for participants and healthcare professionals. Due to the nature of the intervention, it will not be possible to blind the participants, clinicians, or study team to the treatment allocation after randomisation (the statistician and laboratory technicians will remain blinded).

Trial Setting and Recruitment

Participants will be recruited from antenatal clinics at Wythenshawe and St Mary's Hospitals, Manchester Foundation Trust (MFT) between November 2022 and December 2023. This is an urban area within Greater Manchester, and MFT serves patients from a wide range of minority ethnic and socio-demographic backgrounds. Women may self-refer to the antenatal clinic or be referred by their primary care team. Assessments will be carried out at MFT, or remotely if required by COVID-19 restrictions. The qualitative sub-study will be carried out at MFT, remotely, or at a location of the participant's choosing. We aim to recruit eligible participants over a period of 13 months. Potential participants will be given written information about the study and the opportunity to ask questions about the study prior to providing written consent (supplementary files 1 and 2).

For peer review only

Eligibility Criteria

<IMAGE ONE; figure1 inclusion exclusion jpg>

Participant Flow

Participants who fulfil the broad eligibility criteria will be notified about the trial by the GDM nurse/midwife at the time of their diagnosis. Those who are interested will be provided with a comprehensive patient information sheet (supplementary file 1) and more detailed eligibility screening questions. They will be asked to attend their first appointment having fasted for at least 6 hours and complete a four-day food diary (in line with our department's usual care). On attending their first routine clinic appointment, interested participants will receive further information from the research team. They will have the opportunity to ask questions, have their eligibility confirmed, and will be asked for their written consent to take part. Baseline assessments will be taken and participants will be randomised to their allocated treatment group using an online randomisation platform. Participant flow through the study is demonstrated in figure 2.

Sample Size

We plan to recruit 24 participants per study arm (n=48) which, when considering an estimated attrition rate of 15%, will provide complete outcome data on 40 participants(24–26). It has been estimated that 24 participants per group will be sufficient to determine study outcomes, in line with sample size recommendations for feasibility studies(27–29).

This number will allow us to enable estimation of recruitment/retention parameters with sufficient precision. For example, based on 40 completed participants, it will enable recruitment rates in the region of 25% to be estimated with an error of +/-13.42% at most; retention of 85% will be estimated with error of +/-11.07% at most. It is also sufficient for estimation of variability (e.g. standard deviation) in gestational weight gain and capillary glucose concentrations (proposed outcomes for the future definitive trial) with negligible bias (30).

For peer review only

Randomisation

The randomisation schedule will be independently set up and known only by the trial statistician. The trial statistician will be blinded to the participant's identity using "sealed envelope" software (https://www.sealedenvelope.com/). Randomisation will be carried out by generating an online pseudo-random list with random permuted blocks of varying size, known only to the statistician, and will be stratified for two variables:

- Age (18-35, >35 years)
- BMI (27.5-34.99kg/m^2 and >35kg/m2, >25-32.49kg/m^2 and >32.5kg/m^2 for high-risk minority ethnic groups (i.e. South Asian, Black African, African Caribbean)

These stratification variables have been chosen to reduce potential bias as we expect varying severity of GDM with increasing age and BMI, and possible differences in diet adherence(31).

Treatment to intervention and control groups will be allocated in a 1:1 ratio. A member of the research team who will be unaware of the randomisation algorithm (principal investigator, clinical research nurse, clinical research fellow or project manager) will trigger the randomisation procedure onsite; participants and clinicians will then be informed of the allocated treatment group. Clinicians will not be blinded due to the need to remain astute to safety, adherence, and side effects, requiring open and honest discussions with patients at each appointment. The statistician will remain blinded to treatment allocation until all outcome measures for all subjects have been collected.

Interventions

Study Arm 1: Best NHS Care Diet

All dietetic advice will be face to face or via video calls or the telephone. Participants will receive one to one personalised written and verbal advice from a dietitian to follow NICE diet and physical activity recommendations(6,7). Dietitians and midwives will receive training to ensure standardised delivery of information in clinic, and standardised patient information leaflets will be supplied to include information about increased fruit/vegetable intake, low-glycaemic index foods, and a reduction in free sugars. Information will include advice about the importance of regular meals; dietary advice aims to ensure that participants include at least 70g protein/28g fibre, and

For peer review only

predominantly mono- and polyunsaturated fats as per American Diabetes Association recommendations(32). Participants will be advised to be physically active, for example walking for 30 minutes after a meal. Participants will receive ongoing dietetic education and support every 2 weeks until delivery. This level of support is higher than typically provided in NHS GDM antenatal clinics due to limited resources but has been utilised to reflect best NHS care. They will receive suggested menus and recipes to follow the NICE recommended healthy diet for GDM. Participants will be asked to measure their capillary glucose four times each day and their ketones on two random (recorded) days of the week of their choosing (supplementary file 3).

Study Arm 2: Intermittent Low-Energy Diet (ILED)

Participants will receive the same level of dietetic support as the best NHS care group. They will be given advice on adopting an ILED which involves 2 non-consecutive low-energy diet days/week (1000kcal to include 100g low-GI carbohydrate and 70g of protein) and 5 days/week of the NICE healthy eating low-GI diet and physical activity recommended for the best NHS care group. The low-energy days involve women selecting a set number of portions of protein, carbohydrate, fat, fruit, vegetables, and dairy/dairy alternatives as described in previous studies(33). Each low-energy day includes ~210g of lean protein foods, 3-4 portions of wholegrain carbohydrates, 1x7g portion of fat, 5 portions of vegetables, 2 of fruit, and 3 of dairy/dairy alternatives. Food and drink will be self-selected and not provided by the study team. Participants will be provided with comprehensive food lists, advice on portion sizes for the low-energy days and suggested menus and recipes to follow for both the low-energy and NICE recommended healthy diet days (supplementary file 4). Both diets can be successfully adapted for people of different ethnicities and those following omnivorous, vegetarian and vegan diets. Participants will be asked to measure their capillary glucose four times each day and their ketones on (and the morning after) the two low-energy days (supplementary file 3).

<IMAGE 2: figure 2 flow jpg>

<IMAGE 3: figure 3 sched assessments jpg>

For peer review only

Outcomes

Primary outcomes

- Uptake rate measured as a percentage of eligible participants who consent to take part, including the proportion of women who were screened who did not meet the eligibility criteria, and the number of women who did not give consent to take part
- Recruitment rate measured as the number of eligible participants who consent to take part per month
- Retention rate measured as the number of randomised participants who complete the trial (those who attend the final visit) and the percentage of participants who attend all 8 visits
- Adherence to the dietary interventions assessed from self-reported adherence to the potential low-calorie days between randomisation and delivery
- Completion of self-assessed glucose and ketone readings assessed as a percentage of the required readings

- Safety outcomes:
    - Percentage of women following ILED/best NHS care with hypoglycaemia (episodes of blood glucose of <3.0mmol/mol) and hypoglycaemia requiring third-party assistance as measured by participants
    - Percentage of women who develop significant ketonaemia in both groups (defined as ≥1.0mmol/L) as measured by participants
    - Percentage of neonatal hypoglycaemic episodes requiring intervention (blood glucose checked 2- hours post-delivery and 2-hours thereafter for 12 hours according to local protocol), neonatal birth weight, gestational age at delivery, hyperbilirubinaemia/jaundice, and/or admission to Special Care Baby Unit or neonatal intensive care, and stillbirths
    - The incidence and rate of other adverse events (e.g. headaches, lethargy, constipation, or complications requiring hospital admission)

For peer review only

between the start of the trial intervention and delivery recorded as mild, moderate and severe, as defined by Common Terminology Criteria for Adverse Events version 5 (CTCAEv5)(34). Hospital admission for routine labour and delivery will not be classified as an adverse event.

Secondary outcomes

- Completeness of collection of trial endpoints including the percentage of completed weight measurements, 4-day food diaries, and International Physical Activity Questionnaire (IPAQ) scores
- Fidelity of delivery of the interventions will be measured through the number and modality of completed planned patient contacts, electronic and paper food diaries, and self-reported capillary glucose and ketone measurements
- Qualitative analysis of the acceptability and implementation of the interventions will be explored amongst a subset of participants (~10 in each group) and healthcare professionals through in-depth interviews

Exploratory outcomes

The following outcomes will be explored without statistical inference.

1. Maternal outcomes:
    - The percentage of women requiring metformin and/or insulin
    - Four-point capillary glucose profiles during third trimester (four times daily until delivery)
    - Change in fasting blood test results between baseline measurements, 36-37 weeks' gestation, and 12 weeks post-delivery (including oral glucose tolerance tests (OGTT)
    - Mode of delivery, development of preeclampsia, polyhydramnios (maximum liquor volume pool depth ≥8 cm)
    - Quality of life and health status questionnaires (WHOQoL-BREF and SF-36 questionnaires)(35,36)
2. Foetal outcomes:
    - Foetal weight
    - Gestational age at delivery

For peer review only

Measurements

The full schedule of assessments can be found in figure 3.

Physical measurements

Height, weight and blood pressure will be measured using standardised calibrated equipment in antenatal clinic.

Blood samples

Fasting venous blood samples will be collected to assess maternal HbA1c, fasting glucose, insulin, beta-hydroxybutyrate, liver function tests, free fatty acids, thyroid function tests, and full blood count. At the end of the study all samples will be disposed of in accordance with the Human Tissue Act (2004).

Questionnaires

Participants will be asked to complete four questionnaires at four time points throughout the trial (self-reported). Quality of life and health status will be assessed using the World Health Organisation Quality of Life Questionnaire (brief version) and the 36-Item Short Form Survey respectively(35,36). Physical activity will be measured using the International Physical Activity Questionnaire – Short Form, and diet quality will be assessed using the UK Diabetes and Diet Questionnaire(37,38). These questionnaires are self-reported by participants and have been chosen as they are widely used and validated tools.

Food Diaries

4-day dietary records will be completed using Libro (Nutritics Mobile Application) or paper food diaries, which will be entered into Nutritics software (Nutritics, Dublin, Ireland)(39). Participants who wish to use Libro will receive one to one training to use this by the study dietitian. Diaries will provide the research team with information about the intake of energy, carbohydrate, fat, protein, fibre, glycaemic index, and the timing of meals for participants in both groups. Participants will be asked what other

For peer review only

dietary modifications, if any, they have made at their fortnightly dietitian reviews to establish the adoption of any alternative dietary practices in the cohort.

Adverse Events

Participants in both groups will be asked about any adverse effects that they have experienced at each visit. These will include, but are not limited to, the potential effects of a low-energy diet, e.g. headache, lethargy, dizziness, constipation, indigestion, poor concentration, and hunger. Adverse events will be graded as per CTAEv5(34). Participants will be issued with a participation/emergency card with emergency contact details for the research team to be carried at all times and to be shown to the attending physician in case of emergency admission to hospital. All participants will be issued with clear instructions as to how to manage a hypoglycaemic and/or ketonaemic event (supplementary file 5).

Data management

Participant data will be anonymised and will be stored in line with the Medicines for Human Use (Clinical Trials) Amended Regulations 2006 and the Data Protection Act (2018) and archived in line with the Medicines for Human Use (Clinical Trials) Amended Regulations (2006) as defined in the MFT Clinical Trials Office Archiving SOP (11; Retention of Data, Off-Site Archiving, and Destroying Documents). Deidentified data will be stored in a study-specific Research Electronic Data Capture (REDCap) database. The sponsor will periodically audit the site study file, a sample of the case report form, consent forms, and source data, and check accuracy of the study database to ensure satisfactory completion.

Statistical methods

A statistical analysis plan specifying the full details of the primary and secondary outcomes, other variables, and methods, will be produced prior to trial analysis. The main analysis will be conducted via intention-to-treat population and will not undertake any significance tests. Descriptive, graphical (summary), and basic statistics (e.g. i. number, frequencies and percentages, ii. mean and standard deviation, or iii. median and quartiles as appropriate) will be presented as appropriate for each group respectively, for group difference jointly, and for each stratum. Per-protocol analysis will be considered as a secondary analysis. Levels of missing data will be investigated and used to inform future studies. No imputation will

For peer review only

be used. The end of study questionnaire will be analysed using appropriate descriptive statistics for closed questions and key themes will be extracted without formal analysis from open questions to inform future research.

Progression Criterion

The success of the feasibility trial will be defined by the progression criteria as outlined in table 1. Any concerns regarding a low retention rate will be discussed with the PPIE group. Interviews will include those who withdraw from the study to address potential reasons for withdrawal with the aim to improve retention in future.

	Feasible (green)	Feasible with modification of the protocol (amber)	Not feasible (red)
**Recruitment**	≥4 patients/month	>2 patients/month	≤2 patients/month
**Uptake to the feasibility study**	≥15%	10-15%	<10%
**Retention to the feasibility study**	>70%	50-70%	<50%
**Adherence to the ILED intervention**	>50% of the low-energy days completed (2/week between weeks 24-28 and delivery)	30-50% of the low-energy days completed (2/week between weeks 24-28 and delivery)	<30% of the low-energy days completed (2/week between weeks 24-28 and delivery)

Table 1: Trial progression criterion

Qualitative sub-study

Participants will be invited to take part in an optional qualitative sub-study at 11-13 weeks post-partum. Healthcare professionals delivering the interventions will also be invited to take part in this study.

We will undertake 11-12 semi-structured interviews with a subset of women from each group (ILED n=10 and best NHS Care n=10) at around 12 weeks post-delivery. The final sample size will be contingent on obtaining data saturation. We will also interview a sample of healthcare professionals (HCPs) involved in the delivery of care to study participants, including dieticians, obstetricians and midwives, including

For peer review only

those with leadership and clinical managerial roles. Sampling will be purposive, aiming to obtain women from a range of ethnic groups, ages, socioeconomic backgrounds, and self-reported engagement with the intervention. Participants and HCPs will be asked about their experiences and thoughts regarding the intervention, including motivating factors, and facilitators/barriers to engagement. Interviews will be conducted by a researcher from the University of Manchester/MFT who is independent from the research staff involved in the delivery and assessment of the programmes. Analysis will be conducted by two independent researchers at the University of Manchester/MFT using Braun and Clarke's thematic analysis approach to identify key issues around the acceptability, usefulness of the programmes, and feasibility of a subsequent trial(40). Analysis will be inductive: open-ended, exploratory, and driven by the data.

All participants will also be asked to complete an optional and anonymous end of study questionnaire developed by the study team at their post-partum visit (supplementary file 6). This will give participants the opportunity to feedback on their experience and will enable the study team to identify improvements to the design of a possible follow-up study.

Trial Steering Committee (TSC)

The trial steering committee will include an independent consultant endocrinologist, obstetrician, dietitian, and the patient representative. The committee will oversee the trial to ensure that it is carried out to the expected standards. The TSC will liaise with the CI to develop a schedule of meetings, proposed to occur every four months, with meetings to occur no less than annually. Minutes will be taken at TSC meetings and copies of the minutes will be filed in the Trial Master File; they will be shared with relevant stakeholders as appropriate.

Patient and public involvement

Patient and public involvement was actively sought throughout the planning and design of this trial and continues to form a key part of the trial as it progresses. The patient and public involvement and engagement (PPIE) group assisted in the development of all participant materials and provided valuable insight into the wording of participant information and acceptability of the proposed intervention. The

For peer review only

PPIE group will be updated as the trial progresses and a further focus group will be held to advise on the interview schedule and wording for the qualitative sub-study. The group will also be invited to aid in the development of summarising key findings for dissemination to relevant patient groups.

Ethics and dissemination

This study has been approved by the Cambridge East Research Ethics Committee and is sponsored by MFT. Findings will be disseminated via publication in peer-reviewed journals, conference presentations, and shared with diabetes charitable bodies and organisations in the UK, such as Diabetes UK and the Association of British Clinical Diabetologists. Anonymised data will be available upon formal request once the principal results of the study have been published. Planned modifications to the protocol will be approved by the research ethics committee before they are adopted into the study. An audit trail of ethical amendments and documentation will allow monitoring by the research team and external regulatory bodies.

This is the first study to assess the feasibility and safety of an ILED in GDM as compared to best NHS care. Given the increasing incidence of GDM and associated health risks this research is both pertinent and important. The study is not powered to show differences between ILED and best NHS care, however the planned quantitative and qualitative assessments will inform the feasibility of the programme and a future definitive trial.

For peer review only

Full References

1. The British Diabetic Association. Diabetes UK. [cited 2019 Nov 27]. What is gestational diabetes? Available from: https://www.diabetes.org.uk/diabetes-the-basics/gestational-diabetes

2. Kim C. Gestational diabetes: risks, management, and treatment options. Int J Womens Health. 2010 Oct 7;2:339–51.

3. Kim SY, Sharma AJ, Callaghan WM. Gestational diabetes and childhood obesity: what is the link? Curr Opin Obstet Gynecol. 2012 Dec;24(6):376–81.

4. Vounzoulaki E, Khunti K, Abner SC, Tan BK, Davies MJ, Gillies CL. Progression to type 2 diabetes in women with a known history of gestational diabetes: systematic review and meta-analysis. BMJ. 2020 May 13;m1361.

5. Harper L, Tita A, Biggio J. The Institute of Medicine Guidelines for Gestational Weight Gain after a Diagnosis of Gestational Diabetes and Pregnancy Outcomes. Am J Perinatol. 2014 Jun 27;32(03):239–46.

6. Webber J, Charlton M, Johns N. Diabetes in pregnancy: management of diabetes and its complications from preconception to the postnatal period (NG3). Br J Diabetes. 2015 Sep 1;15(3):107.

7. NHS. NHS Treatment Gestational Diabetes [Internet]. 2019 [cited 2020 Jan 10]. Available from: https://www.nhs.uk/conditions/gestational-diabetes/treatment/

8. Mayo K, Melamed N, Vandenberghe H, Berger H. The impact of adoption of the international association of diabetes in pregnancy study group criteria for the screening and diagnosis of gestational diabetes. Am J Obstet Gynecol. 2015 Feb;212(2):224.e1-9.

9. Han S, Middleton P, Shepherd E, Van Ryswyk E, Crowther CA. Different types of dietary advice for women with gestational diabetes mellitus. Cochrane Database Syst Rev. 2017 25;2:CD009275.

10. Carter S, Clifton PM, Keogh JB. Effect of Intermittent Compared With Continuous Energy Restricted Diet on Glycemic Control in Patients With Type 2 Diabetes: A Randomized Noninferiority Trial. JAMA Netw Open. 2018 Jul 20;1(3):e180756.

11. Antoni R, Johnston KL, Collins AL, Robertson MD. Effects of intermittent fasting on glucose and lipid metabolism. Proc Nutr Soc. 2017 Aug;76(3):361–8.

For peer review only

12. Cioffi I, Evangelista A, Ponzo V, Ciccone G, Soldati L, Santarpia L, et al. Intermittent versus continuous energy restriction on weight loss and cardiometabolic outcomes: a systematic review and meta-analysis of randomized controlled trials. J Transl Med. 2018 Dec;16(1):371.

13. Harris L, Hamilton S, Azevedo LB, Olajide J, De Brún C, Waller G, et al. Intermittent fasting interventions for treatment of overweight and obesity in adults: a systematic review and meta-analysis. JBI Database Syst Rev Implement Rep. 2018 Feb;16(2):507–47.

14. Harvie M, Howell A. Potential Benefits and Harms of Intermittent Energy Restriction and Intermittent Fasting Amongst Obese, Overweight and Normal Weight Subjects—A Narrative Review of Human and Animal Evidence. Behav Sci. 2017 Jan 19;7(4):4.

15. Harvie MN, Pegington M, Mattson MP, Frystyk J, Dillon B, Evans G, et al. The effects of intermittent or continuous energy restriction on weight loss and metabolic disease risk markers: a randomized trial in young overweight women. Int J Obes. 2011 May;35(5):714–27.

16. Wang X, Li Q, Liu Y, Jiang H, Chen W. Intermittent fasting versus continuous energy-restricted diet for patients with type 2 diabetes mellitus and metabolic syndrome for glycemic control: A systematic review and meta-analysis of randomized controlled trials. Diabetes Res Clin Pract. 2021 Sep;179:109003.

17. He S, Wang J, Zhang J, Xu J. Intermittent Versus Continuous Energy Restriction for Weight Loss and Metabolic Improvement: A Meta-Analysis and Systematic Review. Obesity. 2021 Jan;29(1):108–15.

18. McDiarmid S, Harvie M, Johnson R, Vyas A, Aglan A, Moran J, et al. Manchester Intermittent versus Daily Diet App Study (MIDDAS): A pilot randomized controlled trial in patients with type 2 diabetes. Diabetes Obes Metab. 2022 Mar;24(3):432–41.

19. Carolan M. Women's experiences of gestational diabetes self-management: A qualitative study. Midwifery. 2013 Jun;29(6):637–45.

20. Gao F, Luo H, Jones K, Nicholson W, Bell RA. Gestational Diabetes and Health Behaviors Among Women: National Health and Nutrition Examination Survey, 2007–2014. Prev Chronic Dis. 2018 Oct 25;15:180094.

21. McMillan B, Easton K, Goyder E, Delaney B, Madhuvrata P, Abdelgalil R, et al. Reducing risk of type 2 diabetes after gestational diabetes: a qualitative study to explore the potential of technology in primary care. Br J Gen Pract. 2018 Apr;68(669):e260–7.

22. National Institute for Health and Care Excellence. Diabetes in pregnancy: management from preconception to the postnatal period. NICE guideline. [Internet]. 2020. Available from: https://www.nice.org.uk/guidance/ng3/resources/diabetes-in-pregnancy-management-from-preconception-to-the-postnatal-period-pdf-51038446021

23. National Institute for Health and Care Excellence. Obesity: identification, assessment and management. NICE guideline. [Internet]. 2014 Nov [cited 2023 Aug 30]. Available

from: https://www.nice.org.uk/guidance/cg189/resources/obesity-identification-assessment-and-management-pdf-35109821097925

24. H. Al Wattar B, Dodds J, Placzek A, Beresford L, Spyreli E, Moore A, et al. Mediterranean-style diet in pregnant women with metabolic risk factors (ESTEEM): A pragmatic multicentre randomised trial. Persson LÅ, editor. PLOS Med. 2019 Jul 23;16(7):e1002857.

25. Moreno-Castilla C, Hernandez M, Bergua M, Alvarez MC, Arce MA, Rodriguez K, et al. Low-carbohydrate diet for the treatment of gestational diabetes mellitus: a randomized controlled trial. Diabetes Care. 2013 Aug;36(8):2233–8.

26. Rae A, Bond D, Evans S, North F, Roberman B, Walters B. A randomised controlled trial of dietary energy restriction in the management of obese women with gestational diabetes. Aust N Z J Obstet Gynaecol. 2000 Nov;40(4):416–22.

27. Julious SA. Sample size of 12 per group rule of thumb for a pilot study. Pharm Stat. 2005 Oct;4(4):287–91.

28. Sim J, Lewis M. The size of a pilot study for a clinical trial should be calculated in relation to considerations of precision and efficiency. J Clin Epidemiol. 2012 Mar;65(3):301–8.

29. Lancaster GA. Pilot and feasibility studies come of age! Pilot Feasibility Stud. 2015 Dec;1(1):1, 2055-5784-1–1.

30. Teare MD, Dimairo M, Shephard N, Hayman A, Whitehead A, Walters SJ. Sample size requirements to estimate key design parameters from external pilot randomised controlled trials: a simulation study. Trials. 2014 Dec;15(1):264.

31. Makgoba M, Savvidou M, Steer P. An analysis of the interrelationship between maternal age, body mass index and racial origin in the development of gestational diabetes mellitus: Gestational diabetes mellitus and risk factors. BJOG Int J Obstet Gynaecol. 2012 Feb;119(3):276–82.

32. American Diabetes Association. 13. Management of Diabetes in Pregnancy: *Standards of Medical Care in Diabetes—2018*. Diabetes Care. 2018 Jan;41(Supplement 1):S137–43.

33. Harvie M, Wright C, Pegington M, McMullan D, Mitchell E, Martin B, et al. The effect of intermittent energy and carbohydrate restriction *v* . daily energy restriction on weight loss and metabolic disease risk markers in overweight women. Br J Nutr. 2013 Oct 28;110(8):1534–47.

34. National Cancer Institute. Cancer Therapy Evaluation Program. 2017 [cited 2020 May 7]. Common Terminology Criteria for Adverse Events (CTCAE). Available from: https://ctep.cancer.gov/protocolDevelopment/electronic_applications/ctc.htm#ctc_50

35. World Health Organisation. WHOQOL: Measuring Quality of Life [Internet]. Available from: https://www.who.int/tools/whoqol

For peer review only

36. Nair R, Kachan P. Outcome tools for diabetes-specific quality of life: Study performed in a private family practice clinic. Can Fam Physician Med Fam Can. 2017 Jun;63(6):e310–5.

37. Ekelund U, Sepp H, Brage S, Becker W, Jakes R, Hennings M, et al. Criterion-related validity of the last 7-day, short form of the International Physical Activity Questionnaire in Swedish adults. Public Health Nutr. 2006 Apr;9(2):258–65.

38. England CY, Thompson JL, Jago R, Cooper AR, Andrews RC. Development of a brief, reliable and valid diet assessment tool for impaired glucose tolerance and diabetes: the UK Diabetes and Diet Questionnaire. Public Health Nutr. 2017 Feb;20(2):191–9.

39. Nutritics [Internet]. [cited 2020 Jul 12]. Available from: https://www.nutritics.com/p/home?utm_source=adwords&utm_campaign=Nutritics+Brand&utm_term=&utm_medium=ppc&hsa_grp=78385303355&hsa_mt=b&hsa_kw=&hsa_src=g&hsa_ad=380634402696&hsa_cam=1619635404&hsa_net=adwords&hsa_acc=2361676125&hsa_ver=3&hsa_tgt=dsa-814211721343&gclid=Cj0KCQjw6ar4BRDnARIsAITGzlAm_uy2YNRBU5N-egXXyehUQ_9CHKylLQI_LgCYJK9Jpet4oIvUM5saAkzREALw_wcB

40. Braun V, Clarke V. Successful qualitative research: a practical guide for beginners. Los Angeles: SAGE; 2013. 382 p.

Authors' contributions

Michelle Harvie, Basil Issa and Elizabeth Dapre designed the study, wrote the protocol and secured funding. Brian McMillan designed and worded the qualitative sub-study. Ting-Li Su developed the statistical analysis plan. Fahmy Hanna reviewed and advised on overall study design. Andrea Pilkington provided expert obstetric guidance for the protocol design. Avni Vyas, Cheryl Lombardelli and Michelle Harvie conducted dietetic reviews. Womba Mubita helped with recruitment and review of participants. James Yates and Benjamin Evans were responsible for project management and data reporting. Sarah Mackie coordinated the clinical trial. Elizabeth Dapre drafted the manuscript for publication, with input from Michelle Harvie, Basil Issa, Brian McMillan and Ting-Li Su. All authors have proofed and checked the manuscript.

Acknowledgments

For peer review only

With thanks to Mrs Rebecca Lumsden, our patient expert, whose insight has been invaluable in the design of the study. We are most grateful to Mrs Jodie Aspinall, and Miss Lisa Brew-Butler, specialist midwives who continue to help identify suitable candidates for the study, and Jessy Athirampuzha who has played a critical role in the recruitment and follow up of participants throughout the trial.

Funding statement:

This trial is funded by the National Institute for Health Research (NIHR201944) and sponsored by Manchester University NHS Foundation Trust (MFT). The funders of the study had no role in the study design or writing of the report. Dr Dapre is an NIHR sponsored GP academic clinical fellow.

Competing interests statement.

Michelle Harvie has co-authored three self-help books for the public to follow intermittent diets. All author proceeds are paid directly to the charity Prevent Breast Cancer (registered charity number 1109839) to fund breast cancer research.

For peer review only

Appendix

1.0 Patient Information Sheet (supplementary document 1)

2.0 Consent Form (supplementary document 2)

3.0 Self-monitoring schedule for capillary glucose and ketone monitoring (supplementary document 3)

4.0 Intermittent Low Energy Diet Day Example (supplementary document 4)

5.0 Medical Management Protocols (supplementary document 5)

6.0 End of Study Questionnaire (supplementary document 6)

LEGENDS

- <image 1>: Figure 1: Inclusion and exclusion criteria
- <image 2>: Figure 2: Participant flow through trial
- <image 3>: Figure 3: Schedule of assessments

For peer review only

Inclusion Criteria

- Pregnant women ≥18 years
- BMI of ≥27.5kg/m2 or a BMI ≥25 kg/m^2 in high risk minority ethnic group (i.e. South Asian, Black African, African Caribbean) and <50 kg/m2 at booking appointment (8-12 weeks' gestation)
- Newly diagnosed GDM according to local diagnostic criteria (fasting glucose ≥5.3mmol/l and/or 2-hour postprandial glucose ≥8.5mmol/l in a 75g OGTT) scheduled to receive first line diet and physical activity (best NHS care)
- 24-30 weeks' pregnant at screening appointment

Exclusion Criteria

- Pregestational type 1 or type 2 diabetes.
- Fasting glucose of ≥7 or 2-hour postprandial of ≥11 on OGTT (immediate intervention with medication would be required in this group of women)
- Current multiple pregnancy
- Maturity Onset Diabetes of the Young (MODY)
- Significant comorbid disease that in PI's opinion would preclude participation in the study e.g. chronic kidney disease, significant cardiac disease, significant history of disordered eating or severe psychological problems.
- Current participation in a GDM medication treatment trial
- People who are not capable of providing informed consent or adhering to the monitoring and safety protocols
- People who have previously had bariatric surgery for weight loss including gastric bypass and sleeve gastrectomy, and/or those prescribed weight loss medications (e.g. orlistat).
- Medications at the time of the OGTT that may interfere with results (e.g. high dose oral steroids, immunosuppressants)
- Previous history of intrauterine growth restriction
- Women who have lost more than 5% of their weight from booking appointment to screening appointment.

Figure 1: Inclusion and exclusion criteria

159x144mm (220 x 220 DPI)

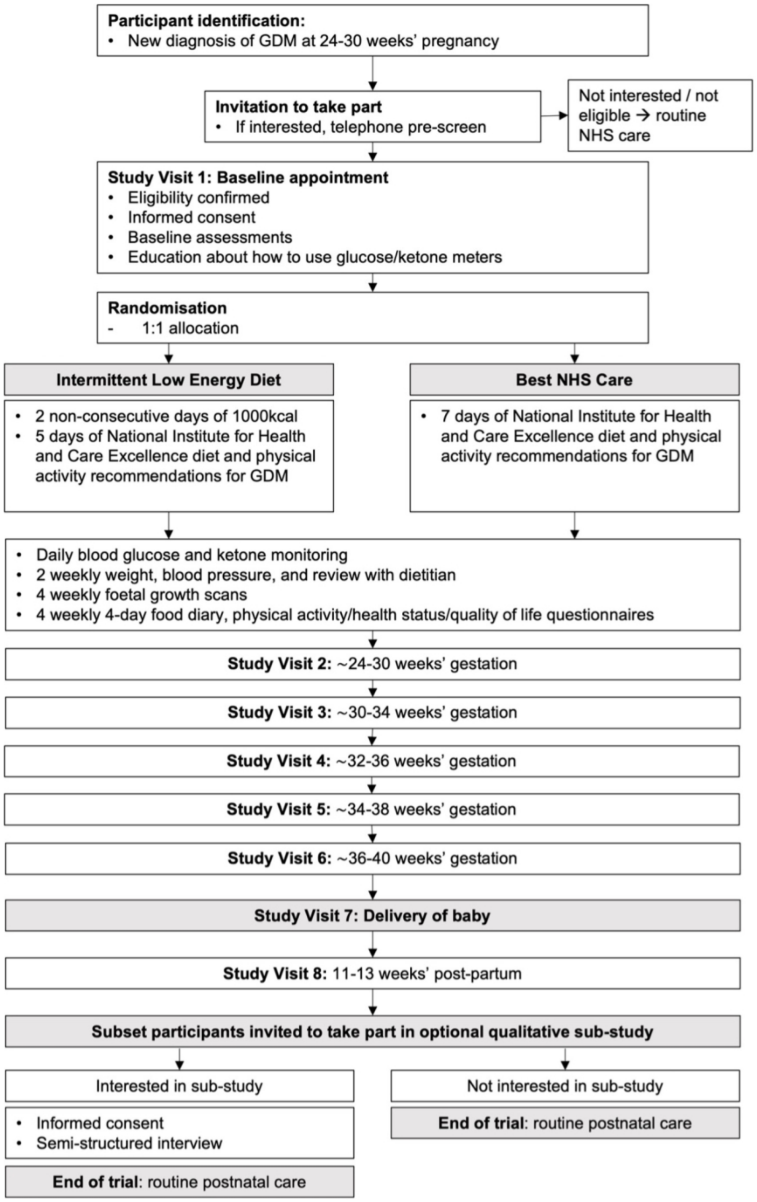

Figure 2: Participant flow through trial

154x245mm (220 x 220 DPI)

	Study Visit							
	1	2	3	4	5	6	7	8
Gestation (weeks)	~24-30	~24-30	~30-34	~32-36	~34-38	~36-40	delivery	11-13 post-partum
Eligibility confirmed	X							
Informed consent	X							
Randomisation	X							
Tailored dietitian review (face to face or remote)	X	X	X	X	X	X		X
Height	X							
Weight ^	X	X	X	X	X	X	X	X
Blood Pressure ^	X	X	X	X	X	X	X	X
Fasting blood sample *	X				X			X
Questionnaires #	X		X		X			X
4-day food diary		X			X			X
Foetal growth scan	X		X		X			
Review of glucose and ketone measurements		X	X	X	X	X		
Neonatal measurements §							X	
Oral glucose tolerance test								X
Exit interview / end of study questionnaires								X
Invitation to optional qualitative sub-study $								X

^ Frequency of assessment will be 2-4 weekly depending on whether appointment is face-to-face or virtual due to COVID-19
* Fasting bloods: urea and electrolytes, liver function tests, bone profile, lipids, thyroid function tests, HbA1c, beta-hydroxybutyrate, free fatty acids, full blood count, fasting glucose, insulin
# Questionnaires: World Health Organisation Quality of Life (brief version), 36-Item Short Form Survey, International Physical Activity Questionnaire (short form), UK Diabetes and Diet Questionnaire
§ Neonatal measurements include gestational age at delivery, mode of delivery, and neonatal weight
$ Sub-study involves semi-structured interviews exploring thoughts and experiences of the trial

159x239mm (72 x 72 DPI)

Manchester University NHS Foundation Trust

The University of Manchester

NIHR | National Institute for Health Research

Consultant Endocrinologist – Dr. Basil Issa
Research Dietitian – Dr. Michelle Harvie
Email: mft.middas.gdm@nhs.net
Tel: 07815987910

1st Floor Education & Research Centre
Manchester University NHS Foundation Trust
Wythenshawe Hospital
Manchester
M23 9LT

MIDDAS-GDM

Manchester Intermittent Diet in Gestational Diabetes Acceptability Study

Participant information sheet

We would like to invite you to take part in a research study **that is testing two different diet programmes which aim to help people with gestational diabetes control their blood sugars.**

If you decide to take part:

- You will be assigned to one of two diet programmes for the duration of your pregnancy. One involves following the standard NHS healthy diet recommendations for pregnancy, and the other follows the standard NHS healthy diet for 5 days/week plus two non-consecutive calorie restricted days of 1,000 kcal per week (both groups will be encouraged to be physically active).

- You will be asked to attend your routine appointments at Wythenshawe or St Marys Hospital and will have fortnightly appointments until delivery of your baby (some appointments may be virtual depending on COVID-19 restrictions). You will be asked to attend the hospital for a blood test 12 weeks after having your baby.

- You will be supported by a diabetes specialist dietitian, midwife, consultant endocrinologist, and your obstetric team throughout the study to help manage your pregnancy and blood glucose safely.

- Throughout the study you will be asked to monitor your food intake via a smartphone/tablet app, or on paper if you prefer, and you will receive feedback on this during your dietary reviews. Comprehensive dietary advice and recipes will be provided.

- Throughout the study you will be asked to monitor your blood sugar using a blood sugar meter four times a day, and you will also be asked to monitor your ketone levels two times on two days of the week (ketones indicate how well your body is using sugar or fat as an energy source). You will be taught how to check your blood sugar and ketone levels.

- If you would like to take part, or you have any questions, then please contact **mft.middas.gdm@nhs.uk**

For peer review only - http://bmjopen.bmj.com/site/about/guidelines.xhtml

Manchester University NHS Foundation Trust

MANCHESTER 1824 The University of Manchester

NIHR | National Institute for Health Research

This study is being carried out by a team of trained dietitians, doctors, nurses, midwives and researchers under the supervision of Dr. Basil Issa and Dr. Michelle Harvie at Wythenshawe and St Marys hospitals (Manchester University NHS Foundation Trust, MFT).

Before you decide if you would like to take part, it is important for you to understand why the research is being done and what taking part would involve for you. Please take your time to read the following information carefully. Discuss it with your friends, relatives, or GP if you wish to. Take time to consider whether or not you wish to take part.

Please ring the research team at the number at the top of the first page, or e-mail mft.middas.gdm@nhs.net if there is anything that is not clear, or if you would like more information. You can attend an information session about the diets and the study before agreeing to take part if you would like to.

Your participation in the study is entirely voluntary; you do not have to take part if you do not want to and you can opt out of the study at any time without giving a reason. Thank you for reading this information. We hope this research will be of interest to you.

Why are we doing this research?

Around 1 in 8 pregnant women can develop gestational diabetes. This condition causes risks to mother and baby from high blood sugar, high blood pressure, induced labours, caesarean-sections, and larger babies. Women often need medication to control blood sugar despite following recommended NHS healthy eating plans for pregnancy. Intermittent low-calorie diets (two non-consecutive days over the course of the week) improve blood sugar control and reduce the need for medication in patients with type 2 diabetes. We want to find out whether intermittent low-calorie diets might also improve blood sugar control in gestational diabetes and reduce the need for medication as it is a similar condition to type 2 diabetes.

What is the purpose of this research?

This study aims to assess the acceptability (to you) and safety of an intermittent low-calorie diet compared to the usual recommended NHS healthy eating and lifestyle plan for gestational diabetes. **A computer system will randomly allocate you to one of the two diets**. We want to find out which diet is most acceptable to women, whether there is any difference in the two diets' effect on blood sugar control, and any side effects experienced by women. The findings of this study will inform a larger study which will be designed to more closely compare the effect of the two diets on blood sugar control in women with gestational diabetes.

Why have I been asked to take part?

You have been invited to take part in this study because you have been diagnosed with gestational diabetes. We hope to recruit around 48 people to take part in this study.

For peer review only - http://bmjopen.bmj.com/site/about/guidelines.xhtml

Manchester University NHS Foundation Trust

MANCHESTER 1824 The University of Manchester

NIHR | National Institute for Health Research

What happens if you agree to take part?
If you agree to take part you will be randomly allocated via a computer system to one of two diet and lifestyle programmes for the remaining weeks of your pregnancy.

Best NHS Care Healthy Diet programme
You will receive personalised advice from a specialist dietician. Recommendations will include increased fruit/vegetable intake, low glycaemic index starchy foods (i.e starchy foods which are slowly absorbed and take a while to raise your blood sugar level), reducing refined sugar, and having regular mealtimes. You will be advised how to design your diet to include the right amount of protein, fats, carbohydrates, and fibre, and will be given meal plans and recipes. You will also be advised to try to complete 150 minutes of moderate intensity exercise a week.

Intermittent Low-Calorie Diet programme
If you are allocated to this group you will receive personalised advice to follow a low-calorie diet of 1,000 kcal on two non-consecutive days of the week and the NHS healthy diet on the other five days of the week. The 1,000 kcal days include a set number of portions of protein, carbohydrates and fat foods, fruits, vegetables and dairy/dairy alternatives typically including ~210g (7 oz) of lean protein foods and 3-4 portions of wholegrain carbohydrates, 5 portions of vegetables, 2 of fruit, and 3 of dairy or dairy alternatives and a small amount of healthy fat. You will also be advised to try to complete 150 minutes of moderate intensity exercise a week.

Monitoring
You will have all your usual routine antenatal appointments including checks on your weight, blood pressure, blood tests and ultrasound scans. Extra blood tests will be done as part of the study, and these will be added on to samples taken during your routine blood tests.

You will be asked to monitor your blood sugar at home four times a day until your baby is born which is usual care in the clinic. In addition, you will be asked to record ketone levels on two days of the week (you will be taught how to do this using a finger prick machine). The results will be recorded when you attend clinic.

When babies are born to mothers with gestational diabetes it is normal that their birth weight is recorded and that their blood sugar is monitored for 12 hours following delivery; these results will be recorded by the research team.

You will be asked to attend an additional glucose tolerance test at the hospital 12 weeks after delivery to assess whether you have any residual diabetes (95% of women do not) and also to assess how sensitive your body is to insulin (an important risk factor for the development of diabetes in the future). You will be asked to attend at 9:00am having fasted (no food or drink apart from water) from midnight. A blood sample (around 10 mL/2 teaspoons) will be taken for glucose and insulin and you will be asked to drink a sugary drink with 75 grams of glucose. A further blood sample will be taken after 2 hours for glucose and insulin. You will need to

For peer review only - http://bmjopen.bmj.com/site/about/guidelines.xhtml

remain in hospital during this time. The reason for this is to help us to understand whether there could be any difference in the body's ability to process sugar between the two diet groups, and also to find out whether any women still have signs of diabetes after pregnancy.

Any blood samples taken as part of the study will be identifiable only using your study identification number and will have none of your personal details. Part of the sample will be sent for immediate analysis and any remaining will be stored securely, accessible only by the research team. At the end of the study any left over samples will be disposed of in accordance with the Human Tissue Act (2004).

You will be asked to record your food intake via the Libro smartphone/tablet app or in a paper diary for four days once a month throughout the study. You will also be asked to complete three questionnaires to assess your wellbeing and level of physical activity in these weeks, and a final end of study questionnaire at the final appointment.

Ongoing support from a specialist team of healthcare professionals
Your specialist team includes a Consultant Endocrinologist, Consultant Obstetrician, diabetes specialist dietitian, midwives, and a GP trainee with a special interest in women's health. The specialist team work closely with the usual obstetric teams involved in your care. Reviews with the team will be either face to face when you attend clinic or remotely using video calls.

Mobile Applications and Glucose Meters
You will be given the option of using a smartphone application called 'Libro' to record your dietary intake during the study. We will ask you to record 4 days of food and drink intake once a month across the study. Your diaries will be viewed by your allocated dietitian who will provide personalised dietary feedback via the app. You are also free to record more days of your diet should you wish, which some people find helpful. If you do not want to use the mobile app you can use paper instead. You will be supported to set up and use the Nutritics Libro App at your appointments. You do not have to use the application to be part of the study.

Your blood sugar will be monitored using a glucose monitoring device which checks your blood sugar using a 'fingerprick' blood test. You will be shown how to do this yourself. With your permission the research team will make a note of your glucose readings at every visit, either by checking your glucose monitoring device, or by uploading your glucose meter readings onto the computer if you are using a mobile application.

The self-monitoring schedule is as follows:

**Intermittent low-energy diet monitoring**		**Best NHS care monitoring**	
Ketones (low kcal days)	Glucose	Ketones (2 days/wk)	Glucose
Fasting before breakfast the morning after each of the low-calorie days	Fasting (morning)	Fasting before breakfast on 2 non-consecutive days/weeks	Fasting (morning)
	1hr post breakfast		1hr post breakfast
	1hr post lunch		1hr post lunch

For peer review only - http://bmjopen.bmj.com/site/about/guidelines.xhtml

Manchester University NHS Foundation Trust

The University of Manchester

NIHR | National Institute for Health Research

1 hour post evening meal on each of the low-calorie days	1hr post dinner	1 hour post evening meal on 2 non-consecutive days/week	1hr post dinner

What should I do if my blood glucose or ketones are out of range?

Low Blood Sugar

It is unlikely that your blood sugar levels will drop too low as a result of being on the intermittent low calorie or the NHS standard diets. You are advised to take 15-20g of 'rapid acting' carbohydrate if your blood glucose is <4 mmol/L). Examples include 170-225ml Lucozade Original (not Lucozade Sport), a small carton of fruit juice, 5-6 glucose tablets, 4/5 jelly babies, or a small tin of cola (150-200ml). You will need to repeat the treatment every 15 minutes until your blood glucose is ≥4 mmol/l.

The following table highlights when you need to consider an additional slower acting carbohydrate:

Situation	Acceptable slow acting carbohydrate
Less than 1 hour before the next meal	Try and avoid
1-2 hour before the next meal	10g (eg half of one of the items below)
More than 2 hours before the next meal	15-20g (eg slice of toast, piece of fruit, small bowl of cereal, glass of milk)

Raised Ketones

It is unlikely that your ketone levels will rise significantly as a result of being on the intermittent low calorie or NHS standard diets.

If your ketone levels are ⩾1.0 mmol/L on a fasting sample:

- Drink 1L fluids and repeat ketone levels after 4 hours.
- If your ketone level has improved (<1.0mmol/L), no further action is required.
- If your ketone level has increased or remains the same, repeat your ketone level after 2 hours.
- If your ketone level is persistently increased, consume 40g carbohydrates (eg one bagel, bowl of cereal and a banana, small jacket potato), and repeat in 2 hours.
- Continue to do this until your ketone levels are <1.0mmol/L.

Make Immediate Contact with the research team if:

Blood glucose	• Your blood glucose is <3.0 mmol/l or you have symptoms requiring medical attention which are thought to be due to low blood glucose,<br>• Your fasting blood glucose is >5.2 mmol/L on more than a quarter of your measurements on two days in a row,<br>• Your 1 hour post-meal blood glucose is >7.7 mmol/L on more than a quarter of your measurements on two days in a row

For peer review only - http://bmjopen.bmj.com/site/about/guidelines.xhtml

Ketones	• Your blood ketones are >1.0 mmol/L

Will I need medications?
If your blood sugars are found to be high despite following the recommended diet and lifestyle programmes you may be advised to start medication to help control your blood sugar. You will be advised on changes to your medications by a diabetes specialist nurse/diabetes midwife and also a Consultant Endocrinologist if required. This is usual practice regardless of whether you are taking part in the study.

What care will I receive after the study has stopped?
At the end of the study, you will be provided appropriate ongoing dietary advice from the study dietitian following your final glucose tolerance test to follow the NHS healthy eating and lifestyle plan. You will receive routine postnatal care from your GP, hospital team, and dietitian if required. You will be advised to see your GP for an annual blood test to check your blood sugar levels (this is routine care for women with gestational diabetes). Approximately 5% of women with gestational diabetes have residual diabetes after delivery. This will be identified from your glucose tolerance test/HbA1c; if this is the case you and your GP will be informed. Your GP will take over the management of your diabetes as per routine care outside the study.

Interview sub study
Women in this study may be invited to take part in an interview at the end of the study . You will be asked about your views and experiences on trying to follow your allocated diet programme. This interview can be arranged at a time that suits you, either at Wythenshawe or St Marys Hospital, at your home, or over the telephone. There is no obligation to take part in this interview study.

Frequently asked questions

Do I have to take part?
No, you do not have to take part if you do not wish to and your decision will not affect any standard of care you receive at Wythenshawe or St Marys hospitals (Manchester University NHS Foundation Trust, MFT).

What happens if I change my mind?
It is OK if you agree to take part in the study but later change your mind. You do not need to give a reason and it will not affect the standard of care you receive. The study team may also choose to withdraw you if it is necessary for your health or safety due to unexpected findings during the study. If you decide to withdraw from the study, or the study is stopped for any reason, you will be asked whether or not you are happy for us to keep the data that may have already been collected. If you do withdraw from the study you will continue to be cared for by your usual specialist diabetes and obstetric teams for the duration of your pregnancy. You will still have the option of completing the end of study questionnaire and/or interview to provide feedback; this is very useful for the research team to help us understand potential reasons you may have chosen to withdraw from the study.

For peer review only - http://bmjopen.bmj.com/site/about/guidelines.xhtml

You will also have the option that if you withdraw, researchers may still collect relevant information about your pregnancy and/or gestational diabetes from your medical records within the 18-month study duration. This will be an option on the consent form.

Are there any benefits from taking part?
You will receive frequent personalised advice and support to follow two diet and lifestyle programmes which may help to control your blood sugar levels throughout your pregnancy. The information gained from this study will also help inform the future NHS care of patients with gestational diabetes.

Are there any risks from taking part?
Research has found that diets consisting of two low-calorie days a week are very low risk. Pregnant women will develop slightly higher levels of ketones when following low calorie diets than women who are not pregnant. Ketones are produced naturally by the body when the body uses fat stores for energy (i.e. when we follow a low calorie diet or haven't eaten enough because we are ill).

Some research suggests that very high levels of ketones throughout pregnancy may cause a higher risk of babies being slightly smaller than average. It is very unlikely that you will develop high levels of ketones by following this diet. You will be provided with a ketone meter, and you will be asked to check your ketone levels after an evening meal on your low-calorie day, and the following morning, to make sure that your ketone levels are normal.

On your low-calorie days you may feel slightly more hungry, or you may experience other effects such as increased nausea, light headedness, or tiredness. It is important that you eat regularly throughout the day to reduce the risk of this happening. You will be asked to report any side effects of following the diet to the team at each appointment.

What happens if my baby or I become unwell during the study?
The safety of you and your baby are of utmost importance and remain our priority. In the instance that either of you become unwell your case will be reviewed by our specialist team and your suitability for continuing in the trial will be decided. Although it remains exceptionally rare, were you to experience the unexpected loss of your baby you will be withdrawn from the trial and supported by the dedicated specialist bereavement team at the hospital. Any information which has been collected as part of the trial will be stored securely and once we have finished the study we will keep some of the data so we can check the results. We will write our reports in a way that no-one can work out that you took part in the study.

What will happen to blood samples which are taken?
Some blood samples taken as part of the study will be sent to the laboratory immediately for analysis and any remaining will be stored securely for the duration of the study. Only your 'study ID' will be used – the samples will have none of your personal details on them. At the end of the study any remaining samples will be disposed of in accordance with the Human Tissue Act (2004).

For peer review only - http://bmjopen.bmj.com/site/about/guidelines.xhtml

NHS Manchester University NHS Foundation Trust

MANCHESTER 1824 The University of Manchester

NIHR | National Institute for Health Research

What happens if something goes wrong?
If you have a concern about any aspect of this study, you should ask to speak with the lead researchers who will do their best to answer your questions (**Dr. Basil Issa** or **Dr. Michelle Harvie – via the study office – via michelle.harvie@manchester.ac.uk or telephone 0161 291 4410**). If you remain unhappy and wish to complain formally, you can do so through the NHS complaints procedure. Details can be obtained from the NHS Patient and Liaison Service (PALS) on **Tel:** 0161 276 8686 or contact the team by email pals@mft.nhs.uk.

The hospital is insured to carry out clinical research through the NHS Indemnity scheme. If something did go wrong and you were harmed or suffered deterioration in your health as a result of taking part in this study then you may have grounds for legal action or compensation.

Additional information about the study

Will my lifestyle be affected if I take part?
An essential aspect of this study is a change to your diet and physical activity patterns with support from a specialist team of healthcare professionals.

Payments
We are able to offer free parking at Wythenshawe/St Marys Hospitals for study visits and offer reimbursement for reasonable travel expenses (car, bus or tram) linked to visits for this study. There are no other payments for taking part.

Will my details be kept confidential?
Yes. The study team and any associated regulatory authorities follow strict ethical and legal guidance regarding participant confidentiality. Any information we have about you will be handled in confidence and will only be used for the purposes of this study. All data recorded will be coded and your name will remain anonymous.

During the study we will inform your GP via letter of your participation in the study and your ongoing results, including your weight, blood tests, any abnormal findings and any recommendations for treatment.

If you join the study, some relevant parts of your medical records may be looked at by authorised personnel at Wythenshawe or St Marys hospitals prior to starting the study. These records may also be looked at by an independent auditing body and regulatory authorities to check that the study is being carried out correctly. We will only access parts of your medical records that are relevant to this research and all information accessed will be kept strictly confidential.

How will we use information about you?
We will need to use information from you and from your medical records for this research project.

For peer review only - http://bmjopen.bmj.com/site/about/guidelines.xhtml

This information will include the following:

- Initials
- NHS number
- Name
- Contact details
- Medical History including test results
- Demographic details

People will use this information to do the research or to check your records to make sure that the research is being done properly. People who do not need to know who you are will not be able to see your name or contact details. Your data will have a code number instead. We will keep all information about you safe and secure.

Once we have finished the study, we will keep some of the data so we can check the results. We will write our reports in a way that no-one can work out that you took part in the study. Other researchers from outside the Trust may ask to see this data for the purposes of furthering their research. We will only share this upon written request to the Trust. The external researchers will be asked to sign a Confidentiality Agreement before any data is shared.

What are your choices about how your information is used?
You can stop being part of the study at any time, without giving a reason, but we will keep information about you that we already have. If you choose to stop taking part in the study, we would like to continue collecting information about your health during pregnancy from your hospital records. If you do not want this to happen, tell us and we will stop. We need to manage your records in specific ways for the research to be reliable. This means that we won't be able to let you see or change the data we hold about you.

Where can you find out more about how your information is used?
You can find out more about how we use your information

- at https://research.cmft.nhs.uk/getting-involved/gdpr-and-research
- by asking one of the research team
- by sending an email to **mft.middas.gdm@nhs.net** or
- by ringing us on **07815987910**

How will my details be used to access the Mobile Applications?
None of your personal details (other than email from which an application is downloaded) will be needed to access the nutritics mobile applications. You can opt to have a 'dummy' e-mail and password under a pseudonym (fake name). Only the research team will know the dummy e-mail address you have been assigned to, in order to be able to review your data. The application will not contain your identifiable data. If you choose to use a mobile application to monitor your blood sugar levels the relevant terms of service for the app and the app developers privacy policy will apply. It will be your responsibility to read and understand these prior to download.

Will my insurance be affected if I take part in this study?

For peer review only - http://bmjopen.bmj.com/site/about/guidelines.xhtml

It is unlikely that your insurance premiums will be affected by participation in this study as the study has the potential to improve your diabetic control and reduce your risk of ill health. However, if you are at all concerned, then we advise that you contact your insurers and seek expert advice before agreeing to participate.

Who has reviewed this study?
Research in the NHS is looked at by an independent group of people called a Research Ethics Committee (REC). The REC is made up of experts, non-experts and members of the general public. Together they review research applications to ensure your safety, rights, wellbeing and dignity are protected at all times. This study has been reviewed and given favourable opinion by REC.

What will happen to the study results?
It is intended that the results of this study will be presented at conferences and published in medical journals so that we can explain to the medical community what our research results have shown. To do this our study information is double-checked by other professionals in research and healthcare. There is a possibility that the study and its results may be publicised for example on radio, television, magazines, books and websites. **You will not be identified in any publicity, reports or publication arising from this study.** If you would like a general summary of the results of the study you can select this on the consent form or please contact the research team.

Who is organising and funding the research?
Researchers from Wythenshawe hospital have designed this study and will be carrying out this research. This study has been funded by the National Institute of Health and Research.

Further information and contact details
For further information about this study, please contact **mft.middas.gdm@nhs.net** or **07815987910.**

Thank you for taking the time to read this information sheet.
We hope it has been of interest to you.

For peer review only - http://bmjopen.bmj.com/site/about/guidelines.xhtml

Manchester University
NHS Foundation Trust

MANCHESTER 1824
The University of Manchester

NIHR | National Institute for Health Research

Participant pathway for the study

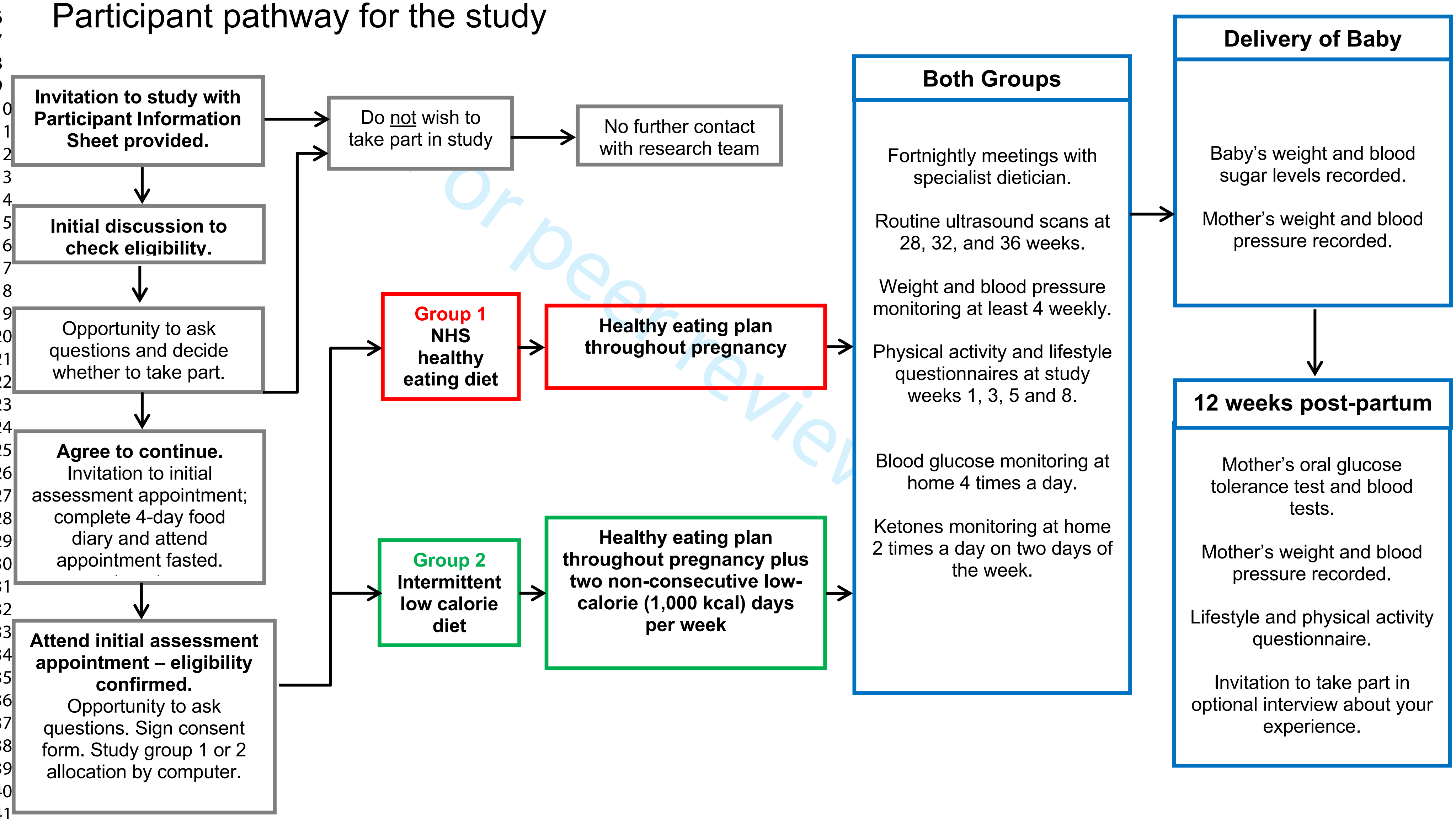

For peer review only - http://bmjopen.bmj.com/site/about/guidelines.xhtml

Consultant Endocrinologist – Dr. Basil Issa
Tel: 0161 291 7070
Research Dietitian – Dr. Michelle Harvie
Tel: 07815987910
Email: mft.middas.gdm@nhs.net

1st Floor Education and Research Centre
Manchester University NHS Foundation Trust
Wythenshawe Hospital
Manchester
M23 9LT

MIDDAS-GDM

Manchester Intermittent Diet in Gestational Diabetes Acceptability Study

Participant Informed Consent Form

Participant Identification Number:

Please **initial** box

1. I confirm that I have read and understand the participant information sheet (version …..) for the above study. I have had the opportunity to consider the information and ask questions, and have had these answered satisfactorily. ☐

2. I understand that my participation is voluntary and that I am free to withdraw at any time, without giving any reason, without my medical care or legal rights being affected. ☐

3. I understand that relevant sections of my medical notes and data collected during the study may be looked at by individuals from Manchester University NHS Foundation Trust and regulatory authorities, where it is relevant to my taking part in the research. I give permission for these individuals to have access to my records. ☐

4. I consent to the collection of blood samples to be collected as described in the participant information sheet. ☐

5. I understand that the information collected about me will be used to support other research in the future and may be shared anonymously with other researchers. ☐

6. I agree that my blood sugar readings can be recorded by the study team. ☐

7. I agree to my GP being informed of my participation in this study and changes to my weight, body measurements, blood results, questionnaire results and medications as required ☐

8. I understand that the information I provide to mobile applications as described in the Patient Information Sheet will be treated in line with the relevant terms of service and the app developers privacy policy at the time of downloading the application. ☐

For peer review only - http://bmjopen.bmj.com/site/about/guidelines.xhtml

Manchester University NHS Foundation Trust

MANCHESTER 1824 The University of Manchester

NIHR | National Institute for Health Research

9. I have informed the study team of any health issues, including those which may affect my ability to follow the diet, and I will inform the study team of any unusual symptoms that occur during the diet. I will inform the study team of changes to my health status during the study. ☐

10. I have informed the study team of any health issues, including those which may affect my ability to exercise, and I will inform the study team of changes to my health status during the study. ☐

11. I consent to the storage of personal information (including electronic) for the purposes of this study. I understand that any information that could identify me will be kept strictly confidential and that no personal information will be included in the study report or other publication. ☐

12. I agree to take part in the above study. ☐

13. I agree that relevant information about my pregnancy and/or gestational diabetes can be obtained from my medical records within the 18-month study duration if I withdraw from the study early. ☐

14. I am aware that my non-identifiable trial data may be shared with other researchers for the purposes of research. ☐

Optional (delete as appropriate)

15. I agree to be approached to take part in sub-study 1 (interview study), and understand that I will be approached to take part in the sub-study regardless of whether I withdraw from the main study YES/NO

16. I would like to receive a summary of the final study results YES/NO

17. I agree to be contacted regarding future research opportunities YES/NO

My preferred contact (*please tick and include email if preferred*)

Do not contact ☐ Post ☐ Email ☐ ______________________________

For peer review only - http://bmjopen.bmj.com/site/about/guidelines.xhtml

Signatures

……………………………..……….... ……………………..   ……………..………………
Name of participant   Date   Signature

……………………………………..……... …………………….   ……………...……………
Name of person taking consent   Date   Signature

When completed: 1 for participant; 1 for patient file; 1 for medical notes;; 1 (original) for site file

For peer review only

For peer review only - http://bmjopen.bmj.com/site/about/guidelines.xhtml

Self-monitoring schedule for capillary glucose and ketone monitoring

ILED		Best NHS Care	
**Ketones (low kcal days)**	**Glucose**	**Ketones (2 days/wk)**	**Glucose**
Fasting before breakfast the morning after each of the low-energy days	Fasting (morning)	Fasting before breakfast on 2 non-consecutive days / week	Fasting (morning)
1 hour post evening meal on each of the low-energy days	1hr post breakfast	1 hour post evening meal on 2 non-consecutive days / week	1hr post breakfast
	1hr post lunch		1hr post lunch
	1hr post dinner		1hr post dinner

For peer review only - http://bmjopen.bmj.com/site/about/guidelines.xhtml

Example of 1 day meal plan for Diet Day

The diet days aim to limit the calories to 1000 calories per day. You are aiming to include 2 (not consecutive) diet days each week. The other 5 days, follow the Mediterranean diet as described earlier. To keep the calories to 1000, the diet day will look like this:

Mixed diet		Vegetarian/ vegan diet
**4**	Carbohydrate portions	3
**6**	Protein portions	7
**5**	Vegetable portions	5
**2**	Fruit portions	2
**3**	Dairy portions	3
**1**	Fat portions	1

Below are some examples of meals that can be used to help you follow a 1000 calorie diet.. There are options for a mixed diet or vegan or vegetarian options, if you feel you wanted to try meat free days. Filling up on vegetables will make you feel less hungry

For peer review only

Mixed diet options

Breakfast	Portion	Dairy	Protein	Carb	Veg	Fruit	Fat
Grilled lean bacon	1 rasher	0	1	0	0	0	0
Grilled tomatoes	7 cherry tomatoes	0	0	0	1	0	0
Tea/ coffee	1 mug	0	0	0	0	0	0
Midmorning							
Diet or natural yogurt	1 small carton	1	0	0	0	0	0
Lunch							
Wholegrain bread	2 medium slices	0	0	2	0	0	0
Tuna	⅓ of a 120g can	0	1	0	0	0	0
Green salad	Cereal bowl full / 80 g with oil-free dressing	0	0	0	1	0	0
Satsumas	2	0	0	0	0	1	0
Mid afternoon							
Low fat cheese	30g / match box size	1	0	0	0	0	0
Apple slices	I medium apple (80g)	0	0	0	0	1	0
Tea/ coffee		0	0	0	0	0	0
Evening							
Vegetable rice	4 tablespoons cooked rice 160g of mix vegetables	0	0	2	2	0	0
Chicken curry	90g /average chicken breast (no skin) & ½ can tomatoes, 1 desertspoon oil	0	3	0	1	0	1
Bedtime							
Low fat houmous	1 level tablespoon	0	1	0	0	0	0
Pepper sticks	½ red pepper	0	0	0	1	0	0
Milk	1 small glass	1	0	0	0	0	0
**Total portions a day**		**3 portions**	**6 portions**	**4 portions**	**5 portions**	**2 portions**	**1 portion**

Vegetarian option

Breakfast		Dairy	Protein	Carb	Veg	Fruit	Fat
Egg	2 poached	0	2	0	0	0	0
Mushrooms	2 cupped handfuls / 80g	0	0	0	1	0	0
Tea/ coffee	1 mug	0	0	0	0	0	0
Midmorning							
Cheddar cheese	1 match box size / 30g	1	0	0	0	0	0
Cucumber	Sliced handful	0	0	0	1	0	0
Lunch							
Baked beans	2 tablespoons	0	1	0	0	0	0
Seeded bread toasted	1 medium sliced	0	0	1	0	0	0
Blueberries	1 handful	0	0	0	0	1	0
Mid afternoon							
Meat free ham	2 slice small	0	1	0	0	0	0
Pepper	½ sliced	0	0	0	1	0	0
Avocado	¼	0	0	0	0	0	1
Tea/ coffee	1 mug	0	0	0	0	0	0
Evening							
Vegetarian sausage casserole Jacket potato (100g)	1 grilled sausage 2 cereal bowls vegetables 1 ½ egg sized (100 g)	0	2	1	2	0	0
Bedtime							
Pear	1 medium	0	0	0	0	1	0
Low fat cream cheese	1 tablespoon	1	0	0	0	0	0
Whole wheat cracker	2 biscuits	0	0	1	0	0	0
Milk	1 small glass	1	1	0	0	0	0
**Total portions a day**		**3 portions**	**7 portions**	**3 portions**	**5 portions**	**2 portions**	**1 portions**

Vegan options

Breakfast		Dairy equivalent	Protein	Carb	Veg	Fruit	Fat
Branflakes	3 tablespoons	0	0	1	0	0	0
Milk- soya	200 ml	1	0	0	0	0	0
Tea/ coffee	1 mug	0	0	0	0	0	0
Midmorning							
Soya yogurt	3 tablespoons	1	0	0	0	0	0
Lunch							
Kidney bean & Vegetable chilli<br><br>Wholemeal pitta	3 tablespoons of beans 60g with 1 cereal bowl mixed vegetables & 1/2 can chopped tomatoes<br>½ pitta	0	2	1	2	0	0
Banana	1 medium	0	0	0	0	1	0
Mid afternoon							
Low fat hummus	2 level tablespoon	0	2	0	0	0	0
1 carrot	I medium carrot (80g)	0	0	0	1	0	0
Tea/ coffee	1 mug	0	0	0	0	0	0
Evening							
Quinoa	2 tablespoon cooked	0	0	1	0	0	0
Tofu	4 matchbox	0	2	0	0	0	0
Mixed salad with edamame beans	2 x Cereal bowl full with oil free dressing & 1 tablespoons of edamame	0	1	0	2	0	0
Bedtime							
Peanut butter	1 heaped teaspoon	0	0	0	0	0	1
Apple	1 medium sliced	0	0	0	0	1	0
Milk	1 small glass	1	0	0	0	0	0
**Total portions a day**		**3 portions**	**7 portions**	**2 portions**	**5 portions**	**2 portions**	**1 portions**

To help with estimation of portions the following tables outline weight and measures of the different food groups. Where possible household measures are given to make things a little easier. Use these to help you plan your 2 days in the week of 1000 calories.

**Carbohydrate**<br>**4 portions - mixed diet**<br>**3 portions - vegan/vegetarian**	**Equal to**
Wholewheat or oat breakfast cereal, e.g. wholewheat biscuit, malted wholewheat squares, Grapenuts, bran flakes, fruit & fibre	24g or 3 tablespoons or<br>1 whole wheat biscuit
Porridge oats or no-added sugar muesli	20g or 1 heaped tablespoon
Wholegrain, wholemeal, rye, granary bread	36g or medium slice of bread (other than rye), 1½ slices of rye, or ½ roll
Wholemeal or multigrain pitta bread or tortilla wrap, chapatti made without fat	60g or 1x 8" tortilla<br>or 1 standard pitta<br>or small thin chapatti
Rye crispbread, crackers, oak cakes	22g or 2 crispbreads/ 2 oatcakes
Wholegrain rice cake	16g or 2 rice cakes
Wholewheat pasta or rice - cooked amount<br><br>Cous cous, Bulgar wheat, Quinoa, Pearl barley	1 tablespoon uncooked 2 tablespoons cooked<br><br>30g- raw weight or 60g cooked
Lasagne (wholemeal if possible)	20g raw weight or<br>1 large sheet or 1½ smaller sheets
Noodles (wholemeal if possible)	25g raw weight or ½ block/nest
Baked or boiled potato (in skin), cassava, sweet potato	1½ egg sized potatoes or<br>100g raw weight
Wholemeal pizza base (topping is from other food groups)	35g or $^{1}/_{6}$ of thin 10" pizza base
Unsweetened popcorn	20g or 2 handfuls

For peer review only

**Protein**<br>**6 portions – mixed diet**<br>**7 portions – vegan/vegetarian**	**Equal to**
Fresh or smoked white fish (e.g. haddock or cod)	60g or 2oz<br>2 fish finger size
Seafood, e.g. prawns, mussels, crab	45g or 1½oz
Canned tuna or salmon in brine or spring water	45g or 1½oz<br>⅓ standard tin (120g)
Oily fish (fresh or tinned in tomato sauce or olive oil - drained), e.g. mackerel, sardines, salmon, fresh tuna, kippers, smoked salmon or trout	30g or 1oz or<br>¼ standard tin (120g) or<br>¼ fillet of salmon
Chicken, turkey, duck, pheasant (cooked without skin)<br>Lean beef, pork, lamb, rabbit, venison, offal (fat removed)<br>**Quorn fillets, steak, mince or pieces**<br>**Vegetarian mince frozen**	30g or 1oz or<br>1 slice size of playing card
Lean grilled bacon<br>**Quorn ham**	25g or ¾oz or<br>1 rasher
Lean ham<br>**Quorn bacon rashers (not slices)**	30g or 1oz or<br>1 medium, 2 small or 4 wafer thin slices
Eggs	60 g or 2 oz or<br>1 egg
Tofu	50g or $1^{2}/_{3}$ oz or<br>Size of 2 match boxes
Tempeh	25 g or 1 oz or<br>Size of 1 match box
Baked beans (reduced sugar)	60 g or 2 oz or<br>2 tablespoons
Lentils, chickpeas and kidney beans, mung beans, black eye beans, puy lentils, toor dahl, urad dahl,<br>Raw weight<br><br>Cooked or tinned weight	20g or ⅔ oz or<br>1 tablespoon raw<br><br>65g or 2oz or<br>1½ tablespoons cooked /tinned or 1 cupped handful
Soya beans (frozen or cooked) or edamame beans	30g or 1oz or<br>1 tablespoon
Vegetarian sausage	25g or ¾ oz or ½ sausage
Textured vegetable protein (TVP)	10g or ⅓ oz uncooked or<br>1 heaped tablespoon uncooked
Low fat hummus	30g or 1oz or<br>1 level tablespoon

For peer review only - http://bmjopen.bmj.com/site/about/guidelines.xhtml

**Vegetables – min 5 portions**<br>**1 portion = 80g or 2⅔oz**	**1 portion is equal to**
Asparagus, Aubergines, Broccoli, Brussel sprouts, Carrots, Cabbage, Cauliflower, Chinese leaves, Courgettes, Cucumber, Curly kale, Green beans, Lettuce (mixed leaves), Mange tout, Methi, Mushrooms, Okra, Pak choi, Peas, Sugar snap, Spinach, Spring greens cooked, Sweetcorn, Tomatoes, Watercress fresh	80g or 2 ⅔ oz or 2 spears of broccoli, 8 cauliflower florets. 3 heaped tablespoons of vegetables or large cereal bowl of salad.

**Fruit - 2 portions.**<br>**1 portion = 80g or 2⅔oz (30g or 1oz dried fruits)**	**1 portion is equal to**
Berries (e.g. blackberries, blueberries, redcurrants, raspberries, strawberries)<br>Cherries or grapes	80g or 2⅔oz<br>1 handful
Grapefruit, guava and mango	80g or 2⅔oz or<br>½ a whole fruit
Large fruit (e.g. melon, pineapple, papaya)	80g or 2⅔oz or<br>1 medium slice
Medium fruits (e.g. apple, pear, nectarine, orange, peach, banana, pomegranate)	80g or 2⅔oz<br>1 fruit
Small fruit (e.g. fresh apricots, kiwi, clementine, passion fruit, plums)	80g or 2⅔oz or<br>2 fruits
Any stewed fruit—unsweetened or with calorie-free sweetener e.g. apple, rhubarb	80g or 2⅔oz or<br>3 tablespoons
Kumquats, lychees, physalis	5 fruits
Dried fruits (raisins, currants, apricots)	30g or 1oz or<br>1 tablespoon

**Milk and dairy foods - 3 portions**	**Equal to**
Milk (semi-skimmed or skimmed)<br>Alternative 'milks' with added calcium, e.g. soya **	⅓ pint or 200ml or<br>1 small glass
Diet yoghurts, Low fat/fat-free Greek or Greek Style or natural yoghurts, fromage frais or plain soya yoghurt, high protein yogurt	120-150g or 4-5oz or<br>1 small pot or<br>3 tablespoons
Whole milk natural yogurt	80g or 1 ⅔ oz or<br>2 tablespoons
Cottage cheese	75g or 1½oz or<br>¼ pot, 2 tablespoons
Cream cheese (light or extra light)	30g or 1oz or<br>1 tablespoon
Lower fat hard cheeses e.g.:<br>Reduced fat cheddar, Edam,<br>Bavarian smoked, feta, ricotta, mozzarella, reduced fat halloumi, paneer made from semi-skimmed milk	30g or 1oz or Matchbox size<br>No more than 180g or 6oz a week

** we recommend soya milk as coconut, oat and almond milks are lower in protein and calcium

For peer review only

**Fat – 2 portions only**	**Equal to**
Margarine or low-fat spread (avoid the buttery types)<br>Olive oil or other oil<br>Oil based dressing<br>Pesto<br>Mayonnaise	8g or 1 teaspoon<br>1 dessertspoon of oil
Seeds (e.g. linseed, pumpkin, sunflower, sesame, chia, hemp)	7g or 1 dessertspoon
Unsalted or salted or dry roasted nuts	7g or 1 dessertspoon<br>3 walnut halves, 3 Brazil,<br>4 almonds, 8 peanuts,<br>10 cashews or pistachios
Olives	50g or 10 olives
Low fat mayonnaise, Curry paste or Harissa paste	15g or ½ oz or 1 tablespoon
Peanut butter (without palm oil)	11g or ⅓ oz or<br>1 heaped teaspoon
Cocoa powder	12 g or ⅓ oz or<br>2 heaped teaspoons
Avocado	40g or 1⅓ oz or<br>1/4 of an average pear
Low fat guacamole	40g or 1⅓ oz or 2 tablespoons

For peer review only

Medical Management Protocols

Hypoglycaemia

Participants will be advised to take 15-20g of rapid acting carbohydrate in the event of hypoglycaemia, (defined as blood glucose <4 mmol/L) which is anticipated to raise blood glucose by 3 mmol/L. Examples of rapid acting carbohydrate include 170-225ml Lucozade Original (not Lucozade Sport), a small carton of fruit juice, 5-6 glucose tablets, 4/5 jelly babies, or a small tin of cola (150-200ml). Participants will be advised to repeat the treatment every 15 minutes until blood glucose is ≥4 mmol/l. The following table highlights when participants should consider taking additional follow-up slower acting carbohydrate:

Situation	Acceptable slow acting carbohydrate
Less than 1 hour before the next meal	Try and avoid
1-2 hour before the next meal	10g
More than 2 hours before the next meal	15-20g

Ketonaemia

Ketone levels ≥1.0 mmol/L on a fasting sample:

- Drink 1L fluids and repeat ketone levels after 4 hours.
- If ketone level has improved (<1.0mmol/L), no further action required.
- If ketone level has increased or remains the same, repeat ketone level after 2 hours.
- If ketone level is persistently increased, consume 40g carbohydrates and repeat in 2 hours.
- Continue to do this until ketone levels <1.0mmol/L.

If a participant experiences >2 episodes of the above throughout the course of the study their notes will be reviewed by the PI and their suitability for remaining in the trial will be assessed.

For peer review only

Guidance for the introduction of diabetes medication (week 24-delivery)

Diabetes medication will be introduced according to the following protocol:

- If ≥25% fasting blood glucose readings are >5 mmol/l and/or ≥25% of 1 hour postprandial glucose readings are >7 mmol/l in a 7 day period: commence Metformin MR 500 mg daily to be increased every 3 days by 500 mg to 1 gram BD if tolerated.

- If after reaching optimal or maximum tolerated dose Metformin ≥25% fasting blood glucose readings are >5 mmol/l in a 7-day period: commence bedtime isophane insulin 4 units and uptitrate the dose by 2 units every 3 days aiming for a fasting glucose of ≤5 mmol/l,

- and/or ≥25% of 1 hour postprandial glucose readings are >7 mmol/l in a 7 day period: commence prandial fast acting insulin analogue (Humalog or Novorapid) 2-4 units with the relevant meal. Uptitrate the dose by 2 units every 3 days aiming for a 1 hour postprandial glucose of ≤7 mmol/l.

- Medication adjustment will be made in accordance with the above guidance.

For peer review only

NHS
Manchester University
NHS Foundation Trust

MIDDAS-GDM End of Study Questionnaire

Thank you for taking part in the MIDDAS-GDM Study.

This is one of the first studies of its kind. We hope to learn as much as possible from this study, in particular the views of people who have taken part. We are inviting you to provide your views on different aspects of the study and following the diet, and how we can improve our programmes and research studies in future.

Please complete the following questions and return this questionnaire to the MIDDAS-GDM study team in the envelope provided. If there is anything else you would like to say about your experiences of the study, please use the section at the end. Your answers to the questions below will remain anonymous.

1. What were your reasons for joining the study?

………
………
………
………
………

2. How satisfied were you with study overall (recruitment, appointments etc)? *(circle)*

1	2	3	4	5	6	7	8	9	10
Not at all satisfied		Slightly satisfied		Quite satisfied		Very satisfied			Extremely satisfied

Comments:…………………………………………………………………………………………………
………
………

Study Appointments

3. You were asked to attend ……….. additional face to face appointments at the hospital by the study team *(please fill in the number)*

For peer review only - http://bmjopen.bmj.com/site/about/guidelines.xhtml

Manchester University
NHS Foundation Trust

4. **You were asked to attend ……….. additional virtual (i.e. video call or telephone) appointments by the study team** *(please fill in the number)*

5. **How do you feel about the number of additional appointments you were asked to attend?** *(tick)*

☐ I was happy with the number of appointments
☐ I would have preferred fewer face to face appointments
☐ I would have preferred more face to face appointments
☐ I would have preferred fewer virtual appointments
☐ I would have preferred more virtual appointments

Comments:…………………………………………………………………………………………………
……

6. **How do you feel about virtual (i.e. video call or telephone) appointments?**

☐ I prefer virtual appointments to face to face appointments (please explain why below)
☐ I prefer face to face appointments to virtual appointments (please explain why below)

Comments:…………………………………………………………………………………………………
……
……
……

For peer review only

Diet

7. **Which diet were you asked to follow? (*please tick*)**

☐ Best NHS Care (i.e. increased fruit/vegetable intake, low-GI foods, reduction in free sugars, regular meals)
☐ Intermittent low energy diet (5 days of the best NHS care diet plus 2 non-consecutive days of 1000 kcal each week)

8. **Was the diet easy to follow?**

1	2	3	4	5	6	7	8	9	10
Not at all		Slightly		Moderately		Very			Extremely

For peer review only - http://bmjopen.bmj.com/site/about/guidelines.xhtml

NHS
Manchester University
NHS Foundation Trust

Comments:……………………………………………………………………………………………
……………………………………………………………………………………………………

9. Did you enjoy following the diet plan?

1	2	3	4	5	6	7	8	9	10
Not at all		Slightly		Moderately		Very			Extremely

Comments:……………………………………………………………………………………………
……………………………………………………………………………………………………

10. Would you make any changes to the written information (i.e. diet booklets, recipes) you were given on how to follow the diet plan?

☐ Yes

☐ No

If yes, what changes would you make?

Comments:……………………………………………………………………………………………
……………………………………………………………………………………………………

11. How was your first appointment with the dietitian? *(tick all that apply)***?**

☐ The amount of information I received was OK

☐ The amount of information I received was too little

☐ The amount of information I received was too much

☐ I was happy with the advice I received

☐ The advice I received could be improved (specify below)

Comments:……………………………………………………………………………………………
……………………………………………………………………………………………………

For peer review only - http://bmjopen.bmj.com/site/about/guidelines.xhtml

Manchester University
NHS Foundation Trust

12. Would you make any changes to the reviews (calls/face to face appointments) you had with the dietitian during your pregnancy ?

☐ Yes

☐ No

Comments:…………………………………………………………………………………………

………………………………………………………………………………………………………

13. How useful was your final appointment with the dietitian at 12 weeks post-delivery?

1	2	3	4	5	6	7	8	9	10
Not at all		Slightly		Quite		Very			Extremely

Comments:…………………………………………………………………………………………

………………………………………………………………………………………………………

14. Did you feel confident to exercise whilst on the diet plan?

1	2	3	4	5	6	7	8	9	10
Not at all confident		Slightly confident		Quite confident		Very confident			Extremely confident

Comments:…………………………………………………………………………………………

………………………………………………………………………………………………………

Additional support

15. Would any of the following have been useful & if so how often?

	No	Yes	Preferred method of contact	How often
Additional support from dietitian	☐	☐	Face to face / phone	
Additional support from the midwife	☐	☐	Face to face / phone	

For peer review only - http://bmjopen.bmj.com/site/about/guidelines.xhtml

Manchester University
NHS Foundation Trust

Additional support from the doctors in the clinic	☐	☐	Face to face / phone	
More contact with other women in the study following the diets	☐	☐	Face to face / phone	
Other, please specify:				

16. Did you receive any support outside of the study team help to keep you on track as you progressed through the study?

☐ No

☐ Yes

If yes, what support did you receive?

……………………………………………………………………………………………………

……………………………………………………………………………………………………

Record keeping

17. How did you find the finger prick testing requirements on the study? *(tick all those that apply)*

☐ Challenging but on the whole achievable

☐ Challenging and not achievable

☐ Not challenging at all

☐ I felt it was necessary to test this often to ensure my safety

☐ I felt it was unnecessary to test this often to ensure my safety

Comments:……………………………………………………………………………………

……………………………………………………………………………………………………

18. How did you find the ketone testing requirements on the study? *(tick all those that apply)*

☐ Challenging but on the whole achievable

☐ Challenging and not achievable

For peer review only

For peer review only - http://bmjopen.bmj.com/site/about/guidelines.xhtml

Manchester University
NHS Foundation Trust

☐ Not challenging at all

☐ I felt it was necessary to test this often to ensure my safety

☐ I felt it was unnecessary to test this often to ensure my safety

Comments:………………………………………………………………………………………

……………………………………………………………………………………………...............

19. How did you find using Diasend software?

☐ Straightforward

☐ Challenging but on the whole achievable

☐ Challenging and not achievable

☐ I felt uncomfortable using computer software to keep track of my medical details

☐ I felt comfortable using computer software to keep track of my medical details

Comments:………………………………………………………………………………………

……………………………………………………………………………………………...............

20. How did you find completing the food diary during the study? *(tick all those that apply)*

☐ Challenging but on the whole achievable

☐ Challenging and not achievable

☐ Not challenging at all

Comments:………………………………………………………………………………………

……………………………………………………………………………………………...............

21. How did you find the physical activity questionnaires on the study? *(tick all those that apply)*

☐ Challenging but on the whole achievable

☐ Challenging and not achievable

☐ Not challenging at all

Comments:………………………………………………………………………………………

……………………………………………………………………………………………...............

For peer review only

For peer review only - http://bmjopen.bmj.com/site/about/guidelines.xhtml

22. How did you find the quality of life questionnaires on the study? *(tick all those that apply)*

☐ Challenging but on the whole achievable

☐ Challenging and not achievable

☐ Not challenging at all

Comments:……………………………………………………………………………………………

………

Libro® app

23. Did you use the Libro® app?

☐ Yes

☐ No (please move to question 25)

24. Did you find the App helpful?

1	2	3	4	5	6	7	8	9	10
Not at all		Slightly		Moderately		Very			Extremely

Comments……………………………………………………………………………………………

………

25. What did you like about the App?

………

………

………

26. What did you dislike about the App and could be improved?

………

………

27. If you didn't use the app what were the reasons for this? *(tick all that apply)*

☐ Would prefer not to say ☐ Already using other health apps

For peer review only - http://bmjopen.bmj.com/site/about/guidelines.xhtml

Manchester University NHS Foundation Trust

☐ Don't like using apps in general ☐ Not user friendly

☐ Labour intensive / time consuming ☐ Prefer to use pen and paper

☐ Find mobile devices challenging

☐ Lack of regular internet access

☐ Other (provide details below)

……………………………………………………………………………………………………

……………………………………………………………………………………………………

……………………………………………………………………………………………………

Diasend Software

28. Did you find the Diasend software helpful?

1	2	3	4	5	6	7	8	9	10
Not at all		Slightly		Moderately		Very			Extremely

Comments……………………………………………………………………………………………

……………………………………………………………………………………………………

29. What did you like about the Diasend software?

……………………………………………………………………………………………………

……………………………………………………………………………………………………

30. What did you dislike about the Diasend software and could be improved?

……………………………………………………………………………………………………

……………………………………………………………………………………………………

Study Improvements

31. What did you enjoy most about the study?

……………………………………………………………………………………………………

……………………………………………………………………………………………………

……………………………………………………………………………………………………

……………………………………………………………………………………………………

For peer review only

For peer review only - http://bmjopen.bmj.com/site/about/guidelines.xhtml

Manchester University
NHS Foundation Trust

32. What did you enjoy least about the study and could be improved?

……………………………………………………………………………………………
……………………………………………………………………………………………
……………………………………………………………………………………………
……………………………………………………………………………………………
……………………………………………………………………………………………

Any other comments about the study

……………………………………………………………………………………………
……………………………………………………………………………………………
……………………………………………………………………………………………
……………………………………………………………………………………………
……………………………………………………………………………………………
……………………………………………………………………………………………
……………………………………………………………………………………………
……………………………………………………………………………………………

Thank you for completing this questionnaire, please return to:

MIDDAS-GDM Study Team, Nightingale Centre,

Wythenshawe Hospital, Manchester, M23 9LT

For peer review only - http://bmjopen.bmj.com/site/about/guidelines.xhtml

SPIRIT CHECKLIST

Section/Item	Item no
Title	1
Trial Registration	2a
	2b
Protocol Version	3
Funding	4
Roles and responsibilities	5a
	5b
	5c
	5d
**Introduction**	
Background and rationale	6a
	6b
Objectives	7
Trial Design	8
**Methods: Participants, interventions, and outcomes**	
Study setting	9
Eligibility criteria	10
Interventions	11a

For peer review only

	11b
	11c
	11d
Outcomes	12
Participant Timeline	13
Sample Size	14
Recruitment	15
**Methods: Assignment of interventions (for controlled tri**	
Allocation	
Sequence generation	16a
Allocation concealment mechanism	16b
Implementation	16c

For peer review only

Blinding (masking)	17a
	17b
**Methods: Data collection, management, and analysis**	
Data collection methods	18a
	18b
Data management	19
Statistical methods	20a
	20b
	20c
**Methods: Monitoring**	
Data monitoring	21a

For peer review only

	21b
Harms	22
Auditing	23
**Ethics and dissemination**	
Research ethics approval	24
Protocol amendments	25
Consent or assent	26a
	26b
Confidentiality	27
Declaration of interests	28
Access to data	29
Ancillary and post-trial care	30
Dissemination policy	31a

For peer review only

	31b
	31c
**Appendices**	
Informed consent materials	32
Biological specimens	33

For peer review only

Description	Location
Descriptive title identifying the study design, population, interventions, and, if applicable, trial acronym	1
Trial identifier and registry name. If not yet registered, name of intended registry	3
All items from the World Health Organization Trial Registration Data Set	throughout
Date and version identifier	n/a
Sources and types of financial, material, and other support	23
Names, affiliations, and roles of protocol contributors	1
Name and contact information for the trial sponsor	name only, 23
Role of study sponsor and funders, if any, in study design; collection, management, analysis, and interpretation of data; writing of the report; and the decision to submit the report for publication, including whether they will have ultimate authority over any of these activities	23
Composition, roles, and responsibilities of the coordinating centre, steering committee, endpoint adjudication committee, data management team, and other individuals or groups overseeing the trial, if applicable (see Item 21a for data monitoring committee)	18
Description of research question and justification for undertaking the trial, including summary of relevant studies (published and unpublished) examining benefits and harms for each intervention	4-5
Explanation for choice of comparators	4-5
Specific objectives or hypotheses	13-14
Description of trial design including type of trial (eg, parallel group, crossover, factorial, single group), allocation ratio, and framework (eg, superiority, equivalence, noninferiority, exploratory)	6
Description of study settings (eg, community clinic, academic hospital) and list of countries where data will be collected. Reference to where list of study sites can be obtained	6
Inclusion and exclusion criteria for participants. If applicable, eligibility criteria for study centres and individuals who will perform the interventions (eg, surgeons, psychotherapists)	7
Interventions for each group with sufficient detail to allow replication, including how and when they will be administered	9

For peer review only

Criteria for discontinuing or modifying allocated interventions for a given trial participant (eg, drug dose change in response to harms, participant request, or improving/worsening disease)	n/a
Strategies to improve adherence to intervention protocols, and any procedures for monitoring adherence (eg, drug tablet return, laboratory tests)	13
Relevant concomitant care and interventions that are permitted or prohibited during the trial	7
Primary, secondary, and other outcomes, including the specific measurement variable (eg, systolic blood pressure), analysis metric (eg, change from baseline, final value, time to event), method of aggregation (eg, median, proportion), and time point for each outcome. Explanation of the clinical relevance of chosen efficacy and harm outcomes is strongly recommended	13-14
Time schedule of enrolment, interventions (including any run-ins and washouts), assessments, and visits for participants. A schematic diagram is highly recommended (see Figure)	11-12
Estimated number of participants needed to achieve study objectives and how it was determined, including clinical and statistical assumptions supporting any sample size calculations	8
Strategies for achieving adequate participant enrolment to reach target sample size	n/a
**als)**	
Method of generating the allocation sequence (eg, computer-generated random numbers), and list of any factors for stratification. To reduce predictability of a random sequence, details of any planned restriction (eg, blocking) should be provided in a separate document that is unavailable to those who enrol participants or assign interventions	8
Mechanism of implementing the allocation sequence (eg, central telephone; sequentially numbered, opaque, sealed envelopes), describing any steps to conceal the sequence until interventions are assigned	8
Who will generate the allocation sequence, who will enrol participants, and who will assign participants to interventions	7-8

For peer review only

Who will be blinded after assignment to interventions (eg, trial participants, care providers, outcome assessors, data analysts), and how	8
If blinded, circumstances under which unblinding is permissible, and procedure for revealing a participant's allocated intervention during the trial	8
Plans for assessment and collection of outcome, baseline, and other trial data, including any related processes to promote data quality (eg, duplicate measurements, training of assessors) and a description of study instruments (eg, questionnaires, laboratory tests) along with their reliability and validity, if known. Reference to where data collection forms can be found, if not in the protocol	9-15
Plans to promote participant retention and complete follow-up, including list of any outcome data to be collected for participants who discontinue or deviate from intervention protocols	supplementary PIS
Plans for data entry, coding, security, and storage, including any related processes to promote data quality (eg, double data entry; range checks for data values). Reference to where details of data management procedures can be found, if not in the protocol	16
Statistical methods for analysing primary and secondary outcomes. Reference to where other details of the statistical analysis plan can be found, if not in the protocol	16
Methods for any additional analyses (eg, subgroup and adjusted analyses)	16
Definition of analysis population relating to protocol non-adherence (eg, as randomised analysis), and any statistical methods to handle missing data (eg, multiple imputation)	16
Composition of data monitoring committee (DMC); summary of its role and reporting structure; statement of whether it is independent from the sponsor and competing interests; and reference to where further details about its charter can be found, if not in the protocol. Alternatively, an explanation of why a DMC is not needed	18

For peer review only

Description of any interim analyses and stopping guidelines, including who will have access to these interim results and make the final decision to terminate the trial	n/a
Plans for collecting, assessing, reporting, and managing solicited and spontaneously reported adverse events and other unintended effects of trial interventions or trial conduct	13, 16
Frequency and procedures for auditing trial conduct, if any, and whether the process will be independent from investigators and the sponsor	16
Plans for seeking research ethics committee/institutional review board (REC/IRB) approval	n/a
Plans for communicating important protocol modifications (eg, changes to eligibility criteria, outcomes, analyses) to relevant parties (eg, investigators, REC/IRBs, trial participants, trial registries, journals, regulators)	19
Who will obtain informed consent or assent from potential trial participants or authorised surrogates, and how (see Item 32)	7, supplementary consent
Additional consent provisions for collection and use of participant data and biological specimens in ancillary studies, if applicable	n/a
How personal information about potential and enrolled participants will be collected, shared, and maintained in order to protect confidentiality before, during, and after the trial	16
Financial and other competing interests for principal investigators for the overall trial and each study site	23
Statement of who will have access to the final trial dataset, and disclosure of contractual agreements that limit such access for investigators	19
Provisions, if any, for ancillary and post-trial care, and for compensation to those who suffer harm from trial participation	supplementary PIS
Plans for investigators and sponsor to communicate trial results to participants, healthcare professionals, the public, and other relevant groups (eg, via publication, reporting in results databases, or other data sharing arrangements), including any publication restrictions	19

For peer review only

Authorship eligibility guidelines and any intended use of professional writers	n/a
Plans, if any, for granting public access to the full protocol, participant- level dataset, and statistical code	19
Model consent form and other related documentation given to participants and authorised surrogates	supplementary materials
Plans for collection, laboratory evaluation, and storage of biological specimens for genetic or molecular analysis in the current trial and for future use in ancillary studies, if applicable	15

For peer review only

Missing

For peer review only

For peer review only

For peer review only

form

For peer review only

For peer review only

MeSH Descriptors

Infant, newborn
Pregnancy
Glucose Intolerance
Diabetes, Gestational
Insulin
Hyperglycaemia
Prediabetic state
Metformin
Glycated Hemoglobin
Overweight
Obesity
Diabetes Mellitus, Type 2
Diet, Healthy
Feasibility Studies
Mobile Applications
Body Weight
Hypoglycaemic agents
Fasting
Intermittent Fasting

For peer review only

TIDieR
Template for Intervention Description and Replication

The TIDieR (Template for Intervention Description and Replication) Checklist*:

Information to include when describing an intervention and the location of the information

Item number	Item	Where located ** Primary paper (page or appendix number)	Other † (details)
	**BRIEF NAME**		
**1.**	Provide the name or a phrase that describes the intervention.	2	
	**WHY**		
**2.**	Describe any rationale, theory, or goal of the elements essential to the intervention.	4-5	______________
	**WHAT**		
**3.**	Materials: Describe any physical or informational materials used in the intervention, including those provided to participants or used in intervention delivery or in training of intervention providers. Provide information on where the materials can be accessed (e.g. online appendix, URL).	12, 15	_appendix____
**4.**	Procedures: Describe each of the procedures, activities, and/or processes used in the intervention, including any enabling or support activities.	6-12	______________
	**WHO PROVIDED**		
**5.**	For each category of intervention provider (e.g. psychologist, nursing assistant), describe their expertise, background and any specific training given.	1, 7-12, 17-18	______________
	**HOW**		

For peer review only

For peer review only - http://bmjopen.bmj.com/site/about/guidelines.xhtml

**6.**	Describe the modes of delivery (e.g. face-to-face or by some other mechanism, such as internet or telephone) of the intervention and whether it was provided individually or in a group.	6-18	______________
	**WHERE**		
**7.**	Describe the type(s) of location(s) where the intervention occurred, including any necessary infrastructure or relevant features.	6-18	______________
	**WHEN and HOW MUCH**		
**8.**	Describe the number of times the intervention was delivered and over what period of time including the number of sessions, their schedule, and their duration, intensity or dose.	6-18	______________
	**TAILORING**		
**9.**	If the intervention was planned to be personalised, titrated or adapted, then describe what, why, when, and how.	6-18	______________
	**MODIFICATIONS**		
**10.‡**	If the intervention was modified during the course of the study, describe the changes (what, why, when, and how).	N/A	______________
	**HOW WELL**		
**11.**	Planned: If intervention adherence or fidelity was assessed, describe how and by whom, and if any strategies were used to maintain or improve fidelity, describe them.	13-14	______________
**12.‡**	Actual: If intervention adherence or fidelity was assessed, describe the extent to which the intervention was delivered as planned.	N/A	______________

For peer review only

For peer review only - http://bmjopen.bmj.com/site/about/guidelines.xhtml

** **Authors** - use N/A if an item is not applicable for the intervention being described. **Reviewers** – use '?' if information about the element is not reported/not sufficiently reported.

† If the information is not provided in the primary paper, give details of where this information is available. This may include locations such as a published protocol or other published papers (provide citation details) or a website (provide the URL).

‡ If completing the TIDieR checklist for a protocol, these items are not relevant to the protocol and cannot be described until the study is complete.

* We strongly recommend using this checklist in conjunction with the TIDieR guide (see *BMJ* 2014;348:g1687) which contains an explanation and elaboration for each item.

* The focus of TIDieR is on reporting details of the intervention elements (and where relevant, comparison elements) of a study. Other elements and methodological features of studies are covered by other reporting statements and checklists and have not been duplicated as part of the TIDieR checklist. When a **randomised trial** is being reported, the TIDieR checklist should be used in conjunction with the CONSORT statement (see www.consort-statement.org) as an extension of **Item 5 of the CONSORT 2010 Statement.** When a **clinical trial protocol** is being reported, the TIDieR checklist should be used in conjunction with the SPIRIT statement as an extension of **Item 11 of the SPIRIT 2013 Statement** (see www.spirit-statement.org). For alternate study designs, TIDieR can be used in conjunction with the appropriate checklist for that study design (see www.equator-network.org).

For peer review only

For peer review only - http://bmjopen.bmj.com/site/about/guidelines.xhtml
